# Ocean Species Discoveries 13–27 — Taxonomic contributions to the diversity of Polychaeta, Mollusca and Crustacea

**DOI:** 10.3897/BDJ.13.e160349

**Published:** 2025-10-15

**Authors:** Senckenberg Ocean Species Alliance (SOSA), Luiz F. Andrade, Christopher B. Boyko, Angelika Brandt, Barbara Buge, Yasmín Dávila Jiménez, Mats Henseler, Pablo Hernández Alcántara, Piotr Jóźwiak, Henry Knauber, Fabrizio Marcondes Machado, Carlos A. Martínez-Muñoz, Farzaneh Momtazi, Yumi Nakadera, Jian-Wen Qiu, Torben Riehl, Greg W. Rouse, Julia D. Sigwart, Boris Sirenko, Jesser F. Souza-Filho, Jan Steger, Anna Stępień, Ekin Tilic, Bianca Trautwein, Katarzyna Vončina, Jason D. Williams, Junlong Zhang

**Affiliations:** 1 Senckenberg Research Institute and Natural History Museum Frankfurt, Department of Marine Zoology, Frankfurt am Main, Germany Senckenberg Research Institute and Natural History Museum Frankfurt, Department of Marine Zoology Frankfurt am Main Germany; 2 University of Lodz, Faculty of Biology and Environmental Protection, Department of Invertebrate Zoology and Hydrobiology, 12/16 Banacha, Lodz, Poland University of Lodz, Faculty of Biology and Environmental Protection, Department of Invertebrate Zoology and Hydrobiology, 12/16 Banacha Lodz Poland; 3 Hofstra University, Department of Biology, Hempstead, NY 11549-1140, United States of America Hofstra University, Department of Biology Hempstead, NY 11549-1140 United States of America; 4 Johann Wolfgang Goethe University Frankfurt, Department of Biological Sciences, Institute of Ecology, Evolution and Diversity, Max-von-Laue-Str. 13, Frankfurt am Main, Germany Johann Wolfgang Goethe University Frankfurt, Department of Biological Sciences, Institute of Ecology, Evolution and Diversity, Max-von-Laue-Str. 13 Frankfurt am Main Germany; 5 Muséum national d’Histoire naturelle, Direction des Collections, CP 51, 55 rue de Buffon, Paris, France Muséum national d’Histoire naturelle, Direction des Collections, CP 51, 55 rue de Buffon Paris France; 6 Universidad Nacional Autónoma de México, Facultad de Ciencias, Unidad Multidisciplinaria de Docencia e Investigación Sisal, Sisal, Mexico Universidad Nacional Autónoma de México, Facultad de Ciencias, Unidad Multidisciplinaria de Docencia e Investigación Sisal Sisal Mexico; 7 Universidad Nacional Autónoma de México, Unidad Académica de Ecología y Biodiversidad Acuática, Instituto de Ciencias del Mar y Limnología, Circuito Exterior S/N, Cd. Universitaria, Mexico City, Mexico Universidad Nacional Autónoma de México, Unidad Académica de Ecología y Biodiversidad Acuática, Instituto de Ciencias del Mar y Limnología, Circuito Exterior S/N, Cd. Universitaria Mexico City Mexico; 8 University of Lodz, Department of Invertebrate Zoology and Hydrobiology, Faculty of Biology and Environmental Protection, 12/16 Banacha, Lodz, Poland University of Lodz, Department of Invertebrate Zoology and Hydrobiology, Faculty of Biology and Environmental Protection, 12/16 Banacha Lodz Poland; 9 Universidade Estadual de Campinas, Institute of Biology, 13083-970, Campinas, São Paulo, Brazil Universidade Estadual de Campinas, Institute of Biology, 13083-970, Campinas São Paulo Brazil; 10 Hong Kong Baptist University, Department of Biology, Kowloon, 999077, Hong Kong, China Hong Kong Baptist University, Department of Biology, Kowloon, 999077 Hong Kong China; 11 University of California San Diego, Scripps Institution of Oceanography, La Jolla, CA, United States of America University of California San Diego, Scripps Institution of Oceanography La Jolla, CA United States of America; 12 Zoological Institute of the Russian Academy of Sciences, Universitetskaya Emb, 1, St Petersburg, Russia Zoological Institute of the Russian Academy of Sciences, Universitetskaya Emb, 1 St Petersburg Russia; 13 Universidade Federal de Pernambuco, Museu de Oceanografia Prof. Petrônio Alves Coelho, Laboratório de Carcinologia, Av. Arquitetura s/n, Cidade Universitária, Recife, Brazil Universidade Federal de Pernambuco, Museu de Oceanografia Prof. Petrônio Alves Coelho, Laboratório de Carcinologia, Av. Arquitetura s/n, Cidade Universitária Recife Brazil; 14 University of Vienna, Department of Palaeontology, Josef-Holaubek-Platz 2, Vienna, Austria University of Vienna, Department of Palaeontology, Josef-Holaubek-Platz 2 Vienna Austria; 15 University of Lodz, Faculty of Biology and Environmental Protection, Department of Invertebrate Zoology and Hydrobiology, 12/16 Banacha, Łódź, Poland University of Lodz, Faculty of Biology and Environmental Protection, Department of Invertebrate Zoology and Hydrobiology, 12/16 Banacha Łódź Poland; 16 Chinese Academy of Sciences, Institute of Oceanology, Qingdao, China Chinese Academy of Sciences, Institute of Oceanology Qingdao China

**Keywords:** Annelida, Mollusca, Crustacea, alpha taxonomy, taxonomy service lab, collaborative taxonomy

## Abstract

**Background:**

Despite centuries of exploration, marine invertebrate biodiversity remains notably under-described. The majority of species in major marine groups are still unnamed, limiting our ability to understand and conserve ecosystems facing rapid environmental change. The rate of species discovery continues to outpace the formal process of species description. This gap creates an urgent need for streamlined, scalable approaches to taxonomy. The SENCKENBERG OCEAN SPECIES ALLIANCE was founded to help meet this challenge by facilitating global collaboration, offering technical support for species documentation and promoting efficient taxonomic publishing. Within this framework, *Ocean Species Discoveries* provides a forum for concise, but data-rich descriptions of marine invertebrate taxa. This second collection presents a diverse set of taxonomic contributions, based on recent and historical collections, including newly-described species and a re-description of a previously poorly-known taxon. The integrative documentation of the taxa treated herein was facilitated by the newly-established Discovery Laboratory at the Senckenberg Research Institute, the first service unit dedicated to supporting alpha taxonomists.

**New information:**

This article presents 14 new species and one re-description, two new genera, with taxa spanning three phyla. Newly-described taxa comprise two polychaete annelids: *Nicon salinus* Hernández-Alcántara & Dávila-Jiménez, **sp. nov.** and *Spinther bohnorum* Tilic & Rouse, **sp. nov.** Molluscs span four classes, with three polyplacophorans: *Craspedochiton zefranki* Vončina, **sp. nov.**, *Ferreiraella charazata* Sigwart, **sp. nov.** and a new genus with type species *Pycnodontochiton sinensis* Sirenko, Zhang & Sigwart, **gen. et sp. nov.** and *Pycnodontochiton tenuidontus* (Saito and Okutani, 1990), **comb. nov.** The new monoplacophoran *Veleropilina gretchenae* Sigwart & Steger, **sp. nov.** is one of the first species of this class with a high-quality genome, published from the specimen that is now the holotype. The scaphopod *Laevidentalium wiesei* Sahlmann, 2012 represents a re-description and range extension and the bivalve *Myonera aleutiana* Machado & Sigwart, **sp. nov.** is the second bivalve including an anatomical description with non-invasive methods using micro-CT. Amongst crustaceans, there are two new amphipod species: *Apotectonia senckenbergae* Momtazi & Riehl, **sp. nov.** and *Metharpinia hirsuta* Souza-Filho & Andrade, **sp. nov.** Three isopod species were described, including the parasitic species *Zeaione everta* Boyko & Williams, **sp. nov.** that is the only species in the new genus *Zeaione* Boyko & Williams, **gen. nov.** and two free-living isopods: *Haploniscus bulbosus* Henseler, Knauber & Riehl, **sp. nov.** and *Macrostylis peteri* Riehl, **sp. nov.** Finally, there are two new tanaidaceans: *Hoplopolemius olo* Jóźwiak & Stępień, **sp. nov.** and *Nesotanais thalassinus* Stępień, **sp. nov.**

The data used for the description of ten of the species and one of the new genera treated herein were wholly or partially obtained at the SOSA Discovery Laboratory using integrative methods including light and electron microscopy, confocal imaging, molecular barcoding and micro-CT scanning. Additional novel findings include the first record of the family Macrostylidae and the genus *Macrostylis* G. O. Sars, 1864 from Australian waters (*Macrostylis peteri*, **sp. nov.**) and novel host associations: *Ferreiraella charazata*, **sp. nov.** is documented with epibiotic tubeworms on its tail valves that are typical of this genus and the decapod *Eucalliaxiopsis aequimana* (Baker, 1907) is newly recorded as a host for bopyrid isopods, representing the first such record for the family Eucalliacidae.

## Introduction

Marine biodiversity remains dramatically under-described. Despite centuries of collecting and studying ocean life, the majority of invertebrate species remain unnamed, hindering efforts to understand and protect ecosystems undergoing increasingly rapid — and potentially irreversible — change. In times of anthropogenically-driven biodiversity loss, the pace of formal species description lags far behind the rate of discovery in the field, creating a critical need for more efficient, scalable approaches to alpha taxonomy (Mora et al. 2011, Sigwart et al. 2023).

The SENCKENBERG OCEAN SPECIES ALLIANCE is an open, growing network of like-minded professionals, skilled amateurs and stakeholders valuing marine biodiversity and supporting its discovery and conservation. We established the *Ocean Species Discoveries* series as a new taxonomy-focused publication platform offering a streamlined, data-rich format tailored to concise species descriptions of marine invertebrates (Senckenberg Ocean Species Alliance (SOSA) et al. 2024). This second issue of the series presents another diverse collection of high-quality taxonomic studies across multiple phyla, demonstrating the variety of approaches scientists are using to accelerate the documentation of species inhabiting our oceans.

The articles include new taxa collected during recent and earlier fieldwork, as well as a re-description of a previously little-known species. Discovering undescribed marine invertebrates often involves representatives of new higher-level taxa, such as the new genera *Pycnodontochiton* Sirenko, Zhang & Sigwart, **gen. nov.** and Zeaione Boyko & Williams, **gen. nov.** described herein, highlighting how little we still know about marine biodiversity, both in deep and shallow waters.

A number of the species described herein were processed partly or entirely using the facilities of the newly-established Discovery Laboratory at the Senckenberg Research Institute and Museum Frankfurt, Germany: Discovery Laboratory of the SENCKENBERG OCEAN SPECIES ALLIANCE. This infrastructure is being developed specifically to support taxonomists describing new species and contributed to nine of the 15 species and one of the two new genera described in this paper.

The concise descriptions assembled in this publication encompass both externally conducted work submitted for publication and projects coordinated and/or supported within the framework of the Ocean Species Alliance. Rather than serving as a showcase of institutional capability, the present article highlights the value of dedicated and openly available technical support in enabling more scientists to contribute high-quality taxonomic research (Sigwart et al. 2023). The type specimens of the species described herein have been deposited in institutions worldwide following the preferences and requirements of the individual taxonomic authors (not necessarily in Senckenberg). Our shared vision is making taxonomy faster, more efficient, more accessible and more visible. The studies in this issue add to the growing body of taxonomic knowledge on marine invertebrates and provide examples for the outcome of efficient workflows for documenting our planet's vast species diversity. This collaborative effort is intended to illustrate the continued need for integrative taxonomic work and highlights the range of tools and collaborations now being applied to accelerate species discovery in the marine realm, inspired by a shared commitment to increasing the accessibility, transparency and speed of taxonomic publication.

This publication is based on a broad suite of methods and approaches, reflecting the diversity of marine taxa covered. The descriptions underscore the enduring importance of morphological characters and morphological diagnoses, in particular, for practical utility in understanding marine biodiversity. Generally, molecular data accompany only 20% of new marine species descriptions (Bouchet et al. 2023), but we reported such information for seven species here, including one species of monoplacophoran that was initially identified as new by examining the complete genome (Chen et al. 2025).

It should be emphasised that, while this work is a collaborative effort, it is also a compilation of many independent projects assembled across the growing SENCKENBERG OCEAN SPECIES ALLIANCE network. The structure of this article closely follows that of the first issue of the *Ocean Species Discoveries* series (Senckenberg Ocean Species Alliance (SOSA) et al. 2024), with individual species treatments arranged in traditional systematic order. Following the first 12 species reported in the initial paper, species herein begin at discovery number 13 and continue to number 27, with much higher numbers of species yet to come in the future.

### Summary of contents

**Classification of the taxa (re-)described in this article**

**Phylum Annelida Lamarck, 1802**

Class Polychaeta Grube, 1850

Subclass Errantia Audouin & H. Milne-Edwards, 1832

Superorder Aciculata Rouse & Fauchald, 1997

Order Phyllodocida Dales, 1962

Family Nereididae Blainville, 1818

Genus *Nicon* Kinberg, 1865, emended

**13.** *Nicon salinus* Hernández-Alcántara & Dávila-Jiménez, **sp. nov.** (contributed by **Pablo Hernández-Alcántara** and **Yasmín Dávila-Jiménez**)

Order Aciculata *incertae sedis*

Family Spintheridae Augener, 1913

Genus *Spinther* Johnston, 1845

**14.** *Spinther bohnorum* Tilic & Rouse, **sp. nov.** (contributed by **Ekin Tilic** and **Greg W. Rouse**)

**Phylum Mollusca Linnaeus, 1758**

Class Polyplacophora Gray, 1821

Subclass Neoloricata Bergenhayn, 1955

Order Chitonida Thiele, 1909

Suborder Acanthochitonina Bergenhayn, 1930

Superfamily Cryptoplacoidea H. Adams & A. Adams, 1858

Family Acanthochitonidae Pilsbry, 1893

Genus *Craspedochiton* Shuttleworth, 1853

**15.** *Craspedochiton zefranki* Vončina, **sp. nov.** (contributed by **Katarzyna Vončina**)

Order Lepidopleurida Thiele, 1909

Family Abyssochitonidae Dell'Angelo & Palazzi, 1989

Genus Ferreiraella Sirenko, 1988

**16.** *Ferreiraella charazata* Sigwart, **sp. nov.** (contributed by **Julia D. Sigwart** and **Jian-Wen Qiu**)

Family Leptochitonidae Dall, 1889

Genus *Pycnodontochiton* Sirenko, Zhang & Sigwart, **gen. nov.**

**17.** *Pycnodontochiton sinensis* Sirenko, Zhang & Sigwart, **sp. nov.** (contributed by **Boris Sirenko**, **Junlong Zhang**, **Jian-Wen Qiu** and **Julia D. Sigwart**)

Class Monoplacophora Odhner, 1940

Order Neopilinida Lauterbach, 1983

Family Neopilinidae Knight & Yochelson, 1958

Genus *Veleropilina* Starobogatov & Moskalev, 1987

**18.** *Veleropilina gretchenae* Sigwart & Steger, **sp. nov.** (contributed by **Julia D. Sigwart** and **Jan Steger**)

Class Scaphopoda Bronn, 1862

Order Dentaliida Starobogatov, 1974

Family Laevidentaliidae C.P. Palmer, 1974

Genus *Laevidentalium* Cossmann, 1888

**19.** *Laevidentalium wiesei* Sahlmann, 2012, **re-description** (contributed by **Jan Steger** and **Julia D. Sigwart**)

Class Bivalvia Linnaeus, 1758

Superorder Anomalodesmata Dall, 1889

Order Poromyida Ridewood, 1903

Superfamily Cuspidarioidea Dall, 1886

Family Cuspidariidae Dall, 1886

Genus *Myonera* Dall & E. A. Smith, 1886

**20.** *Myonera aleutiana* Machado & Sigwart, **sp. nov.** (contributed by **Fabrizio Marcondes Machado** and **Julia D. Sigwart**)

**Phylum Arthropoda Gravenhorst, 1843**

Subphylum Crustacea Brunnich, 1772

Class Malacostraca Latreille, 1802

Superorder Peracarida Calman, 1904

Order Amphipoda Latreille, 1816

Family Alicellidae Lowry & De Broyer, 2008

Genus *Apotectonia* Barnard & Ingram, 1990

**21.** *Apotectonia senckenbergae* Momtazi & Riehl, **sp. nov.** (contributed by **Farzaneh Momtazi** and **Torben Riehl**)

Family Phoxocephalidae G.O. Sars, 1891

Genus *Metharpinia* Schellenberg, 1931

**22.** *Metharpinia hirsuta* Souza-Filho & Andrade, **sp. nov.** (contributed by **Jesser F. Souza-Filho** and **Luiz F. Andrade**)

Order Isopoda Latreille, 1816

Family Bopyridae Rafinesque, 1815

Subfamily Pseudioninae R. Codreanu, 1967

Genus *Zeaione* Boyko & Williams, **gen. nov.**

**23.** *Zeaione everta* Boyko & Williams, **sp. nov.** (contributed by **Christopher B. Boyko** and **Jason D. Williams**)

Family Haploniscidae Hansen, 1916

Genus *Haploniscus* Richardson, 1908

**24.** *Haploniscus bulbosus* Henseler, Knauber & Riehl, **sp. nov.** (contributed by **Mats Henseler**, **Henry Knauber** and **Torben Riehl**)

Family Macrostylidae Hansen, 1916

Genus *Macrostylis* G.O. Sars, 1864

**25.** *Macrostylis peteri* Riehl, **sp. nov.** (contributed by **Torben Riehl**)

Order Tanaidacea Dana, 1849

Suborder Apseudomorpha Sieg, 1980

Family Metapseudidae Lang, 1970

Genus *Hoplopolemius* Sganga & Roccatagliata, 2016

**26.** *Hoplopolemius olo* Jóźwiak & Stępień, **sp. nov.** (contributed by **Piotr Jóźwiak** and **Anna Stępień**)

Suborder Tanaidomorpha Sieg, 1980

Family Nototanaidae Sieg, 1976

Genus *Nesotanais* Shiino, 1968

**27.** *Nesotanais thalassinus* Stępień, **sp. nov.** (contributed by **Anna Stępień**)

**New geographical distributions**

*Laevidentalium wiesei* Sahlmann, 2012 is recorded from the Aleutian Trench, Northeast Pacific Ocean (cf. Sigwart et al. (2025)). *Macrostylis peteri* Riehl, **sp. nov**. is the first record of this genus and the family Macrostylidae from Australian waters.

**New host records**

The axiidean shrimp *Eucalliaxiopsis aequimana* (Baker, 1907) (Crustacea, Decapoda, Eucalliacidae) is reported for the first time as a host of any bopyrid species. No other species in Eucalliacidae Manning & Felder, 1991 has previously been reported harbouring bopyrids, although other axiidean families are known to host species of Bopyridae and Ionidae H. Milne Edwards, 1840 (Boyko et al. 2017, Hernáez et al. 2023).

## Materials and methods

**General methods**

This second issue of *Ocean Species Discoveries* marks a key advancement in the mission of the SENCKENBERG OCEAN SPECIES ALLIANCE project to bridge gaps in marine invertebrate taxonomy. Central to this progress has been the new Discovery Laboratory at SENCKENBERG, Frankfurt, which supports global taxonomic efforts by offering access to advanced infrastructure and tailored technical services. Showcasing this capability, the issue presents (re)descriptions of several species — including polychaetes (*Spinther bohnorum*, **sp. nov.**), molluscs in four taxonomic classes (*Craspedochiton zefranki*, **sp. nov.**, *Ferreiraella charazata*, **sp. nov.**, *Pycnodontochiton sinensis*, **gen. et sp. nov.**, *Veleropilina gretchenae*, **sp. nov.**, *Laevidentalium wiesei* Sahlmann, 2012, *Myonera aleutiana*, **sp. nov.**) and crustaceans (*Apotectonia senckenbergae*, **sp. nov.**, *Haploniscus bulbosus*, **sp. nov.**, *Macrostylis peteri*, **sp. nov.**) — for which data were wholly or partially acquired in the Discovery Laboratory and associated facilities, either independently by the contributors or by staff funded by the SENCKENBERG OCEAN SPECIES ALLIANCE project. The lab was designed to address the practical challenges posed by the vast taxonomic diversity of marine invertebrates and a growing suite of methods available for their study.

The equipment and methods used at this facility to process specimens are detailed within each individual species description. In general, these descriptions included macrophotography, optical and scanning electron microscopy (HITACHI TM4000 tabletop SEM), confocal laser scanning (LEICA DM2500 microscope), dissection, barcoding, scientific illustration and non-destructive micro-CT scanning for visualising internal anatomy (WERTH TomoScope® XS Plus scanner). Beyond infrastructure, the Discovery Laboratory offers flexible and scalable hands-on support that is constantly being further developed through feedback from the taxonomic community, aiming at empowering taxonomists by providing high-quality data for integrative species descriptions. Where methods represent widely-accepted standard procedures, their description may be brief, but references are provided to original publications with more detailed explanations in order to appropriately credit previous taxonomic and methodological work.

Specimen metadata are provided in the "materials" section at the start of each taxon treatment; for ease of readability, each species description also includes a short summary of the type material, material examined and type locality as subsections of the description. While the species descriptions all follow a standard format, there are many elements that differ amongst specialist communities; as a group of contributors, we recognise the importance of allowing each description a certain latitude in formatting and terminology, to enable a collective effort on divergent taxa and traditions.

### Further description of methods

Taxon-specific methods are given alongside the species descriptions.

### Notes on crustacean descriptions

In the description of crustaceans, all appendages’ article-length ratios are given in proximal to distal order, excluding setae. Many ratios are used for descriptions in this paper. To avoid multiple repetition of the word ‘times,’ these are reported as a multiplier of an object of a telegraphic phrase to indicate the size of the subject of the phrase. For example, ‘Uropod length 2.2 width’ means ‘the length of the uropod is 2.2 times its width’. This example is mathematically equivalent to the equation ‘L = 2.2W’. Dependent object clauses, separated off by a comma, do not repeat the subject.

Amongst all descriptions of crustacean taxa herein, abbreviations of important morphological terms were standardised, extending the approach suggested by: A1 – Antenna 1/antennula; A2 – Antenna 2; Acc flag – Accessory flagellum; Art – Article (of antennae and legs); C – Coxa; Ceph Cephalothorax; Ch – Cheliped (in Tanaidacea only); Ep – Epimeral plate/epimeron; Lbi – Labium; Lbr – Labrum; Md – Mandible; Mo – Mouthparts; Mx1 – Maxilla 1/maxillula; Mx2 – Maxilla 2; Mxp – Maxilliped; Op – Operculum; P – Pereopod; Pl – Pleomere/pleonite; Plp – Pleopod; Plt – Pleotelson; Prn – Pereomere/pereonite; T – Telson; U – Uropod.

The terms "subequal" and "subsimilar" are used herein as follows. Subequal means "almost equal" in size or quantity, as in two animals measuring 10.2 cm and 10.5 cm are *subequal*. Subsimilar is used to mean "partially similar" in shape or structure, as in two shells with different contours, but a vaguely similar outline are *subsimilar*.

## Taxon treatments

### Nicon salinus

Hernández-Acántara & Dávila-Jiménez sp. nov.

50D8DB81-A9E9-58DD-8472-7E966F85C124

A8C8FCBC-0245-4D49-8341-D32DD4EE4365

#### Materials

- **Type status:** Holotype. **Occurrence:** catalogNumber: CNAP-POH-39-003; recordedBy: Yasmín Dávila-Jiménez; individualCount: 1; lifeStage: adult; occurrenceStatus: present; preparations: 70% EtOH; disposition: in collection; occurrenceID: 1EB9D302-9286-5C39-8966-5C9A5EEC3762; **Taxon:** scientificName: *Nicon salinus* Hernández-Alcántara & Dávila-Jiménez; kingdom: Animalia; phylum: Annelida; class: Polychaeta; order: Phyllodocida; family: Nereididae; genus: Nicon; specificEpithet: *salinus*; taxonRank: species; scientificNameAuthorship: Hernández-Alcántara & Dávila-Jiménez; nomenclaturalCode: ICZN; **Location:** continent: North America; waterBody: Southern Gulf of Mexico; country: Mexico; countryCode: MX; stateProvince: Yucatán; locality: Río Lagartos, hypersaline coastal lagoon; verbatimDepth: 1.03 m; locationRemarks: hypersaline coastal lagoon; verbatimLatitude: 21°35.74'N; verbatimLongitude: 88°04.28'W; verbatimCoordinateSystem: degrees decimal minutes; **Event:** samplingProtocol: Ponar standard dredge 0.052 m^2^; eventDate: 19-05-2005; habitat: 14.3% silt, 69.9% sand, 15.8% gravel, 31.5°C, 34.3 psu, 7.21 mg/l, pH 8.16; fieldNumber: 15-RL1805; **Record Level:** institutionCode: ICML-UNAM; collectionCode: CNAP; basisOfRecord: PreservedSpecimen
- **Type status:** Paratype. **Occurrence:** catalogNumber: CNAP-POP-39-005; recordedBy: Yasmín Dávila-Jiménez; individualCount: 8; lifeStage: adult; occurrenceStatus: present; preparations: 70% EtOH; disposition: in collection; occurrenceID: 3C2085B4-BD95-59C5-BF36-EB11CC6B9FC9; **Taxon:** scientificName: *Nicon salinus* Hernández-Alcántara & Dávila-Jiménez; kingdom: Animalia; phylum: Annelida; class: Polychaeta; order: Phyllodocida; family: Nereididae; genus: Nicon; specificEpithet: *salinus*; taxonRank: species; scientificNameAuthorship: Hernández-Alcántara & Dávila-Jiménez; nomenclaturalCode: ICZN; **Location:** continent: North America; waterBody: Southern Gulf of Mexico; country: Mexico; countryCode: MX; stateProvince: Yucatán; locality: Río Lagartos, hypersaline coastal lagoon; verbatimDepth: 1.09 m; locationRemarks: hypersaline coastal lagoon; verbatimLatitude: 21°36.46'N; verbatimLongitude: 88°08.12'W; verbatimCoordinateSystem: degrees decimal minutes; **Event:** samplingProtocol: Ponar standard dredge 0.052 m^2^; eventDate: 19-05-2018; habitat: 6.9% silt, 66.8% sand, 26.28% gravel, 30.5°C, 35.19 psu, 9.87 mg/l, pH 8.39; fieldNumber: 12-RL1805; **Record Level:** institutionCode: ICML-UNAM; collectionCode: CNAP; basisOfRecord: PreservedSpecimen
- **Type status:** Paratype. **Occurrence:** catalogNumber: CNAP-POP-39-006; recordedBy: Yasmín Dávila-Jiménez; individualCount: 2; lifeStage: adult; occurrenceStatus: present; preparations: coated with gold; disposition: in collection; occurrenceID: 8CCD5200-0822-5099-B539-B648643070C8; **Taxon:** scientificName: *Nicon salinus* Hernández-Alcántara & Dávila-Jiménez; kingdom: Animalia; phylum: Annelida; class: Polychaeta; order: Phyllodocida; family: Nereididae; genus: Nicon; specificEpithet: *salinus*; taxonRank: species; scientificNameAuthorship: Hernández-Alcántara & Dávila-Jiménez; nomenclaturalCode: ICZN; **Location:** continent: North America; waterBody: Southern Gulf of Mexico; country: Mexico; countryCode: MX; stateProvince: Yucatán; locality: Río Lagartos, hypersaline coastal lagoon; verbatimDepth: 1.09 m; locationRemarks: hypersaline coastal lagoon; verbatimLatitude: 21°36.46'N; verbatimLongitude: 88°08.12'W; verbatimCoordinateSystem: degrees decimal minutes; **Event:** samplingProtocol: Ponar standard dredge 0.052 m^2^; eventDate: 19-05-2018; habitat: 6.9% silt, 66.8% sand, 26.28% gravel, 30.5°C, 35.19 psu, 9.87 mg/l, pH 8.39; fieldNumber: 12-RL1805; **Record Level:** institutionCode: ICML-UNAM; collectionCode: CNAP; basisOfRecord: specimens coated with gold for SEM studies
- **Type status:** Paratype. **Occurrence:** catalogNumber: CNAP-POP-39-007; recordedBy: Yasmín Dávila-Jiménez; individualCount: 5; lifeStage: adult; occurrenceStatus: present; preparations: 70% EtOH; disposition: in collection; occurrenceID: E0E83DBD-1A2D-57CD-81AF-FA6092D56DB9; **Taxon:** scientificName: *Nicon salinus* Hernández-Alcántara & Dávila-Jiménez; kingdom: Animalia; phylum: Annelida; class: Polychaeta; order: Phyllodocida; family: Nereididae; genus: Nicon; specificEpithet: *salinus*; taxonRank: species; scientificNameAuthorship: Hernández-Alcántara & Dávila-Jiménez; nomenclaturalCode: ICZN; **Location:** continent: North America; waterBody: Southern Gulf of Mexico; country: Mexico; countryCode: MX; stateProvince: Yucatán; locality: Río Lagartos, hypersaline coastal lagoon; verbatimDepth: 1.03 m; locationRemarks: hypersaline coastal lagoon; verbatimLatitude: 21°35.74'N; verbatimLongitude: 88°04.28'W; verbatimCoordinateSystem: degrees decimal minutes; **Event:** samplingProtocol: Ponar standard dredge 0.052 m^2^; eventDate: 19-05-2005; habitat: 14.3% silt, 69.9% sand, 15.8% gravel, 31.5°C, 34.3 psu, 7.21 mg/l, pH 8.16; fieldNumber: 15-RL1805; **Record Level:** institutionCode: ICML-UNAM; collectionCode: CNAP; basisOfRecord: PreservedSpecimen
- **Type status:** Other material. **Occurrence:** catalogNumber: CNAP-PO-39-034/2025-GMX-LE; recordedBy: Yasmín Dávila-Jiménez; individualCount: 7; lifeStage: adult; occurrenceStatus: present; preparations: 70% EtOH; disposition: in collection; occurrenceID: 64C2C811-6FA9-5B81-B86A-2EC2325FA5A0; **Taxon:** scientificName: *Nicon salinus* Hernández-Alcántara & Dávila-Jiménez; kingdom: Animalia; phylum: Annelida; class: Polychaeta; order: Phyllodocida; family: Nereididae; genus: Nicon; specificEpithet: *salinus*; taxonRank: species; scientificNameAuthorship: Hernández-Alcántara & Dávila-Jiménez; nomenclaturalCode: ICZN; **Location:** continent: North America; waterBody: Southern Gulf of Mexico; country: Mexico; countryCode: MX; stateProvince: Yucatán; locality: Río Lagartos, hypersaline coastal lagoon; verbatimDepth: 1.09 m; locationRemarks: hypersaline coastal lagoon; verbatimLatitude: 21°36.46'N; verbatimLongitude: 88°08.12'W; verbatimCoordinateSystem: degrees decimal minutes; **Event:** samplingProtocol: Ponar standard dredge 0.052 m^2^; eventDate: 19-05-2018; habitat: 6.9% silt, 66.8% sand, 26.28% gravel, 30.5°C, 35.19 psu, 9.87 mg/l, pH 8.39; fieldNumber: 12-RL1805; **Record Level:** institutionCode: ICML-UNAM; collectionCode: CNAP; basisOfRecord: PreservedSpecimen
- **Type status:** Other material. **Occurrence:** catalogNumber: CNAP-PO-39-034/2026-GMX-LE; recordedBy: Yasmín Dávila-Jiménez; individualCount: 8; lifeStage: adult; occurrenceStatus: present; preparations: 70% EtOH; disposition: in collection; occurrenceID: 45D22B06-60DE-5337-8BCE-BC242D073016; **Taxon:** scientificName: *Nicon salinus* Hernández-Alcántara & Dávila-Jiménez; kingdom: Animalia; phylum: Annelida; class: Polychaeta; order: Phyllodocida; family: Nereididae; genus: Nicon; specificEpithet: *salinus*; taxonRank: species; scientificNameAuthorship: Hernández-Alcántara & Dávila-Jiménez; nomenclaturalCode: ICZN; **Location:** continent: North America; waterBody: Southern Gulf of Mexico; country: Mexico; countryCode: MX; stateProvince: Yucatán; locality: Río Lagartos, hypersaline coastal lagoon; verbatimDepth: 0.61 m; locationRemarks: hypersaline coastal lagoon; verbatimLatitude: 21°35.73'N; verbatimLongitude: 88°04.28'W; verbatimCoordinateSystem: degrees decimal minutes; **Event:** samplingProtocol: Ponar standard dredge 0.052 m^2^; eventDate: 28-02-2018; habitat: 49.3% silt, 50.1% sand, 0.6% gravel, 26.7°C, 54.46 psu, 8.09 mg/l, pH 8.24; fieldNumber: 15-RL-1802; **Record Level:** institutionCode: ICML-UNAM; collectionCode: CNAP; basisOfRecord: PreservedSpecimen
- **Type status:** Other material. **Occurrence:** catalogNumber: CNAP-PO-39-034/2027-GMX-LE; recordedBy: Yasmín Dávila-Jiménez; individualCount: 5; lifeStage: adult; occurrenceStatus: present; preparations: 70% EtOH; disposition: in collection; occurrenceID: 50C8E210-996E-54B1-86F7-56172AC00AA5; **Taxon:** scientificName: *Nicon salinus* Hernández-Alcántara & Dávila-Jiménez; kingdom: Animalia; phylum: Annelida; class: Polychaeta; order: Phyllodocida; family: Nereididae; genus: Nicon; specificEpithet: *salinus*; taxonRank: species; scientificNameAuthorship: Hernández-Alcántara & Dávila-Jiménez; nomenclaturalCode: ICZN; **Location:** continent: North America; waterBody: Southern Gulf of Mexico; country: Mexico; countryCode: MX; stateProvince: Yucatán; locality: Río Lagartos, hypersaline coastal lagoon; verbatimDepth: 1.03 m; locationRemarks: hypersaline coastal lagoon; verbatimLatitude: 21°35.74'N; verbatimLongitude: 88°04.28'W; verbatimCoordinateSystem: degrees decimal minutes; **Event:** samplingProtocol: Ponar standard dredge 0.052 m^2^; eventDate: 19-05-2005; habitat: 14.3% silt, 69.9% sand, 15.8% gravel, 31.5°C, 34.3 psu, 7.21 mg/l, pH 8.16; fieldNumber: 15-RL1805; **Record Level:** institutionCode: ICML-UNAM; collectionCode: CNAP; basisOfRecord: PreservedSpecimen

#### Description

**Figure 1.**
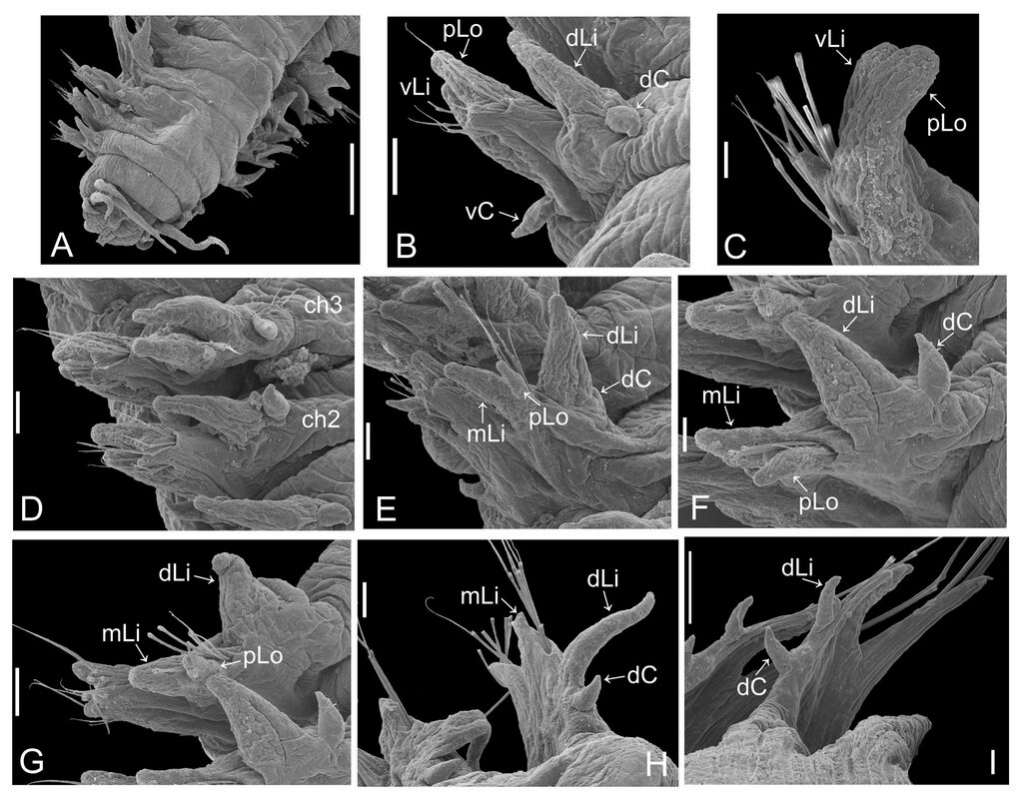
*Nicon salinus* Hernández-Acántara & Dávila-Jiménez, **sp. nov.** Paratype (CNAP-ICML: POP-39-005). **A** Anterior region, ventral view; **B** Chaetiger 1; **C** Notopodium of chaetiger 1; **D** Chaetigers 2, 3; **E** Chaetiger 4; **F** Notopodium of chaetiger 8; **G** Chaetiger 9; **H** Chaetiger 16; **I** Posterior chaetiger. Abbreviations: ch3, ch2 = chaetigers 2 and 3; dC = dorsal cirrus; dLi = dorsal ligule; mLi = median ligule; pLo = postchaetal lobule; vC = ventral cirrus; vLi = ventral ligule. Scale bars: 200 μm (A), 50 μm (B, D–I), 20 μm (C).

**Figure 2.**
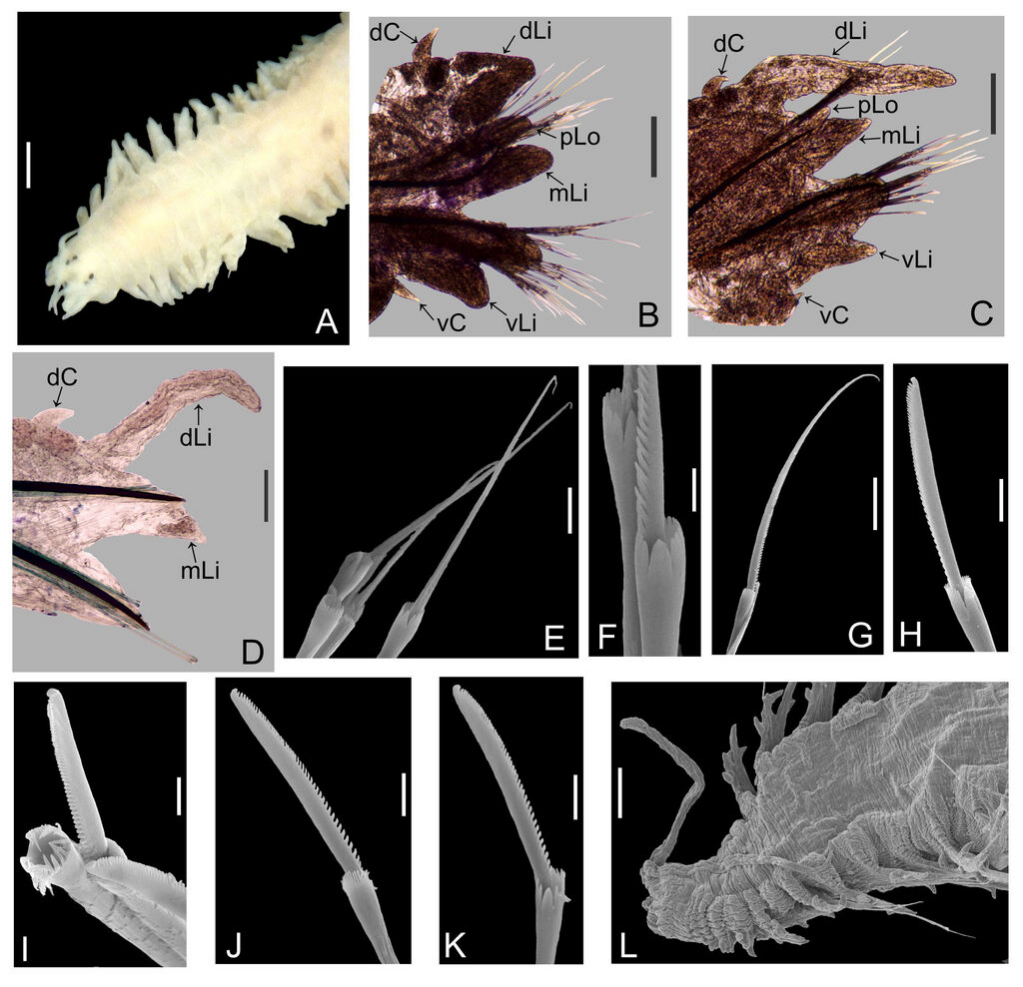
*Nicon salinus* Hernández-Acántara & Dávila-Jiménez, **sp. nov.** Holotype (CNAP-POH-39-003). **A** Anterior region, dorsal view. Additional material (CNAP-PO-39-034/2027); **B** Chaetiger 10; **C** Chaetiger 30; **D** Chaetiger 50. Paratype (CNAP-ICML: POP-39-005); **E** Homogomph spiniger of chaetiger 1; **F** Homogomph articulation of spiniger of chaetiger 2; **G** Homogomph spiniger of chaetiger 10; **H-K** Homogomph falcigers of chaetigers 12, 15, 25, 30; **L** Pygidium, ventral view. Abbreviations: dC = dorsal cirrus; dLi = dorsal ligule; mLi = median ligule; pLo = postchaetal lobule; vC = ventral cirrus; vLi = ventral ligule. Scale bars: 200 μm (A), 50 μm (B–D), 10 μm (E, H, J, K), 5 μm (F, I), 20 μm (G), 100 μm (L).

Holotype complete, with 84 segments, 30 mm long, 2 mm wide at chaetiger 15 (without chaetae). Paratypes incomplete, with 20–56 segments, 4–25 mm long, 0.8–1.0 mm wide. Colour in ethanol light brown. Prostomium slightly wider than long with a frontal cleft; two small frontal digitate antennae (Fig. 1A). Two pairs of eyes in trapezoidal arrangement, similar in size (Fig. 2A). Palps biarticulate, palpophore well developed, barrel-shaped, with subspherical palpostyles (Fig. 1A, Fig. 2A). Peristomium slightly longer than chaetiger 1, with four pairs of tentacular cirri, short, the longest postero-dorsally reaching to chaetiger 2 (Fig. 1A). Oral and maxillary pharyngeal rings without paragnaths or papillae; jaws brown, with 5 teeth on the dentate cutting edge. First two chaetigers sub-birramous; notopodia without aciculae, represented by a subulate dorsal ligule and short dorsal cirrus (Fig. 1B); neuropodia with subulate postchaetal lobe and ventral ligules and short ventral cirrus (Fig. 1C), only bearing homogomph spinigers (Fig. 2E-G). Birramous chaetigers from chaetiger 3 (Fig. 1D-I, Fig. 2B-D).

Anterior notopodia with short cirriform dorsal cirrus inserted at base of the triangular dorsal ligule; subulate median ligule; cirrus-like prechaetal lobe slightly shorter than the subulate median ligule (Fig. 1D–F, Fig. 2B); cirriform prechaetal lobe gradually smaller in middle chaetigers (Fig. 1D–H, Fig. 2C, D). Median and posterior notopodia with long, cirrus-like, dorsal ligule and short dorsal cirrus, inserted at base of ligule (Fig. 1H, I, Fig. 2C, D); median and posterior chaetigers with short prechaetal lobe, as a small process (Fig. 1H, Fig. 2C, D); near to pygidium, the dorsal ligule is shorter (Fig. 1I).

Anterior neuropodia with a short digitiform postchaetal lobe, projecting beyond end of the acicular ligule (Fig. 1G, Fig. 2B), a triangular ventral ligule and a short ventral cirrus (Fig. 2B). Median and posterior neuropodia with a short postchaetal lobe (Fig. 1C); subulate neuropodial ventral ligule, gradually smaller until entirely disappearing in posterior chaetigers; short ventral cirrus (Fig. 2D).

All notochaetae homogomph spinigers, with long, thin blades. Anterior neuropodia with homogomph spinigers, those in the supra- acicular ramus with long-blades and those from the infra-acicular ramus with short-blades (Fig. 2B, E–G). Median and posterior neuropodia with a supra-acicular ramus having long-bladed homogomph spinigers; infracicular ramus with homogomph spinigers superiorly and homogomph falcigers inferiorly, ending in a blunt curved tooth (Fig. 2H–K). Pygidium with broad margin and two long cirriform cirri, as long as last 7–8 segments (Fig. 1B).

##### Type material

Holotype (CNAP-POH-39-003) and fifteen paratypes (CNAP-POP-39-005, CNAP-POP-39-005, CNAP-POP-39-006). Only preserved specimens were available for the original description.

##### Material examined

Holotype (CNAP-POH-39-003) and paratypes CNAP-POP-39-005 (5 specimens, Sta. 15-RL1805, 21°35.74'N, 88°4.28'W), CNAP-POP-39-005 (8 specimens, Sta. 12-RL1805, 21°36.46'N, 88°08.12'W), CNAP-ICML: POP-39-006 (Sta. 12-RL1805, 2 specimens coated with gold for SEM studies). Other additional specimens were examined: CNAP-PO-39-034/2025-GMX-LE (7 specimens, Sta. 12-RL1805; 21°36.46'N, 88°8.12'W); CNAP-PO-39-034/2026-GMX-LE (8 specimens, Sta. 15-RL1802; 21°35.73'N, 88°04.28'W; CNAP-PO-39-034/2027-GMX-LE (5 specimens, Sta. 15-RL1805; 21°35.74'N, 88°04.28'W).

##### Type locality

Southern Gulf of Mexico, Río Lagartos, Sta. 15-RL1805, 21°35.74'N, 88°4.28'W, collected at 1.03 m depth, in fine sand bottoms.

#### Diagnosis

Pharynx with two dentate jaws, without papillae or paragnaths. Anterior notopodia with triangular dorsal ligule and short cirriform dorsal cirri; subulate prechaetal lobe, similar in length to median ligule; median and posterior notopodia with dorsal ligule long, cirrus-like. Neuropodia with an acicular lobe and subulate neuroacicular ligule; triangular ventral ligule decreasing in size towards posterior chaetigers until entirely disappearing; short ventral cirrus. All notochaetae homogomph spinigers. Neuropodia with long and short-bladed homogomph spinigers and homogomph falcigers with blades ending in a blunt curved tooth.

#### Etymology

The species name is derived from the Latin word “*salinus*”, meaning “salty” or “salted”, adjective used to highlight the hypersaline environment that this new nereidid inhabits, tolerating salinities up to 54.46 psu. Gender masculine.

#### Distribution

Only known from type locality

#### Ecology

Habitat: At 0.29–1.09 m depth, in fine sand and mud-sandy bottoms (50.08–78.67% sand). Temperature: 26.5–31.5°C; salinity: 34.30–54.46 psu; dissolved oxygen: 6.13–9.87 mg/l; pH: 7.97–8.39.

#### Taxon discussion

The genus *Nicon* has eleven valid species, including *N. salinus* **sp. nov.** (Read, G.; Fauchald, K. (Ed.) 2024), which can be separated into two large groups, according to presence or absence of notopodial prechaetal lobes (de León-González and Trovant 2013). The species group lacking notopodial prechaetal lobes includes *N. ablepsia* Wang et al., 2021 (Wang et al. 2021), *N. abyssalis* Hartman, 1967, *N. maculatus* Kinberg, 1865 (Kinberg 1865), *N. moniloceras* (Hartman, 1940) (Hartman 1940), *N. pettibonae* de León-González & Salazar-Vallejo, 2003 (de León-González and Salazar-Vallejo 2003) and *N. yaquinae* Fauchald, 1977 (Fauchald 1977) (Table 1).

On the other hand, the species group bearing notopodial prechaetal lobes includes *N. salinus* **sp. nov.** from the Gulf of Mexico, *N. aestuarensis* Knox, 1951 (Knox 1951) from New Zealand, *N. japonicus* Imajima, 1972 (Imajima 1972) from Ariake Sea, Japan, *N. orensanzi* de León-González & Trovant, 2013 from Bunche Beach, Ecuador and *N. rotundus* Hutchings & Reid, 1990 from Port Darwin, Australia. *Nicon salinus* **sp. nov.**, with the anterior notopodial prechaetal lobes long, only slightly shorter than the median ligule and the neuropodia exclusively bearing homogomph spinigers and homogomph falcigers, can be clearly separated from *N. aestuarensis*, *N. japonicus* and *N. rotundus*, which have heterogomph falcigers and short notopodial dorsal ligules. In addition to heterogomph falcigers, *N. rotundus* also bears homogomph falcigers, whereas *N. aestuarensis* and *N. japonicus* bear heterogomph spinigers (Table 1).

In this group, *Nicon orensanzi* and *N. salinus* **sp. nov.** are the only species having long notopodial dorsal ligules, cirrus-like, on median and posterior parapodia. In *N. orensanzi*, the anterior notopodia have small triangular prechaetal lobes, which are absent in median and posterior notopodia and the neuropodia bear homogomph and heterogomph spinigers and sesquigomph falcigers. In contrast, the anterior notopodia in *N. salinus* **sp. nov.** have subulate prechaetal lobes, similar in length to the median ligules, absent in posterior chaetigers and the neuropodia only bear homogomph spinigers and homogomph falcigers (Table 1).

Previously, *N. rotundus* and *N. abyssalis* had been the only species of this genus having neuropodial homogomph falcigers, which were also observed in *N. salinus* **sp. nov.**, but those species lack notopodial prechaetal lobes and, therefore, belong to the other *Nicon* species group (Table 1).

#### Methods

**Sampling**: The biological material was collected in February and May 2018 along the hypersaline Río Lagartos system, as part of the SALINITY GRADIENT ENERGY project of the Centro Mexicano de Innovación en Energía Océano (CEMIE Océano). It is an estuarine system located in the southern Gulf of Mexico (21°26' – 21°38'N; 87°30' – 88°05'W), belonging to the Ría Lagartos Biosphere Reserve. The samples were taken with a Ponar standard dredge (0.052 m^2^) and the sediments were sieved through a 500 μm mesh size to separate the macrofauna.

**Preservation**: The individuals were initially anaesthetised with magnesium chloride, fixed in 4% formaldehyde and later preserved in 70% ethanol (Dávila-Jiménez et al. 2019).

**Morphological methods**: The specimens of the new species were examined in detail to compare their morphological characteristics with those observed in close species: anterior, middle and posterior parapodia were dissected and mounted on glass slides to examine their morphological features and chaetal types. The scanning electron photographs were taken with a JEOL JSM6360LV microscope, the individuals were dehydrated via a graded ethanol series, critical-point dried with liquid CO_2_ and coated with gold.

**Terminology**: To date, different terms have been given to name the nereidid parapodial structures, so, to standardise them we used the terminology for the atokous parapodial suggested by Villalobos-Guerrero and Bakken (2018) with some modifications made by Villalobos-Guerrero et al. (2021). However, due to the neuropodial lobes in the species of *Nicon* not being separable into superior and inferior lobes, the neuropodial lobe was named as the acicular lobe in accordance with Pettibone (1971) and Hutchings and Reid (1990), amongst others. The definition of types of articulation to the compound chaetae (homogomph, sesquigomph and heterogomph) suggested by Villalobos-Guerrero and Bakken (2018) was used to classify the observed homogomph falcigers in the new species.

**Repository**: The holotype and paratypes of the new species and the additional material examined were deposited in the "Colección Nacional de Anélidos Poliquetos" of the ICML, UNAM (CNAP-ICML: DFE.IN.061.0598).

### Spinther bohnorum

Tilic & Rouse sp. nov.

618E959B-79ED-52E3-BC76-EACE9BB877B4

BFED5255-3A68-40F4-A9A2-50B3CFADFF95

https://www.ncbi.nlm.nih.gov/sra/SRR2005641

https://www.ncbi.nlm.nih.gov/nuccore/PV053298

https://www.ncbi.nlm.nih.gov/nuccore/PV054334

Spinther n. sp.: Andrade et al. (2015): fig. 1E, tables 1, 2.
Spinther sp.: Tilic et al. (2022): fig. S2.

#### Materials

- **Type status:** Holotype. **Occurrence:** catalogNumber: A2846; recordedBy: K. White; individualCount: 1; sex: female; lifeStage: adult; occurrenceStatus: present; preparations: fixative: formalin 8%; preservative: ethanol 50%; disposition: in collection; previousIdentifications: *Spinther* sp.; occurrenceID: CE88DCFA-8FEF-5D1B-9FF9-3D51F42F3DCC; **Taxon:** scientificName: *Spinther bohnorum* Tilic & Rouse; kingdom: Animalia; phylum: Annelida; class: Polychaeta; family: Spintheridae; genus: Spinther; specificEpithet: *bohnorum*; taxonRank: species; scientificNameAuthorship: Tilic & Rouse; nomenclaturalCode: ICZN; **Location:** higherGeography: Pacific Ocean; continent: Oceania; waterBody: South Pacific Ocean; islandGroup: Society Islands; island: Mo'orea; country: French Polynesia; countryCode: FP; locality: between Opunohu Bay and Cook's Bay; verbatimDepth: 10-15 m; minimumDepthInMeters: 10; maximumDepthInMeters: 15; verbatimCoordinates: 17.4768°S, 149.8316°W; verbatimLatitude: 17.4768°S; verbatimLongitude: 149.8316°W; decimalLatitude: -17.4768; decimalLongitude: -149.8316; **Identification:** identifiedBy: Greg Rouse; **Event:** eventDate: 14-11-2010; year: 2010; month: 11; day: 14; verbatimEventDate: 11/14/2010; **Record Level:** institutionID: http://biocol.org/urn:lsid:biocol.org:col:14844; collectionID: http://grbio.org/cool/t8vy-919z; institutionCode: SIO; collectionCode: BIC; basisOfRecord: PreservedSpecimen
- **Type status:** Paratype. **Occurrence:** catalogNumber: SMF 32994; recordedBy: K. White; individualCount: 1; lifeStage: juvenile?; occurrenceStatus: present; preparations: fixative: formalin 8%; preservative: ethanol 50%; disposition: in collection; otherCatalogNumbers: SIO:BIC:A18597; previousIdentifications: *Spinther* sp.; occurrenceID: ADD7BF30-2295-5EAA-A693-60F159B03765; **Taxon:** scientificName: *Spinther bohnorum* Tilic & Rouse; kingdom: Animalia; phylum: Annelida; class: Polychaeta; family: Spintheridae; genus: Spinther; specificEpithet: *bohnorum*; taxonRank: species; scientificNameAuthorship: Tilic & Rouse; nomenclaturalCode: ICZN; **Location:** higherGeography: Pacific Ocean; continent: Oceania; waterBody: South Pacific Ocean; islandGroup: Society Islands; island: Mo'orea; country: French Polynesia; countryCode: FP; locality: between Opunohu Bay and Cook's Bay; verbatimDepth: 10-15 m; minimumDepthInMeters: 10; maximumDepthInMeters: 15; verbatimCoordinates: 17.4768°S, 149.8316°W; verbatimLatitude: 17.4768°S; verbatimLongitude: 149.8316°W; decimalLatitude: -17.4768; decimalLongitude: -149.8316; **Identification:** identifiedBy: Greg Rouse; **Event:** eventDate: 14-11-2010; year: 2010; month: 11; day: 14; verbatimEventDate: 11/14/2010; **Record Level:** institutionID: http://biocol.org/urn:lsid:biocol.org:col:34838; institutionCode: SMF; basisOfRecord: PreservedSpecimen
- **Type status:** Other material. **Occurrence:** catalogNumber: —; recordedBy: K. White; individualCount: 1; lifeStage: adult; occurrenceStatus: present; preparations: fixative: RNAlater; disposition: used up; previousIdentifications: *Spinther* sp.; associatedSequences: https://www.ncbi.nlm.nih.gov/sra/SRR2005641, https://www.ncbi.nlm.nih.gov/nuccore/PV053298; occurrenceID: E1001A2A-E6ED-5124-8821-EA1AA88EEEA4; **Taxon:** scientificName: *Spinther bohnorum* Tilic & Rouse; kingdom: Animalia; phylum: Annelida; class: Polychaeta; family: Spintheridae; genus: Spinther; specificEpithet: *bohnorum*; taxonRank: species; scientificNameAuthorship: Tilic & Rouse; nomenclaturalCode: ICZN; **Location:** higherGeography: Pacific Ocean; continent: Oceania; waterBody: South Pacific Ocean; islandGroup: Society Islands; island: Mo'orea; country: French Polynesia; countryCode: FP; locality: between Opunohu Bay and Cook's Bay; verbatimDepth: 10-15 m; minimumDepthInMeters: 10; maximumDepthInMeters: 15; verbatimCoordinates: 17.4768°S, 149.8316°W; verbatimLatitude: 17.4768°S; verbatimLongitude: 149.8316°W; decimalLatitude: -17.4768; decimalLongitude: -149.8316; **Identification:** identifiedBy: Greg Rouse; **Event:** eventDate: 14-11-2010; year: 2010; month: 11; day: 14; verbatimEventDate: 11/14/2010; **Record Level:** institutionID: http://biocol.org/urn:lsid:biocol.org:col:14844; collectionID: http://grbio.org/cool/t8vy-919z; institutionCode: SIO; collectionCode: BIC; basisOfRecord: PreservedSpecimen
- **Type status:** Other material. **Occurrence:** catalogNumber: 1430143; recordedBy: K. White; S. El-Tourky,; individualCount: 1; occurrenceStatus: present; preparations: fixative: Ethanol; disposition: used up; previousIdentifications: *Spinther* sp.; associatedSequences: https://www.ncbi.nlm.nih.gov/nuccore/PV054334; occurrenceID: 1B25BF80-5028-5638-8626-CBCF147EB326; **Taxon:** scientificName: *Spinther bohnorum* Tilic & Rouse; kingdom: Animalia; phylum: Annelida; class: Polychaeta; family: Spintheridae; genus: Spinther; specificEpithet: *bohnorum*; taxonRank: species; scientificNameAuthorship: Tilic & Rouse; nomenclaturalCode: ICZN; **Location:** higherGeography: Pacific Ocean; continent: Oceania; waterBody: South Pacific Ocean; islandGroup: Society Islands; island: Mo'orea; country: French Polynesia; countryCode: FP; locality: Society Islands, Moorea, In front of Hilton; verbatimDepth: 15-17 m; minimumDepthInMeters: 15; maximumDepthInMeters: 17; verbatimCoordinates: -17.4756, -149.842; verbatimLatitude: -17.4756; verbatimLongitude: -149.842; decimalLatitude: -17.4756; decimalLongitude: -149.842; **Identification:** identifiedBy: Christopher Meyer; **Event:** eventDate: 05-12-2009; year: 2009; month: 12; day: 5; verbatimEventDate: 5 Dec 2009; fieldNotes: BIZ-230; **Record Level:** institutionID: http://biocol.org/urn:lsid:biocol.org:col:34871; institutionCode: USNM; basisOfRecord: DNAExtraction

#### Description

**Figure 3.**
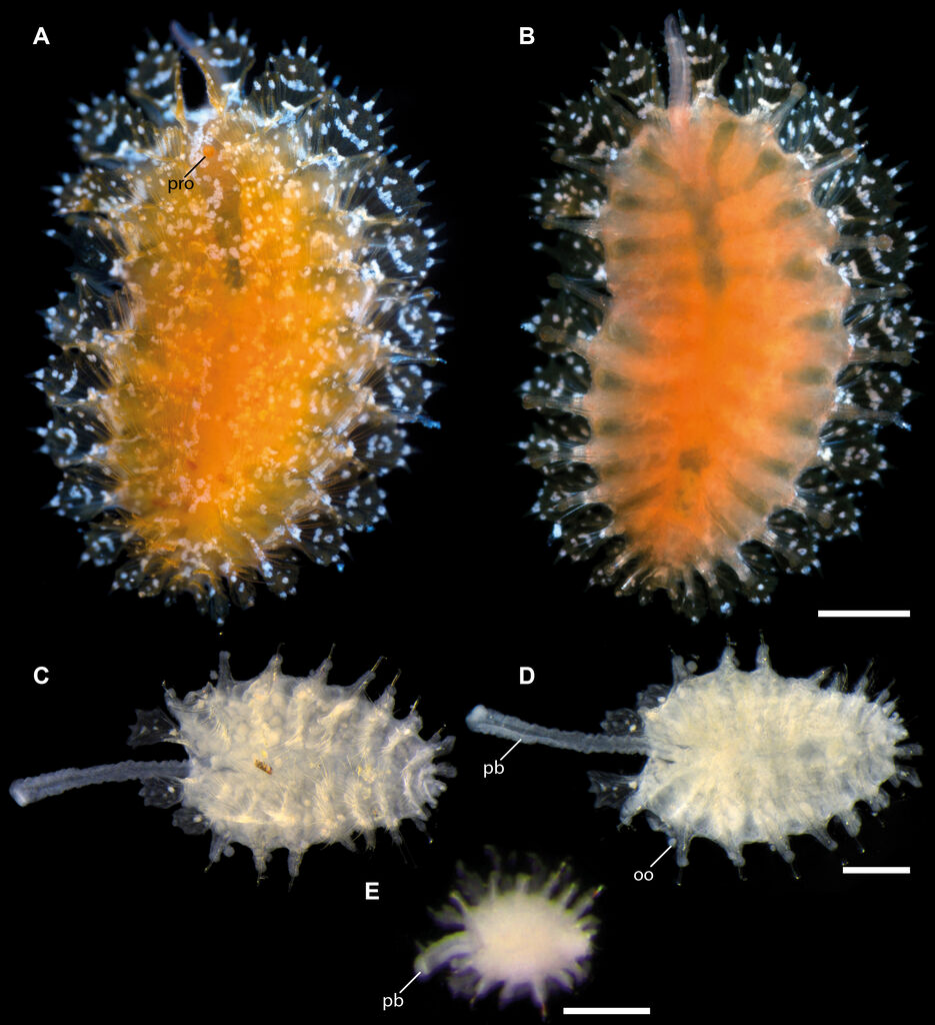
*Spinther bohnorum* Tilic & Rouse, **sp. nov.**, live and preserved specimens. **A, B** Live images of *S. bohnorum* sp. nov.; **A** Dorsal view, showing the notopodial fans covering the dorsum, the small prostomium (*pro*) and the bright orange colouration speckled with white spots; **B** Ventral view; **C, D** Holotype (*S. bohnorum*, SIO BIC A18597); **C** Dorsal view; **D** Ventral view, with long, protruded proboscis (*pb*) visible in all specimens and oocytes (*oo*) apparent in the holotype; **E** Paratype (*S. bohnorum*, SMF 32994), ventral view, also showing a protruded proboscis. Scale bars: 250 µm.

Holotype adult female with nine chaetigers, body 0.95 mm long, 0.63 mm wide at the widest point (excluding parapodia and chaetae). Paratype, possibly juvenile, with seven chaetigers (Fig. 3E). Body dorsoventrally flattened, oval. Live animals bright orange/red speckled with small white spots dorsally (Fig. 3A). Preserved specimens in alcohol white, with faint white spots visible on delicate fan-like notopodial lamellae (Fig. 3C). Notopodial fans covering the dorsum, except for the anteroposterior mid-line.

**Figure 4.**
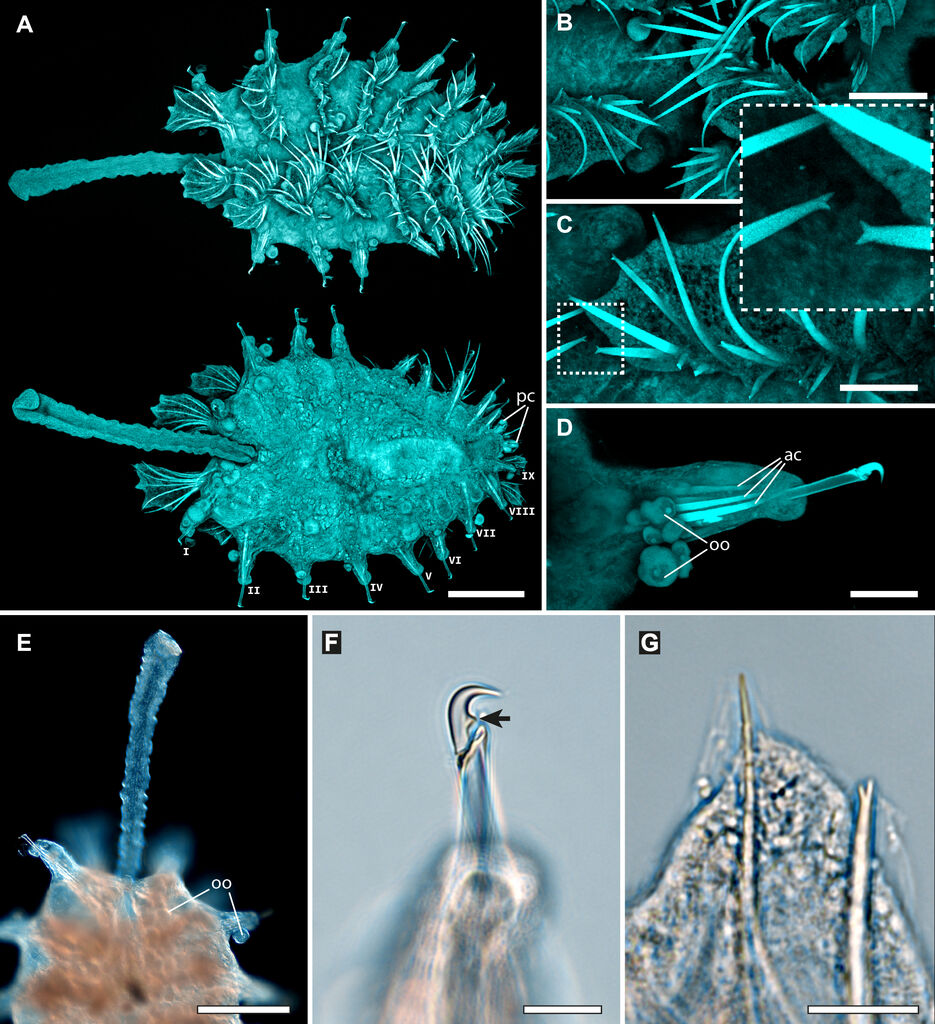
*Spinther bohnorum* Tilic & Rouse, **sp. nov.**, holotype (SIO BIC A18597). (A–D) CLSM images showing autofluorescence. **A** Overview of dorsal and ventral views. The chaetigers are numbered with Roman numerals and the pygidial cirri (pc) are shown in the ventral view; **B, C** Notochaetae, with inset highlighting the bifid distal ends with spread tips; **D** Neuropodium, showing internal aciculae (*ac*), a single protruding falcate compound hook and oocytes (*oo*); **E** Light microscopy (LM) image showing the protruded proboscis and oocytes; **F** Close-up of a compound neuropodial hook, with the lateral tooth marked by an arrow; **G** Detailed view of the distal ends of both bifid and entire notochaetae. Scale bars: 250 µm (A, E), 100 µm (B), 50 µm (C, D), 25 µm (F, G).

Eyespots not visible. Prostomium small, spherical lobe situated dorsally between notopodial fans of chaetiger 2 (Fig. 3A). Mouth ventral, located posterior to chaetiger 1. Proboscis protruded, long (0.84 mm), with a mid-ventral groove and ventrally folded tip (Fig. 4A). Notopodia dorsally radiating, with thin, delicate lamellae between notochaetae (Fig. 3A, Fig. 4A). Most notochaetae bifid, with only a few entire ones irregularly distributed amongst them (Fig. 4G), all similar in thickness, distal parts of bifid notochaetae spread (Fig. 4C). Neuropodia cylindrical with slightly enlarged tips, without parapodial extensions (Fig. 4D). A single, compound neuropodial hook projects distally from each neuropodium. Neuropodial hooks strongly curved, with a prominent lateral tooth (Fig. 4F); 3-4 distally pointed aciculae embedded in neuropodia (Fig. 4D). Pygidium with two elliptical pygidial cirri (Fig. 4A).

Spherical oocytes (diameter ±30 µm) visible through the body wall and attached to neuropodia (Fig. 3D, Fig. 4D, E).

##### Type material

Holotype (SIO BIC A2846) and one paratype (SMF 32994). Both specimens were collected between Opunohu Bay and Cook’s Bay, Mo'orea, Society Islands, French Polynesia, at depths of 10–15 m on 14 November 2010. The holotype is a female adult; the paratype is likely a juvenile.

##### Material examined

Holotype (SIO BIC A2846), paratype (SMF 32994) and two additional specimens:

- one specimen collected at the same type locality on 14 November 2010 (unregistered, adult);
- one specimen collected in front of the Hilton, Mo'orea, Society Islands, French Polynesia (17.4756°S, 149.842°W), at 15–17 m depth on 5 December 2009 (USNM 1430143; associated with DNA extraction).

##### Type locality

South Pacific Ocean, Society Islands, French Polynesia, between Opunohu Bay and Cook’s Bay, Mo'orea (17.4768°S, 149.8316°W), at depths of 10–15 m.

#### Diagnosis

Small species (ca. 1 mm) with bright red-orange colouration and white speckles dorsally. Ventral surface generally smooth. Neuropodia lacking parapodial extensions, with neurochaetae as falcate compound hooks bearing a marked lateral tooth. Notochaetae comprising both entire and bifid forms, with bifid chaetae having spread distal ends.

#### Etymology

*Spinther bohnorum* **sp. nov.** is named after Brenda and Jeffrey Bohn and their family in appreciation of their steadfast support for marine invertebrate taxonomy and biodiversity research.

#### Distribution

Known only from Mo'orea, French Polynesia.

#### Taxon discussion

*Spinther* is a small and easily recognised group of polychaetes, comprising only 12 accepted species reported worldwide, though half have been named from the North Pacific (Table 2). While the taxonomic history of *Spinther* is not extensive, it has been notably convoluted. Similarly, its phylogenetic affinities and placement within the annelid tree of life have long been a subject of debate (Rouse et al. 2022). Despite advances in molecular approaches, the placement of *Spinther* within the annelid tree of life remains unresolved. Andrade et al. (2015) included the transcriptome of *Spinther bohnorum* **sp. nov.** in their phylogenomic study, but their results were inconclusive, likely due to long-branch effects (see also Tilic et al. (2022)). *Spinther* is a monotypic genus, classified as Aciculata *incertae sedis* with uncertain affinities (Rouse et al. 2022), rendering the family name Spintheridae redundant.

Most *Spinther* species live on the surface of sponges, clinging to their hosts using their hooked neurochaetae. Their bright colouration often matches the sponge, providing camouflage. Whether *Spinther* is parasitic or a highly adapted commensal remains unclear. In the case of *Spinther bohnorum* **sp. nov.**, it is also likely to associate with red sponges, given its colouration. However, this species was not collected directly on a sponge, but rather found in washings of coral rubble, suggesting a possible association yet to be confirmed.

Johnston (1845) introduced the name *Spinther oniscoides* Johnston, 1845 for specimens from Ireland. A few years later, Sars (1851), unaware of Johnston’s work, described *Oniscosoma arcticus* Sars, 1851 from northern Norway. *Oniscosoma* is now considered a junior synonym of *Spinther* (Sars 1862). Stimpson (1854), apparently unaware of both Johnston’s and Sars’ studies, described *Cryptonota citrina* Stimpson, 1854 from the Bay of Fundy, Canada, which was also later regarded as a junior synonym of *Spinther* (Sars 1862). Grube (1860) described *S. miniaceus* Grube, 1860 from the Adriatic Sea. Wirén (1883) subsequently used the name *S. arcticus* (Sars, 1851) for a species from the Bering Sea, considering it sufficiently similar to Sars’ species. However, Augener (1928), page 672, disputed this, asserting that Wirén’s specimen, collected during the Vega expedition, was distinct enough to warrant a new species. Augener proposed the name *S. vegae* Augener, 1928 for Wirén’s material, but only mentioned it in passing, without a formal description. Subsequently, Graff (1888) re-described the same specimen under the name *S. arcticus*. Augener criticised this approach, stating that it was inadmissible for Graff to retain the name *S. arcticus* for what he considered a different species. Despite this, *S. vegae* was never properly validated. Adding to the confusion, Hartman (1948) incorrectly interpreted Wirén (1883) as having created a homonym and replaced the name *S. arcticus* Wirén, 1883 with *S. wireni* Hartman, 1948. However, this replacement name was unnecessary and is now considered invalid. Riddell (1909) re-described *S. oniscoides* and highlighted that Graff had misidentified it, showing that Graff’s specimens actually belonged to *S. citrinus*. Levinsen (1883)'s *Spinther major* Levinsen, 1883 is also treated as a junior synonym of *S. oniscoides* by McIntosh (1900). Augener (1913) introduced *S. australiensis* Augener, 1913 from Western Australia. Hartman (1948) listed six species as valid within *Spinther*, including her new species, *S. alaskensis* Hartman, 1948. She treated *S. miniaceus* Grube, 1860 as a junior synonym of *S. arcticus* (Sars, 1851). In the western Pacific, *S. hystrix* Uschakov, 1950 was described from the Sea of Okhotsk, while *S. japonicus* Imajima & Hartman, 1964, *S. ericinus* Yamamoto, 1985 and *S. sagamiensis* Imajima, 2003 were described from Japan. Hartman (1967) also added *S. usarpia* Hartman, 1967 from the Antarctic. These contributions, alongside *S. bohnorum* **sp. nov.**, bring the total number of nominal species to 15. A detailed and integrative investigation of *Spinther* species, combining morphological and molecular data, is needed to confirm or reject some of the proposed synonymies.

*Spinther bohnorum* **sp. nov.** most closely resembles *S. australiensis* in having falcate neurochaetae with a lateral tooth and no parapodial extension. However, *S. bohnorum* **sp. nov.** differs in possessing both bifid and entire notochaetae, whereas *S. australiensis* only has bifid notochaetae. Additionally, *S. bohnorum* **sp. nov.** exhibits a striking red-orange colouration with white speckles, while *S. australiensis* was originally described by Augener (1913) as “dull ochre-yellowish with whitish, colourless skin fans” (matt-ockergelblich mit weißlich farblosen Hautkämmen). Finally, *S. bohnorum* **sp. nov.** is much smaller, with the largest specimen having only 13 chaetigers and the mature female holotype having just nine chaetigers (± 1 mm body length), compared to *S. australiensis*, which was described with 23–31 chaetigers (> 4.5 mm body length).

The COI sequence obtained for a *Spinther bohnorum* **sp. nov.** specimen (USNM 1430143; GenBank: PV054334) matches the COI from the published transcriptome sequence (GenBank: PV053298) and was one of several generated as part of the Mo'orea Biocode project. The other two *Spinther* COI sequences generated (extractions catalogued as USNM 1430144; GenBank: PV054335 and USNM 143322) are arguably for different *Spinther* species, being 8–9% divergent from *Spinther bohnorum* **sp. nov.** and from each other. Unfortunately, there is no voucher material directly associated with these sequences. There are additional *Spinther* specimens collected as part of the Moorea Biocode project held at SIO-BIC and at the Florida Museum of Natural History, but the former were all fixed in formalin and those from the latter have not been sequenced. The presence of sympatric species of *Spinther* at Mo'orea warrants further investigation.

#### Methods

Specimens were obtained from coral rubble collected via SCUBA which was then treated with suspension-decantation and sieving. Live specimens were imaged using a LEICA MZ9.5 stereomicroscope with a CANON Rebel T1i camera. The formalin-fixed holotype was transferred from 70% ethanol to a 1:1 mixture of 99.5% undenatured ethanol and 98% glycerine prior to imaging. Over several days, the solution was gradually replaced by glycerine to allow a gentle transition from ethanol. This clearing step improves tissue transparency and enhances visualisation of chaetae for imaging. The specimen was photographed in dorsal and ventral views using a motorised NIKON SMZ25 stereomicroscope, equipped with a NIKON Digital Sight 10 camera, with image stacks processed using NIKON NIS Elements Basic Research software (v. 5.42.04). Confocal laser scanning microscopy (CLSM) scans were performed without staining (autofluorescence) using a LEICA DM2500 microscope, 405 nm excitation wavelength, a LEICA ACS APO 10× objective (dry) and LAS X (v. 3.5.7.23225) software. Image stacks were processed in Fiji (v. 1.54f). Distal ends of noto- and neurochaetae were imaged without differential interference contrast using a NIKON Eclipse Ni microscope at 40× magnification.

For molecular analyses, a COI sequence was obtained via Sanger sequencing for a *Spinther bohnorum* **sp. nov.** specimen (USNM 1430143) GenBank: PV054334, for which only the extraction remains. This was generated as part of the Moorea Biocode project (https://ocean.si.edu/ecosystems/coral-reefs/welcome-moorea-biocode-project).

The transcriptome assembly of *Spinther bohnorum* **sp. nov.** was used as a BLAST database. The *Spinther* COI sequence was used as a BLAST query to identify and mine the COI barcode sequence, which was subsequently uploaded to GenBank (accession number PV053298).

Specimen data for this description were (in parts) gathered and processed via the Discovery Laboratory of the SENCKENBERG OCEAN SPECIES ALLIANCE.

**Repository**: Type material are deposited at the Benthic Invertebrate Collection, Scripps Institution of Oceanography (SIO), Senckenberg Research Institute and Natural History Museum, Frankfurt (SMF) and Smithsonian National Museum of Natural History (USNM).

### Craspedochiton zefranki

Vončina sp. nov.

AB07D3EA-4BF3-5CAD-875C-12E23621F389

894373F8-FBEF-4035-9AF8-96939980EB02

https://www.ncbi.nlm.nih.gov/nuccore/PV664593

#### Materials

- **Type status:** Holotype. **Occurrence:** catalogNumber: MNHN-IM-2019-34865; recordedBy: leg. Bouchet, Dayrat, Warén & Richer de Forges-IRD; individualCount: 1; lifeStage: adult; preparations: EtOH 100%, partly disarticulated + SEM stubs with parts of girdle and radula; associatedSequences: GenBank nr PV664593; occurrenceID: B359911D-085D-512F-8E8E-407151DD3145; **Taxon:** scientificName: *Craspedochiton zefranki* Vončina; kingdom: Animalia; phylum: Mollusca; class: Polyplacophora; order: Chitonida; family: Acanthochitonidae; genus: Craspedochiton; specificEpithet: *zefranki*; scientificNameAuthorship: Vončina; nomenclaturalCode: ICZN; **Location:** higherGeography: Pacific Ocean; continent: Oceania; country: Solomon Islands; locality: NW San Cristobal; minimumDepthInMeters: 97; maximumDepthInMeters: 223; verbatimLatitude: 10°17'S; verbatimLongitude: 161°43'E; **Identification:** identifiedBy: Katarzyna Vončina; dateIdentified: 21-04-2024; **Event:** eventDate: 06-10-2001; eventRemarks: Expedition: Solomon 1, Station: DW1840, Ship: Alis; **Record Level:** institutionCode: MNHN; collectionCode: IM; basisOfRecord: PreservedSpecimen
- **Type status:** Paratype. **Occurrence:** catalogNumber: SMF 380885; recordedBy: leg. Bouchet, Dayrat, Warén & Richer de Forges-IRD; individualCount: 1; lifeStage: adult; preparations: EtOH 100%; otherCatalogNumbers: MNHN-IM-2019-35217; occurrenceID: B12459B8-0BC6-59F6-805F-02F09F435567; **Taxon:** scientificName: *Craspedochiton zefranki* Vončina; kingdom: Animalia; phylum: Mollusca; class: Polyplacophora; order: Chitonida; family: Acanthochitonidae; genus: Craspedochiton; specificEpithet: *zefranki*; scientificNameAuthorship: Vončina; nomenclaturalCode: ICZN; **Location:** higherGeography: Pacific Ocean; continent: Oceania; country: Solomon Islands; locality: NW San Cristobal; verbatimLatitude: 10°17'S; verbatimLongitude: 161°43'E; **Identification:** identifiedBy: Katarzyna Vončina; dateIdentified: 2024-21-04; **Event:** eventDate: 06-10-2001; **Record Level:** institutionCode: SMF; basisOfRecord: PreservedSpecimen

#### Description

**Figure 5.**
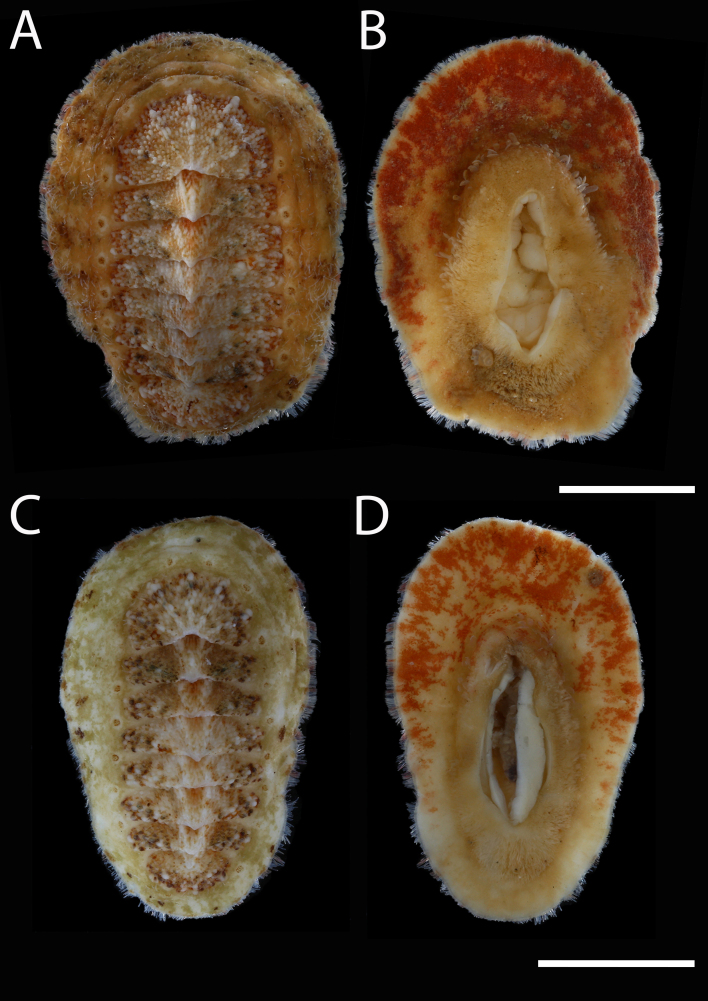
*Craspedochiton zefranki* Vončina, **sp. nov. A, B** Holotype MNHN-IM-2019-34865, dorsal and ventral view, respectively; **C, D** Paratype SMF 380885, dorsal and ventral view, respectively. Scale bar: 5 mm.

Body of small size (holotype: 16 x 10 mm, paratype: 13 x 8 mm), oval, carinated, moderately elevated (quotient of valve II = 0.31), side slopes slightly concave, valves solid, beaked. Tegmentum densely granulated, girdle expanded anteriorly (Fig. 5).

**Figure 6.**
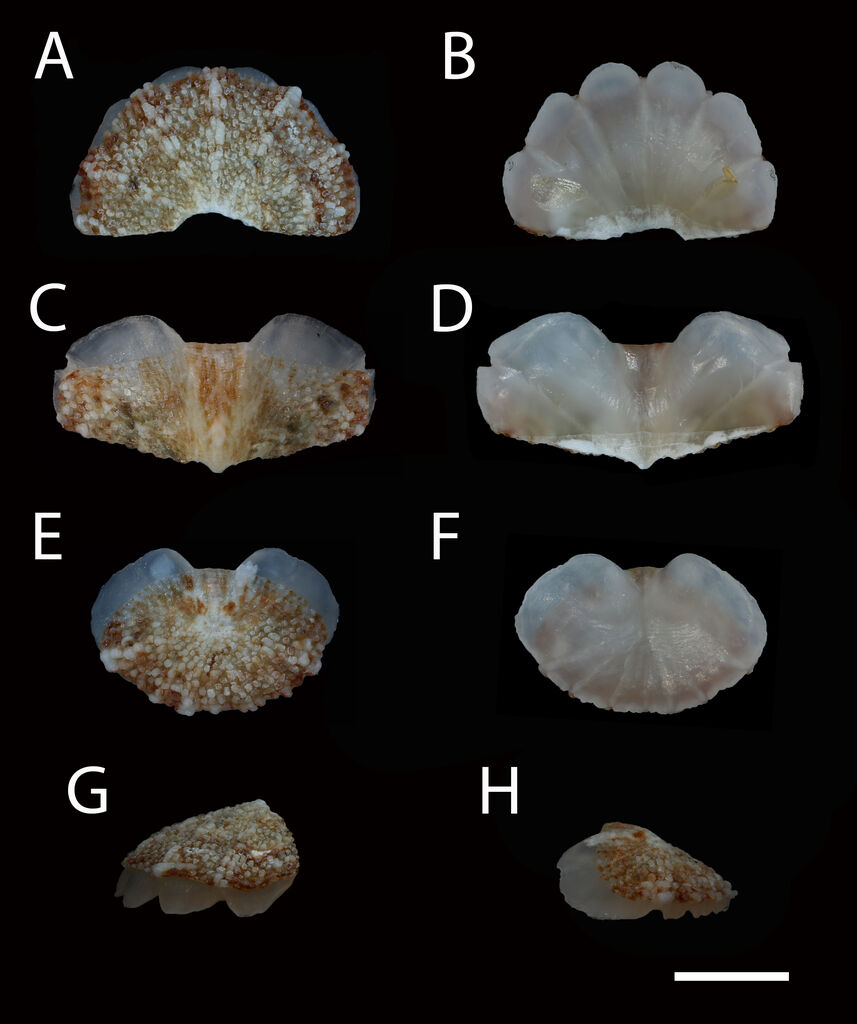
*Craspedochiton zefranki* Vončina, **sp. nov.**, holotype MNHN-IM-2019-34865. **A, C, E** Valves I, II, VIII, respectively, dorsal view; **B, D, F** Valves I, II, VIII, respectively, ventral view; **G, H** Valve I, VIII, respectively, lateral view. Scale bar: 250 μm.

**Figure 7.**
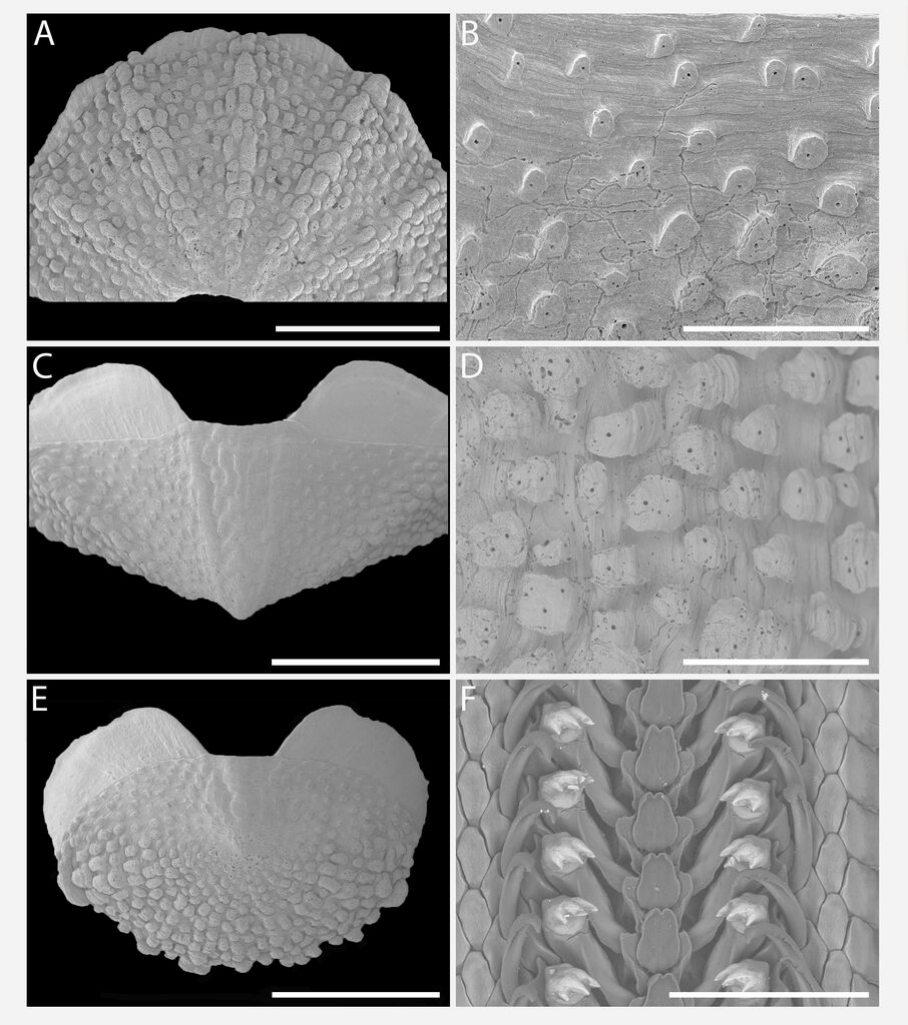
*Craspedochiton zefranki* Vončina, **sp. nov.**, holotype MNHN-IM-2019-34865. **A, C, E** Valve I, II, VIII; **B, D** Details of tegmental surface of valve II in the anterior and posterior part of lateropleural area, respectively; **F** Central portion of radula. Scale bars: 2 mm (A, C, E), 500 µm (B, D), 200 µm (F).

Tegmentum ground colouration yellowish, dark orange at the valves edges and jugum, with mostly white pustules and irregular small green maculation; girdle yellow to orange with irregular brownish patches and white blotches around the tufts (Figs 5, 6). Dorsum, despite well-defined jugum, hardly keeled, side slopes straight to slightly convex. Tegmentum densely covered with polymorphic pustules (Figs 6, 7A–E).

Head valve semicircular with rather straight posterior margin, notched in the middle, with five elevated ribs consisting of irregular, elongate pustules, larger towards anterior and side margins of the valve, pustules between the ribs smaller, roundish in the apical region and larger, more elongate towards the margins (Fig. 6A, G, Fig. 7A). Intermediate valves roughly rectangular, weakly produced forward at jugal part, the posterior edge of the valve having a central protrusion that extends outwards, creating a small beak, flanked by straight edges on either side (Fig. 6C, Fig. 7C). Jugal area with wavy ridges, wedge-shaped, separated from the lateropleural areas. Lateropleural areas moderately elevated with an inconspicuous diagonal ridge consisting of slightly elevated and larger pustules, separating areas with different kind of granulation: anterior part covered with sparsely distributed, small and oval pustules (Fig. 6C, Fig. 7B, C) and posterior part with densely arranged, much larger and irregular pustules (Fig. 6C, Fig. 7C, D). Tail valve small, almost circular, slightly wider than long, with antemedian flat mucro, straight postmucronal slope; seven inequidistance elevated ribs formed by elevated large pustules correspond to the articulamentum slits; granulation of the tail valve similar to the intermediate valves, with the antemucronal area covered with small oval pustules and the postmucronal area with densely distributed, much larger and elongate pustules (Fig. 6E, H, Fig. 7E).

Articulamentum strongly developed, creamy-pink, but white in apophyses and under the pleural areas of the intermediate valves. Slit formula: 5/1/7. Slits deep and wide, smooth, but with distinct dorsal grooves; insertion plates long and wide. Apophyses well-developed, rounded in a head valve, trapezoidal in valve II and very short, but wide, rectangular with rounded edges in tail valve (Fig. 6B, D, F).

**Figure 8.**
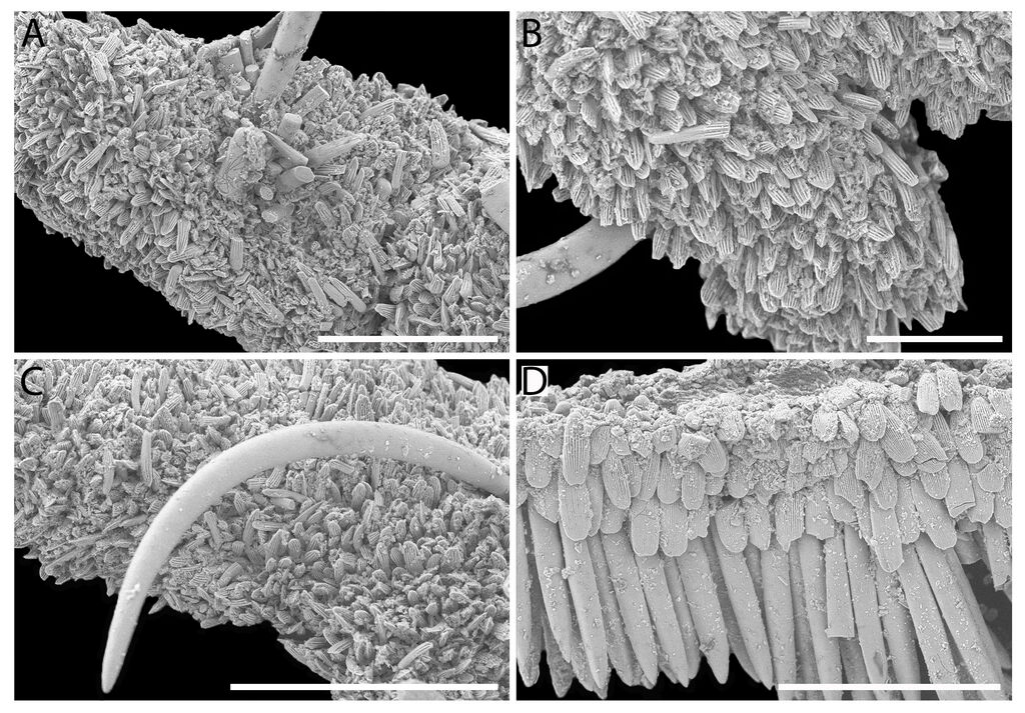
*Craspedochiton zefranki* Vončina, **sp. nov.**, holotype MNHN-IM-2019-34865. **A, B** Dorsal girdle spicules from the anterior part of perinotum; **C** Dorsal girdle spicules and hair from the anterior part of perinotum; **D** Marginal spicules. Scale bars: (1) 200 µm (A), 100 µm (B), 300 µm (C, D).

**Figure 9.**
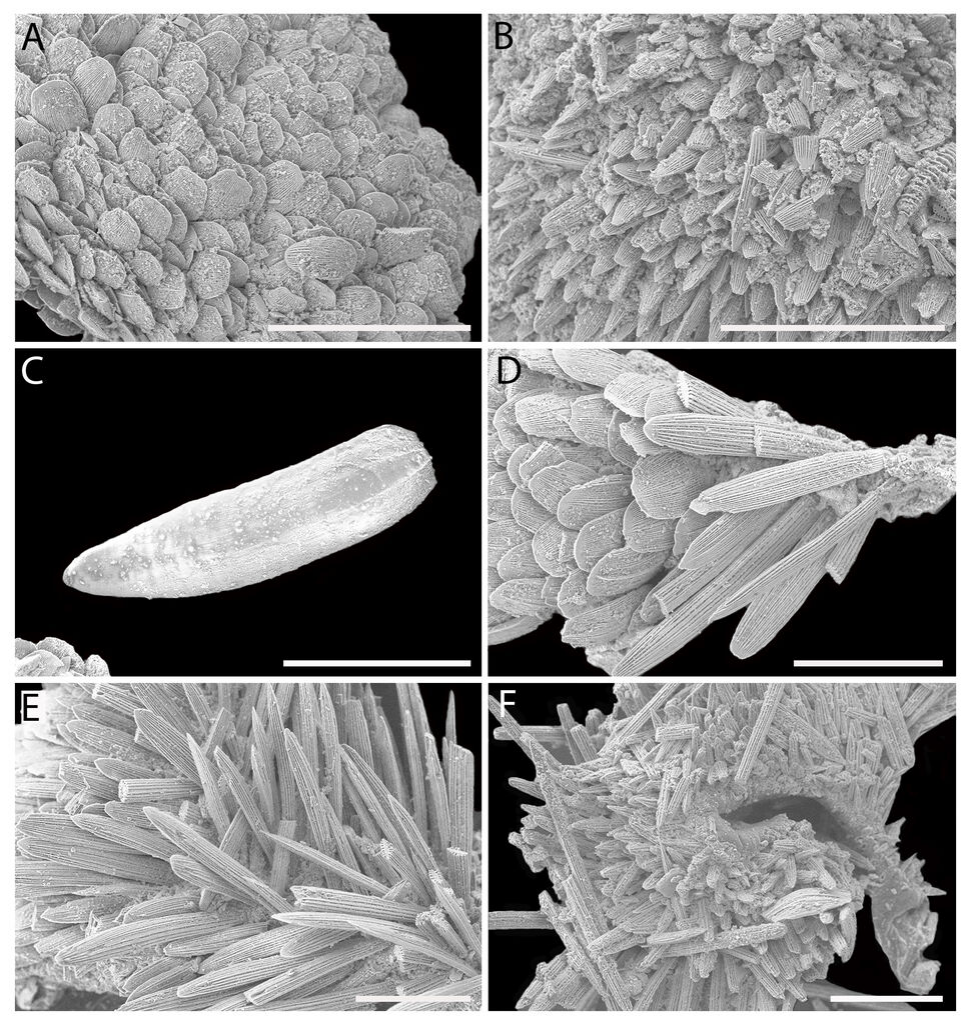
*Craspedochiton zefranki* Vončina, **sp. nov.**, holotype MNHN-IM-2019-34865. **A–F** Ventral spicules. **A** Spicules of the central part of hyponotum; **B** Spicules of the posterior part of hyponotum; **C** Single spicule from the anterior part of the mantle fold; **D, E** Spicules from the central part of the mantle fold; **F** Spicules from the posterior part of the mantle fold. Scale bars: 300 µm (A, B) 400 µm (C), 200 µm (D, E, F).

Girdle anteriorly expanded, light orange with irregular brownish patches and white blotches around the tufts. Dorsally densely covered with short, flattened spicules, with distinct radial ribs in their upper half – ribs usually not reaching the tip which tends to be tapered and smooth, L: 56–62 μm (mean = 58 μm, n = 10), W: 10–15 μm (mean = 12 μm, n = 10), intermingled with long, randomly, but densely distributed hair-like, smooth long spicules, usually bent, L: 210–420 μm (mean = 318 μm, n = 3), W: 33–42 μm (mean = 37 μm, n = 3) (Fig. 8A–C); 18 sutural tufts indistinct, consisting of very short (likely broken), thick, straight spicules. Marginal fringe of elongate, rounded, straight and point-ended spicules (some spicules striated), L: 322–347 μm (mean = 340 μm, n = 3), W: 37–50 μm (mean = 42 μm, n = 3) (Fig. 8D). Ventral spicules densely arranged, tiled, polymorphic, very elongate to oval with a clear zonation. The anterior and middle part of hyponotum covered with longitudinally densely striated, flattened scale-like spicules, L: 75–120 μm (mean = 87.6 μm, n = 7), W: 40–75 μm (mean = 53.4 μm, n = 7) (Fig. 9A). The posterior part of hyponotum covered with elongate, flattened, sharply pointed or slightly tapered and deeply striated spicules, L: 170 μm, W: 30 μm, n = 1, (Fig. 9B). Mantle fold with smooth, flat, elongated scales at the edges (L: 800 μm, W: 200 μm, n=1) and very long, straight, deeply striated spicules, L: 451–553 μm (mean = 499 μm, n = 8), W: 21–44 μm (mean = 32.5 μm, n = 8), intermingled with similar, but much shorter spicules (up to 250 μm), more abundant closer to the interior part of the mantle fold (Fig. 9C–F).

Radula of holotype small, ca. 3 mm in length, with 46 rows of teeth, of which 40 are matured. Central tooth subrectangular, the apical edge is very thin and folded, which gives it a bicuspid look, with wide base and tapering towards the top, the antero-lateral corner of the centro-lateral tooth is obtuse and smooth, thin. First lateral tooth elongate, major lateral tooth robust, with tricuspid head, denticles pointed, of similar size (Fig. 7F).

Gills merobranchial, six ctenidia per side.

##### Type material

Holotype (MNHN-IM-2019-34865) now partly disarticulated; parts of girdle and radula on two SEM stubs and the vial with the specimen stored in 96% ethanol; and one paratype (SMF 380885) stored in 96% ethanol.

##### Material examined

Only known from the type material.

##### Type locality

Solomon Islands, NW San Cristobal, 10°17'S, 161°43'E, 97–223 m depth.

#### Diagnosis

Chitons of small size, up to 15 mm, body oval, girdle expanded anteriorly; colour of the tegmentum yellowish, mottled with dark orange and green; girdle yellow to orange with brownish maculation and white patches around the tufts. Valves carinated, moderately elevated, densely covered with pustules. Head valve with five distinct ribs, tail valve almost circular, mucro antemedian, flat. Perinotum covered with short, ribbed spicules and scattered with hair-like long spicules; hyponotum covered with scale-like, deeply striated spicules and very long thin spicules on the mantle fold.

#### Etymology

The specific epithet *zefranki* is a masculine adjective formed from the name of Hosea Jan "Ze” Frank, an online performance artist known for his wit, creativity and humorous approach to scientific knowledge in the YouTube series TRUE FACTS. The name honours his influential contributions to internet culture and the vision he has brought to the SENCKENBERG OCEAN SPECIES ALLIANCE as a member of its advisory board.

#### Distribution

At present known only from its type locality, the Solomon Islands.

#### Taxon discussion

**Morphological discussion**

The genus *Craspedochiton* consists of 14 currently accepted species and five of them belong to *Thaumastochiton*-group Thiele, 1909 (Schwabe and Els 2019) which is characterised by the uplifted posterior mucro and unslitted callus in the tail valve. As *Craspedochiton zefranki* **sp. nov.** has antemedian positioned mucro, it surely does not belong to this group. The new species can be immediately distinguished from the rest of the species belonging to the same genus by its roundish insertion plates in the head valve (in all other species they are rectangular). Additionally, below there are provided more detailed differences between the new species and other *Craspedochiton* reported from the similar geographic range. *C. zefranki* **sp. nov.** differs from:

*Craspedochiton elegans* (Iredale & Hull, 1925) by sculpture of the valves (small, fine pustules in *C. elegans* vs. much larger, coarser pustules in *C. zefranki* **sp. nov.**), by the shape of the tail valve (rhomboidal in *C. elegans* vs. roundish in *C. zefranki* **sp. nov.**), position of the mucro (in posterior third and elevated in *C. elegans* vs. antemedian and flat mucro in *C. zefranki* **sp. nov.**);

*Craspedochiton hystricosus* Kaas, 1991 by the sculpture of the valves (much finer, smaller pustules, roundish or elongate vs. much larger, irregular, but squarish in *C. zefranki* **sp. nov.**), longitudinal ridges in the pleural area of the intermediate valves (present in *C. hystricosus* vs. absent in *C. zefranki* **sp. nov.**), position of mucro (more posteriorly located in *C. hystricosus* vs. antemedian in *C. zefranki* **sp. nov.**), ridges on the head valves (only weakly indicated in *C. hystricosus* vs. conspicuous in *C. zefranki* **sp. nov.**);

*Craspedochiton jaubertensis* Ashby, 1924 by the sculpture of the valves (much larger, solid pustules in *C. jaubertensis* vs. smaller, less coarse pustules in *C. zefranki* **sp. nov.**), the shape of the valves (median trapezoidal in *C. jaubertensis* vs. rectangular in *C. zefranki* **sp. nov.**), position of mucro (more central in C. *jaubertensis* vs. more anteriorly located in *C. zefranki* **sp. nov.**), apophyses in tail valve (longer, more produced forward in *C. jaubertensis* vs. shorter, reaching the postmucronal areas in *C. zefranki* **sp. nov.**);

*Craspedochiton laqueatus* (G. B. Sowerby II, 1842; Dell’Angelo et al. (2010) – illustrations of type material) by the sculpture (larger, coarser pustules, especially in the pleural areas of intermediate valves in *C. laqueatus* vs. smaller, more densely arranged pustules, rounded and small in pleural areas of intermediate valves in *C. zefranki* **sp. nov.**), by the shape of the second valve (convex anterior edge, protruding beak flanked by concave posterior edges in *C. laqueatus* vs. almost straight, slightly protruding at jugal part and a small beak flanked by straight posterior edges in *C. zefranki* **sp. nov.**), morphology of hyponotum scales (less numerous *striae* in *C. laqueatus* vs. much more numerous *striae* in *C. zefranki* **sp. nov.**);

*Craspedochiton tesselatus* Nierstrasz, 1905 by the shape of the apophyses in the head valve (rectangular in *C. tesselatus* vs. roundish in *C. zefranki* **sp. nov.**), position of mucro and postmucronal slope (median mucro and strongly concave slope in *C. tesselatus* vs. antemedian mucro and straight slope in *C. zefranki* **sp. nov.**).

**Molecular discussion**

The obtained sequence was positively checked as belonging to Polyplacophora against the GenBank database; however, with only ca. 86% similarity to published sequences. The closest relative in GenBank was *Acanthochitona ferreirai* W. G. Lyons, 1988 (86.02% similarity, acc. No. MK016365.1). The number of publicly available sequences of *Craspedochiton* is relatively low (2 COI sequences belonging to two species). A phylogenetic analysis which could explain low similarity to the other sequences from the same genus was not conducted; the COI barcode of the new species is provided for future use.

#### Methods

Live animals were collected at depths of 97–223 m by the French expedition SOLOMON 1 at Station DW1840 near the Solomon Islands. Specimens were fixed in 100% ethanol. The systematic classification follows Sirenko (2006), the morphological nomenclature following Schwabe (2010).

For scanning electron microscopy (SEM), the valves and radula were removed, cleaned with a diluted bleach solution (1:1 with H_2_O) and rinsed in distilled water. Several small pieces of dorsal and ventral girdle were sampled, as the spicules tend to be highly polymorphic at intra-individual level, following Schwabe and Els (2019) recommendations. Girdle tissue was only air dried. Objects were placed on SEM stubs using double-sided adhesive tabs. Samples were examined with a HITACHI TM4000 tabletop SEM. After SEM examination, spicules which best represented variability of the species girdle were presented in the figures. All figures were assembled in Adobe Photoshop.

For DNA barcoding, a small fragment of tissue from the holotype’s foot was sampled. DNA was extracted using QIAamp DNA Micro Kit (QIAGEN), following the manufacturer’s protocol. The cytochrome oxidase subunit I (COI primers LCO1490 and HCO2198; Folmer et al. (1994)) was amplified using TaKaRa Taq HS Perfect Mix from TaKaRa. The PCR conditions involved an initial denaturation step at 94ºC for 5 minutes; then 35 cycles of denaturation at 94ºC for 45 seconds, annealing at 50ºC for 45 seconds and extension at 72°C for 1 minute and 30 seconds; followed by a final extension step at 72°C for 5 minutes. The obtained sequence was manually inspected and trimmed to the length 612 bp in Geneious Prime v.2023.1 and was made publicly available on GenBank under accession number PV664593.

Specimen data for this description were (in parts) processed via the Discovery Laboratory of the SENCKENBERG OCEAN SPECIES ALLIANCE.

Abbreviations used in the text are as follows: Muséum National d'Histoire Naturelle, Paris, France (MNHN); Senckenberg Research Institute and Natural History Museum Frankfurt (SMF).

**Repository**: Holotype (MNHN-IM-2019-34865) is now deposited in the collection of MNHN; one paratype (SMF 380885, old MNHN number: MNHN-IM-2019-35217) is deposited in the malacological collection of SMF.

### Ferreiraella charazata

Sigwart sp. nov.

F6D468B7-8F43-5766-997F-556B750653A4

65EDCFF9-FFAD-4B64-8674-31BE9A989BC4

#### Materials

- **Type status:** Holotype. **Occurrence:** catalogNumber: SCSMBC240287; recordedBy: ROV ROPOS on R/V TAN KAH KEE; individualCount: 1; lifeStage: adult; occurrenceID: 1E1FA6C7-42D2-56D6-B5D2-BBC070119095; **Taxon:** scientificName: *Ferreiraella charazata* Sigwart; kingdom: Animalia; phylum: Mollusca; class: Polyplacophora; order: Lepidopleurida; family: Abyssochitonidae; genus: Ferreiraella; specificEpithet: charazata; taxonRank: species; scientificNameAuthorship: Sigwart; nomenclaturalCode: ICZN; **Location:** higherGeography: Pacific Ocean; waterBody: Western Pacific Ocean; verbatimDepth: 3003 m; verbatimCoordinates: 21°18.3411N, 119°11.7281E; verbatimLatitude: 21°18.3411N; verbatimLongitude: 119°11.7281E; verbatimCoordinateSystem: degrees decimal minutes; **Identification:** identifiedBy: Julia D. Sigwart; dateIdentified: 2024; **Event:** samplingProtocol: remotely operated vehicle (ROV) ROPOS on R/V TAN KAH KEE; eventDate: 9-May-18; **Record Level:** institutionCode: SCSMBC; basisOfRecord: PreservedSpecimen
- **Type status:** Paratype. **Occurrence:** catalogNumber: SMF 380825; recordedBy: ROV ROPOS on R/V TAN KAH KEE; individualCount: 1; lifeStage: adult; occurrenceID: 5EFBBA70-2307-5E1C-A9C9-BC95C0190D7F; **Taxon:** scientificName: *Ferreiraella charazata* Sigwart; kingdom: Animalia; phylum: Mollusca; class: Polyplacophora; order: Lepidopleurida; family: Abyssochitonidae; genus: Ferreiraella; specificEpithet: charazata; taxonRank: species; scientificNameAuthorship: Sigwart; nomenclaturalCode: ICZN; **Location:** higherGeography: Pacific Ocean; waterBody: Western Pacific Ocean; verbatimDepth: 3003 m; verbatimCoordinates: 21°18.3411N, 119°11.7281E; verbatimLatitude: 21°18.3411N; verbatimLongitude: 119°11.7281E; verbatimCoordinateSystem: degrees decimal minutes; **Identification:** identifiedBy: Julia D. Sigwart; dateIdentified: 2024; **Event:** samplingProtocol: remotely operated vehicle (ROV) ROPOS on R/V TAN KAH KEE; eventDate: 9-May-18; **Record Level:** institutionCode: SMF; basisOfRecord: PreservedSpecimen
- **Type status:** Paratype. **Occurrence:** catalogNumber: SMF 380826; recordedBy: ROV ROPOS on R/V TAN KAH KEE; individualCount: 1; lifeStage: adult; occurrenceID: B886AD47-B4EF-57D5-B2BD-FBDFA0A0ED69; **Taxon:** scientificName: *Ferreiraella charazata* Sigwart; kingdom: Animalia; phylum: Mollusca; class: Polyplacophora; order: Lepidopleurida; family: Abyssochitonidae; genus: Ferreiraella; specificEpithet: charazata; taxonRank: species; scientificNameAuthorship: Sigwart; nomenclaturalCode: ICZN; **Location:** higherGeography: Pacific Ocean; waterBody: Western Pacific Ocean; verbatimDepth: 3003 m; verbatimCoordinates: 21°18.3411N, 119°11.7281E; verbatimLatitude: 21°18.3411N; verbatimLongitude: 119°11.7281E; verbatimCoordinateSystem: degrees decimal minutes; **Identification:** identifiedBy: Julia D. Sigwart; dateIdentified: 2024; **Event:** samplingProtocol: remotely operated vehicle (ROV) ROPOS on R/V TAN KAH KEE; eventDate: 9-May-18; **Record Level:** institutionCode: SMF; basisOfRecord: PreservedSpecimen
- **Type status:** Paratype. **Occurrence:** catalogNumber: SMF 380827; recordedBy: ROV ROPOS on R/V TAN KAH KEE; individualCount: 1; lifeStage: adult; occurrenceID: 7AB36057-98EC-5EEA-AE3B-ADFE5E4B24B5; **Taxon:** scientificName: *Ferreiraella charazata* Sigwart; kingdom: Animalia; phylum: Mollusca; class: Polyplacophora; order: Lepidopleurida; family: Abyssochitonidae; genus: Ferreiraella; specificEpithet: charazata; taxonRank: species; scientificNameAuthorship: Sigwart; nomenclaturalCode: ICZN; **Location:** higherGeography: Pacific Ocean; waterBody: Western Pacific Ocean; verbatimDepth: 3003 m; verbatimCoordinates: 21°18.3411N, 119°11.7281E; verbatimLatitude: 21°18.3411N; verbatimLongitude: 119°11.7281E; verbatimCoordinateSystem: degrees decimal minutes; **Identification:** identifiedBy: Julia D. Sigwart; dateIdentified: 2024; **Event:** samplingProtocol: remotely operated vehicle (ROV) ROPOS on R/V TAN KAH KEE; eventDate: 9-May-18; **Record Level:** institutionCode: SMF; basisOfRecord: PreservedSpecimen

#### Description

Holotype 18.0 × 8.5 mm, oval. Overall colour of valves light brown, frequently marked with scratches revealing white shell.

**Figure 10.**
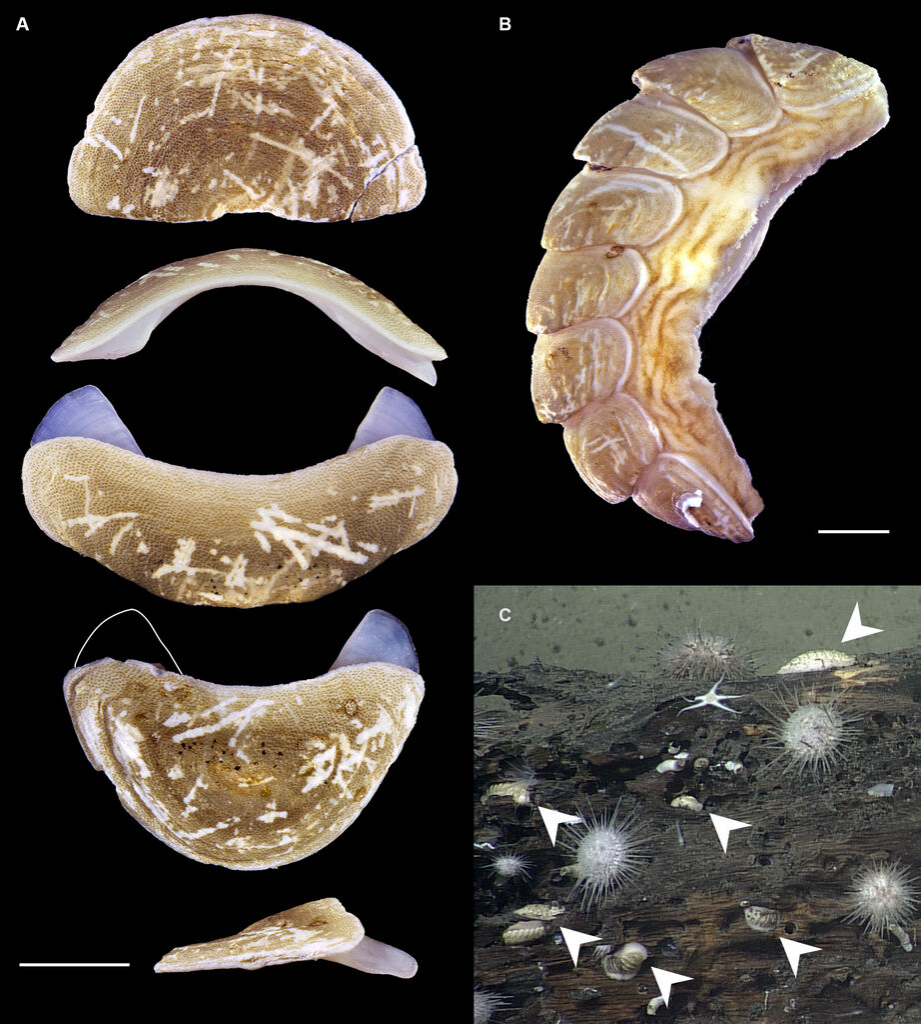
*Ferreiraella charazata* Sigwart, **sp. nov. A** Holotype SCSMBC240287, valves. From top to bottom: valve I, valve III (anterior view), valve V, valve VIII and valve VIII in lateral view; **B** Paratype 1 SMF 380825; **C** In situ photograph of multiple specimens (indicated with arrowheads) on sunken wood, together with other fauna. Scale bars: 2 mm.

**Figure 11.**
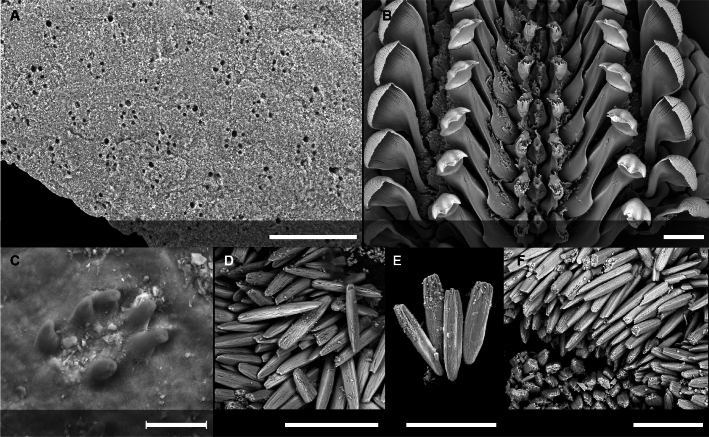
*Ferreiraella charazata* Sigwart, **sp. nov.**, holotype SCSMBC240287, SEM micrographs. **A** Aesthete pores, shown from valve I; **B** Radula; **C** Aesthete caps, shown from valve IV; **D–F** Girdle scales (perinotum). All scale bars: 100 μm (A, B, D–F), 25 μm (C).

Valves rounded, little elevated (dorsal elevation ratio 0.28 in valve III, Fig. 10A). Head valve semicircular. Intermediate valves slightly recurved, lateral margins rounded, anterior margin concave, posterior margin paralleling anterior margin, apex not projecting. Lateral areas not raised or visibly demarcated. Tail valve as wide as head valve, flattened, mucro central, posterior slope flat (Fig. 10A, B). Tegmentum smooth, covered with tissue and apical projections from aesthete pores. Aesthetes in bundles of one megalaesthete and approximately six micraesthetes with caps protruding from aesthete openings (Fig. 11A, C). Aesthete bundles typically in two parallel rows of three micraesthetes, but often irregularly arranged (sometimes 5 or 7 per megalaesthete).

Articulamentum white, well developed, without insertion plate or callus, apophyses moderately narrow, triangular in the intermediate valves and tail valve. Jugal sinus wide and convex.

Central tooth of radula small, bud-like, first (inner) lateral teeth with brush-like projections. Second (major) lateral teeth with flattened, tridentate mineralised cusps. Third uncinal (sweeper) teeth with extremely large and broad, spoon-shaped, but comb-like blade (Fig. 11B).

Girdle very wide, dorsally densely covered with oblong spicule-scales (up to 80–100 × 15–20 μm). Scales are proximally smooth, suboval in cross section, but include two types: distally tapering to a blunt point or distally stellate with four or six ribs. Ventral side of girdle naked.

Gills 11 on each side (paratype 3), smaller to the anterior, extending from valve VI to the anus.

##### Type material

Holotype (SCSMBC240287), now disarticulated, consisting of mounts of shell, perinotum and radula; three paratypes (Paratype 1: SMF 380825; Paratype 2: SMF 380826; Paratype 3: SMF 380827).

##### Material examined

Only known from the type material.

##### Type locality

Western Pacific Ocean, South China Sea, 21°18.3411N, 119°11.7281E, at 3003 m depth.

#### Diagnosis

Animal medium, holotype approximately 18.0 mm long (estimated from curled position). Overall colour brown, frequently marked with scratches revealing white shell. Shell rounded, valves not beaked, mucro of tail valve slightly anterior, tail valve flattened. Tegmentum smooth; aesthetes in bundles of one megalaesthete and six micraesthetes, with protruding caps. Girdle densely covered with oblong spicule-scales, flattened oval in profile. Scales include two types: distally bluntly pointed or stellate. Major lateral teeth of radula with relatively flattened, tridentate cusps. Sweeper teeth large, expanded, comb-like. Eleven gills on each side.

#### Etymology

From the Latin *charazo* meaning scratch or engrave, noting the common white scratch marks on the brown dorsal shells in this species (adjective, feminine).

#### Distribution

Only known from the type locality.

#### Taxon discussion

Sigwart and Sirenko (2012) provided a dichotomous key to all species of the genus *Ferreiraella*. The new species has a tail valve most similar to *F. plana*. In geographic range, *F. charazata* **sp. nov.** could be most comparable to *F. tsuchidai* Saito 2006, but the intermediate valves are oval in *F. tsuchidai* and cardioid in *F. charazata* **sp. nov.** The tail valve in *F. tsuchidai* is more oval with a straight postmucronal slope, round in *F. plana* and, in *F. charazata* **sp. nov.**, the tail valve is distinctly triangular and the postmucronal area very flat. Specimens of *F. charazata* **sp. nov.** and *F. tsuchidai* frequently host epibiotic tubeworms on their tail valves.

#### Methods

Individuals of *F. charazata* **sp. nov.** were collected in 2018 from sunken wood by remotely operated vehicle (ROV) ROPOS aboard R/V TAN KAH KEE, in the framework of the research programme “Deep Sea Process and Evolution of the South China Sea”.

Specimens were photographed using a motorised NIKON SMZ25 stereomicroscope, equipped with a NIKON Digital Sight 10 camera and stacked with NIKON NIS Elements Basic Research software (v. 5.42.04). Body size was measured from these photographs using ImageJ software (v.1.54g); to estimate body length in curled specimens, a curved line was fitted along the perinotum-plate boundary of lateral-view images and subsequently digitally straightened.

The holotype SCSMBC240287 was dissected to remove head, tail and representative intermediate valves, the radula and a piece of the perinotum for spicule preparation. Small cuts of the radula and girdle, as well as a fragment of the head valve were cleaned of soft tissue using diluted (max. 50%) household bleach for a few minutes, carefully rinsed with fresh water and transferred to aluminium SEM stubs with double-sided adhesive carbon tabs, either via air drying (valve fragment, spicules) or following dehydration through a graded ethanol series and subsequent chemical drying by means of hexamethyldisilazane (radula). SEM images were taken with a HITACHI TM4000Plus Tabletop scanning electron microscope with (chemically-dried radula) and without (other preparations) gold-palladium coating.

Spicules were measured using scales on SEM images and ImageJ. Images were processed with Adobe Photoshop 2024.

Specimen data for this description were gathered and processed via the Discovery Laboratory of the SENCKENBERG OCEAN SPECIES ALLIANCE.

**Repository**: Specimens are housed in the South China Sea Marine Biological Collections, Chinese Academy of Sciences, Guangzhou, China (SCSMBC) and the Senckenberg Research Institute and Natural History Museum Frankfurt (SMF).

### Pycnodontochiton

Sirenko, Zhang & Sigwart gen. nov.

AF69CE2C-B2E0-56BC-89E4-2EC594543E35

7CD8C680-1A43-4FFC-A9B9-DE7C8F6EF050

Pycnodontochiton sinensis Sirenko, Zhang & Sigwart. Type species.

#### Diagnosis

Chitons of small to medium size with body length up to 19 mm, elongate-oval, valves moderately elevated (dorsal elevation about 0.35). Head valve slightly narrower than tail valve. Valves V and VI widest. Tail valve with anterior mucro. Ratio of length of postmucronal area to length of antemucronal area 1.4. Tegmentum sculptured with small elongated granules closely arranged in longitudinal rows in central areas of intermediate valves and in antemucronal areas of tail valve and in quincunx patterns or in a random manner in other areas. Most granules with 1, 2 or 3 aesthete pores. Perinotum narrow, 5.5 times narrower than valve V, covered with bluntly pointed, slightly flattened, long spicules with 10–12 vague longitudinal ribs around spicule. Marginal needles and ventral spicules with vague longitudinal ribs or smooth. Radula ca. 5 mm long, with about 126 rows of mature teeth; central tooth large and unusual wide, with round dorsal edge, first lateral tooth narrow, roughly L-shaped, major lateral teeth large, with flat elongate heads, first uncinal tooth unusually narrow and elongated, major uncinal tooth long with well-developed blade. Number of gills in adult specimens 22.

#### Etymology

The genus name is a combination of the Greek roots *pycno*- (dense), *dont*- (tooth) and chiton, referring to the distinct dense tooth rows of the radula in this genus. The Greek word χιτών (chiton) is a masculine noun in the third declension.

#### Distribution

Deep-water chemosynthetic habitats from the East China Sea to the South China Sea.

#### Taxon discussion

The new genus includes two species: *Pycnodontochiton tenuidontus* (Saito and Okutani, 1990) **comb. nov.**, found in hydrothermal vent sites in the Okinawa Trough area and *P. sinensis* **gen. et sp. nov.**, from the Haima cold seep area in the South China Sea, at depths of 1385–1392 m.

Although new genus is superficially similar to *Leptochiton* as considered by Saito and Okutani (1990), it differs significantly from all known species in the teeth of the radula. Really no species of chitons, including species of the genus *Leptochiton*, have such huge central teeth and such long first uncinal tooth, which undoubtedly confirms the validity of the new genus *Pycnodontochiton* **gen. nov.**

### Pycnodontochiton sinensis

Sirenko, Zhang & Sigwart sp. nov.

2946159D-8C3D-5D28-A51B-62CA39A00167

C373B04B-70AF-49CA-BA87-AB0B18C988FC

OSD002-25

#### Materials

- **Type status:** Holotype. **Occurrence:** catalogNumber: MBM229047; individualCount: 1; lifeStage: adult; occurrenceID: 95C13B24-441F-5EBB-A332-74086077580A; **Taxon:** scientificName: *Pycnodontochiton sinensis* Sirenko, Zhang & Sigwart; kingdom: Animalia; phylum: Mollusca; class: Polyplacophora; order: Lepidopleurida; family: Leptochitonidae; genus: Pycnodontochiton; specificEpithet: sinensis; taxonRank: species; scientificNameAuthorship: Sirenko, Zhang & Sigwart; nomenclaturalCode: ICZN; **Location:** higherGeography: Western Pacific Ocean; locality: Haima cold seeps; verbatimDepth: 1392 m; verbatimCoordinates: 16°43′45″N, 110°28′23″E; verbatimLatitude: 16°43′45″N; verbatimLongitude: 110°28′23″E; verbatimCoordinateSystem: degrees minutes seconds; decimalLatitude: 16.729167; decimalLongitude: 110.473056; **Identification:** identifiedBy: Julia D. Sigwart, Boris Sirenko; dateIdentified: 2024; **Event:** eventID: H707TV6-3; eventDate: 7/7/2022; year: 2022; month: 7; day: 7; **Record Level:** institutionCode: MBM; basisOfRecord: PreservedSpecimen
- **Type status:** Paratype. **Occurrence:** catalogNumber: MBM229048; individualCount: 1; lifeStage: adult; occurrenceID: 1E9EAE51-45BB-58EB-883F-EFB037FB1F82; **Taxon:** scientificName: *Pycnodontochiton sinensis* Sirenko, Zhang & Sigwart; kingdom: Animalia; phylum: Mollusca; class: Polyplacophora; order: Lepidopleurida; family: Leptochitonidae; genus: Pycnodontochiton; specificEpithet: sinensis; taxonRank: species; scientificNameAuthorship: Sirenko, Zhang & Sigwart; nomenclaturalCode: ICZN; **Location:** higherGeography: Western Pacific Ocean; locality: Haima cold seeps; verbatimDepth: 1392 m; verbatimCoordinates: 16°43′45″N, 110°28′23″E; verbatimLatitude: 16°43′45″N; verbatimLongitude: 110°28′23″E; verbatimCoordinateSystem: degrees minutes seconds; decimalLatitude: 16.729167; decimalLongitude: 110.473056; **Identification:** identifiedBy: Julia D. Sigwart, Boris Sirenko; dateIdentified: 2024; **Event:** eventID: H707TV6-3; eventDate: 7/7/2022; year: 2022; month: 7; day: 7; **Record Level:** institutionCode: MBM; basisOfRecord: PreservedSpecimen
- **Type status:** Paratype. **Occurrence:** catalogNumber: SMF 380828; individualCount: 1; lifeStage: adult; previousIdentifications: *Leptochiton* sp.; occurrenceID: 7B64F365-BF4E-50FB-8D77-D7620E06B757; **Taxon:** scientificName: *Pycnodontochiton sinensis* Sirenko, Zhang & Sigwart; kingdom: Animalia; phylum: Mollusca; class: Polyplacophora; order: Lepidopleurida; family: Leptochitonidae; genus: Pycnodontochiton; specificEpithet: sinensis; taxonRank: species; scientificNameAuthorship: Sirenko, Zhang & Sigwart; nomenclaturalCode: ICZN; **Location:** higherGeography: Western Pacific Ocean; locality: Haima cold seeps; verbatimDepth: 1385 m; verbatimCoordinates: 16°43.937'N, 110°27.681'E; verbatimLatitude: 16°43.937'N; verbatimLongitude: 110°27.681'E; verbatimCoordinateSystem: degrees decimal minutes; decimalLatitude: 16.732283; decimalLongitude: 110.46135; **Identification:** identifiedBy: Julia D. Sigwart, Boris Sirenko; dateIdentified: 2024; **Event:** samplingProtocol: suction sampler on ROV HAIMA, on board R/V HAIYANG 6 cruise HYLH201902; eventDate: 5/5/2019; year: 2019; month: 5; day: 5; **Record Level:** institutionCode: SMF; basisOfRecord: PreservedSpecimen
- **Type status:** Paratype. **Occurrence:** catalogNumber: SCSMBC240288; individualCount: 1; lifeStage: adult; previousIdentifications: *Leptochiton* sp.; occurrenceID: F00A3CBA-7173-5494-A5FF-6B22B79BE5ED; **Taxon:** scientificName: *Pycnodontochiton sinensis* Sirenko, Zhang & Sigwart; kingdom: Animalia; phylum: Mollusca; class: Polyplacophora; order: Lepidopleurida; family: Leptochitonidae; genus: Pycnodontochiton; specificEpithet: sinensis; taxonRank: species; scientificNameAuthorship: Sirenko, Zhang & Sigwart; nomenclaturalCode: ICZN; **Location:** higherGeography: Western Pacific Ocean; locality: Haima cold seeps; verbatimDepth: 1385 m; verbatimCoordinates: 16°43.937'N, 110°27.681'E; verbatimLatitude: 16°43.937'N; verbatimLongitude: 110°27.681'E; verbatimCoordinateSystem: degrees decimal minutes; decimalLatitude: 16.732283; decimalLongitude: 110.46135; **Identification:** identifiedBy: Julia D. Sigwart, Boris Sirenko; dateIdentified: 2024; **Event:** samplingProtocol: suction sampler on ROV HAIMA, on board R/V HAIYANG 6 cruise HYLH201902; eventDate: 5/5/2019; year: 2019; month: 5; day: 5; **Record Level:** institutionCode: SCSMBC; basisOfRecord: PreservedSpecimen

#### Description

Holotype (body length 17.0 mm) elongate oval, valves subcarinated, moderately elevated (dorsal elevation 0.35), not beaked, side slope slightly convex, apex damaged, tegmentum white in colour.

**Figure 12.**
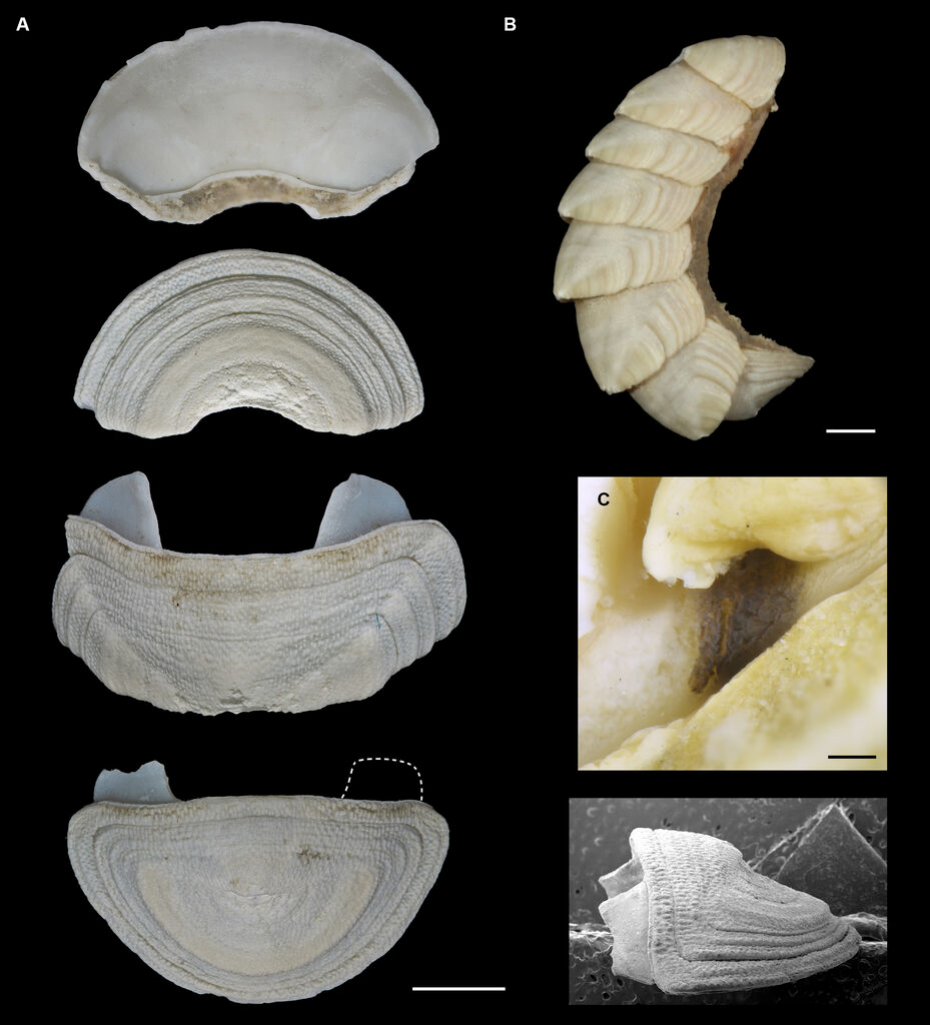
*Pycnodontochiton sinensis* Sirenko, Zhang & Sigwart, **sp. nov. A** Holotype MBM229047, valves. From top to bottom: valve I (ventral view), valve I (dorsal view), valve III, valve VIII; SEM image on lower right: valve VIII in lateral view; **B** Paratype 1 MBM229048, lateral view; **C** Paratype 3 SCSMBC240288, Schwabe organ (dark region, adjacent to mouth). Scale bars: 2 mm (A, B), 0.25 mm (C).

Head valve semicircular, hind margin with notch; intermediate valves rectangular, valves V and VI widest, anterior margin convex in valve II and concave in other intermediate valves, posterior margin slightly convex, lateral area weakly raised; tail valve is 1.1 times wider than head valve, mucro anterior, postmucronal slope first concave then convex, antemucronal area convex (Fig. 12 A, B).

**Figure 13.**
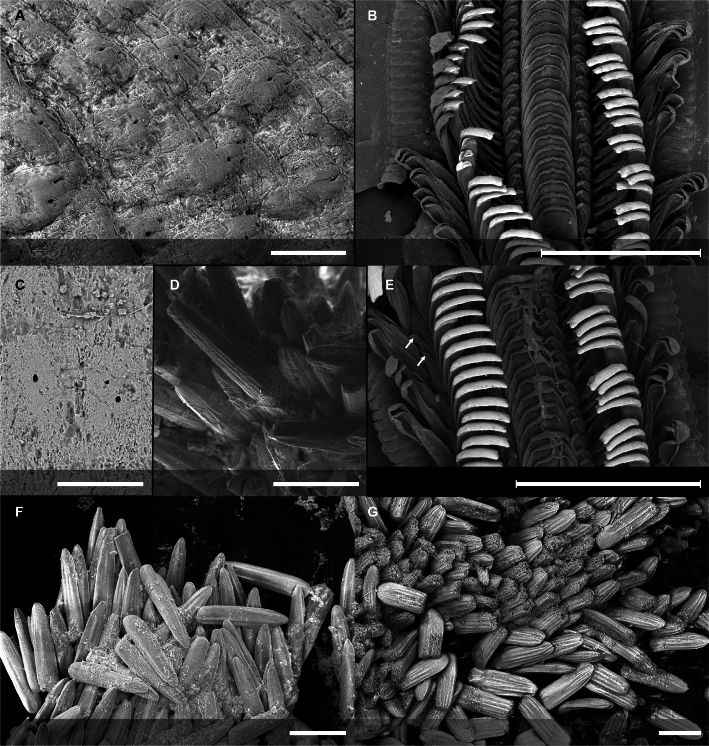
*Pycnodontochiton sinensis* Sirenko, Zhang & Sigwart, **sp. nov.**, SEM micrographs. **A** Paratype 2 SMF 380828, aesthete pores, shown from valve III; **B–E** Holotype MBM229047, radula (B), aesthete pores (C), girdle scales showing marginal scales (D), radula at a different position on the radular ribbon, showing the unusually large first uncinal (arrowheads; E); **F, G** Paratype 2 SMF 380828, ventral girdle scales (F), dorsal girdle scales (G). Scale bars: 100 μm (A, C, D, F, G), 500 μm (B, E).

Tegmentum sculptured with small elongated granules (160–200 x 60–70 µm) closely arranged in longitudinal rows in central areas of intermediate valves and in antemucronal areas of tail valve and in quincunx patterns or in a random manner in other areas. Each granule with one aesthete or sometimes two aesthetes, irregularly placed within low granules (Fig. 13A, C). Head valve, lateral areas of intermediate valves and postmucronal area of tail valve with 5–6 concentric terraced growth rings.

Articulamentum well developed, white, apophyses small, widely separated from each other, more or less triangular in intermediate valves or trapezoidal in tail valve. Ratio of width of jugal sinus to width of apophyses 1.3.

Girdle at valve V, 5.5 times narrower than valve width, dorsally covered with bluntly pointed, slightly flattened, long spicules (160 x 50 µm) with 10–12 vague longitudinal ribs around spicule. Marginal needles (200–210 µm) and ventral spicules (100–110 x 20–30 µm) also with vague longitudinal ribs, slightly square in cross section (Fig. 13D, F, G).

Holotype with gills extending from valve V to near anus. Paratype 1 with 19 gills on each side (actual number 17, estimated 19 due to defect), paratype 2 with 22 gills.

Radula 5 mm long, with 126 transverse rows of mature teeth, central tooth large and unusually wide (over 130 µm), with round dorsal edge; first lateral tooth narrow, roughly L-shaped, with a small blade; major lateral teeth large, with bidentate heads; first uncinal tooth unusually narrowly elongated, major uncinal tooth long with well-developed blade (Fig. 13B, E).

Width of valves (holotype): I-8.0 mm, II-9.2 mm, III-10.0 mm, IV-10.9 mm, V-11 mm, VI-11.0 mm, VII-10.2 mm, VIII-8.9 mm. Schwabe organ large, dark brown-black (Fig. 12C).

##### Type material

Holotype (MBM229047), now disarticulated, consisting of SEM stub of valves I, II, V, VIII, part of perinotum and radula, mount of part of perinotum and radula and vial with other valves. Paratype 1 (MBM229048) now disarticulated and the parts of the dorsal spicules, marginal spicules and aesthetes are provided. Paratype 2 (SMF 380828), now partially disarticulated, consisting of ethanol-preserved body, air-dried dissected valves I, II, III, VIII, radula preparations (in ethanol + dry on SEM stub) and SEM stub with bleached fragment of valve III and perinotal spicule preparation. Paratype 3 (SCSMBC240288), partially disarticulated.

##### Material examined

Only known from the type material.

##### Type locality

Western Pacific Ocean, Haima cold seeps, in the western part of the South China Sea. Collected at two sites: 16°43′45″N, 110°28′23″E, depth 1392 m and 16°43.937'N, 110°27.681'E, depth 1385 m.

#### Diagnosis

As for genus.

#### Etymology

Named for its occurrence off the coast of China.

#### Distribution

Only known from the type locality.

#### Taxon discussion

As already noted, *P. sinensis* **gen. et sp. nov.** is closely related to the congener *P. tenuidontus*. *Pycnodontochiton sinensis* **gen. et sp. nov.** differs from *P. tenuidontus* in having an even wider central tooth of radula and without its narrowing in distal part and having bidentate heads of the major lateral teeth and also by its ribbed marginal needles and by ribbed ventral spiculae. The new species is distinguished from all other species of chitons by the remarkably long and flat central tooth of radula and an unusually long first uncinal tooth.

The radula of *P. sinensis* **gen. et sp. nov.** is its most distinctive feature. *Pycnodontochiton tenuidontus*, from hydrothermal areas, has a similar radula that was described as unique because of its flat, overlapping teeth (Saito and Okutani 1990), similar to *P. sinensis* **gen. et sp. nov.** and both species have indistinct granular sculpture. The radula of *P. tenuidontus* also has a prominent, wide and round central tooth and a distinctive inner small lateral (between the major lateral and sweeper teeth), two features that are absent in *P. sinensis* **gen. et sp. nov.** *Pycnodontochiton tenuidontus* has three aesthetes in a bundle on each granule, but *P. sinensis* **gen. et sp. nov.** has one or two aesthetes per granule. The valves in *P. tenuidontus* are also round-backed, compared to subcarinate in *P. sinensis* **gen. et sp. nov.** Two other species with densely-packed, flat-headed major lateral teeth in the radula are *Leptochiton kerguelensis* (Haddon, 1886), from Antarctic and sub-Antarctic regions and *Leptochiton ferreirai* Sirenko & Sellanes, 2016 from deep water near Chile. Both those species are much smaller in body size than *Pycnodontochiton* spp. and live in distant regions and other features of the radula differ. The radula of *L. kerguelensis* has large, broad sweeper teeth not like the elongate first uncinal seen in *P. sinensis* **gen. et sp. nov.** The major lateral teeth in the radula of *L. ferreirai* are distinctly tricuspid and pointed, not square and bicuspid as in *P. sinensis* **gen. et sp. nov.**

#### Methods

Paratypes SMF 380828 and SCSMBC240288 were prepared as described for *Ferreiraella charazata* Sigwart, **nov. sp.**, but all preprations for SEM imaging were air dried directly on the stubs and observed without metal coating.

For DNA barcoding, a small fragment of tissue from the foot of paratype 3 was sampled and amplification and sequencing were performed using the primer sets LCO1490/HCO2198 (Folmer et al. 1994).

Specimen data for this description were (in parts) gathered and processed via the Discovery Laboratory of the SENCKENBERG OCEAN SPECIES ALLIANCE.

**Repository**: Specimens are housed in the Marine Biological Museum, Chinese Academy of Sciences, Qingdao, China (MBM), the South China Sea Marine Biological Collections, Chinese Academy of Sciences, Guangzhou, China (SCSMBC) and the Senckenberg Research Institute and Natural History Museum Frankfurt (SMF).

### Veleropilina gretchenae

Sigwart & Steger sp. nov.

9B789CA4-735E-5339-A5C4-766EAFF39354

EF4C7C97-2A14-4827-A458-93BDFA8C636D

OSD001-25

Veleropilina cf. oligotropha (Rokop, 1972): Brandt (2022): 157, 165, Fig. 5.96, tab. 5.26
Veleropilina sp. Sigwart et al. (2025): table 1, fig. 4B
Veleropilina sp. Chen et al. (2025): fig. 1, text

#### Materials

- **Type status:** Holotype. **Occurrence:** catalogNumber: SMF 373808; recordedBy: R/V SONNE AleutBio cruise SO293; individualCount: 1; lifeStage: adult; previousIdentifications: Veleroplina cf. oligotropha | *Veleropilina* sp.; associatedReferences: Brandt, A. (2022) (ed.) SO293 AleutBio (Aleutian Trench Biodiversity Studies). SONNE-Berichte (R/V SONNE cruise reports). 209 pp.; occurrenceID: A8D84ADE-70F5-50BB-8363-96C3208E1D3D; **Taxon:** scientificName: *Veleropilina gretchenae* Sigwart & Steger; kingdom: Animalia; phylum: Mollusca; class: Monoplacophora; order: Neopilinida; family: Neopilinidae; genus: Veleropilina; specificEpithet: gretchenae; taxonRank: species; scientificNameAuthorship: Sigwart & Steger; nomenclaturalCode: ICZN; **Location:** higherGeography: Pacific Ocean; waterBody: Northeast Pacific Ocean, U.S. Exclusive Economic Zone, Alaska Region; locality: Aleutian Trench, S of Unalaska Island, AleutBio station SO293_9-16 AGT; verbatimDepth: 6465-6465 m; minimumDepthInMeters: 6465; maximumDepthInMeters: 6465; verbatimCoordinates: 51°55.94'N, 166°51.84'W to 51°55.94'N, 166°51.85'W; verbatimCoordinateSystem: degrees decimal minutes; **Identification:** identifiedBy: Julia D. Sigwart; **Event:** eventID: SO293_9-16 AGT; samplingProtocol: Agassiz trawl, contents sieved through a 1 mm mesh; samplingEffort: 990 m trawled distance; eventDate: 2022-08-18T05:41Z/2022-08-18T06:15Z; eventTime: 05:41Z/06:15Z; year: 2022; month: 8; day: 18; habitat: hadal sediment; fieldNumber: AB 3572 | MOL-0570; **Record Level:** language: en; institutionCode: SMF; basisOfRecord: PreservedSpecimen

#### Description

**Figure 14.**
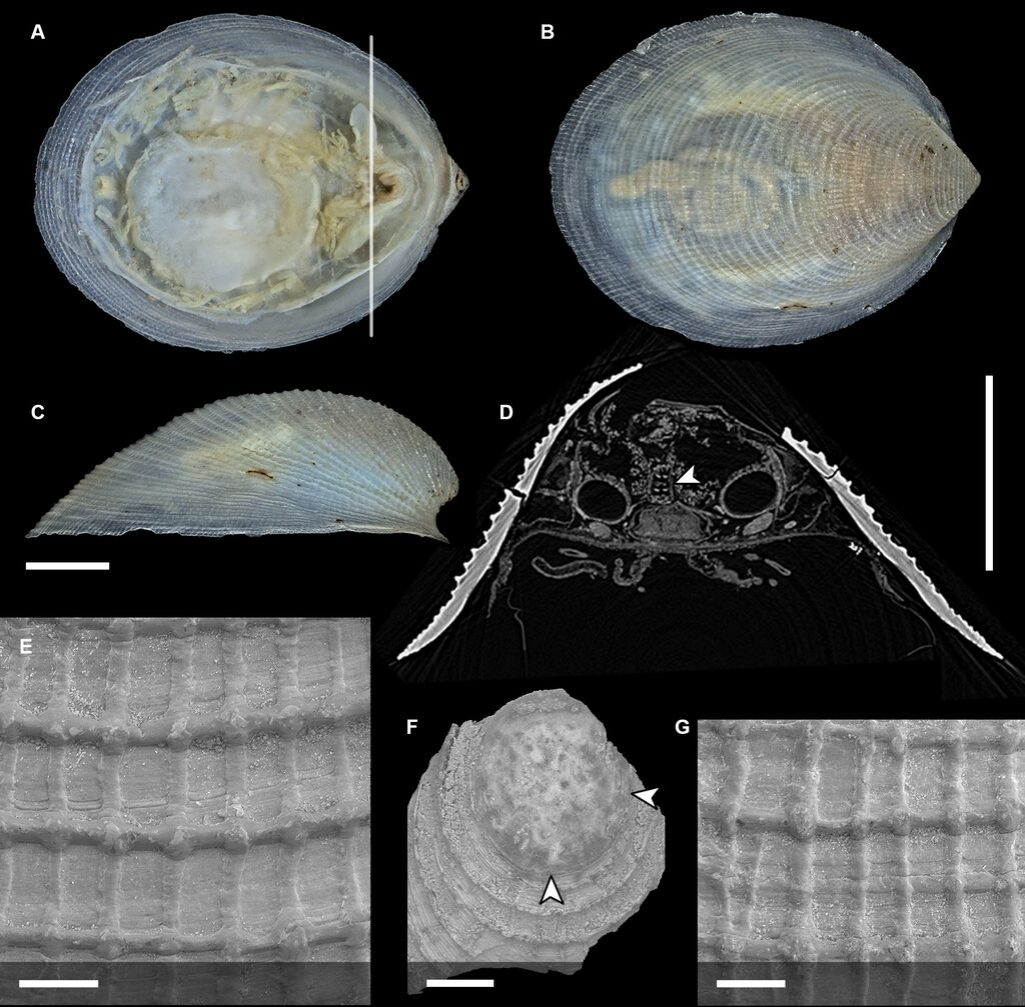
*Veleropilina gretchenae* Sigwart & Steger, **sp. nov.**, holotype SMF 373808. **A** Ventral view (line indicates approximate location of x-ray virtual cross section shown in image D); **B** Dorsal view; **C** Lateral view (right side); **D** Virtual cross section from synchrotron x-ray micro-CT, with arrowhead indicating position of radula; **E** Detail of teleoconch sculpture; **F** Apical cap, with arrowheads indicating the position of concentric ridges marking the transition to the reticulate teleoconch; **G** Detail of teleoconch sculpture showing minor irregularity. Scale bars: 1 mm (C–D), 100 μm (E–G).

Animal relatively large for the genus (5.2 mm long, 4.0 mm wide, 2.0 mm high), width to length ratio 0.77, height to length ratio 0.38 (Fig. 14A–C). Shell fragile, pale, translucent, whitish. Aperture ovoid, evenly rounded posteriorly, slightly narrowing anteriorly. Apex protruding beyond anterior shell margin, but forming a regularly curved arc with dorsal teleoconch surface in lateral view. Apical cap approximately 280 μm long, 250 μm wide, positioned at an angle of slightly more than 100° relative to the ventral plane, eroded in the holotype, smooth except for six closely-spaced concentric ridges at transition to teleoconch (Fig. 14F, arrowheads). Shell surface ornamented with prominent reticulate sculpture comprised of numerous concentric ribs with irregular interspacing, but becoming slightly closer together distally. Radial ribs slightly thinner and less prominent than the concentric sculpture, spaced more closely at posterior, with broader interspacing at anterior. Rib intersections with weak nodes (Fig. 14E, G), delimiting rectangular interspaces of variable size.

Foot prominent, subcircular, surrounded by a wide, shallow pallial groove; five digitate gills per side, each approximately as long as the width of the pallial groove in the preserved specimen. Anterior velar lobes large, elongated, leaf-like, almost three times as long as the diameter of the anterior lip. Postoral tentacles well-developed. Radula docoglossan with one pair of prominent major teeth (visible in micro-CT; Fig. 14D); radula cartilages large. Eight dorso-ventral muscles per side, first pair (counting from anterior) immediately posterior to radula cartilages; second pair divided into two distinct bundles of inequal size (anterior part smaller). Gut with six loops.

##### Type material

Holotype (SMF 373808).

##### Material examined

Only known from the holotype.

##### Type locality

Northeast Pacific Ocean, Aleutian Trench, south of Unalaska Island, 51°55.94'N, 166°51.84'W to 51°55.94'N, 166°51.85'W, 6465 m depth.

#### Diagnosis

Large-sized *Veleropilina* (shell length > 5 mm); shell moderately elevated (height to length ratio 0.38), surface covered by prominent reticulate sculpture with almost equally thick radial and concentric ribs delimiting rectangular to squarish interspaces. Aperture ovoid, narrowing anteriorly. Apex protruding beyond anterior shell margin, forming a regularly curved arc with the dorsal teleoconch surface when viewed laterally. Apical cap large, approximately 280 μm long and 250 μm wide, positioned at an angle of slightly more than 100° relative to the ventral plane. Postoral tentacles and velar lobes well-developed, the latter more than three times longer than the diameter of the anterior lip. COI mitochondrial barcode region with 12.71% difference (87.29% BLAST similarity) to that recovered from the *V. oligotropha* (s.l.) genome of Chen et al. (2025) and 7.23% difference (92.77% similarity) to another previously published COI sequence of *V. oligotropha* (NCBI accession MF157522, Wiklund et al. (2017)) in a smaller region of sequence overlap.

#### Etymology

Named after Dr Gretchen Van Meer Sigwart, civil engineer and professor, in recognition for her pioneering accomplishments and advocacy for equality and support for women in science, LGBTQ rights and people with disabilities.

#### Distribution

Only known from the type locality.

#### Taxon discussion

The new species differs from other congeners in several respects. Previously published analyses of the genome of *Veleropilina gretchenae* **sp. nov.** (sequenced from the holotype, as *Veleropilina* sp.) and a specimen identified as *V. oligotropha* (Rokop, 1972) revealed significant differences with an estimated divergence time of 72 million years (Chen et al. 2025). This substantial separation sheds new light on the importance of apparently minor morphological differences. Amongst these, the most obvious are body size, apertural shape and lateral shell profile: *V. gretchenae* **sp. nov.** is considerably larger than *V. oligotropha* and has an aperture that narrows anteriorly; its apical cap is well-aligned with the dorsal teleconch surface located behind it, forming a smooth, regularly curved arc, whereas it represents a prominent, growth stage-independent discontinuity or bump in *V. oligotropha*. In size, proportions of the shell and dimensions of the apical cap, *V. gretchenae* **sp. nov.** is most similar to *V. zografi* (Dautzenberg & H. Fischer, 1896) from the North Atlantic Ocean, but differs in the sculpture (relatively broad and low-profile, very narrowly-spaced concentric ribs in *V. zografi*) as well as geography (Warén and Gofas 1996). The radula of *V. gretchenae* **sp. nov.** as visible in micro-CT appears to be structurally similar to *Veleropilina seisuimaruae* Kano et al., 2012, although this is not entirely clear from the available data; as the holotype of the former species was badly damaged due to tissue sampling for genomic analysis, we elected not to pursue further dissection. All previously described species in *Veleropilina*, except *V. oligotropha*, occur at much shallower depths (Kano et al. 2012). *Veleropilina gretchenae* **sp. nov.** can most usefully be compared to other species in the genus reported from the Pacific (Table 3), highlighting the distinctive characters of *V. gretchenae* **sp. nov.**: larger size, relatively tall shell and large apical cap.

#### Methods

The holotype of *V. gretchenae* **sp. nov.** was collected during the SO293 AleutBio expedition of German R/V SONNE in 2022, using a 3.5 m-wide Agassiz trawl with 10 mm cod end mesh size (OKTOPUS GmbH). Contents of the net were sieved on a 1 mm mesh and subsequently preserved in 96% ethanol (Brandt 2022).

Photographs of the intact specimen were taken on board, prior to tissue sampling for genome sequencing (methods described in Chen et al. (2025), Electronic Supplementary Materials).

To molecularly compare the new species with the morphologically similar congener *V. oligotropha* (Rokop, 1972), both species’ COI sequences were extracted from published genomic datasets (Chen et al. (2025), with *V. gretchenae* **sp. nov.** reported as *V.* sp.); a further COI sequence of *V. oligotropha* from the Clarion-Clipperton Zone was downloaded from GenBank (accession no. MF157522; Wiklund et al. (2017)). Sequence similarity between *V. gretchenae* **sp. nov.** was assessed using NCBI BLAST (Camacho et al. 2009). The COI sequence isolated from the genome of *V. gretchenae* **sp. nov.** for the purpose of this study was uploaded to the Barcode of Life Data Systems (BOLD) (see Materials section for details).

Shell microsculpture and apical cap morphology was investigated and documented, based on shell fragments (produced by invasive tissue sampling) after air drying them, using a HITACHI TM4000Plus Tabletop scanning electron microscope (SEM) without metal coating.

Soft body anatomy was studied from a micro-computed tomography (micro-CT or µCT) scan performed at the ANATOMIX beamline, synchrotron SOLEIL, Paris, following the methods and settings described in Ampuero and Sigwart (in press). Prior to CT scanning, the specimen was incubated in a contrasting solution of 0.3% phosphotungstic acid and 3% dimethyl sulphoxide in 95% ethanol (Senckenberg Ocean Species Alliance (SOSA) et al. 2024) for ca. 1 week. The x-ray virtual section shown was produced with Dragonfly software (v. 2022.2; OBJECT RESEARCH SYSTEMS).

Shell measurements (length, width, height) were taken from habitus images of the intact specimen using NIKON NIS Elements Basic Research software (v. 5.42.04). Apical cap size was determined from SEM images using TM4000 software (HITACHI Ltd, Tokyo, Japan).

Image processing and figure plate assembly was performed in Adobe Photoshop 2025.

Specimen data for this description were gathered and processed via the Discovery Laboratory of the SENCKENBERG OCEAN SPECIES ALLIANCE.

**Repository**: The holotype is housed at the Senckenberg Research Institute and Natural History Museum Frankfurt (SMF).

### Laevidentalium wiesei

Sahlmann, 2012

D3A9D79C-3516-576D-8D0A-7450600C4FA2

https://www.marinespecies.org/aphia.php?p=taxdetails&id=716154

OSD004-25

OSD003-25

OSD005-25

Laevidentalium wiesei n. spec.: Sahlmann (2012): 26, Plate 6, figs. 1–3; 27, Plate 7, figs. 1a, 2a–b, 3a.
Dentaliida sp. M: Brandt (2022): 157, 158, 166, Tab. 5.26, fig. 5.96; Sigwart et al. (2025): fig. 4L.
“deep-sea scaphopod-anemone association”: Linse and Neuhaus (2024): 14.
Laevidentalium wiesei : Sigwart et al. (2025): tab. 1.

#### Materials

- **Type status:** Holotype. **Occurrence:** catalogNumber: HNC 43947; individualCount: 1; associatedReferences: Sahlmann, B. (2012) Description of a new abyssal scaphopod, *Laevidentalium wiesei*, from the Kurile-Kamchatka Trench (Mollusca, Scaphopoda). Schriften zur Malakozoologie aus dem Haus der Natur – Cismar, 27, 25–28.; occurrenceID: 975F8F10-C2E4-5DEF-9143-CD8AFEBD9439; **Taxon:** scientificNameID: urn:lsid:marinespecies.org:taxname:716154; scientificName: *Laevidentalium wiesei* Sahlmann, 2012; kingdom: Animalia; phylum: Mollusca; class: Scaphopoda; order: Dentaliida; family: Laevidentaliidae; genus: Laevidentalium; specificEpithet: wiesei; taxonRank: species; scientificNameAuthorship: Sahlmann; nomenclaturalCode: ICZN; **Location:** higherGeography: Pacific Ocean; waterBody: Northwest Pacific Ocean, Russian Exclusive Economic Zone, Kuril-Kamchatka Trench area; locality: Kuril-Kamchatka Trench area; verbatimLocality: RUS/Kurile-Kamchatka Trench; verbatimDepth: 5035-5210 m; minimumDepthInMeters: 5035; maximumDepthInMeters: 5210; verbatimCoordinates: 45°N, 156°E; verbatimLatitude: 45°N; verbatimLongitude: 156°E; verbatimCoordinateSystem: degrees; decimalLatitude: 45; decimalLongitude: 156; **Identification:** identifiedBy: Bernd Sahlmann; **Record Level:** language: en; institutionCode: HNC; basisOfRecord: PreservedSpecimen
- **Type status:** Paratype. **Occurrence:** catalogNumber: HNC 82015; individualCount: 1; associatedReferences: Sahlmann B (2012) Description of a new abyssal scaphopod, *Laevidentalium wiesei*, from the Kurile-Kamchatka Trench (Mollusca, Scaphopoda). Schriften zur Malakozoologie aus dem Haus der Natur – Cismar, 27, 25–28.; occurrenceID: 930E2C3E-17E1-58BF-807C-D87C0727FBEC; **Taxon:** scientificNameID: urn:lsid:marinespecies.org:taxname:716154; scientificName: *Laevidentalium wiesei* Sahlmann, 2012; kingdom: Animalia; phylum: Mollusca; class: Scaphopoda; order: Dentaliida; family: Laevidentaliidae; genus: Laevidentalium; specificEpithet: wiesei; taxonRank: species; scientificNameAuthorship: Sahlmann; nomenclaturalCode: ICZN; **Location:** higherGeography: Pacific Ocean; waterBody: Northwest Pacific Ocean, Kuril-Kamchatka Trench area; locality: Kuril-Kamchatka Trench area; verbatimLocality: SU/Kurile-Kamchatka Trench; verbatimDepth: 4500 m; minimumDepthInMeters: 4500; maximumDepthInMeters: 4500; verbatimCoordinates: 41°N, 156°E; verbatimLatitude: 41°N; verbatimLongitude: 156°E; verbatimCoordinateSystem: degrees; decimalLatitude: 41; decimalLongitude: 156; **Identification:** identifiedBy: Bernd Sahlmann; **Event:** habitat: abyssal/hadal; **Record Level:** language: en; institutionCode: HNC; basisOfRecord: PreservedSpecimen
- **Type status:** Other material. **Occurrence:** catalogNumber: SMF 366425; recordNumber: AB-4794; recordedBy: R/V SONNE AleutBio cruise SO293; individualCount: 1; preparations: whole animal (EtOH); previousIdentifications: Dentaliida sp. M; associatedReferences: Brandt, A. (2022) (ed.) SO293 AleutBio (Aleutian Trench Biodiversity Studies). SONNE-Berichte (R/V SONNE cruise reports). 209 pp.; associatedSequences: https://portal.boldsystems.org/record/OSD004-25; occurrenceID: 89137760-8030-5134-B7E4-808F729A9F05; **Taxon:** scientificNameID: urn:lsid:marinespecies.org:taxname:716154; scientificName: *Laevidentalium wiesei* Sahlmann, 2012; kingdom: Animalia; phylum: Mollusca; class: Scaphopoda; order: Dentaliida; family: Laevidentaliidae; genus: Laevidentalium; specificEpithet: wiesei; taxonRank: species; scientificNameAuthorship: Sahlmann; nomenclaturalCode: ICZN; **Location:** higherGeography: Pacific Ocean; waterBody: Northeast Pacific Ocean, U.S. Exclusive Economic Zone, Alaska Region; locality: Aleutian Trench, AleutBio/SO293 cruise, AleutBio station SO293_14-10 AGT; verbatimDepth: 4877 m; minimumDepthInMeters: 4877; maximumDepthInMeters: 4877; verbatimCoordinates: 52°40.90'N, 161°51.71'W to 52°40.90'N, 161°51.72'W; verbatimCoordinateSystem: degrees decimal minutes; **Identification:** identifiedBy: Jan Steger; dateIdentified: 2024; identificationReferences: Sahlmann, B. (2012) Description of a new abyssal scaphopod, *Laevidentalium wiesei*, from the Kurile-Kamchatka Trench (Mollusca, Scaphopoda). Schriften zur Malakozoologie aus dem Haus der Natur – Cismar, 27, 25–28.; **Event:** eventID: SO293_14-10 AGT; samplingProtocol: Agassiz trawl, contents sieved through a 1 mm mesh; samplingEffort: 1006 m trawled distance; eventDate: 2022-08-29T09:57Z/2022-08-29T10:30Z; eventTime: 09:57Z/10:30Z; year: 2022; month: 8; day: 29; habitat: abyssal sediment; fieldNumber: AB-4794 | MOL-1000; **Record Level:** language: en; institutionCode: SMF; basisOfRecord: PreservedSpecimen
- **Type status:** Other material. **Occurrence:** catalogNumber: SMF 366426; recordNumber: AB-4794; recordedBy: R/V SONNE AleutBio cruise SO293; individualCount: 1; preparations: whole animal (EtOH); previousIdentifications: Dentaliida sp. M; associatedReferences: Brandt, A. (2022) (ed.) SO293 AleutBio (Aleutian Trench Biodiversity Studies). SONNE-Berichte (R/V SONNE cruise reports). 209 pp.; associatedSequences: https://portal.boldsystems.org/record/OSD005-25; occurrenceID: 66033612-F89F-5029-A941-F6C49979C583; **Taxon:** scientificNameID: urn:lsid:marinespecies.org:taxname:716154; scientificName: *Laevidentalium wiesei* Sahlmann, 2012; kingdom: Animalia; phylum: Mollusca; class: Scaphopoda; order: Dentaliida; family: Laevidentaliidae; genus: Laevidentalium; specificEpithet: wiesei; taxonRank: species; scientificNameAuthorship: Sahlmann; nomenclaturalCode: ICZN; **Location:** higherGeography: Pacific Ocean; waterBody: Northeast Pacific Ocean, U.S. Exclusive Economic Zone, Alaska Region; locality: Aleutian Trench, AleutBio/SO293 cruise, AleutBio station SO293_14-10 AGT; verbatimDepth: 4877 m; minimumDepthInMeters: 4877; maximumDepthInMeters: 4877; verbatimCoordinates: 52°40.90'N, 161°51.71'W to 52°40.90'N, 161°51.72'W; verbatimCoordinateSystem: degrees decimal minutes; **Identification:** identifiedBy: Jan Steger; dateIdentified: 2024; identificationReferences: Sahlmann, B. (2012) Description of a new abyssal scaphopod, *Laevidentalium wiesei*, from the Kurile-Kamchatka Trench (Mollusca, Scaphopoda). Schriften zur Malakozoologie aus dem Haus der Natur – Cismar, 27, 25–28.; **Event:** eventID: SO293_14-10 AGT; samplingProtocol: Agassiz trawl, contents sieved through a 1 mm mesh; samplingEffort: 1006 m trawled distance; eventDate: 2022-08-29T09:57Z/2022-08-29T10:30Z; eventTime: 09:57Z/10:30Z; year: 2022; month: 8; day: 29; habitat: abyssal sediment; fieldNumber: AB-4794 | MOL-1000; **Record Level:** language: en; institutionCode: SMF; basisOfRecord: PreservedSpecimen
- **Type status:** Other material. **Occurrence:** catalogNumber: SMF 366427; recordNumber: AB-4794; recordedBy: R/V SONNE AleutBio cruise SO293; individualCount: 2; preparations: empty shell (fragments, in EtOH and air-dried); previousIdentifications: Dentaliida sp. M; associatedReferences: Brandt, A. (2022) (ed.) SO293 AleutBio (Aleutian Trench Biodiversity Studies). SONNE-Berichte (R/V SONNE cruise reports). 209 pp.; occurrenceID: 37910B5D-DE34-51A0-90E4-BC1FB33E21D1; **Taxon:** scientificNameID: urn:lsid:marinespecies.org:taxname:716154; scientificName: *Laevidentalium wiesei* Sahlmann, 2012; kingdom: Animalia; phylum: Mollusca; class: Scaphopoda; order: Dentaliida; family: Laevidentaliidae; genus: Laevidentalium; specificEpithet: wiesei; taxonRank: species; scientificNameAuthorship: Sahlmann; nomenclaturalCode: ICZN; **Location:** higherGeography: Pacific Ocean; waterBody: Northeast Pacific Ocean, U.S. Exclusive Economic Zone, Alaska Region; locality: Aleutian Trench, AleutBio/SO293 cruise, AleutBio station SO293_14-10 AGT; verbatimDepth: 4877 m; minimumDepthInMeters: 4877; maximumDepthInMeters: 4877; verbatimCoordinates: 52°40.90'N, 161°51.71'W to 52°40.90'N, 161°51.72'W; verbatimCoordinateSystem: degrees decimal minutes; **Identification:** identifiedBy: Jan Steger; dateIdentified: 2024; identificationReferences: Sahlmann, B. (2012) Description of a new abyssal scaphopod, *Laevidentalium wiesei*, from the Kurile-Kamchatka Trench (Mollusca, Scaphopoda). Schriften zur Malakozoologie aus dem Haus der Natur – Cismar, 27, 25–28.; **Event:** eventID: SO293_14-10 AGT; samplingProtocol: Agassiz trawl, contents sieved through a 1 mm mesh; samplingEffort: 1006 m trawled distance; eventDate: 2022-08-29T09:57Z/2022-08-29T10:30Z; eventTime: 09:57Z/10:30Z; year: 2022; month: 8; day: 29; habitat: abyssal sediment; fieldNumber: AB-4794 | MOL-1000; **Record Level:** language: en; institutionCode: SMF; basisOfRecord: PreservedSpecimen
- **Type status:** Other material. **Occurrence:** catalogNumber: SMF 373200; recordNumber: AB-4794; recordedBy: R/V SONNE AleutBio cruise SO293; individualCount: 1; preparations: animal (partially dissected) and part of radula (EtOH) | metal-coated radula preparation on SEM stub (dry); previousIdentifications: Dentaliida sp. M; associatedReferences: Brandt, A. (2022) (ed.) SO293 AleutBio (Aleutian Trench Biodiversity Studies). SONNE-Berichte (R/V SONNE cruise reports). 209 pp.; associatedSequences: https://portal.boldsystems.org/record/OSD003-25; occurrenceID: 968328A7-7937-5320-9CE6-4757C0D36D10; **Taxon:** scientificNameID: urn:lsid:marinespecies.org:taxname:716154; scientificName: *Laevidentalium wiesei* Sahlmann, 2012; kingdom: Animalia; phylum: Mollusca; class: Scaphopoda; order: Dentaliida; family: Laevidentaliidae; genus: Laevidentalium; specificEpithet: wiesei; taxonRank: species; scientificNameAuthorship: Sahlmann; nomenclaturalCode: ICZN; **Location:** higherGeography: Pacific Ocean; waterBody: Northeast Pacific Ocean, U.S. Exclusive Economic Zone, Alaska Region; locality: Aleutian Trench, AleutBio/SO293 cruise, AleutBio station SO293_14-10 AGT; verbatimDepth: 4877 m; minimumDepthInMeters: 4877; maximumDepthInMeters: 4877; verbatimCoordinates: 52°40.90'N, 161°51.71'W to 52°40.90'N, 161°51.72'W; verbatimCoordinateSystem: degrees decimal minutes; **Identification:** identifiedBy: Jan Steger; dateIdentified: 2024; identificationReferences: Sahlmann, B. (2012) Description of a new abyssal scaphopod, *Laevidentalium wiesei*, from the Kurile-Kamchatka Trench (Mollusca, Scaphopoda). Schriften zur Malakozoologie aus dem Haus der Natur – Cismar, 27, 25–28.; **Event:** eventID: SO293_14-10 AGT; samplingProtocol: Agassiz trawl, contents sieved through a 1 mm mesh; samplingEffort: 1006 m trawled distance; eventDate: 2022-08-29T09:57Z/2022-08-29T10:30Z; eventTime: 09:57Z/10:30Z; year: 2022; month: 8; day: 29; habitat: abyssal sediment; fieldNumber: AB-4794 | MOL-1000; **Record Level:** language: en; institutionCode: SMF; basisOfRecord: PreservedSpecimen
- **Type status:** Other material. **Occurrence:** catalogNumber: SMF 374281; recordNumber: AB-4794; recordedBy: R/V SONNE AleutBio cruise SO293; individualCount: 3; previousIdentifications: Dentaliida sp. M; associatedReferences: Brandt, A. (2022) (ed.) SO293 AleutBio (Aleutian Trench Biodiversity Studies). SONNE-Berichte (R/V SONNE cruise reports). 209 pp.; occurrenceID: 654B832B-EE22-597E-9AD4-9F1876B7741B; **Taxon:** scientificNameID: urn:lsid:marinespecies.org:taxname:716154; scientificName: *Laevidentalium wiesei* Sahlmann, 2012; kingdom: Animalia; phylum: Mollusca; class: Scaphopoda; order: Dentaliida; family: Laevidentaliidae; genus: Laevidentalium; specificEpithet: wiesei; taxonRank: species; scientificNameAuthorship: Sahlmann; nomenclaturalCode: ICZN; **Location:** higherGeography: Pacific Ocean; waterBody: Northeast Pacific Ocean, U.S. Exclusive Economic Zone, Alaska Region; locality: Aleutian Trench, AleutBio/SO293 cruise, AleutBio station SO293_14-10 AGT; verbatimDepth: 4877 m; minimumDepthInMeters: 4877; maximumDepthInMeters: 4877; verbatimCoordinates: 52°40.90'N, 161°51.71'W to 52°40.90'N, 161°51.72'W; verbatimCoordinateSystem: degrees decimal minutes; **Identification:** identifiedBy: Jan Steger; dateIdentified: 2024; identificationReferences: Sahlmann, B. (2012) Description of a new abyssal scaphopod, *Laevidentalium wiesei*, from the Kurile-Kamchatka Trench (Mollusca, Scaphopoda). Schriften zur Malakozoologie aus dem Haus der Natur – Cismar, 27, 25–28.; **Event:** eventID: SO293_14-10 AGT; samplingProtocol: Agassiz trawl, contents sieved through a 1 mm mesh; samplingEffort: 1006 m trawled distance; eventDate: 2022-08-29T09:57Z/2022-08-29T10:30Z; eventTime: 09:57Z/10:30Z; year: 2022; month: 8; day: 29; habitat: abyssal sediment; fieldNumber: AB-4794 | MOL-1000; **Record Level:** language: en; institutionCode: SMF; basisOfRecord: PreservedSpecimen

#### Description

**Figure 15.**
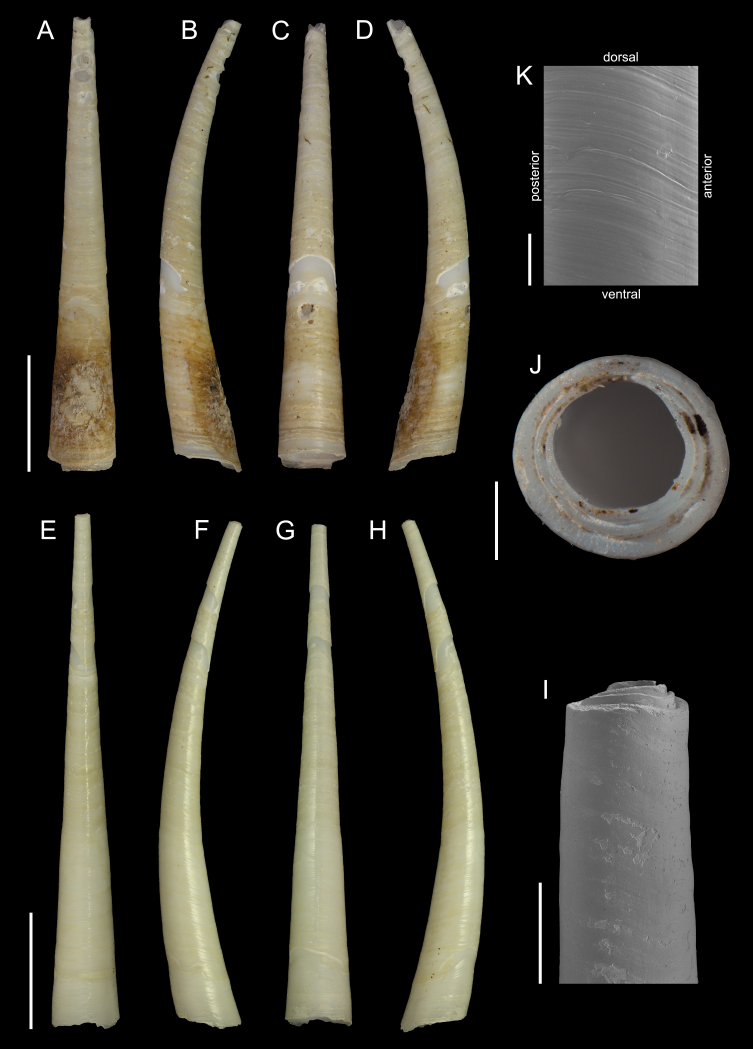
*Laevidentalium wiesei* Sahlmann, 2012, type material. **A–D** Holotype HNC 43947, anterior (A), right side (B), posterior (C) and left side (D) views. Note the dry remains of the epizoic anemone attached to the anterior face of the shell; **E–K** Paratype HNC 82015, anterior (E), right side (F), posterior (G) and left side (H) views; dorsal aperture in lateral (I; scanning electron micrograph) and apical (J) view; scanning electron micrograph showing the microsculpture consisting exclusively of densely arranged incremental lines (K). Scale bars: 10 mm (A–H), 1 mm (I, K), 0.5 mm (J).

**Figure 16.**
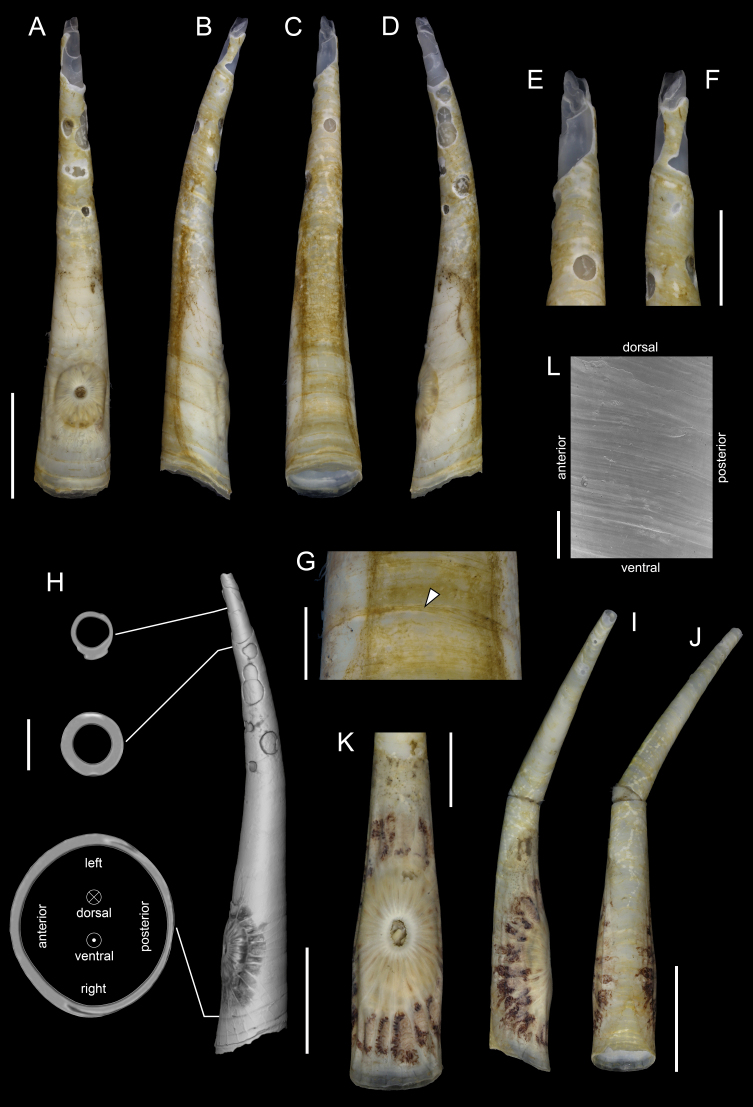
*Laevidentalium wiesei* Sahlmann, 2012, specimens from the AleutBio expedition. **A–H** Specimen SMF 366426, anterior (A), right side (B), posterior (C) and left side (D) views; close-ups of dorsal part of shell in posterior (E) and right side (F) view; detail of postero-ventral shell portion with growth mark indicated by arrowhead (G); micro-CT-based 3D reconstruction (left side view) and virtual cuts through the shell (perpendicular to the central axis) (H). Note the subcircular – wider than long – lumen of the ventral part of the shell that becomes circular dorsally; **I–K** Specimen SMF 373200 (shell broken during sampling event), right side (I) and posterior (J) views; close-up of the epizoic anemone attached to the anterior face of the shell (K); **L** Shell fragment from lot SMF 366427, scanning electron micrograph showing microsculpture. Scale bars: 10 mm (A–D, H – shell reconstruction, I–J), 5 mm (E–F, K), 2.5 mm (G), 2 mm (H – virtual shell sections), 1 mm (L).

**Figure 17.**
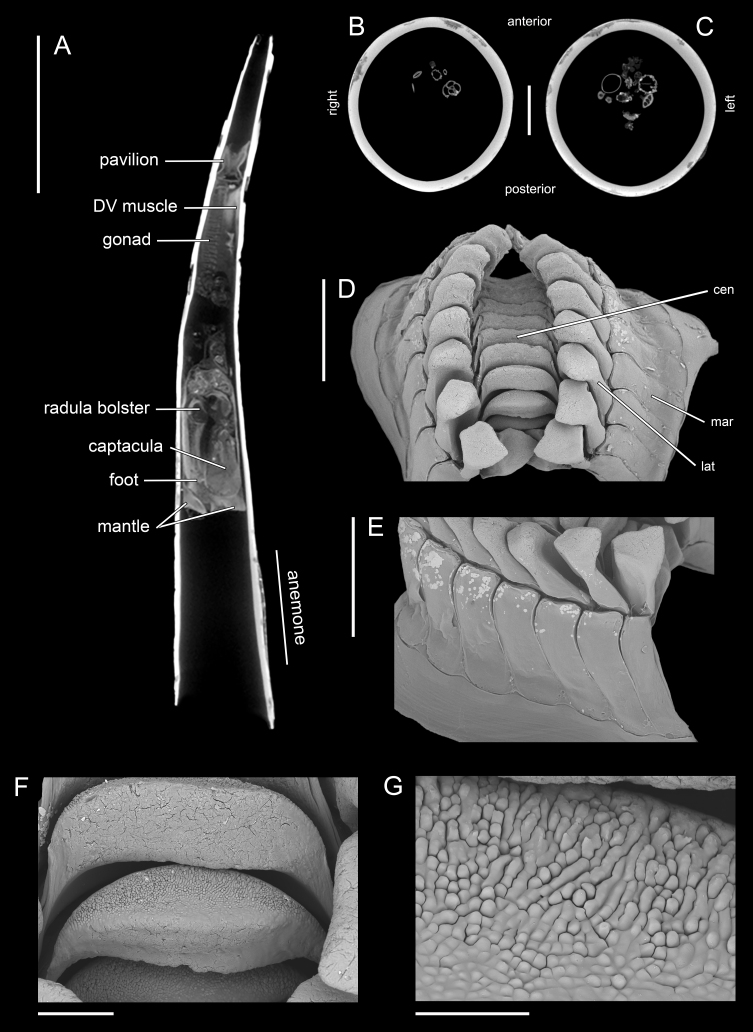
*Laevidentalium wiesei* Sahlmann, 2012, anatomy and radula. **A** Specimen SMF 366426, virtual cross section from x-ray tomography with major internal structures indicated. Abbreviations: DV muscle, dorso-ventral muscle; **B–G** Specimen SMF 373200, virtual cross sections from x-ray tomography showing tests of ingested foraminifera (B–C; scaphopod soft body not shown for clarity), overview of radula (D; Abbreviations: cen, central tooth; lat, lateral tooth; mar, marginal tooth), close-up of lateral and marginal teeth (E), of central tooth (F) and detail of granulose face of the latter (G). Scale bars: 10 mm (A), 1 mm (B–C), 0.5 mm (D–E), 0.1 mm (F), 0.03 mm (G).

Shell up to 45.6 mm long, maximum ventral aperture length 6.7 mm, ventral aperture width to 7.4 mm (holotype, Fig. 15A–D: 38.5 mm long, ventral aperture length 5.4 mm, ventral aperture width 5.9 mm), thin, but solid, tusk-shaped, gently recurved, with point of maximum curvature close to the mid-point of shell length in most of the specimens, located either dorsally or ventrally of it (Table 4). Outer surface glossy, ivory white to corneous in colour, ornamented with fine, densely arranged, irregular prosocline growth lines, without longitudinal (dorso-ventral) sculpture (Fig. 15I, K, Fig. 16L); growth interruptions are apparent at irregular intervals, but rather inconspicuous (Fig. 16G) and partly obscured by corrosion; intermediate shell layer white where exposed. Aperture subcircular, slightly wider than long (Table 4, Fig. 16H – bottom virtual section), edge prosocline, sharp. Dorsal (apical) end of the shell eroded in all specimens known, with the exposed intermediate and/or inner shell layers taking various shapes, either truncate (e.g. HNC 43946 – Sahlmann (2012)), shallow ring-like (e.g. HNC 82015, Fig. 15I–J) or more or less irregularly notched due to breakage along different sides (e.g. HNC 43947 – Fig. 15C, SMF 366426 – Fig. 16E–F); apical lumen circular in cross-section (Fig. 16H – middle virtual section). Protoconch morphology unknown. Inner shell surface white, smooth and glossy. Anterior (concave) side of shell often hosts an unidentified (and potentially undescribed) species of large-sized (up to 23 mm in preserved specimens) epibiotic sea anemone (Table 4, Fig. 15A–B, Fig. 15D, Fig. 16A, Fig. 16H–K); CT scans revealed no evidence of the anemone corroding or penetrating the scaphopod shell (Fig. 17A).

Gross anatomy (Fig. 17) follows typical dentaliid body plan (Fig. 17A); preserved body length of SMF 366426 (23.5 mm) around half the shell length (45.6 mm), gonad visible in CT scan is likely male. Captacular mass large and very dense. Dorsal pavilion relatively long (3 mm), robust.

Radula well-developed, with five teeth per row (formula 1-1-1-1-1; Fig. 17D); mature teeth brown in colour/mineralised, consistent with a foraminifera-dominated diet (Fig. 17B–C). Central teeth crescent-shaped, with convex superior face (eroding to concave in worn teeth) and adjacent granulose face (Fig. 17F–G); lateral teeth dumb-bell-shaped without cusps on working surface (Fig. 17D–E), marginals rather stout for the genus (cf. Glover et al. (2003) Lamprell and Healy (1998), Glover et al. (2003)), upright rectangular with rounded corners, basal part almost straight-sided, bent on quarter closest to lateral teeth (Fig. 17E).

##### Type material

Holotype (HNC 43947) and three paratypes (HNC 43946, HNC 82014, HNC 82015). Only dry-preserved shells were available for the original description, although their overall condition and glossy inner surface suggests they originated from live-collected specimens.

##### Material examined

Holotype HNC 43947 and paratype HNC 82015 (specimens examined and measured), plus new material from the Aleutian Trench (lots SMF 366425, 366426, 366427, 373200, 374281 – all collected together at a single station, see Materials for details).

##### Type locality

Kurile-Kamchakta [*sic*] Trench, 45°N, 156°E (Sahlmann 2012), collected at water depths ranging from 4500 m to 5210 m.

#### Diagnosis

Adult shell length to > 45 mm, ratio of shell length to ventral aperture length 7–9, gently recurved, without longitudinal keels, surface glossy, but often heavily corroded, smooth, except for dense prosocoline incremental lines; colour ivory white to corneous; ventral aperture subcircular, slightly wider than long, prosocline and sharp-edged. Anterior shell face usually hosting a single epibiotic sea anemone. Radula with crescent-shaped central teeth with granulose anterior face, lateral teeth without cusps, marginals rather stout for the genus, rectangular with rounded corners, basal part almost straight-sided, bent on quarter closest to lateral teeth.

#### Distribution

Known from the abyssal plain east of the Kuril Kamchatka Trench (type locality; Sahlmann (2012)) and the southern edge of the eastern Aleutian Trench (Sigwart et al. 2025, this study) at depths of 4500–5210 m. Sahlmann (2012) suspected that the records of “*Laevidentalium* sp.” reported by Okutani (1975), p. 76, from R/V SOYO-MARU Station 150 (Philippine Sea southeast of Kyushu, Japan, 29°52.4’N, 133°06.2’E, 3610 m water depth) and R/V KAIYO-MARU Station D (abyssal plain east of northern Honshu, Japan, 36°03.2’N, 157°51.4’E, 4370 m water depth) might correspond to this species; alternatively, these records may concern a closely-related species or species complex (see remarks in Okutani (1975)).

#### Taxon discussion

Two other *Laevidentalium* species have been recorded from abyssal depths in the Pacific Ocean – *L. largicrescens* (Tate, 1899) and *L. leptosceles* (R. B. Watson, 1879). Both can be distinguished from *L. wiesei* by shell and radula characters; furthermore, no *Laevidentalium* species other than *L. wiesei* is known to host an epibiotic sea anemone. *Laevidentalium largicrescens* is conchologically most similar, but differs from *L. wiesei* by the circular ventral aperture, the occasional presence of a posterior apical notch, cusps on the working surface of the lateral radular teeth and sigmoidal, much more elongated marginal teeth. It has a very different geographical range, occurring in the Southern Hemisphere, off the eastern Australian coast at 284–3058 m depth (Lamprell and Healy 1998). *Laevidentalium leptosceles* (R. B. Watson, 1879) is more slender, bears a weak, flexuous longitudinal sculpture in the apical part of the shell – lacking in the synonymised, shallower-water taxon *L. banale* (Boissevain, 1906) – and has a subcircular apical cross-section (Watson 1879, Lamprell and Healy 1998); it is widely distributed in the Indian Ocean and Western Pacific, with live records ranging from 2200–5300 m depth (Scarabino 1995).

The Atlantic abyssal *L. abyplainae* Scarabino & Scarabino, 2011, differs – besides its biogeography – by a straighter shell that expands less in diameter towards the ventral aperture, as well as by the presence of fine longitudinal striations on its apical part (Scarabino and Scarabino 2011, Sahlmann 2012). It shares with *L. wiesei* the granulose anterior face of the central radular teeth, but the lateral teeth are shaped differently and bear cusps (Scarabino and Scarabino 2011); the marginal teeth are relatively shorter than in *L. wiesei*.

The only large-sized scaphopod within the North Pacific recorded at abyssal depths and superficially resembling *L. wiesei* is *Rhabdus toyamaensis* (Kuroda & Kikuchi, 1933). Although it usually inhabits much shallower depths (Kuroda and Kikuchi 1933, Habe 1964, Habe 1977, Okutani 2000), a single specimen was collected at 5100 m in the Aleutian Trench, south of Attu Island (52°12’N, 175°44’E) (Sahlmann (2015), as *Rhadbus toyamaense* (Kuroda & Kikuchi, 1933)). *Rhabdus toyamaensis* attains much larger sizes than *L. wiesei* (up to 10 cm long), is more slender (the ventral aperture diameter of the Aleutian Trench specimen is just 4 mm at 61.5 mm shell length), has a completely circular ventral aperture and a shell sometimes bearing alternating light and dark bands. The abyssal specimen reported by Sahlmann (2015) – besides its unusual bathymetric and geographic location – also shows morphological differences from typical *R. toyamaensis*, such as subtle, but clearly visible annular swellings along the shell in addition to regular growth increments and an extremely glossy surface, suggesting it might actually be an undescribed species.

The most obvious feature of *L. wiesei* is the epizoic anemones attached to the anterior shell face of live individuals, an association previously reported only from members of the genus *Fissidentalium* (Dall 1908, Shimek and Moreno 1996, Shimek 1997, Linse and Neuhaus 2024). The shells of these species – *F. actiniophorum* Shimek, 1997, *F. aurae* Linse & Neuhaus, 2024, *F. megathyris* (Dall, 1890) and *F. peruvianum* (Dall, 1908) – however, all have distinct longitudinal ribs, which differentiate them at first sight from the smooth-shelled *L. wiesei*.

#### Notes

*Laevidentalium wiesei* Sahlmann, 2012 was originally described, based on four empty shells collected at abyssal depths in the Kuril Kamchatka Trench region, north-west Pacific Ocean. The type material, entirely housed in the malacological collection of Haus der Natur – Cismar (Germany), was obtained by the Museum in 1995 from private shell collections, accompanied by only basic locality data. Following its description (Sahlmann 2012), no further records of this poorly-known species were published until several, mostly live-collected, specimens were sampled in 2022 by the AleutBio expedition to the Aleutian Trench and adjacent abyssal parts of the Bering Sea, north-east Pacific Ocean (Brandt 2022, Sigwart et al. 2025). This new material enabled a more detailed, integrative re-description and constitutes a significant geographic range extension.

#### Methods

Specimens of *L. wiesei* from the SO293 AleutBio expedition were collected using a 3.5 m-wide Agassiz trawl with 10 mm cod end mesh size (OKTOPUS GmbH). Contents of the net were sieved on a 1 mm mesh and subsequently preserved in 96% ethanol (lots SMF 366425, SMF 366426, SMF 366427 and SMF 373200) or 4% buffered formalin-seawater (SMF 374281; Brandt (2022)).

Photographs of entire scaphopods were taken with a CANON EOS 6D camera, equipped with an EF 100 mm 1:2.8 IS USM macro lens and serial images stacked with Helicon Focus software (v. 5.3 X64; HELICON SOFT). Details of the outer shell surface were photographed using: (i) a motorised NIKON SMZ25 stereomicroscope with an attached NIKON Digital Sight 10 camera and (ii) a HITACHI TM4000Plus Tabletop scanning electron microscope (SEM) without metal coating. Stereo-microscopic images were stacked with NIKON NIS Elements Basic Research (BR) software (v. 5.42.04).

After initial photographic documentation, shell shape and soft body anatomy were studied by micro-computed tomography (micro-CT), using a WERTH TomoScope XS Plus CT scanner with the following settings [scan of SMF 373200/scan of SMF 366426]: acceleration voltage 60/80 kV, emission current 180/200 µA, exposure time 666 ms, voxel size 6.29/21.81 µm, number of images per revolution 2000/1000. Prior to CT scanning, specimens had been contrasted in a solution of 0.3% phosphotungstic acid and 3% dimethyl sulphoxide in 95% ethanol (Senckenberg Ocean Species Alliance (SOSA) et al. 2024) for 27 days. X-ray virtual sections were produced in Dragonfly software (v. 2022.2; OBJECT RESEARCH SYSTEMS). The 3D shell reconstruction of SMF 366426 and virtual shell sections were obtained by first cropping the raw tomographic dataset in Avizo3D (v. 2024.1; THERMO FISHER SCIENTIFIC) and postprocessing the resultant file in VGSTUDIO MAX (v. 2024.3; VOLUME GRAPHICS). The tomographic datasets and the surface model of SMF 366426 are available from Zenodo (https://doi.org/10.5281/zenodo.15470822).

Shell morphometric measurements follow Shimek and Moreno (1996) and were taken from macrophotographic images using NIS Elements BR software.

For radula preparation, the entire buccal mass of specimen SMF 373200 was dissected and the soft tissue dissolved in diluted commercial bleach (a 5% sodium hypochlorite solution). Following a ca. 10 s cleaning step in an ultrasonic bath (cf. Shimek and Moreno (1996)), the radula was dehydrated in absolute ethanol, transferred to hexamethyldisilazane (HMDS) through an ascending series of ethanol-HMDS mixtures and finally left to air-dry at room temperature in a fume hood overnight. The preparation was subsequently mounted on an aluminium stub with a double-sided adhesive tab, sputter-coated with gold-palladium and imaged using a HITACHI TM4000Plus Tabletop SEM.

Figure plates for this contribution were assembled with Adobe Photoshop 2025.

COI sequences of *L. wiesei* were obtained by the procedures detailed in Linse and Neuhaus (2024).

Specimen data for this description were gathered and processed via the Discovery Laboratory of the SENCKENBERG OCEAN SPECIES ALLIANCE.

**Repository**: Type material is held at Haus der Natur – Cismar (HNC), Cismar, Germany and additional specimens at the Senckenberg Research Institute and Natural History Museum Frankfurt (SMF).

### Myonera aleutiana

Machado & Sigwart sp. nov.

9185F898-A195-5350-9FE5-6249F956D275

D6F37247-7B9D-4C5F-87EF-D792478A0F32

#### Materials

- **Type status:** Holotype. **Occurrence:** catalogNumber: SMF 374321; recordNumber: AB4950D1; recordedBy: Die Expedition AleutBio (Aleutian Trench Biodiversity Studies); individualCount: 1; lifeStage: adult; preparations: whole animal (ETOH); occurrenceID: B2767C84-6115-5F0D-B11B-5A833BF687CF; **Taxon:** scientificName: *Myonera aleutiana* Machado & Sigwart; kingdom: Animalia; phylum: Mollusca; class: Bivalvia; order: Anomalodesmata; family: Cuspidariidae; genus: Myonera; specificEpithet: aleutiana; taxonRank: species; scientificNameAuthorship: Machado & Sigwart; nomenclaturalCode: ICZN; **Location:** higherGeography: Pacific Ocean; waterBody: North Pacific; islandGroup: Aleutian Islands, off Alaska, United States of America; locality: Aleutian Islands, off Alaska, United States of America; verbatimDepth: 5170 m; verbatimLatitude: 51°41.711'N; verbatimLongitude: 166°28.024'W; **Identification:** identifiedBy: Fabrizio Marcondes Machado; **Event:** samplingProtocol: box corer?; eventDate: 20/8/2022; habitat: abyssal sediment; **Record Level:** institutionCode: SMF; basisOfRecord: PreservedSpecimen
- **Type status:** Paratype. **Occurrence:** catalogNumber: SMF 373797; recordNumber: Mol558/AB3563; recordedBy: Die Expedition AleutBio (Aleutian Trench Biodiversity Studies); individualCount: 1; lifeStage: adult; preparations: whole animal (ETOH); occurrenceID: FFDD565F-A761-5896-8F1E-C8D888A8FFE6; **Taxon:** scientificName: *Myonera aleutiana* Machado & Sigwart; kingdom: Animalia; phylum: Mollusca; class: Bivalvia; order: Anomalodesmata; family: Cuspidariidae; genus: Myonera; specificEpithet: aleutiana; taxonRank: species; scientificNameAuthorship: Machado & Sigwart; nomenclaturalCode: ICZN; **Location:** higherGeography: Pacific Ocean; waterBody: North Pacific; islandGroup: Aleutian Islands, off Alaska, United States of America; locality: Aleutian Islands, off Alaska, United States of America; verbatimDepth: 5280 m; verbatimLatitude: 50°38.253'N; verbatimLongitude: 169°45.744'W; **Identification:** identifiedBy: Fabrizio Marcondes Machado; **Event:** samplingProtocol: box corer?; eventDate: 8/8/2022; habitat: abyssal sediment; **Record Level:** institutionCode: SMF; basisOfRecord: PreservedSpecimen

#### Description

**Holotype**

**Figure 18.**
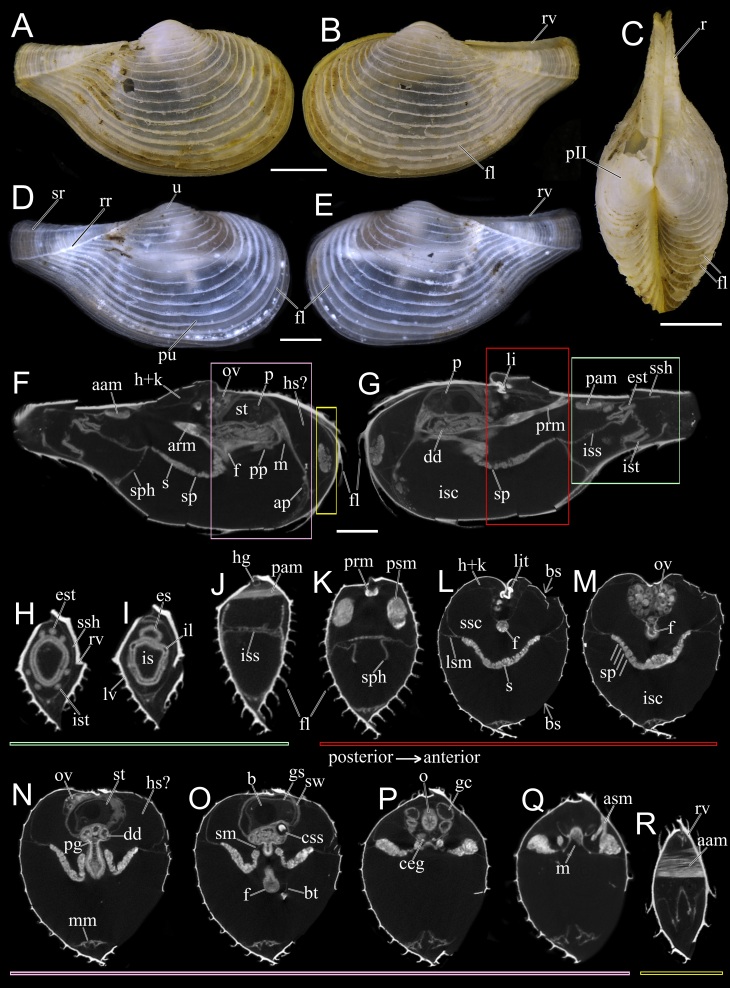
*Myonera aleutiana* Machado & Sigwart, **sp. nov.** shell and anatomy. **A–C** Photomicrography of holotype (SMF 373402), right and left valves plus a dorsal view, respectively; **D**–**R** Paratype (SMF 374320), right and left views, respectively; **F**–**R** Selected X-ray slices/images showing the arrangement and details of the pallial cavity organs and visceral mass; **F**–**G** Tomographic sagittal sections of different parts of the specimen; purple, yellow, red and green squares indicate the approximate areas of the transversal sections; **H**–**R** Tomographic transversal sections, from posterior to anterior orientation. Abbreviations: ap, anterior labial palp; aam, anterior adductor muscle; arm, anterior pedal retractor muscle; asm, anterior septal retractor muscle; b, bubble (artifact); bs, broken shell area; bt, byssal thread; ceg, cerebro-pleural (= circum-esophagic glanglia); css, crystalline style sac; dd, digestive diverticula; es, exhalant siphon; est, exhalant siphonal tentacles; f, foot; fl, foliaceus lamellae; gc, gastric caecum; gs, gastric shield; h + k, heart + kidney; hg, hind gut; hs?, haemocoel spaces; il, Inhalant lateral sinus; is, inhalant siphon; isc, infra-septal chamber; iss, inter-siphonal septum; ist, inhalant siphonal tentacles; lit, lithodesma; m, mouth; mm, mantle margin (ventral); o, oesophagus; ov, ovary; p, prey(s) inside stomach; pp, posterior labial palps; pu, pustules; pII, prodissoconch II; pam, posterior adductor muscle; prm, posterior pedal retractor muscle; r, rostrum; rr, radial ridge; rv, right valve; s, septum; sp, septal pores; sm, septal membrane; sr, secondary ridge; st, stomach; sph, sphincter of inhalant siphon; ssc, supra-septal chamber; ssh, siphonal sheath; u, umbones. Scale bars: 1.5 mm (A–C), 1 mm (D–R).

Dimensions: Length: 7.4 mm; Height: 4.1 mm; Width: 3.1 mm (Fig. 18A–C).

**Paratype**

Dimensions: Length: 6.8 mm; Height: 3.5 mm; Width: 2.5 mm (Fig. 18D–R). This individual presents a single and short byssal thread (bt), visible both through the shell's transparency and in tomographic images.

Shell small (up to 7.4 mm in length), extremely fragile, translucent to olive, inflated, inequilateral, elongate, rostrate, slightly inequivalve; posterior dorsal margin almost straight, anterior dorsal margin wider and rounded, dorsal and posteroventral margins of right valve (rv) overlapping left (lv) (Fig. 18A–E); umbones inflated and rounded, slightly prosogyrate; prodissoconch I barely visible, prodissoconch II (pII) (1040 ± 20 μm, n = 2) elliptical, smooth, well preserved in the two individuals analysed (Fig. 18C). External surface with 16-18 equidistant concentric foliaceous lamellae (fl), covering the entire shell, extending to the umbo (u) and complete to the rostral ridge (Fig. 18A–E); countless small pustules (pu) present between the lamellae. Prominent rostral ridge (rr) present, with a secondary ridge (sr) parallel to the posterior dorsal margin, usually visible (Fig. 18D). Hinge edentulous. Resilifer and lithodesma (lit) elongate, deflected, posteriorly pointed.

**Anatomy**:

Mantle margin with two fused points, anteriorly forming short pedal gape and posteriorly forming siphonal apertures; posteroventrally, mantle margin (mm) fusion (Fig. 18J-Q) probably involving inner and middle folds (Type B) (Yonge 1982); absence of fourth pallial aperture. No evidence of arenophilic glands (= radial mantle glands), likely due to the resolution limitations of the micro-CT scan, as these glands are commonly found in cuspidariids (Morton and Machado 2019).

Siphons separated, inter-siphonal septum (iss) present; inhalant (is) large and contracted (Fig. 18H), probably funnel-shaped; both surrounded by small finger-shaped tentacles at base, four in inhalant and three in exhalant siphon (es), encased in a thin and muscular siphonal sheath (ssh) (Fig. 18G-I). Inhalant lateral sinus present (il). Inhalant sphincter (sph) in the base of the inhalant siphon, which is dorsally attached to the posterior septal retractor muscle (prm) (Fig. 18F, K). The inhalant sphincter plays a crucial role in regulating water flow and the intake of prey into the infra-septal chamber (isc) and appears to be present exclusively in members of Cuspidariidae (Machado et al. 2016). Carnivorous bivalves with highly modified inhalant siphons, often regarded as active predators (e.g. *Allogramma formosa* (Jeffreys, 1882) or *Poromya granulata* (Nyst & Westendorp, 1839)), do not possess an inhalant sphincter.

Septum horizontal, pierced by four pores (sp) is present dividing the mantle cavity into infra (ventral/isc) and supraseptal (dorsal/ssc) chambers (Fig. 18F, G, L-O). It is suspended in the mantle cavity by posterior and bifurcated anterior septal retractor muscles (psm/asm), attached to the shell valves just above their respective adductor muscles and lie close to the pedal retractor muscles (prm/arm). Lateral septal retractor muscles (lsm) present being more concentrated posteriorly. Extra lateral septal muscle, absent. Left and right halves of the septum are united by the septal membrane (sm), which separates anteriorly to create the siphonal gape through which the foot can protrude (Fig. 18O).

Labial palps small and reduced, anterior and posterior palps present (Fig. 18F). Posterior palps (plp) flattened and located along the postero-lateral margins of the funnel-shaped mouth (m). Anterior palps (alp) greatly reduced and unattached to the posterior ventral surface of the anterior adductor muscle (aam). Unlike most Cuspidariidae, *M. aleutiana* **sp. nov.** does not have the anterior palp attached to the anterior adductor muscle, likely due to the effects of fixation (Yonge 1928, Machado et al. 2016).

Posterior and anterior adductor muscles present (pam/aam), isomyarian; with posterior and anterior pedal retractor muscle (prm/arm); posterior pedal and anterior septal retractor muscles (prm/asm) bifurcated and dorsally attach to the shell close to the posterior adductor muscle (Fig. 18G, Q).

Foot with small, unpronounced heel (Fig. 18F, L–O); presence of a single byssal thread (bt) (Fig. 18O). The presence of byssus threads in Anomalodesmata is rare, having been documented in only 14 species to date (Yonge 1952, Allen and Turner 1974, Morgan and Allen 1976, Krylova 1993, Leal 2008, Simone and Cunha 2008, Machado et al. 2016, Senckenberg Ocean Species Alliance (SOSA) et al. 2024), including *M. aleutiana* **sp. nov.** Amongst the Cuspidariidae, so far, only *Cardiomya cleryana* (A. d'Orbigny, 1846) has had its long and single byssus thread documented through illustrations and video recordings (Machado et al. 2016). The new species is, therefore, the first known representative of *Myonera* to exhibit this feature. The potential functions of the byssus thread in deep-sea carnivorous bivalves have never been tested, but it likely aids in anchoring within the sediment, as lie-in-wait predators require stability to effectively capture their prey. A small pebble can be observed attached to the distal portion of the byssus thread in the new species, reinforcing the anchorage hypothesis (Fig. 18O).

Digestive system with funnel-shaped mouth (m), opening into a short and muscular oesophagus (e) that enters into the anterodorsal portion of the stomach (s) (Fig. 18F, P, Q); stomach Type II (Purchon 1987, Purchon 1990) with a muscular wall (sw), well developed, rounded, with gastric shield (gs), prey inside (p) and associated with a crystalline style sac (css) not conjoined with the mid-gut; surrounded dorsally, anteriorly and laterally by female gonads (ov) and the digestive gland; digestive diverticula (dd) with large gastric caecum (gc) (Fig. 18F, G, N-P).

Pericardium/heart + kidney (h+k) area reduced and poorly visible (Fig. 18G, L). Haemocoel spaces (hs?) probably present in the anterior portion of the visceral mass (Fig. 18F, N).

Probably dioecious, ovary (ov) well visible (> 40 mature oocytes counted, ~ 100 μm in diameter); only mature oocytes observed i.e. larger and usually free in the ovarian lumen (Fig. 18F, M). Testis not found/visible. Although the measurements of prodissoconch I are unavailable, other evidence — such as the small body dimensions (miniaturisation) and the possibility that mature oocytes are encapsulated oocytes (post-fertilisation) — could support the hypothesis of brooding care in *Myonera aleutiana* **sp. nov.** The brooding care has already been described for other anomalodesmatans, such as members of the Spheniopsidae (Morton et al. 2016a, Morton et al. 2016b) and hypothesised for the verticordioideans *Trigonulina ornata* d'Orbigny, 1853 and *Lyonsiella illaesa* Machado & Sigwart, 2024 (Morton et al. 2019, Senckenberg Ocean Species Alliance (SOSA) et al. 2024).

Cerebro-pleural ganglia (ceg) present and visceral ganglia not visible. Pedal ganglia (pg) present, with no statocysts associated.

##### Type material

Holotype (SMF 374321), one whole individual with soft parts. Paratype (SMF 373797), one whole individual with soft parts.

##### Material examined

Only known from the type material.

##### Type locality

North Pacific Ocean, Aleutian Islands, off Alaska, United States of America, 51°41.711'N, 166°28.024'W, 5170 m depth.

#### Diagnosis

Shell translucent to olive, thin, fragile, inflated, rostrate; posterior dorsal margin almost straight; presence of two rostral ridges; dorsal and posteroventral margins of right valve overlapping left; outer surface with 16-18 equidistant concentric foliaceous lamellae covering the entire shell with countless small pustules present between them; anterior septal retractor muscle bifurcated; extra lateral septal muscles absent; reduced kidney and pericardial cavity; presence of a single and short byssus thread; probably dioecious.

#### Etymology

The specific epithet *aleutiana* refers to the species type locality, the Aleutian Islands.

#### Distribution

Known only from the Aleutian Islands, off Alaska, North Pacific. Bathymetric range: 5170–5280 m, a new record for the genus previously recorded at 4400 m depth in the Tasman Sea, Southwest Pacific (see Knudsen (1970)).

#### Taxon discussion

**Figure 19.**
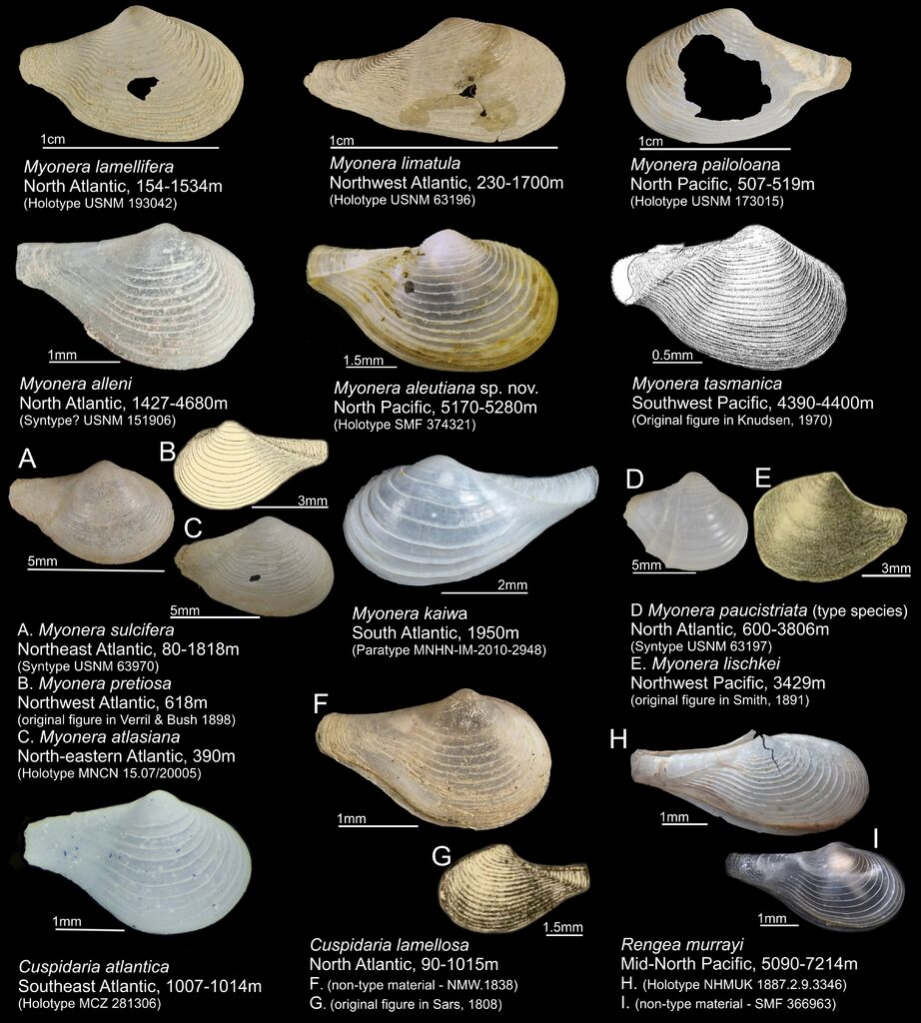
*Myonera aleutiana* Machado & Sigwart, **sp. nov.** comparison with other similar species from Atlantic and Pacific waters, including information on their occurrence, bathymetry, type numbers or original illustrations. External view of the shells of *Myonera lamellifera*, right view; *M. limatula*, right view; *M. pailoloana*, left view; *M. alleni*, right view (holotype not found); *M. aleutiana* **sp. nov.**, right view; *M. tasmanica*, right view (see Knudsen (1970): figs. 110–111); *M. sulcifera*, right view; *M. pretiosa*, left view; *M. atlasiana*, right view (see Utrilla et al. (2020): fig. 6); *M. kaiwa*, left view (after Oliveira and Absalão (2009)); *M. paucistriata*, right view; *M. lischkei*, left view; *Cuspidaria atlantica*, right view; *C. lamellosa*, right and left views, respectively and *Rengea murrayi*, right view. Abbreviations: MNHN, Muséum national d'Histoire naturelle, France; MNCN, Museo Nacional de Ciencias Naturales, Spain; MCZ, Museum of Comparative Zoology, US; NHMD, Natural History Museum of Denmark; NHMUK, Natural History Museum, London, UK; NMW, National Museum Wales, UK; SMF, Senckenberg Museum of Frankfurt, Germany; USNM, Smithsonian National Museum of Natural History, US.

The genus *Myonera* presents significant challenges in distinguishing between some of its species, as well as from certain species of *Cuspidaria* Nardo, 1840 and *Rengea* Kuroda & Habe, 1971, due to the overlap of anatomical and shell features. The new species was, therefore, compared with all morphologically similar species from these three genera through a detailed examination of the holotypes and/or original illustrations (Fig. 19). In general, *Myonera* and *Rengea* are distinguished by the presence of radial and concentric elements forming the outer sculpture in the former and only the presence of strong concentric plications in the latter. Additionally, *Rengea* have umbones depressed and more elongated shells. In *Cuspidaria*, the presence of a posterior lateral tooth in the right valve is the key feature that differentiates it from the other two genera – both edentulous.

This is the second time that a new species of bivalve has been described in detail (including shell and internal tissues) without the use of any invasive tool.

*Myonera aleutiana* new species differs from the other Pacific species, as *M. pailoloana* (Dall, Bartsch & Rehder, 1938), *M. tasmanica* (Knudsen, 1970) and *M. lischkei* (E. A. Smith, 1891) by the presence of fewer (16–18) and more spaced concentric lamellae, with fine pustules interspersed between them, a straighter rostrum and anterior and posterior dorsal margins of the right valve overlapping left. Furthermore, the new species possesses two rostral ridges and differences in shell outline (see Fig. 19). This same set of features was also used to differentiate *Myonera aleutiana* **sp. nov.** from the Atlantic congeners *Myonera lamellifera* (Dall, 1881), *M. limatula* (Dall, 1881), *M. sulcifera* (Jeffreys, 1882), *M. pretiosa* A. E. Verrill & K. J. Bush, 1898, *M. paucistriata* Dall, 1886 (type species), *M. alleni* Poutiers, 1995, *M. kaiwa* Oliveira & Absalão, 2009 and *M. atlasiana* Utrilla, Rueda & C. Salas, 2020. It is worth noting that the new species was thoroughly compared with *M. alleni*, not only through its probable syntype (USNM 151906), but also with the original drawings by Allen and Morgan (1981), figs. 35 and 36. Although the new species is edentulous, a comparison with some similar *Cuspidaria* species was necessary, since the fragility of the shells may prevent access to detailed information about the hinge teeth for future studies. In comparison with these non-congeneric species, besides the absence of teeth on the right and left valves, the new species exhibits a more elongated shell with a wider anterodorsal margin, larger, rounder and more developed umbones, as well as two clearly visible rostral ridges. Finally, *M. aleutiana* was also compared with *Rengea murrayi*, as both species are sympatric in Aleutian Islands and were initially misidentified as the same species during the preliminary screening of the material.

In general, little is known about the anatomy of *Myonera* species, except for some soft parts details of *M. alleni*, *M garreti* and *M. tasmanica* (Allen and Morgan 1981, Knudsen 1970). *Myonera aleutiana* **sp. nov.** is, therefore, the first species of the genus to be described in detail, using the combination of over 2,000 tomographic slices/images. Even so, features, such as a muscular septum perforated by four pairs of pores, presence of lateral septal muscles, reduced labial palps, configuration of 4 + 3 siphonal tentacles, presence of crystalline style sac and absence of projections on the inter-tentacular margin of the exhalant siphon (es), are not, in themselves, sufficient to distinguish the new species from other *Myonera* or from their confamilial relatives. It is worth noting, however, that the presence of an anterior septal retractor muscle bifurcated, a large number of eggs in the ovary (> 40), a significantly reduced kidney area and pericardial cavity and a single, short byssus thread have only been observed in *M. aleutiana* to date. Furthermore, the presence of a single byssus thread may also be debatable, as it could: (i) be broken during sampling procedures; (ii) dissolve in fixation solutions such as formalin and EtOH used in scientific collections or (iii) simply be ejected by the animal throughout its life. In addition to the shell and internal tissue characteristics described here, the new species also exhibits a distinct bathymetric range and geographic distribution compared to its congeners.

Finally, it is worth noting that the small individual (1.5 mm in length) collected by Wiklund et al. (2017) at 4082 m in the polymetallic nodule province of the eastern Pacific may represent juveniles of *M. aleutiana* or *Rengea murrayi*. Genetic data will be used in a future study by the authors to compare specimens from the AleutBio expedition with the available data at http://data.nhm.ac.uk/object/45033e06-fb54-49d5-b632-767e63c1cfd3 for the unique specimen archived in NHMUK under the number 20170037.

#### Methods

The two well-preserved specimens analysed here were collected in the eastern part of the Aleutian Trench, Alaska by the SO293 AleutBio expedition, using an epibenthic sledge (EBS). The new species individuals are part of a larger collection with more than 1,200 lots and 3,500 individuals of Mollusca collected between 2500 and 7500 m depth. Both were described using only non-invasive techniques/tools, such as photos by stereomicroscope (NIKON) and tomographic images using the WERTH micro-CT scanner TomoScope® XS Plus. Only the paratype was scanned; for this purpose, it was previously immersed in a contrast solution containing 0.3% phosphotungstic acid (PTA at a concentration of 99.995%), with 3% dimethyl sulphoxide (DMSO) in 95% ethanol by 10 days (adapted from Machado et al. (2018)). Scanning parameters were as follows: source voltage = 150 kV, current = 125 µA, exposure time = 666 ms, ignore images = 0, image quality = 7, voxel size = 3.56 µm, number of images per revolution = 2500, CTsensor = Kurzer_AAI_Size_10_XL, filter = no, drift correction = on, time of acquisition = 3h 15 min. The 2125 tomographic slices/images produced were reconstructed and analysed using the softwares WinWerth^®^ RAW Viewer, Viewer and 3D Viewer available in  https://www.werth.de/en/downloads.html. In addition, all the volumetric datasets are on-line available at the Harvard’s Dataverse under the link https://doi.org/10.7910/DVN/O2QQIM.

Specimen data for this description were (in parts) gathered via the Discovery Laboratory infrastructure of the SENCKENBERG OCEAN SPECIES ALLIANCE.

**Repository**: Type material is held at the Senckenberg Research Institute and Natural History Museum Frankfurt (SMF). In addition to the holotype and paratype (SMF), other museum lots were also analysed for comparison with the new species, most of them with images available on the websites of the respective institutions: Museum of Comparative Zoology - Harvard, (MCZ,  https://mczbase.mcz.harvard.edu/SpecimenSearch.cfm?collection_id=1), Smithsonian National Museum of Natural History (USNM, https://collections.nmnh.si.edu/search/iz/), Muséum National D’Histoire Naturelle (MNHN, https://science.mnhn.fr/institution/mnhn/collection/im/item/search).

### Apotectonia senckenbergae

Momtazi & Riehl sp. nov.

B52824AC-CB38-5D15-B8AC-8527CBEB7EA9

77B80C83-D50F-4D03-B6B7-08E95B33D824

https://www.marinespecies.org/aphia.php?p=taxdetails&id=488209

#### Materials

- **Type status:** Holotype. **Occurrence:** catalogNumber: SMF 62823; recordNumber: S0606-B06-CC15; recordedBy: R/V FALKOR cruise FKt231024; individualCount: 1; sex: male; lifeStage: adult; occurrenceID: C55370A3-BE0C-543E-9B7D-E935ECA4B48F; **Taxon:** scientificName: *Apotectonia senckenbergae* Momtazi & Riehl; kingdom: Animalia; phylum: Arthropoda; class: Malacostraca; order: Amphipoda; family: Alicellidae ; genus: Apotectonia; specificEpithet: senckenbergae; taxonRank: species; scientificNameAuthorship: Momtazi & Riehl; nomenclaturalCode: ICZN; **Location:** higherGeography: Pacific Ocean; locality: hydrothermal vent fields on the Galápagos Rift, near Rose Garden/Rosebud; verbatimDepth: 2601.6 m; verbatimLatitude: 0°46'16.3"N; verbatimLongitude: 85°55'25.0"W; decimalLatitude: 0.771193; decimalLongitude: -85.923598; **Identification:** identifiedBy: Farzaneh Momtazi; dateIdentified: 2024; **Event:** samplingProtocol: Bio Box mounted on ROV SUBASTIAN, dive #606; eventDate: 29/10/2023; habitat: mussel bed; fieldNumber: S0606-B06-CC15; **Record Level:** institutionCode: SMF; basisOfRecord: PreservedSpecimen

#### Description

**Description of holotype male**

**Figure 20.**
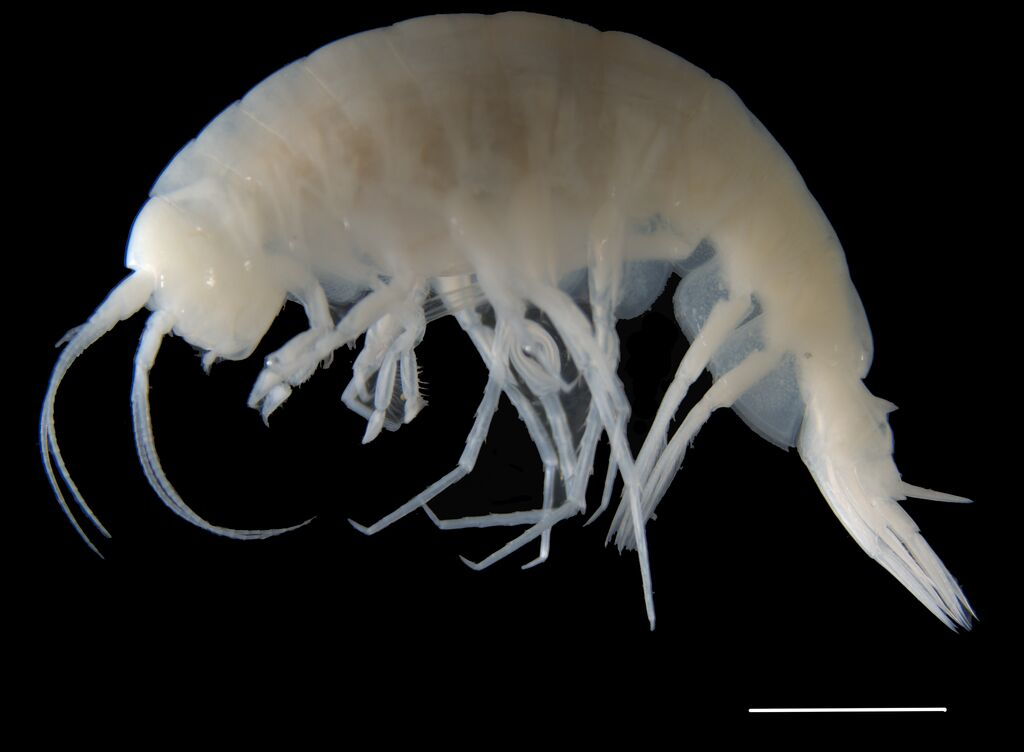
*Apotectonia senckenbergae* Momtazi & Riehl, **sp. nov.**, macro-photograph of male holotype (SMF-62823). Scale bar: 2.5 mm.

**Figure 21.**
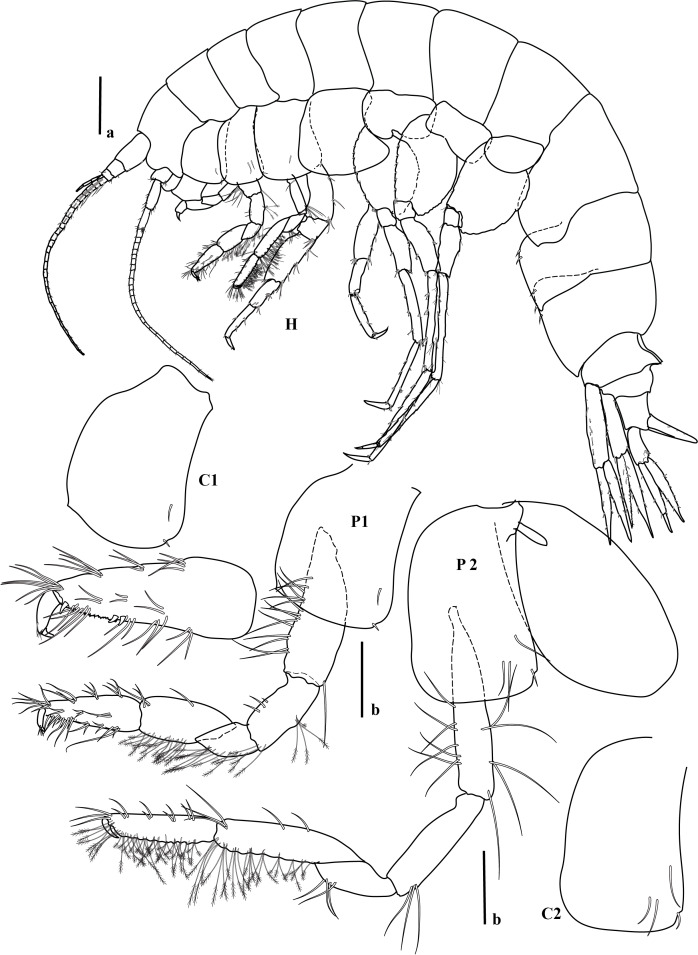
*Apotectonia senckenbergae* Momtazi & Riehl, **sp. nov.**, male holotype (SMF-62823) habitus (H), coxa 1-2 (C1, C2) and pereopods 1–2 (P1, P2). Scale bars: a = 2.5 mm, b = 0.5 mm.

**Figure 22.**
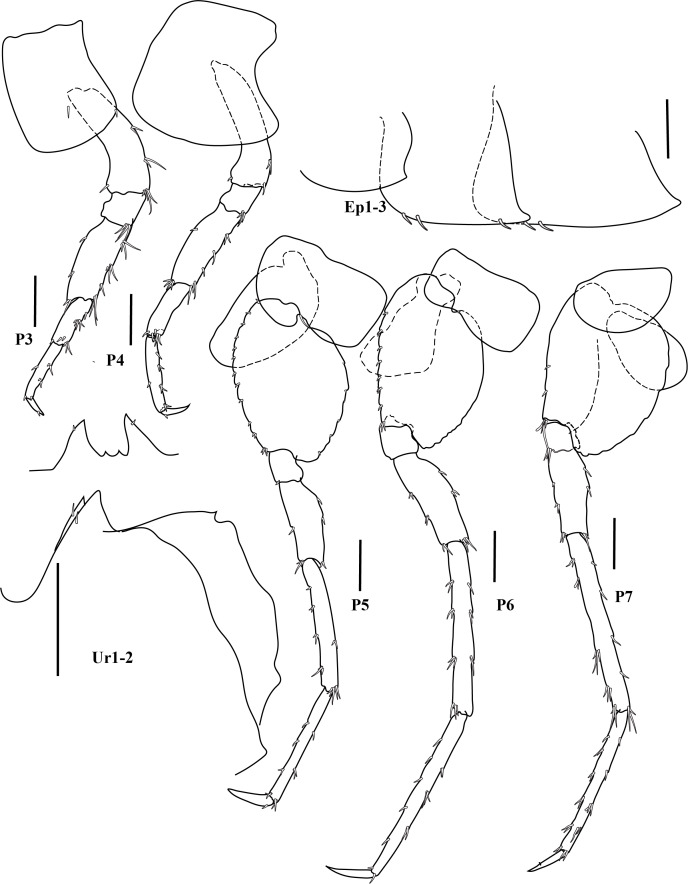
*Apotectonia senckenbergae* Momtazi & Riehl, **sp. nov.**, male holotype (SMF-62823) pereopods 4–7 (P4, P5, P6, P7), epimeral plates 1-3 (Ep 1-3) and urosomites 1-2 (Ur1-2). Scale bars: 0.5 mm.

Body length 15 mm (Figs 20, 21H). Urosomite 1 with dorsally sharp multifid carina (Fig. 22Ur); urosomite 3 dorsally with fine seta (Fig. 21H).

**Figure 23.**
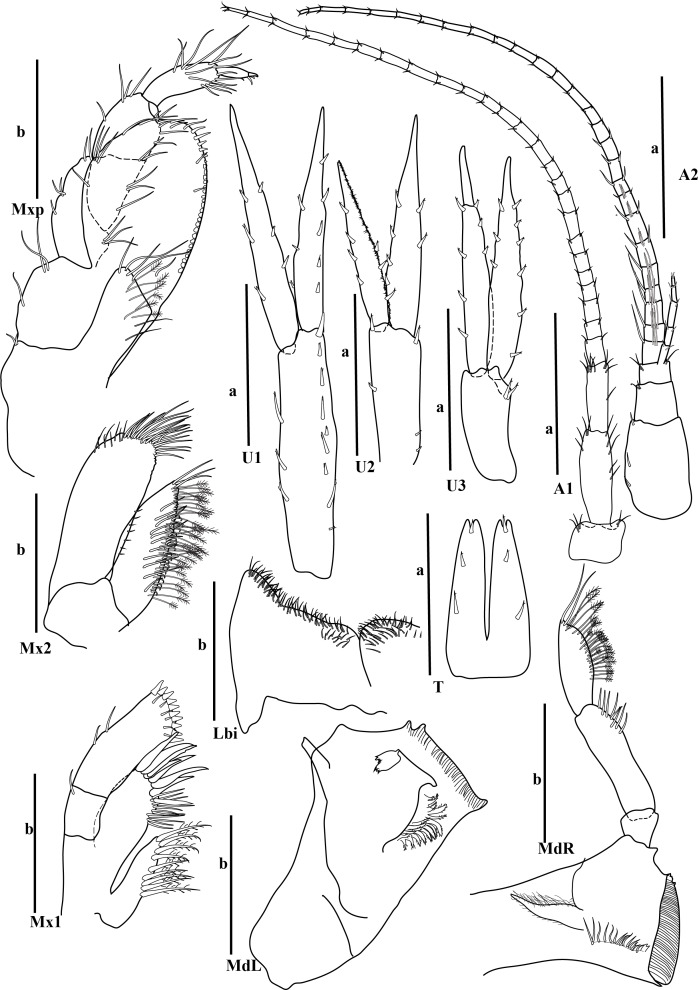
*Apotectonia senckenbergae* Momtazi & Riehl, **sp. nov.**, male holotype (SMF-62823) head appendages including left mandible (MdL), right mandible (MdR), first maxillae (Mx1), second maxillae (Mx2), maxilliped (Mxp), labium (Lbi), uropods 1–3 (U1, U2, U3), telson (T), antennae 1-2 (A1, A2). Scale bars: a = 1 mm, b = 0.5 mm.

**Head** deeper than long; rostrum absent. Eyes not apparent, but may be faded in ethanol. Lateral cephalic lobe well-developed (Fig. 21 H). **A1** (Fig. 23 A1), 0.31 body length; peduncular art1–3 exceed head length; acc flag 5-articulate; left primary flag 27-articulate, setal formula 5L, 2M, S, 3M, 16S (L: large; M: medium, S: small), right primary flag 25-articulate; aesthetascs on right flag art1–8. **A2** (Fig. 23 A2) slender, length 1.1 A1 length; peduncular art5 length 0.6 art4 length; flag 25-articulate; calceoli absent.

**Md** incisor not symmetrical, right incisor (Fig. 23 RMd) with an inner notch-tooth followed by a plate tooth and ending in three outer notch-teeth; the left incisor (Fig. 23 LMd) with an inner notch-tooth followed by a plate tooth and ending in two outer notch-teeth; right lacinia mobilis tiny, bifid, left lacinia mobilis slightly larger and multitoothed, rakers about 18, molars long, slender, pointed, pubescent; mandibular palps symmetrical, art3 blade-like, with two apical long simple, four long plumose and 19 medium plumose setae. **Mx1** (Fig. 23 Mx1) right outer plate with eight spines; inner plate narrow, with plumose setae on the outer margin; palp 2-articulate and well developed, symmetrical, terminal art with 10 apical robust setae, six subterminal and two lateral setae. **Mx2** (Fig. 23 Mx2) inner plate shorter than the outer plate; outer plate lateral margin with simple setae; inner plate with a row of simple setae, a row of plumose setae and downward-facing setae on the inner margin. **Mxp** (Fig. 23 Mxp) inner plate sub-rectangular, with four simple and six plumose apical setae; outer plate large, subovate; palp well-developed; dactylus well-developed, unguis present, surface with small setae.

**Prn** with unplaited gills each with a minor secondary sausage-shaped lobe on C2–7. **P1** (Fig. 21 P1), coxa bevelled anteroventrally, ventral margin produced posteriorly, anterior margin 0.75 posterior margin length, with one posteroventral and one superficial robust seta (Fig. 21 C1); basis L/W ratio 3.0, anterior margin covered with setae; ischium linear, length 0.5 basis length; merus trapezoid, posterior margin with plumose setae; carpus linear, subequal to propodus, posterior margin bearing plumose setae; propodus subtriangular, L/W ratio 2.8; palm straight, finely serrated, defined by pair of robust setae; dactylus short, not reaching to the middle of the palm. **P2** subchelate (Fig. 21 P2); C2 larger than C1, subsimilar in size to C3, quadrate, with one posteroventral and three superficial robust setae (Fig. 21 C2); basis L/W ratio 5.5; ischium linear, L/W ratio 3.5; merus trapezoid, shorter than carpus; carpus elongate, length 1.5 propodus length, anterior margin straight, posterior margin covered with plumose setae; propodus subrectangular, posterior margin with plumose seta; palm oblique; dactylus reaching to end of palm. **P3** similar to P4 (Fig. 22 P3), slightly stouter, C quadrate with a posteroventral and a superficial robust seta; dactylus simple, short. **P4** (Fig. 22 P4) C with well-developed posteroventral lobe; dactylus simple. **P5–6** with similar structures, P6 longer than P5 and P7 (Fig. 22 P5, P6, P7).

**Pleon** with **Ep1** (Fig. 22 Ep1–3) posterodistal corner produced to form sharp tooth; **Ep2** posterodistal corner with one small, rounded tooth, ventral margin with two robust setae; **Ep3** posterodistal corner with medium-sized sharp tooth, ventral margin with three robust setae. **U1** (Fig. 23 U1) length 1.36 U2 length, rami lengths subsimilar, subsimilar to peduncle size; **U2** (Fig. 23 U2) peduncle with three robust setae and two small robust setae; inner ramus length 1.7 peduncle length, inner ramus length 1.3 outer ramus length; **U3** (Fig. 23 U3) L/W ratio 2.25; rami subequal; inner ramus with robust setae; outer ramus biarticulate, Art2 asetose. **T** (Fig. 23 T) deeply cleft (75%), with two marginal setae in each half, apical margin truncate, each apical margin with one stout seta, each lobe distally notched.

##### Type material

Holotype adult male (SMF 62823), ethanol-preserved.

##### Material examined

Only known from the type material.

##### Type locality

Pacific Ocean, Galápagos Rift, hydrothermal vent fields on the near Rose Garden/Rosebud, 2601.6 m depth.

#### Diagnosis

The new species, *Apotectonia senckenbergae* **sp. nov.** is characterised by similar lengths of antennae 1 and 2, the epimeral setal formula 0+2+3, the coxal dorsoventral setal formula 1+1+1 and two medial robust setae on the posterior margin of the propodus of the first pereopod (gnathopod).

#### Etymology

This species’ epithet honours Johanna Rebecca Senckenberg (1716–1743) (maiden name: Riese) from Frankfurt am Main, Germany, who not only guided her husband Johann Christian Senckenberg (1707–1772) in spiritual and charitable matters, but also provided an inheritance that constituted nearly one-third of the funds establishing the Senckenberg Society for Nature Research. Through her much more well-known husband, she was a naturalist and benefactor who supported science and medicine, founding the Dr. Senckenberg Foundation, which later led to the forming of the Senckenberg Society for Nature Research.

#### Distribution

Only known from the type locality.

#### Ecology

Collected from a mussel bed in the vicinity of a hydrothermal vent.

#### Taxon discussion

The genus *Apotectonia* Barnard & Ingram, 1990 was described as a monotypic genus, based on specimens collected from deep-sea hydrothermal vents on the Galápagos Rift (Barnard and Ingram 1990). It is characterised by a simple first gnathopod, a multifid dorsal process on the first urosomite, a vestigial right lacinia mobilis, a bevelled inner plate of the maxilliped, an unreduced coxa 1 and a shortened outer ramus of the second uropod. The new species, *Apotectonia senckenbergae* **sp. nov.**, has also been collected from the Galápagos rift, but slightly deeper at 2,601m depth. The type species, *A. heterostegos* Barnard & Ingram, 1990, was collected at 2,488 m depth and described, based on the female specimen with 11.73 mm. However, they reported variations on the article number of the accessory flagellum and the setal formula on epimera and uropods in the juvenile specimens with 12.4 mm (Barnard and Ingram 1990). The differentiated characters of *A. senckenbergae* **sp. nov.** from *A. heterostegos* include subsimilar lengths of antennae 1 and 2 (instead of bearing a shorter antenna 1 in *A. heterostegos*), nine dental spines on the outer plate of the first maxilla (10 in *A. heterostegos*), the epimeral setal formula 0+2+3 (1+3+4 in *A. heterostegos*), 1+1+1 distoventral setae on coxae 1–3 (1+2+2 in *A. heterostegos*), the serrated inner margin and two robust setae on the outer ramus of the second uropod (asetose in *A. heterostegos*), two medial robust setae on the posterior margin of the propodus of the first pereopod (gnathopod) and other minute differences (see Table 5).

#### Notes

##### Methods

The material studied is part of a donation to the Senckenberg Research Institute and Natural History Museum Frankfurt, Germany from the Schmitt Ocean Institute R/V FALKOR (TOO) research cruise FKt231024 and the PROJECT ZOMBIE: BRINGING DEAD VENTS TO LIFE – ULTRA FINE-SCALE SEAFLOOR MAPPING. This project surveyed several hydrothermal vent fields on the Galápagos Rift using the remotely operated vehicle (ROV) SUBASTIAN. This cruise studied the vent fields Rose Garden/Rosebud (0.81°N, 86.22°W, 2450–2550 m depth), Tempus Fugit (0.77°N, 85.91–93°W, 2500–2560 m depth), Iguanas-Pinguinos (2.10°N, 91.89–94°W, 1650–1700 m depth) and Tortugas (0.95°N, 90.53–56°W, 1500–1600 m depth) (see Chen et al. (2024)). For animal sampling, a seven-function manipulator arm (Schilling Robotics TITAN 4) and a suction sampler mounted on ROV SUBASTIAN were used. Upon recovery onboard, animals were sorted in cold (4°C) seawater. The material used in the present study was collected with a suction sampler from a mussel bed at Tempus Fugit.

At the Senckenberg Laboratory, sample dissection was made in glycerol under a stereomicroscope (LEICA M60). Preliminary drawings were done with a microscope (LEICA DM 2500 LED), equipped with a camera lucida. Illustrations (Figs 21, 22, 23) were made using the methods described by Coleman (2009) using the software Adobe Illustrator CS6. Measurements were taken from the tip of the first antenna along the dorsal body line until the end of the telson and are given in millimetres.

The Discovery Laboratory of the SENCKENBERG OCEAN SPECIES ALLIANCE provided essential facilitation, technical support and logistical assistance that enabled this taxonomic work.

**Repository**: The holotype was deposited in the crustacean collection of the Senckenberg Research Institute and Natural History Museum Frankfurt, Germany, with the registration number SMF 62823.

### Metharpinia hirsuta

Souza-Filho & Andrade sp. nov.

E251FD10-CB18-5709-B541-23FB782C866F

07BCB3A5-BD40-4B7E-BCB2-2B79592B3D04

#### Materials

- **Type status:** Holotype. **Occurrence:** catalogNumber: MOUFPE 22050; recordedBy: Jesser F. Souza-Filho | Luiz F. Andrade; individualCount: 1; sex: female; lifeStage: adult; preparations: whole animal (ETOH); previousIdentifications: *Metharpinia* sp.; occurrenceID: 13EB2920-2820-528E-A68C-CB624E72E4AF; **Taxon:** scientificName: *Metharpinia hirsuta* Souza-Filho & Andrade; kingdom: Animalia; phylum: Arthropoda; class: Malacostraca; order: Amphipoda; family: Phoxocephalidae; genus: Metharpinia; specificEpithet: hirsuta; taxonRank: species; scientificNameAuthorship: Souza-Filho & Andrade; nomenclaturalCode: ICZN; **Location:** locationID: http://marineregions.org/mrgid/32405; higherGeography: Atlantic Ocean; waterBody: Potiguar Basin; country: Brazil; countryCode: BR; stateProvince: Ceará; locality: Potiguar Basin, station 075-CES23; verbatimDepth: 34.4 m; verbatimLatitude: 3°08.6399'S; verbatimLongitude: 38°51.9827'W; verbatimCoordinateSystem: degrees decimal minutes; decimalLatitude: -3.144; decimalLongitude: -38.8664; **Identification:** identifiedBy: Jesser F. Souza-Filho, Luiz F. Andrade; **Event:** samplingProtocol: van Veen grabber; eventDate: 09-07-2009; year: 2009; month: 7; day: 9; habitat: fine sand sediment; fieldNumber: 075-CES23; **Record Level:** institutionCode: MOUFPE; basisOfRecord: PreservedSpecimen
- **Type status:** Paratype. **Occurrence:** catalogNumber: MOUFPE 22051; recordedBy: Jesser F. Souza-Filho | Luiz F. Andrade; individualCount: 1; sex: female; lifeStage: adult; preparations: whole animal (ETOH); previousIdentifications: *Metharpinia* sp.; occurrenceID: 6B9A18A2-26CF-5EA9-AF2A-7B73BFDCF402; **Taxon:** scientificName: *Metharpinia hirsuta* Souza-Filho & Andrade; kingdom: Animalia; phylum: Arthropoda; class: Malacostraca; order: Amphipoda; family: Phoxocephalidae; genus: Metharpinia; specificEpithet: hirsuta; taxonRank: species; scientificNameAuthorship: Souza-Filho & Andrade; nomenclaturalCode: ICZN; **Location:** locationID: http://marineregions.org/mrgid/32405; higherGeography: Atlantic Ocean; waterBody: Potiguar Basin; country: Brazil; countryCode: BR; stateProvince: Ceará; locality: Potiguar Basin, station 075-CES12; verbatimDepth: 35.5 m; verbatimLatitude: 3°08.5838'S; verbatimLongitude: 38°52.0264'W; verbatimCoordinateSystem: degrees decimal minutes; decimalLatitude: -3.1431; decimalLongitude: -38.8671; **Identification:** identifiedBy: Jesser F. Souza-Filho, Luiz F. Andrade; **Event:** samplingProtocol: van Veen grabber; eventDate: 08-07-2009; year: 2009; month: 7; day: 8; habitat: fine sand sediment; fieldNumber: 075-CES12; **Record Level:** institutionCode: MOUFPE; basisOfRecord: PreservedSpecimen

#### Description

**Figure 24.**
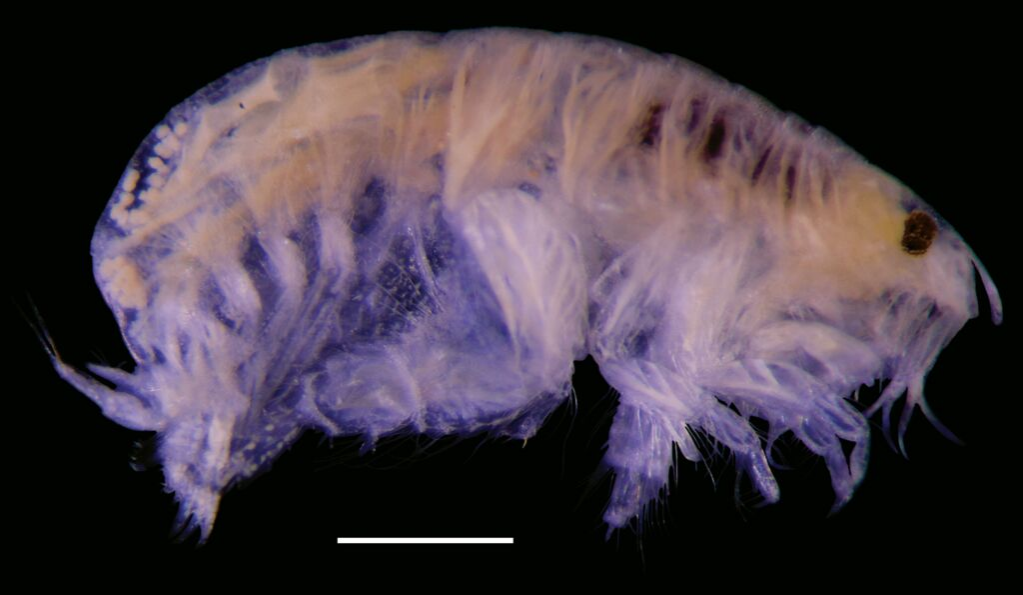
*Metharpinia hirsuta* Souza-Filho & Andrade, **sp. nov.**, female holotype (MOUFPE 22050). Habitus photograph. Scale bar: 1 mm.

**Figure 25.**
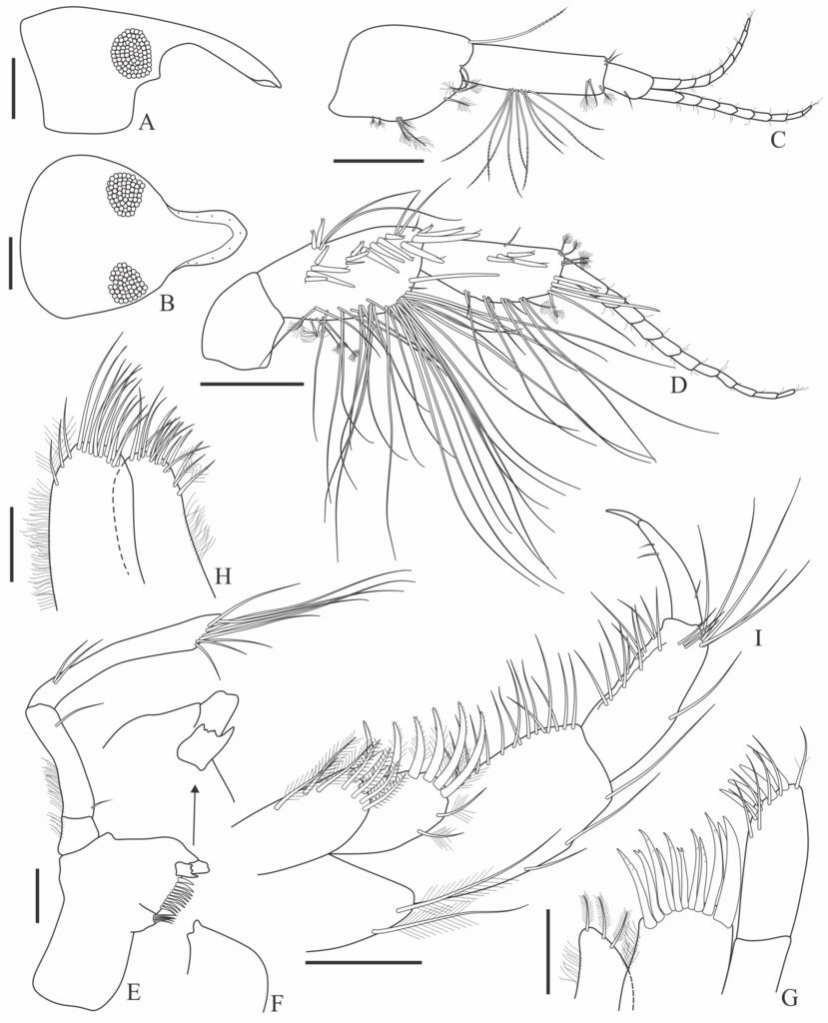
*Metharpinia hirsuta* Souza-Filho & Andrade, **sp. nov.**, female holotype (MOUFPE 22050). **A** Head lateral view; **B** Head dorsal view; **C** Antenna 1; **D** Antenna 2; **E** Left mandible; **F** Right mandible incisor; **G** Maxilla 1; **H** Maxilla 2; **I** Maxilliped. Scale bars: 0.3 mm (A–D), 0.1 mm (E–I).

**Figure 26.**
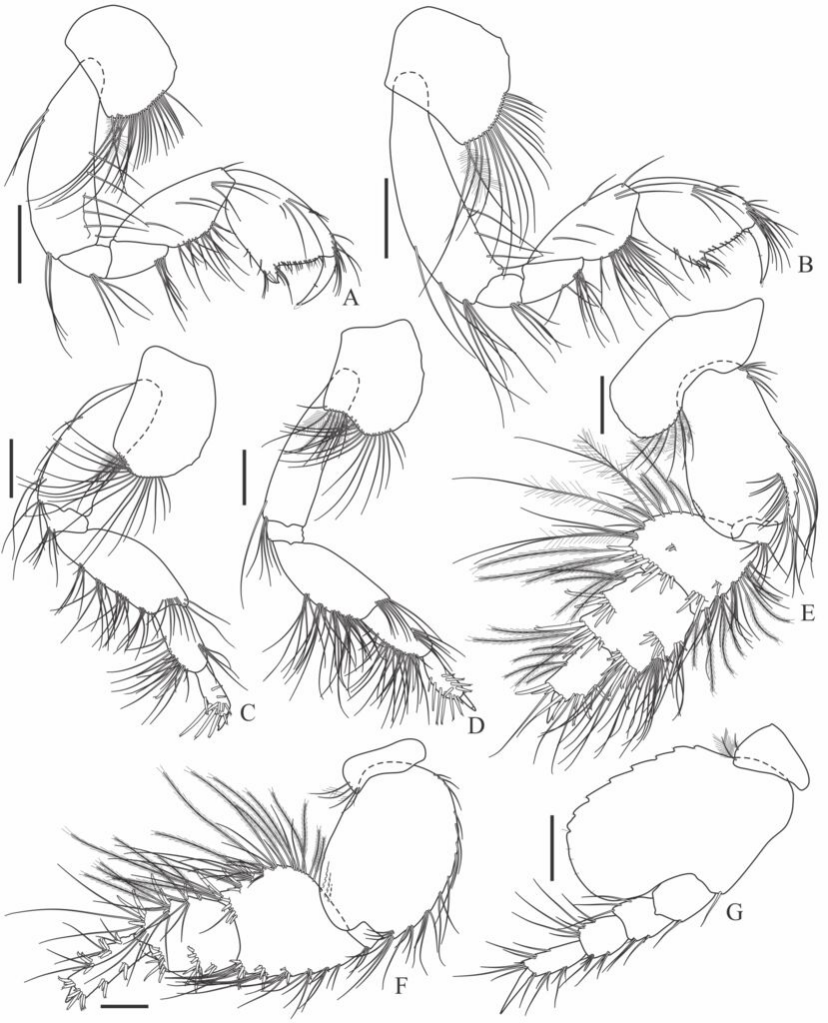
*Metharpinia hirsuta* Souza-Filho & Andrade, **sp. nov.**, female holotype (MOUFPE 22050). **A** Pereopod 1 (gnathopod 1); **B** Pereopod 2 (gnathopod 2); **C** Pereopod 3; **D** Pereopod 4; **E** Pereopod 5; **F** Pereopod 6; **G** Pereopod 7. Scale bars: 0.3 mm.

**Figure 27.**
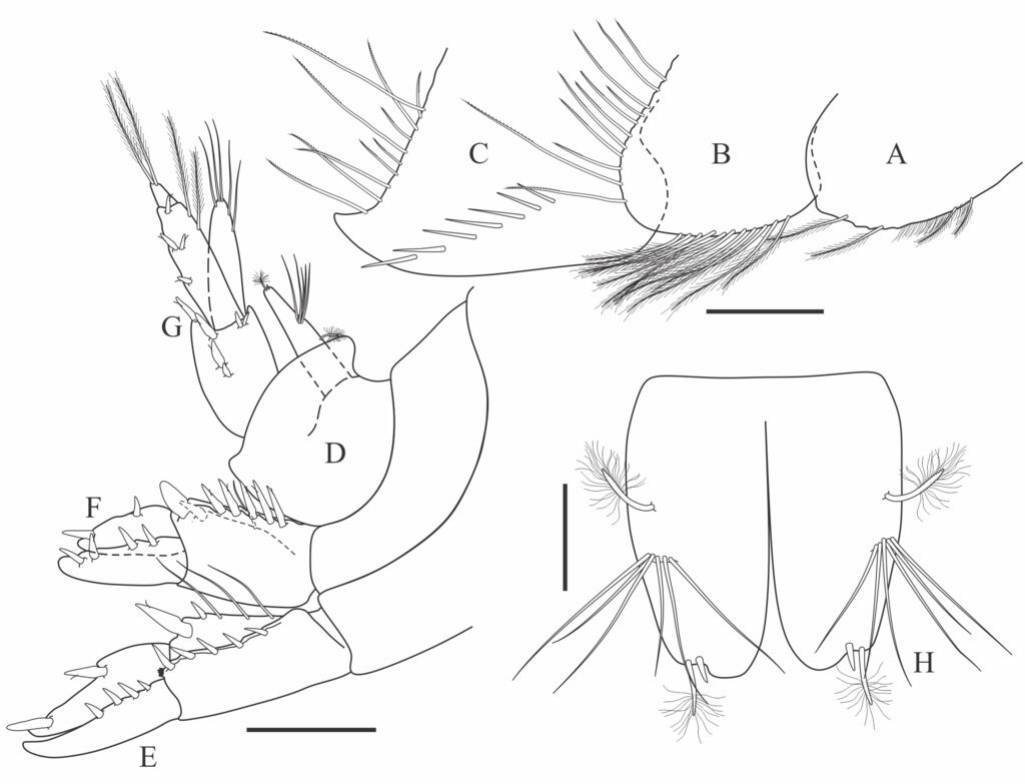
*Metharpinia hirsuta* Souza-Filho & Andrade, **sp. nov.**, female holotype (MOUFPE 22050). **A** Epimeral plate 1; **B** Epimeral plate 2; **C** Epimeral plate 3; **D** Urosomite 3; **E** Uropod 1; **F** Uropod 2; **G** Uropod 3; **H** Telson. Scale bars: 0.3 mm (A–G), 0.1 mm (H).

Holotype, MOUFPE 22050, adult female, 7.1 mm. Figs 24, 25, 26, 27.

**Body** as in Fig. 24. **Head** (Fig. 25A and B) rostrum constricted, elongate and spatulate, reaching the proximal part of A1 art2; eyes rounded. **A1** (Fig. 25C) weakly setose, art1 with few pappose brush setae, art2 elongate, posterior margin with a medial group of setae, art3 short; flagellum 9-articulate; acc flag 8-articulate. **A2** (Fig. 25D) art4 facial stout setae formula 4-4-2-4, ventral margin strongly setose with long setae, dorsal margin with two stout setae, art5 with three facial stout setae; flagellum 11-articulate. **Md** (Fig. 25E and F) incisor with three (left) and two (right) cusps, lacinia mobilis multicuspidate (left) and absent (right), molar as a hump with robust setae; palp 3-articulate, art3 long, 1.7x longer than art2, apex oblique and setose. **Mx1** (Fig. 25G) inner plate with three apical plumose setae; outer plate with 11 robust setae; palp 2-articulate, art2 apex with four robust and few simple setae. **Mx2** (Fig. 25H) inner and outer plates subequal, apically setose. **Mxp** (Fig. 25I) inner plate with plumose setae and two apical robust setae; outer plate with robust papposerrate setae distally increasing in length; palp art2 and art3 moderately setose, art4 bearing an apical nail.

**C1**–**4** (Fig. 26A, B, C and D) short, ventrally setose, with one long pappose setae (except C4 that has seven). **P1** (Fig. 26A) basis broadening distally; carpus posterior margin moderately setose; propodus weakly setose, with one stout seta defining palm; palm acute, palmar hump medium and pointed; dactylus reaching the palm length. **P2** (Fig. 26B) slightly more robust than P1; basis elongate; carpus posterior margin moderately setose; propodus weakly setose, with one stout seta defining palm; palm acute, palmar hump medium and pointed; dactylus reaching the palm length. **P3** (Fig. 26C) basis broad, merus and carpus posterior margin moderately setose, carpus distal long robust seta extending about 80% of propodus; propodus with 11 stout setae; dactylus short. **P4** (Fig. 26D) very similar to P3, but more setose in the posterior margin of merus and carpus. **P5** (Fig. 26E) coxa posteroventral margin with six pappose setae; basis broad, with an anterodistal facial row with nine setae; ischium short and subrectangular; merus margins setose, with simple, plumose and pappose setae, with facial groups of stout setae; carpus and propodus margins setose, but only posterior margin with plumose setae, with facial and lateral groups of stout setae; dactylus short. **P6** (Fig. 26F) stout; coxa posteroventral margin with four pappose setae; basis 1.4x longer than wide, anterior margin with setae; merus and carpus broad, posterior margins with plumose setae, with facial and lateral groups of stout setae; propodus short, with groups of stout setae; dactylus short. **P7** (Fig. 26G) coxa posteroventral margin with two pappose setae; basis 1.4x longer than wide, expanded posteroventrally, reaching the apex of merus, posterior margin serrate; merus, carpus and propodus moderately setose; dactylus long.

**Ep1** (Fig. 27A) with four plumose setae, posteriorly rounded. **Ep2** (Fig. 27B) ventral margin with a row of plumose setae, posterior margin with 11 pectinate setae. **Ep3** (Fig. 27C) with an oblique facial row of seven stout setae, posteroventral corner produced as a spine, posterior margin with 10 pectinate setae. **Urosomite 3** (Fig. 27D) dorsally produced as a blunt hump. **U1** (Fig. 27E) peduncle longer than rami, dorsal margin with seven robust and three simple setae; outer ramus with four dorsal stout setae proximally; inner ramus with one medial dorsal stout seta and one subapical stout seta. **U2** (Fig. 27F) peduncle subequal in length to rami, dorsal margin with seven stout setae; rami short; outer ramus with four dorsal stout setae; inner ramus with one medial dorsal stout seta and one subapical stout seta. **U3** (Fig. 27G) peduncle with six robust setae; outer ramus art1 as long as inner ramus, with four robust setae, art2 with two apical plumose setae; inner ramus with three subapical and two apical setae. **T** (Fig. 27H) about 85% cleft, each side with one dorsal short brush setae and a group of six long setae, apical margin with one stout and one brush seta.

##### Type material

Holotype (MOUFPE 22050) and one paratype.

##### Material examined

Only known from the type material.

##### Type locality

Potiguar Basin, Ceará State, Brazil.

#### Diagnosis

Head rostrum reaching the proximal part of A1 art2. A2 art4 posterior margin strongly setose with long setae. Md molar as a hump with stout setae, palp art3 longer than art1 and art2 combined. C1–3 short, ventrally setose, with one long pappose seta. P1–2 palmar hump medium and pointed. Ep3 posteroventral corner produced as a spine. Urosomite 3 dorsally produced as a blunt hump. U2 peduncle subequal in length to rami, rami short.

#### Etymology

The specific epithet *hirsuta* is derived from the Latin word hirsutus, meaning "hairy" or "bristly", in reference to the setose appendages of this species.

#### Distribution

Only known from Potiguar Basin, Ceará State, Brazil; specimens were found in fine sand sediments between 34.4 m and 35.5 m depth.

#### Taxon discussion

*Metharpinia hirsuta* Souza-Filho & Andrade, **sp. nov.** is most morphologically similar to *Metharpinia grandirama* Alonso de Pina, 2003 (Alonso de Pina 2003) mainly due to the following characters: A1 art2 elongate; U1 and U2 peduncle with a distal large robust seta, rami short and robust, with few dorsal stout setae. However, the new species can be mainly distinguished from *Metharpinia grandirama* by the following characters (characters of *Metharpinia grandirama* in parentheses): A2 art4 posterior margin strongly setose (weakly); C1–3 short, ventral margin strongly setose (long and moderately setose); P1 and P2 palmar hump medium and pointed (blunt); Ep3 posteroventral corner produced as a spine (rounded, without a spine). Urosomite 3 dorsally produced as a blunt hump (produced as a larger acute spine).

The new species can also be distinguished from its congeners by presenting a very setose art4 of A2 and C1–4 ventral margins, broadened and shortened propodus of P6 and rami of U1 and U2.

#### Methods

The preserved specimens analysed here were collected in the Potiguar Basin, north-eastern Brazil, during the project "Avaliação de Impactos Ambientais da Atividade de Perfuração nas Bacias Potiguar e Ceará (PAI UN-RNCE)". The project was developed by the Brazilian Oil Company "Petróleo Brasileiro S/A (PETROBRAS)" and carried out onboard the R/V LUKE THOMAS, using a van Veen grabber. During the campaign, sediment samples were collected for the physical-chemical and biological characterisation of the sediment around four offshore drilling wells and their respective reference areas.

Specimens were fixed and preserved in 75% ethanol. For the taxonomic study, all appendages of the holotype were dissected and mounted in glycerine gelatin slides. Appendage photographs were taken using a NIKON Eclipse Ci-L, equipped with a Delta Optical DLT-Cam PRO 5MP and subsequently used as the basis for line drawings, which were inked with CorelDRAW 2018.

**Repository**: Specimens are held in the crustacean collection of Museu de Oceanografia Petrônio Alves Coelho, Universidade Federal de Pernambuco, City of Recife, Brazil (MOUFPE).

### Zeaione

Boyko & Williams gen. nov.

3943E060-98F5-5DE3-B01B-9E9F9988F85E

0DED2B37-472B-45A5-89FC-110A2B1D962C

Zeaione everta Boyko & Williams. Type species.

#### Diagnosis

**Female** body elongate, weakly distorted (< 5°); head weakly bilobed; frontal lamina present, expanded laterally. Eyes absent. Maxilliped with setose articulated non-segmented palp, margin of anterior lobe highly setose. Barbula with three smooth lobes on each side, median region smooth. Oostegite 1 with lobe ovate, posterior lobe oblong, smaller than anterior lobe, lobes almost fused; internal ridge smooth. Coxal plates and dorsolateral bosses present; tergal projections absent. Mediodorsal lobes absent. Pereopods not elongate, without propodal sockets. Pleon not abruptly narrower than pereon; dorsolateral margins of pleomeres 1–6 with numerous, irregular, mostly semi-spherical protuberances. Lateral plates broad, lamellar, with scalloped posterior margins; five pairs of smooth biramous pleopods with endopod bilobed, with scalloped posterior margins; uropods biramous, smooth, lamellar. **Male** approximately 3 times as long as wide, head narrower than pereon, pereomeres slightly narrower posteriorly. Small, irregular eyes present. Segmented maxillipeds present, distal segment with terminal setae. Pereopods subequal. Mid-ventral tubercles absent. Pleon of six pleomeres; lateral plates absent; five pairs of uniramous low rounded pleopods; posterolateral corners of pleomere 6 extended; anal cone present; uropods absent.

#### Etymology

The genus name is a combination of *Zea* (after the corn genus *Zea* Linnaeus, 1753) and -*ione* (a common suffix for epicaridean genera) and, in combination with the type species name, refers to the resemblance between the irregular protuberances on the dorsolateral margins of the female pleomeres and popped kernels of popcorn. The gender is feminine.

### Zeaione everta

Boyko & Williams sp. nov.

FEEEA8CE-792D-57AF-AD19-3840A1CA5996

304A9242-DE90-4623-ABE0-F15E20D9855C

#### Materials

- **Type status:** Holotype. **Occurrence:** catalogNumber: NMV J62877; occurrenceRemarks: infesting left branchial chamber of female *Eucalliaxiopsis aequimana* (Baker, 1907) (9.6 mm carapace length; MV J59759); recordedBy: Museum Research Group of Victoria; individualCount: 1; sex: female; lifeStage: adult; reproductiveCondition: mature; preparations: 70% EtOH; occurrenceID: F3E9A10D-072C-57F1-A9DA-57A45CDC716B; **Taxon:** scientificName: *Zeaione everta* Boyko & Williams; kingdom: Animalia; phylum: Arthropoda; class: Malacostraca; order: Isopoda; family: Bopyridae; genus: Zeaione; specificEpithet: everta; taxonRank: species; scientificNameAuthorship: Boyko & Williams; nomenclaturalCode: ICZN; **Location:** higherGeography: Pacific Ocean; continent: Australia; country: Australia; stateProvince: Victoria; locality: San Remo, transect 4, northwest from rock outcrop 1.1 km; verbatimDepth: intertidal; verbatimCoordinates: 38°32'S, 145°23'E; verbatimLatitude: 38°32'S; verbatimLongitude: 145°23'E; **Identification:** identifiedBy: G.C.B. Poore (host); C.B. Boyko, J.D. Williams (parasite); **Event:** samplingProtocol: hand collected; eventDate: 13-03-1993; habitat: intertidal sediment; **Record Level:** institutionID: http://grscicoll.org/institution/museum-victoria; institutionCode: NMV; basisOfRecord: PreservedSpecimen
- **Type status:** Paratype. **Occurrence:** catalogNumber: NMV J76267; occurrenceRemarks: infesting left branchial chamber of female *Eucalliaxiopsis aequimana* (Baker, 1907) (9.6 mm carapace length; MV J59759); recordedBy: Museum Research Group of Victoria; individualCount: 1; sex: male; lifeStage: adult; reproductiveCondition: mature; preparations: 70% EtOH; occurrenceID: 1C2648FB-0E92-51B1-ABC2-4C83D475554C; **Taxon:** scientificName: *Zeaione everta* Boyko & Williams; kingdom: Animalia; phylum: Arthropoda; class: Malacostraca; order: Isopoda; family: Bopyridae; genus: Zeaione; specificEpithet: everta; taxonRank: species; scientificNameAuthorship: Boyko & Williams; nomenclaturalCode: ICZN; **Location:** higherGeography: Pacific Ocean; continent: Australia; country: Australia; stateProvince: Victoria; locality: San Remo, transect 4, northwest from rock outcrop 1.1 km; verbatimDepth: intertidal; **Identification:** identifiedBy: G.C.B. Poore (host); C.B. Boyko, J.D. Williams (parasite); **Event:** samplingProtocol: hand collected; eventDate: 13-03-1993; habitat: intertidal sediment; **Record Level:** institutionID: http://grscicoll.org/institution/museum-victoria; institutionCode: NMV; basisOfRecord: PreservedSpecimen

#### Description

**Figure 28.**
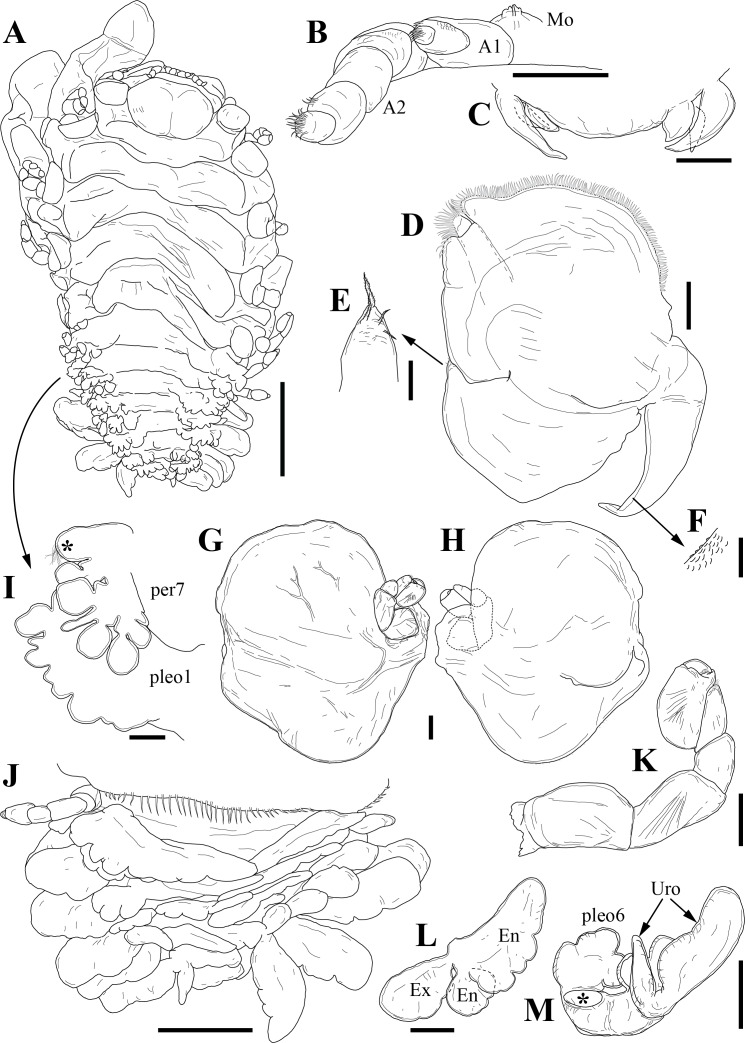
*Zeaione everta* Boyko & Williams, **gen. et sp. nov.**, adult female holotype (NMV J62877). **A** Habitus, dorsal view; **B** Right antennule, antenna and mouthparts, ventral view; **C** Barbula, dashed lines indicate third extension behind outer two extensions; **D** Left maxilliped with outer barbula lobe, external view. **E** Maxilliped spur, close-up showing setae. **F** Scales on outer barbula lobe; **G** Left oostegite 1, external view; **H** Left oostegite 1, internal view; **I** Dorsolateral view of pereomere 7 and pleomere 1 showing irregular protuberences and thalli of an unidentified species of Eccrinales (indicated by asterisk); **J** Pleon, ventral view; **K** Right pereopod 7; **L** Right pleopod 1; **M** Pleomere 6 showing biramous uropod on right side, scar of left uropod shown by asterisk. Abbreviations: A1 = antennule, A2 = antenna, Ex = exopod, En = endopod, Mo = mouthparts, per7 = pereomere 7, pleo1, 6 = pleomere 1, 6, Uro = uropod. Scale bars: 2 mm (A), 250 µm (B, D, G–I, K), 500 µm (C, L, M), 50 µm (E, F), 1 mm (J).

**Female holotype** (Fig. 28) body length 8.7 mm, maximal width 5.1 mm, head length 1.2 mm, head width 1.9 mm, pleon length 2.3 mm. Body elongate (Fig. 28A), approximately straight, head deflected very slightly to right (sinistrally rotated < 5°).

**Head** ovate (Fig. 28A), wider than long, anterior margin straight, narrow frontal lamina extending slightly beyond lateral margins of head, head weakly bilobed. Eyes absent. Barbula with three smooth tapered lobes on each side, smallest lobe covered by other two, median region smooth (Fig. 28C, D, F). Antennules (Fig. 28B) of three articles each, extending beyond anterior margin of head; antennae (Fig. 28B) of five articles each; terminal two articles of antennules and antennae with distal setae. Maxilliped (Fig. 28D–F) anterior lobe broad, rounded, with low rounded articulated palp, palp and anterior margin with many setae; posterior lobe with small, rounded spur with few plumose setae (Fig. 28E). First oostegite anterior lobe ovate, posterior lobe oblong, smaller than anterior lobe, with small setae along posterior margin, lobes almost fused, internal ridge smooth, only present on mesiad half of inner surface (Fig. 28G, H).

**Pereon** (Fig. 28A) of seven pereomeres, broadest across pereomeres 2 and 3, tapering anteriorly and posteriorly. Dorsolateral margins of pereomeres 6 and 7 with numerous, irregular, mostly semi-spherical protuberances (Fig. 28I). Small coxal plates on sides of pereomeres 1–5; dorsolateral bosses on pereomeres 1–5. Oostegites smooth; oostegite 5 with fringe of setae on posterior margin (Fig. 28J). Pereopods 1–7 (Fig. 28G, K) subequal in size and of similar morphology, small curved dactylus, ovate propodus, triangular carpus, small triangular merus, elongate ischium and subquadrate basis, basis more elongate on posterior pereopods; carpus with stout setae on distal tip (Fig. 28K).

**Pleon** (Fig. 28A, J) of six pleomeres, dorsolateral margins of pleomeres 1–6 with numerous, irregular, mostly semi-spherical protuberances (Fig. 28I). Pleomeres 1–5 with biramous, lamellar pleopods with scalloped posterior margins, decreasing in size posteriorly (Fig. 28J); exopod ovate; endopod bilobed, outer lobe smaller, round, inner lobe elongate (Fig. 28L). Five pairs of broad lateral plates, lamellar, with scalloped posterior margins. Pleotelson with biramous, elongate lamellar uropods (missing on left side), inner lobe smaller (Fig. 28M).

**Figure 29.**
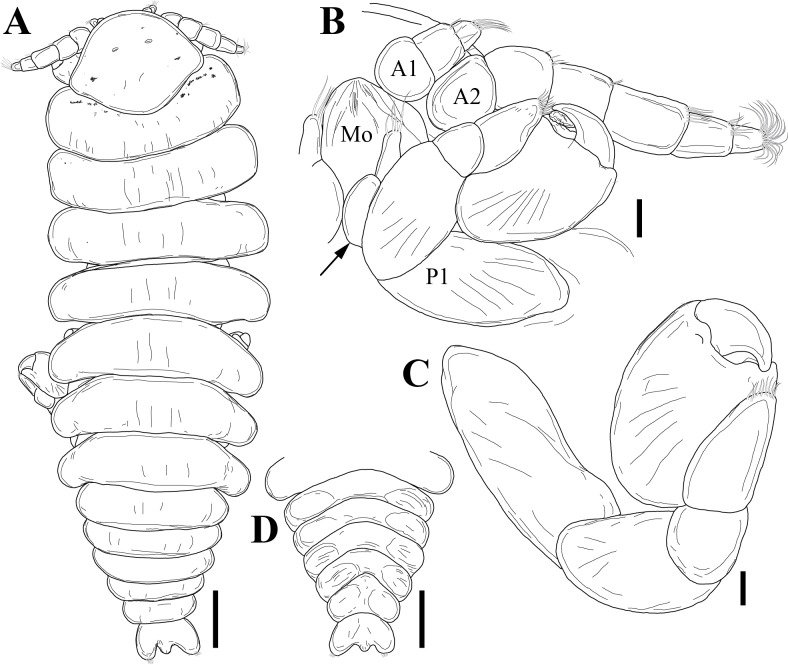
*Zeaione everta* Boyko & Williams, **gen. et sp. nov.**, adult male paratype (allotype) (NMV J62877a). **A** Habitus, dorsal view; **B** Left antennule, antenna, mouthparts, maxillipeds (base shown by arrow) and pereopod 1, ventral view; **C** Left pereopod 7; **D** Pleon, ventral view. Abbreviations: A1 = antennule, A2 = antenna, Mo = mouthparts, P7 = pereopod 7. Scale bars: 250 mm (A, D), 50 mm (B, C).

**Male paratype (allotype)** (Fig. 29) body length 2.8 mm, maximal width 1.0 mm, head length 0.5 mm, head width 0.6 mm, pleon length 0.7 mm. Body elongate. Small, irregular patches of dark pigmentation on anterior portion of pereomere 1.

**Head** distinct from segment 1, anterior margin of head narrow and rounded, head widest medially (Fig. 29A), small eyespots mediolaterally. Antennules of three articles each, extending slightly beyond anterior margin of head; antennae of six robust articles each, extending well beyond lateral margins of head; antennae and antennules with few distal setae on each segment, except for basal one; terminal segments of each with tuft of numerous setae (Fig. 29B). Maxilliped present, two-segmented, distal segment with four long simple setae (Fig. 29B).

**Pereon** of seven pereomeres, broadest across pereomeres 3 and 4, slightly tapering anteriorly and posteriorly. Lateral margins of pereomeres 1 and 2 directed anteriorly, 3 and 4 directed laterally, 5–7 directed posteriorly. Pereopods (Fig. 29B, C) subequal; all articles distinct, curved dactylus with minute setae, ovate propodus, triangular carpus, rounded short merus, elongate ischium and basis; carpus with stout setae at anterior tip; pereopod 7 (Fig. 29C) similar in morphology to pereopod 1, except dactylus slightly shorter, propodus proportionally larger and basis more elongate.

**Pleon** (Fig. 29A, D) of six pleomeres, markedly narrower than pereon, pleomeres rounded posteriorly, all pleomeres distinctly segmented, pleomeres 1­–5 with very low rounded pleopods; no mid-ventral tubercles (Fig. 29D). Pleotelson (Fig. 29A, D) indented medially with robust anal cone, distolaterally produced into rounded lobes, with scales and setae; uropods absent.

##### Type material

Holotype (NMV J62877) and allotype (NMV J62877a). The holotype is an adult female; the paratype (allotype) is an adult male.

##### Material examined

Holotype (NMV J62877), ♀ adult, in intertidal sediment, infesting left branchial chamber of ♀ *Eucalliaxiopsis aequimana*.

Paratype (allotype) (NMV J62877a), ♂ adult, same data as holotype.

##### Type locality

Pacific Ocean, Australia, Victoria, San Remo, 38°32'S, 145°23'E, intertidal.

#### Diagnosis

As for genus.

#### Etymology

The species name is given after the historical name of the corn variety Zea mays var. everta Bailey, 1925 (now considered a synonym of *Z. mays mays* Linnaeus, 1753) and, in combination with the genus name, refers to the resemblance between the irregular protuberances on the dorsolateral margins of the female pleomeres and popped kernels of popcorn.

#### Distribution

Only known from type locality.

#### Ecology

**Host**: *Eucalliaxiopsis aequimana* (Baker, 1907) (Crustacea, Decapoda, Axiidea, Eucalliacidae Manning & Felder, 1991), an axiidean shrimp known from intertidal to subtidal sediments in New South Wales (as far north as 33°S), Tasmania, Victoria, South Australia, Western Australia (as far north as 25°S), Australia (Poore 2021). The female host was cited by Poore (2021), but the presence of the parasite was not noted.

**Additional associates**: Many patches of long, thin thalli with septa were observed on some pereomeres (Fig. 28I), pleomeres and appendages of the holotype. We interpret these as an unidentified species of Ichthyosporea (formerly Mesomycetozoea): Eccrinales that are known to be ecto- and endosymbionts associated with crustaceans and other hosts (e.g. Boyko et al. (2017), Williams et al. (2019), Shields (2022) and Shabardina et al. (2024)).

#### Taxon discussion

The present record of a bopyrid parasitising *Eucalliaxiopsis aequimana* is unique amongst the 45 valid species described in Eucalliacidae. This is perhaps not surprising, given that only 15 hosts in Axiidea identified to species have previously been reported harbouring bopyrids (Markham 2001, An et al. 2009, Romero-Rodríguez and Álvarez 2025) out of the 927 valid species in Axiidea (WoRMS 2024). Clearly, axiideans are a group that is greatly undersampled in terms of the diversity of bopyrids and almost certainly in terms of other parasites as well.

The new genus and species reported herein is a member of Pseudioninae but, while the male resembles those belonging to a number of genera and is relatively non-descript, the female with the popcorn-like protuberances on the posterior pereon and pleon and possessing bilobed pleopodal endopods is not similar to that of any other described pseudionine species. Females of the genus *Ionella* possess extensive digitation on the sides of the pereomeres and pleomeres (Romero-Rodríguez and Álvarez 2025) similar to those of some females of species belonging to Keponinae and Ionidae associated with axiidean hosts, but these long, thin digitate extensions are also found on the lateral plates and other surfaces, unlike in the new genus where the protuberances are rounded. Other female characters and those of males are very different between these taxa and the new genus. Digitations or protuberances may be an adaptation (e.g. increased surface area for respiration) found in those bopyroids associated with axiidean and other tube-dwelling shrimp hosts.

#### Methods

Line drawings of the bopyrid isopods were made by using camera lucida drawing tubes attached to Olympus compound (OLYMPUS CX31) and dissecting microscopes (OLYMPUS SZX12). Adobe Illustrator and a WACOM Cintiq pen display were used to trace original sketches and produce final figures. All parasite specimen measurements were made from camera lucida drawing tube sketches and slide micrometers. Morphological terminology follows that of Boyko et al. (2017).

**Repository**: The type specimens of the new species were sourced from the Museums Victoria (formerly National Museum of Victoria, NMV) and are deposited there.

### Haploniscus bulbosus

Henseler, Knauber & Riehl sp. nov.

60B97DC8-3D49-5B82-B105-DBF3BD4D16ED

D93623F0-8CB9-4CD4-9BAA-A39CECF1F02A

https://www.marinespecies.org/aphia.php?p=taxdetails&id=118351

#### Materials

- **Type status:** Holotype. **Occurrence:** catalogNumber: SMF 62946; recordNumber: KBHap003; individualCount: 1; sex: male; lifeStage: adult; preparations: whole animal (ETOH); occurrenceID: C7CBAC4D-BD5E-557E-B66D-85C1D2AF559A; **Taxon:** scientificName: *Haploniscus bulbosus* Henseler, Knauber & Riehl; kingdom: Animalia; phylum: Arthropoda; class: Malacostraca; order: Isopoda; family: Haploniscidae; genus: Haploniscus; specificEpithet: bulbosus; scientificNameAuthorship: Henseler, Knauber & Riehl; nomenclaturalCode: ICZN; **Location:** higherGeography: Pacific Ocean; locality: North-west Pacific Ocean, KuramBio station SO223-10-09; verbatimDepth: 5264-5266 m; verbatimLatitude: 41°11'16.188''N; verbatimLongitude: 150°5'36.888''E; decimalLatitude: 41.18783; decimalLongitude: 150.09358; **Identification:** identifiedBy: Henry Knauber; **Event:** samplingProtocol: Benthos trawl, Camera-Epibenthic Sledge, sieved through 0.3 mm mesh | Riehl T, Brenke N, Brix S, Driskell A, Kaiser S, Brandt A (2014) Field and laboratory methods for DNA studies on deep-sea isopod crustaceans. Polish Polar Research 35: 205–226. https://doi.org/10.2478/popore−2014−0018 | Brandt A, Malyutina MV (2012) The German-Russian deep-sea expedition KuramBio (Kurile Kamchatka Biodiversity Study) : to the Kurile Kamchatka Trench and abyssal plain on board of the R/V SONNE, 223rd Expedition, 21 July - 7 September 2012. University of Hamburg; Biozentrum Grindel & Zoologisches Museum Hamburg, Hamburg. Cruise Report, 100pp. Available from: http://edok01.tib.uni-hannover.de/edoks/e01fb13/741102293.pdf.; eventDate: 26.08.2012; habitat: abyssal sediment; fieldNumber: SO223-10-09; **Record Level:** collectionID: Crustacea; institutionCode: SMF; basisOfRecord: PreservedSpecimen
- **Type status:** Paratype. **Occurrence:** catalogNumber: SMF 62947; recordNumber: KBHap004; individualCount: 1; sex: male; lifeStage: adult; preparations: whole animal (ETOH); occurrenceID: 1F5497CF-228B-5015-B6B1-293994CF9A30; **Taxon:** scientificName: *Haploniscus bulbosus* Henseler, Knauber & Riehl; kingdom: Animalia; phylum: Arthropoda; class: Malacostraca; order: Isopoda; family: Haploniscidae; genus: Haploniscus; specificEpithet: bulbosus; scientificNameAuthorship: Henseler, Knauber & Riehl; nomenclaturalCode: ICZN; **Location:** higherGeography: Pacific Ocean; locality: North-west Pacific Ocean, KuramBio station SO223-10-09; verbatimDepth: 5264-5266 m; verbatimLatitude: 41°11'16.188''N; verbatimLongitude: 150°5'36.888''E; decimalLatitude: 41.18783; decimalLongitude: 150.09358; **Identification:** identifiedBy: Henry Knauber; **Event:** samplingProtocol: Benthos trawl, Camera-Epibenthic Sledge, sieved through 0.3 mm mesh | Riehl T, Brenke N, Brix S, Driskell A, Kaiser S, Brandt A (2014) Field and laboratory methods for DNA studies on deep-sea isopod crustaceans. Polish Polar Research 35: 205–226. https://doi.org/10.2478/popore−2014−0018 | Brandt A, Malyutina MV (2012) The German-Russian deep-sea expedition KuramBio (Kurile Kamchatka Biodiversity Study) : to the Kurile Kamchatka Trench and abyssal plain on board of the R/V SONNE, 223rd Expedition, 21 July - 7 September 2012. University of Hamburg; Biozentrum Grindel & Zoologisches Museum Hamburg, Hamburg. Cruise Report, 100 pp. Available from: http://edok01.tib.uni-hannover.de/edoks/e01fb13/741102293.pdf.; eventDate: 26.08.2012; habitat: abyssal sediment; fieldNumber: SO223-10-09; **Record Level:** collectionID: Crustacea; institutionCode: SMF; basisOfRecord: PreservedSpecimen
- **Type status:** Other material. **Occurrence:** catalogNumber: SMF 62948; recordNumber: KBHap005; individualCount: 1; sex: female; lifeStage: adult; preparations: whole animal (ETOH); occurrenceID: 297F6FA9-A715-5427-BD57-EBCC4CEDFAAB; **Taxon:** scientificName: *Haploniscus bulbosus* Henseler, Knauber & Riehl; kingdom: Animalia; phylum: Arthropoda; class: Malacostraca; order: Isopoda; family: Haploniscidae; genus: Haploniscus; specificEpithet: bulbosus; scientificNameAuthorship: Henseler, Knauber & Riehl; nomenclaturalCode: ICZN; **Location:** higherGeography: Pacific Ocean; locality: North-west Pacific Ocean KuramBio station SO223-10-09; verbatimDepth: 5264-5266 m; verbatimLatitude: 41°11'16.188''N; verbatimLongitude: 150°5'36.888''E; decimalLatitude: 41.18783; decimalLongitude: 150.09358; **Identification:** identifiedBy: Henry Knauber; **Event:** samplingProtocol: Benthos trawl, Camera-Epibenthic Sledge, sieved through 0.3 mm mesh | Riehl T, Brenke N, Brix S, Driskell A, Kaiser S, Brandt A (2014) Field and laboratory methods for DNA studies on deep-sea isopod crustaceans. Polish Polar Research 35: 205–226. https://doi.org/10.2478/popore−2014−0018 | Brandt A, Malyutina MV (2012) The German-Russian deep-sea expedition KuramBio (Kurile Kamchatka Biodiversity Study) : to the Kurile Kamchatka Trench and abyssal plain on board of the R/V SONNE, 223rd Expedition, 21 July - 7 September 2012. University of Hamburg; Biozentrum Grindel & Zoologisches Museum Hamburg, Hamburg. Cruise Report, 100pp. Available from: http://edok01.tib.uni-hannover.de/edoks/e01fb13/741102293.pdf.; eventDate: 26.08.2012; habitat: abyssal sediment; fieldNumber: SO223-10-09; **Record Level:** institutionCode: SMF; basisOfRecord: PreservedSpecimen
- **Type status:** Other material. **Occurrence:** catalogNumber: SMF 62949; recordNumber: KBHap011; individualCount: 1; sex: female; lifeStage: adult; preparations: whole animal (ETOH); occurrenceID: C3ADCEA9-307F-5AF8-86FB-BF41665F52AD; **Taxon:** scientificName: *Haploniscus bulbosus* Henseler, Knauber & Riehl; kingdom: Animalia; phylum: Arthropoda; class: Malacostraca; order: Isopoda; family: Haploniscidae; genus: Haploniscus; specificEpithet: bulbosus; scientificNameAuthorship: Henseler, Knauber & Riehl; nomenclaturalCode: ICZN; **Location:** higherGeography: Pacific Ocean; locality: North-west Pacific Ocean, KuramBio station SO223-09-09; verbatimDepth: 5399-5421 m; verbatimLatitude: 40°34'22.8''N; verbatimLongitude: 150°59'54.888''E; decimalLatitude: 40.573; decimalLongitude: 150.99858; **Identification:** identifiedBy: Henry Knauber; **Event:** samplingProtocol: Benthos trawl, Camera-Epibenthic Sledge, sieved through 0.3 mm mesh | Riehl T, Brenke N, Brix S, Driskell A, Kaiser S, Brandt A (2014) Field and laboratory methods for DNA studies on deep-sea isopod crustaceans. Polish Polar Research 35: 205–226. https://doi.org/10.2478/popore−2014−0018 | Brandt A, Malyutina MV (2012) The German-Russian deep-sea expedition KuramBio (Kurile Kamchatka Biodiversity Study) : to the Kurile Kamchatka Trench and abyssal plain on board of the R/V SONNE, 223rd Expedition, 21 July - 7 September 2012. University of Hamburg; Biozentrum Grindel & Zoologisches Museum Hamburg, Hamburg. Cruise Report, 100pp. Available from: http://edok01.tib.uni-hannover.de/edoks/e01fb13/741102293.pdf.; eventDate: 23.08.2012; habitat: abyssal sediment; fieldNumber: SO223-09-09; **Record Level:** institutionCode: SMF; basisOfRecord: PreservedSpecimen
- **Type status:** Other material. **Occurrence:** catalogNumber: SMF 62950; recordNumber: KBHap012; individualCount: 1; sex: female; lifeStage: adult; preparations: whole animal (ETOH); occurrenceID: DE20034C-9543-50F7-BE92-8F2982423588; **Taxon:** scientificName: *Haploniscus bulbosus* Henseler, Knauber & Riehl; kingdom: Animalia; phylum: Arthropoda; class: Malacostraca; order: Isopoda; family: Haploniscidae; genus: Haploniscus; specificEpithet: bulbosus; scientificNameAuthorship: Henseler, Knauber & Riehl; nomenclaturalCode: ICZN; **Location:** higherGeography: Pacific Ocean; locality: North-west Pacific Ocean, KuramBio station SO223-09-09; verbatimDepth: 5399-5421 m; verbatimLatitude: 40°34'22.8''N; verbatimLongitude: 150°59'54.888''E; decimalLatitude: 40.573; decimalLongitude: 150.99858; **Identification:** identifiedBy: Henry Knauber; **Event:** samplingProtocol: Benthos trawl, Camera-Epibenthic Sledge, sieved through 0.3 mm mesh | Riehl T, Brenke N, Brix S, Driskell A, Kaiser S, Brandt A (2014) Field and laboratory methods for DNA studies on deep-sea isopod crustaceans. Polish Polar Research 35: 205–226. https://doi.org/10.2478/popore−2014−0018 | Brandt A, Malyutina MV (2012) The German-Russian deep-sea expedition KuramBio (Kurile Kamchatka Biodiversity Study) : to the Kurile Kamchatka Trench and abyssal plain on board of the R/V SONNE, 223rd Expedition, 21 July - 7 September 2012. University of Hamburg; Biozentrum Grindel & Zoologisches Museum Hamburg, Hamburg. Cruise Report, 100pp. Available from: http://edok01.tib.uni-hannover.de/edoks/e01fb13/741102293.pdf.; eventDate: 23.08.2012; habitat: abyssal sediment; fieldNumber: SO223-09-09; **Record Level:** institutionCode: SMF; basisOfRecord: PreservedSpecimen
- **Type status:** Other material. **Occurrence:** catalogNumber: SMF 62951; recordNumber: KBHap013; individualCount: 1; sex: male; lifeStage: adult; preparations: whole animal (ETOH); occurrenceID: DDC1F330-B393-54FF-B6BD-3E96D6A23ED4; **Taxon:** scientificName: *Haploniscus bulbosus* Henseler, Knauber & Riehl; kingdom: Animalia; phylum: Arthropoda; class: Malacostraca; order: Isopoda; family: Haploniscidae; genus: Haploniscus; specificEpithet: bulbosus; scientificNameAuthorship: Henseler, Knauber & Riehl; nomenclaturalCode: ICZN; **Location:** higherGeography: Pacific Ocean; locality: North-west Pacific Ocean, KuramBio station SO223-09-09; verbatimDepth: 5399-5421 m; verbatimLatitude: 40°34'22.8''N; verbatimLongitude: 150°59'54.888''E; decimalLatitude: 40.573; decimalLongitude: 150.99858; **Identification:** identifiedBy: Henry Knauber; **Event:** samplingProtocol: Benthos trawl, Camera-Epibenthic Sledge, sieved through 0.3 mm mesh | Riehl T, Brenke N, Brix S, Driskell A, Kaiser S, Brandt A (2014) Field and laboratory methods for DNA studies on deep-sea isopod crustaceans. Polish Polar Research 35: 205–226. https://doi.org/10.2478/popore−2014−0018 | Brandt A, Malyutina MV (2012) The German-Russian deep-sea expedition KuramBio (Kurile Kamchatka Biodiversity Study) : to the Kurile Kamchatka Trench and abyssal plain on board of the R/V SONNE, 223rd Expedition, 21 July - 7 September 2012. University of Hamburg; Biozentrum Grindel & Zoologisches Museum Hamburg, Hamburg. Cruise Report, 100pp. Available from: http://edok01.tib.uni-hannover.de/edoks/e01fb13/741102293.pdf.; eventDate: 23.08.2012; habitat: abyssal sediment; fieldNumber: SO223-09-09; **Record Level:** institutionCode: SMF; basisOfRecord: PreservedSpecimen
- **Type status:** Other material. **Occurrence:** catalogNumber: SMF 62952; recordNumber: KBHap014; individualCount: 1; sex: female; lifeStage: ovigerous; preparations: whole animal (ETOH); occurrenceID: 1C11618E-1084-5692-9F9D-9DA52699D85B; **Taxon:** scientificName: *Haploniscus bulbosus* Henseler, Knauber & Riehl; kingdom: Animalia; phylum: Arthropoda; class: Malacostraca; order: Isopoda; family: Haploniscidae; genus: Haploniscus; specificEpithet: bulbosus; scientificNameAuthorship: Henseler, Knauber & Riehl; nomenclaturalCode: ICZN; **Location:** higherGeography: Pacific Ocean; locality: North-west Pacific Ocean, KuramBio station SO223-09-09; verbatimDepth: 5399-5421 m; verbatimLatitude: 40°34'22.8''N; verbatimLongitude: 150°59'54.888''E; decimalLatitude: 40.573; decimalLongitude: 150.99858; **Identification:** identifiedBy: Henry Knauber; **Event:** samplingProtocol: Benthos trawl, Camera-Epibenthic Sledge, sieved through 0.3 mm mesh | Riehl T, Brenke N, Brix S, Driskell A, Kaiser S, Brandt A (2014) Field and laboratory methods for DNA studies on deep-sea isopod crustaceans. Polish Polar Research 35: 205–226. https://doi.org/10.2478/popore−2014−0018 | Brandt A, Malyutina MV (2012) The German-Russian deep-sea expedition KuramBio (Kurile Kamchatka Biodiversity Study) : to the Kurile Kamchatka Trench and abyssal plain on board of the R/V SONNE, 223rd Expedition, 21 July - 7 September 2012. University of Hamburg; Biozentrum Grindel & Zoologisches Museum Hamburg, Hamburg. Cruise Report, 100pp. Available from: http://edok01.tib.uni-hannover.de/edoks/e01fb13/741102293.pdf.; eventDate: 23.08.2012; habitat: abyssal sediment; fieldNumber: SO223-09-09; **Record Level:** institutionCode: SMF; basisOfRecord: PreservedSpecimen
- **Type status:** Paratype. **Occurrence:** catalogNumber: SMF 62953; recordNumber: KBHap015; individualCount: 1; sex: female; lifeStage: adult; preparations: whole animal (ETOH); occurrenceID: 01DE2A09-15AB-5C55-A11F-56C724499F9F; **Taxon:** scientificName: *Haploniscus bulbosus* Henseler, Knauber & Riehl; kingdom: Animalia; phylum: Arthropoda; class: Malacostraca; order: Isopoda; family: Haploniscidae; genus: Haploniscus; specificEpithet: bulbosus; scientificNameAuthorship: Henseler, Knauber & Riehl; nomenclaturalCode: ICZN; **Location:** higherGeography: Pacific Ocean; locality: North-west Pacific Ocean, KuramBio station SO223-10-09; verbatimDepth: 5264-5266 m; verbatimLatitude: 41°11'16.188''N; verbatimLongitude: 150°5'36.888''E; decimalLatitude: 41.18783; decimalLongitude: 150.09358; **Identification:** identifiedBy: Henry Knauber; **Event:** samplingProtocol: Benthos trawl, Camera-Epibenthic Sledge, sieved through 0.3 mm mesh | Riehl T, Brenke N, Brix S, Driskell A, Kaiser S, Brandt A (2014) Field and laboratory methods for DNA studies on deep-sea isopod crustaceans. Polish Polar Research 35: 205–226. https://doi.org/10.2478/popore−2014−0018 | Brandt A, Malyutina MV (2012) The German-Russian deep-sea expedition KuramBio (Kurile Kamchatka Biodiversity Study) : to the Kurile Kamchatka Trench and abyssal plain on board of the R/V SONNE, 223rd Expedition, 21 July - 7 September 2012. University of Hamburg; Biozentrum Grindel & Zoologisches Museum Hamburg, Hamburg. Cruise Report, 100 pp. Available from: http://edok01.tib.uni-hannover.de/edoks/e01fb13/741102293.pdf.; eventDate: 26.08.2012; habitat: abyssal sediment; fieldNumber: SO223-10-09; **Record Level:** institutionCode: SMF; basisOfRecord: PreservedSpecimen
- **Type status:** Other material. **Occurrence:** catalogNumber: SMF 62954; recordNumber: KBHap016; individualCount: 1; sex: female; lifeStage: adult; preparations: whole animal (ETOH); occurrenceID: 286E2FC4-BDA3-5A4D-BDFF-045F736C159E; **Taxon:** scientificName: *Haploniscus bulbosus* Henseler, Knauber & Riehl; kingdom: Animalia; phylum: Arthropoda; class: Malacostraca; order: Isopoda; family: Haploniscidae; genus: Haploniscus; specificEpithet: bulbosus; scientificNameAuthorship: Henseler, Knauber & Riehl; nomenclaturalCode: ICZN; **Location:** higherGeography: Pacific Ocean; locality: North-west Pacific Ocean, KuramBio station SO223-10-09; verbatimDepth: 5264-5266 m; verbatimLatitude: 41°11'16.188''N; verbatimLongitude: 150°5'36.888''E; decimalLatitude: 41.18783; decimalLongitude: 150.09358; **Identification:** identifiedBy: Henry Knauber; **Event:** samplingProtocol: Benthos trawl, Camera-Epibenthic Sledge, sieved through 0.3 mm mesh | Riehl T, Brenke N, Brix S, Driskell A, Kaiser S, Brandt A (2014) Field and laboratory methods for DNA studies on deep-sea isopod crustaceans. Polish Polar Research 35: 205–226. https://doi.org/10.2478/popore−2014−0018 | Brandt A, Malyutina MV (2012) The German-Russian deep-sea expedition KuramBio (Kurile Kamchatka Biodiversity Study) : to the Kurile Kamchatka Trench and abyssal plain on board of the R/V SONNE, 223rd Expedition, 21 July - 7 September 2012. University of Hamburg; Biozentrum Grindel & Zoologisches Museum Hamburg, Hamburg. Cruise Report, 100pp. Available from: http://edok01.tib.uni-hannover.de/edoks/e01fb13/741102293.pdf.; eventDate: 26.08.2012; habitat: abyssal sediment; fieldNumber: SO223-10-09; **Record Level:** institutionCode: SMF; basisOfRecord: PreservedSpecimen
- **Type status:** Other material. **Occurrence:** catalogNumber: SMF 62955; recordNumber: KBHap017; individualCount: 1; sex: female; lifeStage: adult; preparations: whole animal (ETOH); occurrenceID: CBD96BF5-EFC2-5049-A53D-A52EB5DE1A9D; **Taxon:** scientificName: *Haploniscus bulbosus* Henseler, Knauber & Riehl; kingdom: Animalia; phylum: Arthropoda; class: Malacostraca; order: Isopoda; family: Haploniscidae; genus: Haploniscus; specificEpithet: bulbosus; scientificNameAuthorship: Henseler, Knauber & Riehl; nomenclaturalCode: ICZN; **Location:** higherGeography: Pacific Ocean; locality: North-west Pacific Ocean, KuramBio station SO223-10-09; verbatimDepth: 5264-5266 m; verbatimLatitude: 41°11'16.188''N; verbatimLongitude: 150°5'36.888''E; decimalLatitude: 41.18783; decimalLongitude: 150.09358; **Identification:** identifiedBy: Henry Knauber; **Event:** samplingProtocol: Benthos trawl, Camera-Epibenthic Sledge, sieved through 0.3 mm mesh | Riehl T, Brenke N, Brix S, Driskell A, Kaiser S, Brandt A (2014) Field and laboratory methods for DNA studies on deep-sea isopod crustaceans. Polish Polar Research 35: 205–226. https://doi.org/10.2478/popore−2014−0018 | Brandt A, Malyutina MV (2012) The German-Russian deep-sea expedition KuramBio (Kurile Kamchatka Biodiversity Study) : to the Kurile Kamchatka Trench and abyssal plain on board of the R/V SONNE, 223rd Expedition, 21 July - 7 September 2012. University of Hamburg; Biozentrum Grindel & Zoologisches Museum Hamburg, Hamburg. Cruise Report, 100pp. Available from: http://edok01.tib.uni-hannover.de/edoks/e01fb13/741102293.pdf.; eventDate: 26.08.2012; habitat: abyssal sediment; fieldNumber: SO223-10-09; **Record Level:** institutionCode: SMF; basisOfRecord: PreservedSpecimen
- **Type status:** Other material. **Occurrence:** catalogNumber: SMF 62956; recordNumber: KBHap018; individualCount: 1; sex: female; lifeStage: adult; preparations: whole animal (ETOH); occurrenceID: 3E8E3D3F-9F71-52C2-A8BD-F50CE7147046; **Taxon:** scientificName: *Haploniscus bulbosus* Henseler, Knauber & Riehl; kingdom: Animalia; phylum: Arthropoda; class: Malacostraca; order: Isopoda; family: Haploniscidae; genus: Haploniscus; specificEpithet: bulbosus; scientificNameAuthorship: Henseler, Knauber & Riehl; nomenclaturalCode: ICZN; **Location:** higherGeography: Pacific Ocean; locality: North-west Pacific Ocean, KuramBio station SO223-09-09; verbatimDepth: 5399-5421 m; verbatimLatitude: 40°34'22.8''N; verbatimLongitude: 150°59'54.888''E; decimalLatitude: 40.573; decimalLongitude: 150.99858; **Identification:** identifiedBy: Henry Knauber; **Event:** samplingProtocol: Benthos trawl, Camera-Epibenthic Sledge, sieved through 0.3 mm mesh | Riehl T, Brenke N, Brix S, Driskell A, Kaiser S, Brandt A (2014) Field and laboratory methods for DNA studies on deep-sea isopod crustaceans. Polish Polar Research 35: 205–226. https://doi.org/10.2478/popore−2014−0018 | Brandt A, Malyutina MV (2012) The German-Russian deep-sea expedition KuramBio (Kurile Kamchatka Biodiversity Study) : to the Kurile Kamchatka Trench and abyssal plain on board of the R/V SONNE, 223rd Expedition, 21 July - 7 September 2012. University of Hamburg; Biozentrum Grindel & Zoologisches Museum Hamburg, Hamburg. Cruise Report, 100pp. Available from: http://edok01.tib.uni-hannover.de/edoks/e01fb13/741102293.pdf.; eventDate: 23.08.2012; habitat: abyssal sediment; fieldNumber: SO223-09-09; **Record Level:** institutionCode: SMF; basisOfRecord: PreservedSpecimen
- **Type status:** Other material. **Occurrence:** catalogNumber: SMF 62957; recordNumber: KBHap019; individualCount: 1; sex: female; lifeStage: ovigerous; preparations: whole animal (ETOH); occurrenceID: D2BC8647-7AAB-5E93-A45F-D9F73A52E45C; **Taxon:** scientificName: *Haploniscus bulbosus* Henseler, Knauber & Riehl; kingdom: Animalia; phylum: Arthropoda; class: Malacostraca; order: Isopoda; family: Haploniscidae; genus: Haploniscus; specificEpithet: bulbosus; scientificNameAuthorship: Henseler, Knauber & Riehl; nomenclaturalCode: ICZN; **Location:** higherGeography: Pacific Ocean; locality: North-west Pacific Ocean, KuramBio station SO223-09-09; verbatimDepth: 5399-5421 m; verbatimLatitude: 40°34'22.8''N; verbatimLongitude: 150°59'54.888''E; decimalLatitude: 40.573; decimalLongitude: 150.99858; **Identification:** identifiedBy: Henry Knauber; **Event:** samplingProtocol: Benthos trawl, Camera-Epibenthic Sledge, sieved through 0.3 mm mesh | Riehl T, Brenke N, Brix S, Driskell A, Kaiser S, Brandt A (2014) Field and laboratory methods for DNA studies on deep-sea isopod crustaceans. Polish Polar Research 35: 205–226. https://doi.org/10.2478/popore−2014−0018 | Brandt A, Malyutina MV (2012) The German-Russian deep-sea expedition KuramBio (Kurile Kamchatka Biodiversity Study) : to the Kurile Kamchatka Trench and abyssal plain on board of the R/V SONNE, 223rd Expedition, 21 July - 7 September 2012. University of Hamburg; Biozentrum Grindel & Zoologisches Museum Hamburg, Hamburg. Cruise Report, 100pp. Available from: http://edok01.tib.uni-hannover.de/edoks/e01fb13/741102293.pdf.; eventDate: 23.08.2012; habitat: abyssal sediment; fieldNumber: SO223-09-09; **Record Level:** institutionCode: SMF; basisOfRecord: PreservedSpecimen
- **Type status:** Other material. **Occurrence:** catalogNumber: SMF 62958; recordNumber: KBHap020; individualCount: 1; sex: female; lifeStage: adult; preparations: whole animal (ETOH); occurrenceID: 774F1232-C5C3-5F72-8239-2666504C745A; **Taxon:** scientificName: *Haploniscus bulbosus* Henseler, Knauber & Riehl; kingdom: Animalia; phylum: Arthropoda; class: Malacostraca; order: Isopoda; family: Haploniscidae; genus: Haploniscus; specificEpithet: bulbosus; scientificNameAuthorship: Henseler, Knauber & Riehl; nomenclaturalCode: ICZN; **Location:** higherGeography: Pacific Ocean; locality: North-west Pacific Ocean, KuramBio station SO223-09-09; verbatimDepth: 5399-5421 m; verbatimLatitude: 40°34'22.8''N; verbatimLongitude: 150°59'54.888''E; decimalLatitude: 40.573; decimalLongitude: 150.99858; **Identification:** identifiedBy: Henry Knauber; **Event:** samplingProtocol: Benthos trawl, Camera-Epibenthic Sledge, sieved through 0.3 mm mesh | Riehl T, Brenke N, Brix S, Driskell A, Kaiser S, Brandt A (2014) Field and laboratory methods for DNA studies on deep-sea isopod crustaceans. Polish Polar Research 35: 205–226. https://doi.org/10.2478/popore−2014−0018 | Brandt A, Malyutina MV (2012) The German-Russian deep-sea expedition KuramBio (Kurile Kamchatka Biodiversity Study) : to the Kurile Kamchatka Trench and abyssal plain on board of the R/V SONNE, 223rd Expedition, 21 July 21 - 7 September 2012. University of Hamburg; Biozentrum Grindel & Zoologisches Museum Hamburg, Hamburg. Cruise Report, 100pp. Available from: http://edok01.tib.uni-hannover.de/edoks/e01fb13/741102293.pdf.; eventDate: 23.08.2012; habitat: abyssal sediment; fieldNumber: SO223-09-09; **Record Level:** institutionCode: SMF; basisOfRecord: PreservedSpecimen
- **Type status:** Other material. **Occurrence:** catalogNumber: SMF 62959; recordNumber: KBHap021; individualCount: 1; sex: female; lifeStage: adult; preparations: whole animal (ETOH); occurrenceID: E4C6FCDC-B423-5423-ABE7-2452ED74FC87; **Taxon:** scientificName: *Haploniscus bulbosus* Henseler, Knauber & Riehl; kingdom: Animalia; phylum: Arthropoda; class: Malacostraca; order: Isopoda; family: Haploniscidae; genus: Haploniscus; specificEpithet: bulbosus; scientificNameAuthorship: Henseler, Knauber & Riehl; nomenclaturalCode: ICZN; **Location:** higherGeography: Pacific Ocean; locality: North-west Pacific Ocean, KuramBio station SO223-09-09; verbatimDepth: 5399-5421 m; verbatimLatitude: 40°34'22.8''N; verbatimLongitude: 150°59'54.888''E; decimalLatitude: 40.573; decimalLongitude: 150.99858; **Identification:** identifiedBy: Henry Knauber; **Event:** samplingProtocol: Benthos trawl, Camera-Epibenthic Sledge, sieved through 0.3 mm mesh | Riehl T, Brenke N, Brix S, Driskell A, Kaiser S, Brandt A (2014) Field and laboratory methods for DNA studies on deep-sea isopod crustaceans. Polish Polar Research 35: 205–226. https://doi.org/10.2478/popore−2014−0018 | Brandt A, Malyutina MV (2012) The German-Russian deep-sea expedition KuramBio (Kurile Kamchatka Biodiversity Study) : to the Kurile Kamchatka Trench and abyssal plain on board of the R/V SONNE, 223rd Expedition, 21 July - 7 September 2012. University of Hamburg; Biozentrum Grindel & Zoologisches Museum Hamburg, Hamburg. Cruise Report, 100pp. Available from: http://edok01.tib.uni-hannover.de/edoks/e01fb13/741102293.pdf.; eventDate: 23.08.2012; habitat: abyssal sediment; fieldNumber: SO223-09-09; **Record Level:** institutionCode: SMF; basisOfRecord: PreservedSpecimen
- **Type status:** Other material. **Occurrence:** catalogNumber: SMF 62960; recordNumber: KBHap022; individualCount: 1; sex: male; lifeStage: adult; preparations: whole animal (ETOH); occurrenceID: 9AB51605-AF2A-52AE-AB02-D160266E20D6; **Taxon:** scientificName: *Haploniscus bulbosus* Henseler, Knauber & Riehl; kingdom: Animalia; phylum: Arthropoda; class: Malacostraca; order: Isopoda; family: Haploniscidae; genus: Haploniscus; specificEpithet: bulbosus; scientificNameAuthorship: Henseler, Knauber & Riehl; nomenclaturalCode: ICZN; **Location:** higherGeography: Pacific Ocean; locality: North-west Pacific Ocean, KuramBio station SO223-09-09; verbatimDepth: 5399-5421 m; verbatimLatitude: 40°34'22.8''N; verbatimLongitude: 150°59'54.888''E; decimalLatitude: 40.573; decimalLongitude: 150.99858; **Identification:** identifiedBy: Henry Knauber; **Event:** samplingProtocol: Benthos trawl, Camera-Epibenthic Sledge, sieved through 0.3 mm mesh | Riehl T, Brenke N, Brix S, Driskell A, Kaiser S, Brandt A (2014) Field and laboratory methods for DNA studies on deep-sea isopod crustaceans. Polish Polar Research 35: 205–226. https://doi.org/10.2478/popore−2014−0018 | Brandt A, Malyutina MV (2012) The German-Russian deep-sea expedition KuramBio (Kurile Kamchatka Biodiversity Study) : to the Kurile Kamchatka Trench and abyssal plain on board of the R/V SONNE, 223rd Expedition, 21 July - 7 September 2012. University of Hamburg; Biozentrum Grindel & Zoologisches Museum Hamburg, Hamburg. Cruise Report, 100pp. Available from: http://edok01.tib.uni-hannover.de/edoks/e01fb13/741102293.pdf.; eventDate: 23.08.2012; habitat: abyssal sediment; fieldNumber: SO223-09-09; **Record Level:** institutionCode: SMF; basisOfRecord: PreservedSpecimen
- **Type status:** Other material. **Occurrence:** catalogNumber: SMF 62961; recordNumber: KBHap023; individualCount: 1; sex: male; lifeStage: juvenile; preparations: whole animal (ETOH); occurrenceID: 64FB98CA-0ED0-5700-A563-C215C1A9DAE6; **Taxon:** scientificName: *Haploniscus bulbosus* Henseler, Knauber & Riehl; kingdom: Animalia; phylum: Arthropoda; class: Malacostraca; order: Isopoda; family: Haploniscidae; genus: Haploniscus; specificEpithet: bulbosus; scientificNameAuthorship: Henseler, Knauber & Riehl; nomenclaturalCode: ICZN; **Location:** higherGeography: Pacific Ocean; locality: North-west Pacific Ocean, KuramBio station SO223-09-09; verbatimDepth: 5399-5421 m; verbatimLatitude: 40°34'22.8''N; verbatimLongitude: 150°59'54.888''E; decimalLatitude: 40.573; decimalLongitude: 150.99858; **Identification:** identifiedBy: Henry Knauber; **Event:** samplingProtocol: Benthos trawl, Camera-Epibenthic Sledge, sieved through 0.3 mm mesh | Riehl T, Brenke N, Brix S, Driskell A, Kaiser S, Brandt A (2014) Field and laboratory methods for DNA studies on deep-sea isopod crustaceans. Polish Polar Research 35: 205–226. https://doi.org/10.2478/popore−2014−0018 | Brandt A, Malyutina MV (2012) The German-Russian deep-sea expedition KuramBio (Kurile Kamchatka Biodiversity Study) : to the Kurile Kamchatka Trench and abyssal plain on board of the R/V SONNE, 223rd Expedition, 21 July - 7 September 2012. University of Hamburg; Biozentrum Grindel & Zoologisches Museum Hamburg, Hamburg. Cruise Report, 100pp. Available from: http://edok01.tib.uni-hannover.de/edoks/e01fb13/741102293.pdf.; eventDate: 23.08.2012; habitat: abyssal sediment; fieldNumber: SO223-09-09; **Record Level:** institutionCode: SMF; basisOfRecord: PreservedSpecimen
- **Type status:** Other material. **Occurrence:** catalogNumber: SMF 62962; recordNumber: KBHap024; individualCount: 1; sex: female; lifeStage: adult; preparations: whole animal (ETOH); occurrenceID: 6D727CF7-1F5E-5FDD-92DA-D69DC959FA7A; **Taxon:** scientificName: *Haploniscus bulbosus* Henseler, Knauber & Riehl; kingdom: Animalia; phylum: Arthropoda; class: Malacostraca; order: Isopoda; family: Haploniscidae; genus: Haploniscus; specificEpithet: bulbosus; scientificNameAuthorship: Henseler, Knauber & Riehl; nomenclaturalCode: ICZN; **Location:** higherGeography: Pacific Ocean; locality: North-west Pacific Ocean, KuramBio station SO223-09-09; verbatimDepth: 5399-5421 m; verbatimLatitude: 40°34'22.8''N; verbatimLongitude: 150°59'54.888''E; decimalLatitude: 40.573; decimalLongitude: 150.99858; **Identification:** identifiedBy: Henry Knauber; **Event:** samplingProtocol: Benthos trawl, Camera-Epibenthic Sledge, sieved through 0.3 mm mesh | Riehl T, Brenke N, Brix S, Driskell A, Kaiser S, Brandt A (2014) Field and laboratory methods for DNA studies on deep-sea isopod crustaceans. Polish Polar Research 35: 205–226. https://doi.org/10.2478/popore−2014−0018 | Brandt A, Malyutina MV (2012) The German-Russian deep-sea expedition KuramBio (Kurile Kamchatka Biodiversity Study) : to the Kurile Kamchatka Trench and abyssal plain on board of the R/V SONNE, 223rd Expedition, 21 July - 7 September 2012. University of Hamburg; Biozentrum Grindel & Zoologisches Museum Hamburg, Hamburg. Cruise Report, 100pp. Available from: http://edok01.tib.uni-hannover.de/edoks/e01fb13/741102293.pdf.; eventDate: 23.08.2012; habitat: abyssal sediment; fieldNumber: SO223-09-09; **Record Level:** institutionCode: SMF; basisOfRecord: PreservedSpecimen
- **Type status:** Other material. **Occurrence:** catalogNumber: SMF 62963; recordNumber: KBHap145; individualCount: 1; sex: female; lifeStage: ovigerous; preparations: anterior body only (ETOH); occurrenceID: 793E20C4-A245-56EB-AB40-F939E117F518; **Taxon:** scientificName: *Haploniscus bulbosus* Henseler, Knauber & Riehl; kingdom: Animalia; phylum: Arthropoda; class: Malacostraca; order: Isopoda; family: Haploniscidae; genus: Haploniscus; specificEpithet: bulbosus; scientificNameAuthorship: Henseler, Knauber & Riehl; nomenclaturalCode: ICZN; **Location:** higherGeography: Pacific Ocean; locality: North-west Pacific Ocean, KuramBio station SO223-09-09; verbatimDepth: 5399-5421 m; verbatimLatitude: 40°34'22.8''N; verbatimLongitude: 150°59'54.888''E; decimalLatitude: 40.573; decimalLongitude: 150.99858; **Identification:** identifiedBy: Henry Knauber; **Event:** samplingProtocol: Benthos trawl, Camera-Epibenthic Sledge, sieved through 0.3 mm mesh | Riehl T, Brenke N, Brix S, Driskell A, Kaiser S, Brandt A (2014) Field and laboratory methods for DNA studies on deep-sea isopod crustaceans. Polish Polar Research 35: 205–226. https://doi.org/10.2478/popore−2014−0018 | Brandt A, Malyutina MV (2012) The German-Russian deep-sea expedition KuramBio (Kurile Kamchatka Biodiversity Study) : to the Kurile Kamchatka Trench and abyssal plain on board of the R/V SONNE, 223rd Expedition, 21 July - 7 September 2012. University of Hamburg; Biozentrum Grindel & Zoologisches Museum Hamburg, Hamburg. Cruise Report, 100pp. Available from: http://edok01.tib.uni-hannover.de/edoks/e01fb13/741102293.pdf.; eventDate: 23.08.2012; habitat: abyssal sediment; fieldNumber: SO223-09-09; **Record Level:** institutionCode: SMF; basisOfRecord: PreservedSpecimen
- **Type status:** Other material. **Occurrence:** catalogNumber: SMF 62964; recordNumber: KBHap146; individualCount: 1; sex: indet.; lifeStage: indet.; preparations: anterior body only (ETOH); occurrenceID: AF33497D-95CB-5389-9AD6-958DFE47003C; **Taxon:** scientificName: *Haploniscus bulbosus* Henseler, Knauber & Riehl; kingdom: Animalia; phylum: Arthropoda; class: Malacostraca; order: Isopoda; family: Haploniscidae; genus: Haploniscus; specificEpithet: bulbosus; scientificNameAuthorship: Henseler, Knauber & Riehl; nomenclaturalCode: ICZN; **Location:** higherGeography: Pacific Ocean; locality: North-west Pacific Ocean, KuramBio station SO223-09-09; verbatimDepth: 5399-5421 m; verbatimLatitude: 40°34'22.8''N; verbatimLongitude: 150°59'54.888''E; decimalLatitude: 40.573; decimalLongitude: 150.99858; **Identification:** identifiedBy: Henry Knauber; **Event:** samplingProtocol: Benthos trawl, Camera-Epibenthic Sledge, sieved through 0.3 mm mesh | Riehl T, Brenke N, Brix S, Driskell A, Kaiser S, Brandt A (2014) Field and laboratory methods for DNA studies on deep-sea isopod crustaceans. Polish Polar Research 35: 205–226. https://doi.org/10.2478/popore−2014−0018 | Brandt A, Malyutina MV (2012) The German-Russian deep-sea expedition KuramBio (Kurile Kamchatka Biodiversity Study) : to the Kurile Kamchatka Trench and abyssal plain on board of the R/V SONNE, 223rd Expedition, 21 July - 7 September 2012. University of Hamburg; Biozentrum Grindel & Zoologisches Museum Hamburg, Hamburg. Cruise Report, 100pp. Available from: http://edok01.tib.uni-hannover.de/edoks/e01fb13/741102293.pdf.; eventDate: 23.08.2012; habitat: abyssal sediment; fieldNumber: SO223-09-09; **Record Level:** institutionCode: SMF; basisOfRecord: PreservedSpecimen
- **Type status:** Other material. **Occurrence:** catalogNumber: SMF 62965; recordNumber: KBHap147; individualCount: 1; sex: indet.; lifeStage: indet.; preparations: anterior body only (ETOH); occurrenceID: 5F294075-2B30-5D14-87D2-D075E6E1A7C4; **Taxon:** scientificName: *Haploniscus bulbosus* Henseler, Knauber & Riehl; kingdom: Animalia; phylum: Arthropoda; class: Malacostraca; order: Isopoda; family: Haploniscidae; genus: Haploniscus; specificEpithet: bulbosus; scientificNameAuthorship: Henseler, Knauber & Riehl; nomenclaturalCode: ICZN; **Location:** higherGeography: Pacific Ocean; locality: North-west Pacific Ocean, KuramBio station SO223-09-09; verbatimDepth: 5399-5421 m; verbatimLatitude: 40°34'22.8''N; verbatimLongitude: 150°59'54.888''E; decimalLatitude: 40.573; decimalLongitude: 150.99858; **Identification:** identifiedBy: Henry Knauber; **Event:** samplingProtocol: Benthos trawl, Camera-Epibenthic Sledge, sieved through 0.3 mm mesh | Riehl T, Brenke N, Brix S, Driskell A, Kaiser S, Brandt A (2014) Field and laboratory methods for DNA studies on deep-sea isopod crustaceans. Polish Polar Research 35: 205–226. https://doi.org/10.2478/popore−2014−0018 | Brandt A, Malyutina MV (2012) The German-Russian deep-sea expedition KuramBio (Kurile Kamchatka Biodiversity Study) : to the Kurile Kamchatka Trench and abyssal plain on board of the R/V SONNE, 223rd Expedition, 21 July - 7 September 2012. University of Hamburg; Biozentrum Grindel & Zoologisches Museum Hamburg, Hamburg. Cruise Report, 100pp. Available from: http://edok01.tib.uni-hannover.de/edoks/e01fb13/741102293.pdf.; eventDate: 23.08.2012; habitat: abyssal sediment; fieldNumber: SO223-09-09; **Record Level:** institutionCode: SMF; basisOfRecord: PreservedSpecimen

#### Description

**Description of male**

**Figure 30.**
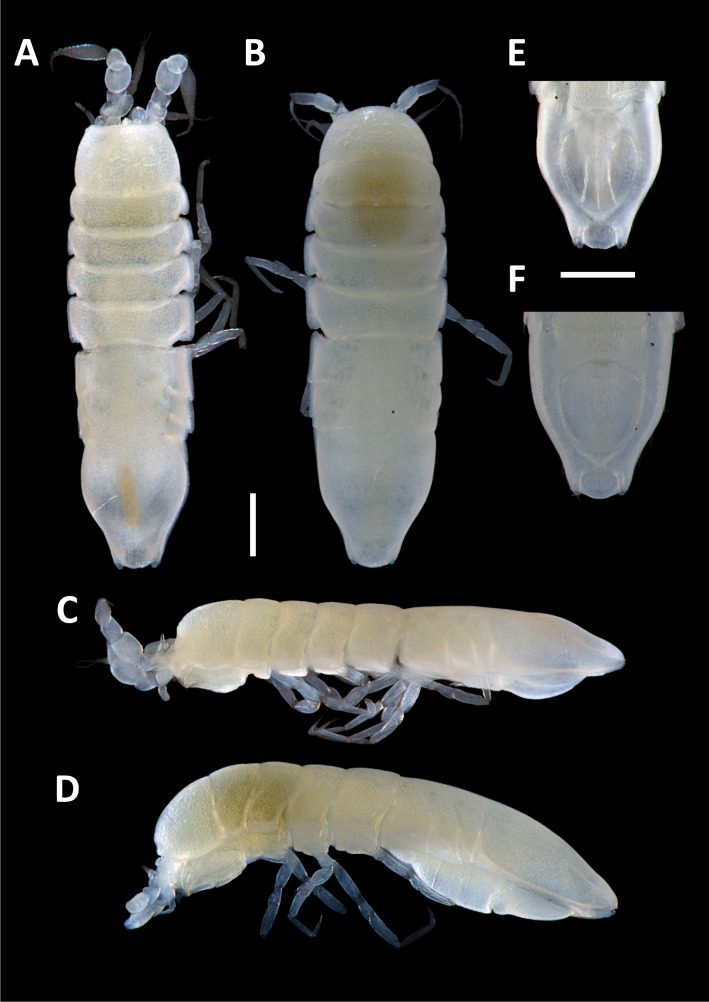
*Haploniscus bulbosus* Henseler, Knauber & Riehl, **sp. nov.** macrophotographs. **A, C, E** male holotype (SMF 62946); **B, D, F** female paratype (SMF 62953). Scale bars: 1.8 mm.

**Figure 31.**
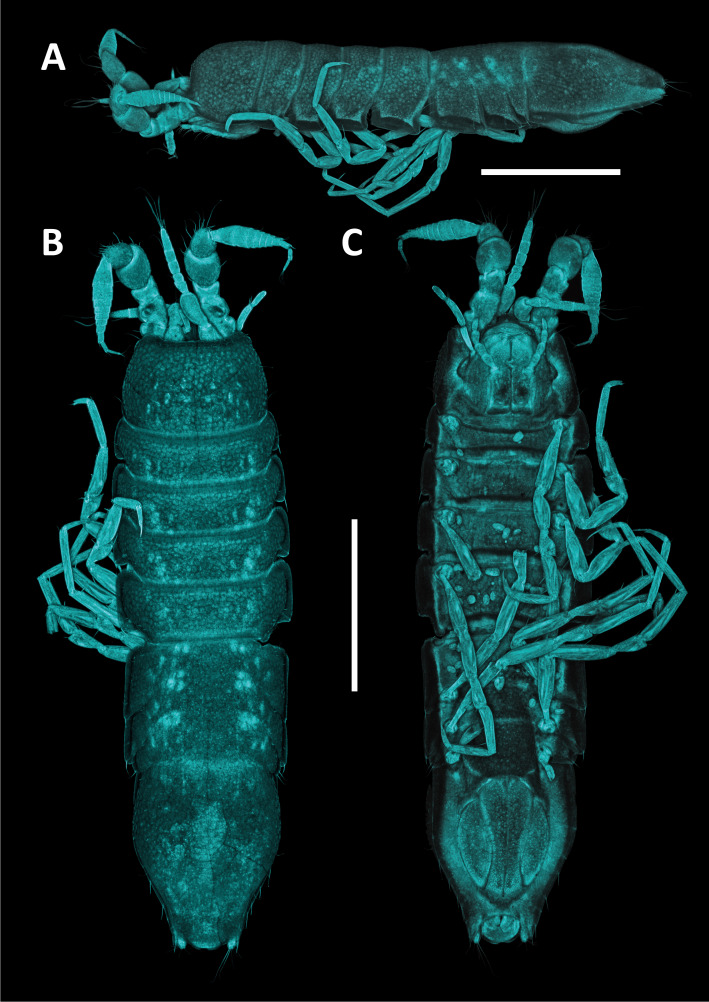
*Haploniscus bulbosus* Henseler, Knauber & Riehl, **sp. nov.** male holotype (SMF 62946) habitus. **A** Lateral view; **B** Dorsal view; **C** Ventral view. Scale bars = 0.4 mm. Confocal laser-scanning micrographs with pseudo-colour.

**Figure 32.**
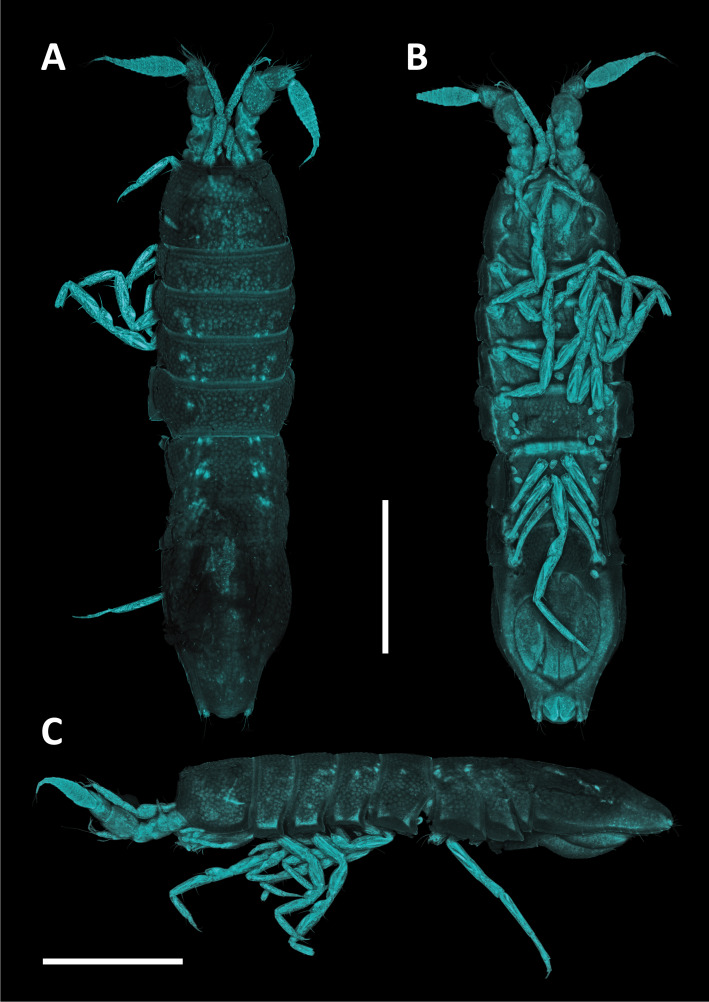
*Haploniscus bulbosus* Henseler, Knauber & Riehl, **sp. nov.** male paratype (SMF 62947) habitus. **A** Dorsal view; **B** Ventral view; **C** Lateral view. Scale bars = 0.4 mm. Confocal laser-scanning micrographs with pseudo-colour.

**Figure 33.**
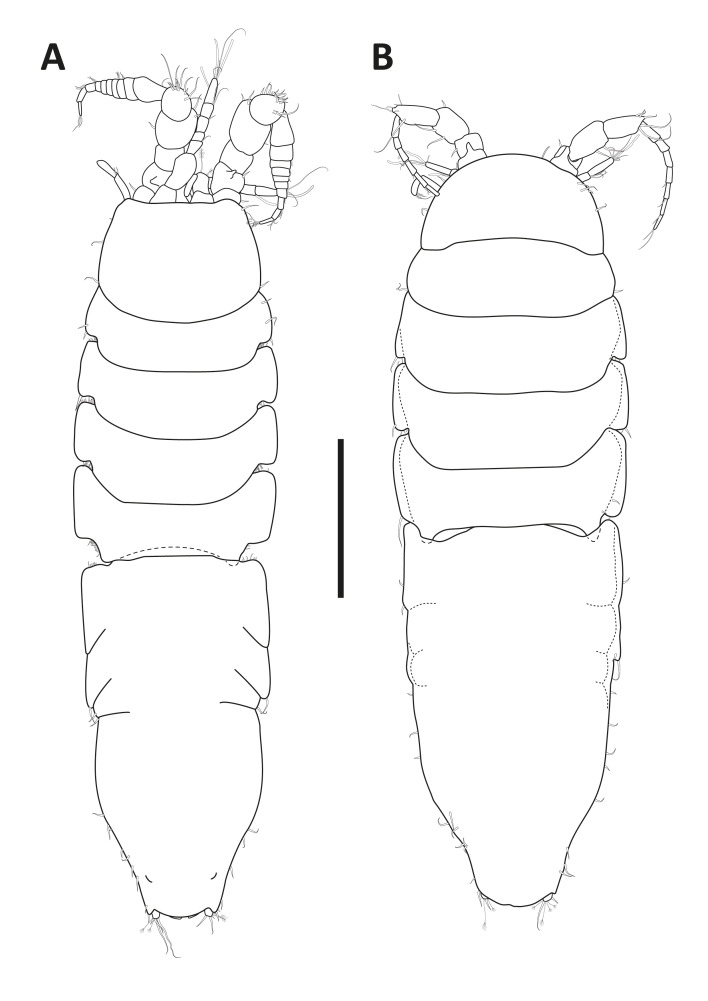
*Haploniscus bulbosus* Henseler, Knauber & Riehl, **sp. nov.** dorsal habitus. **A** Male holotype (SMF 62946); **B** Female paratype (SMF 62953). Scale bar = 0.4 mm.

**Figure 34.**
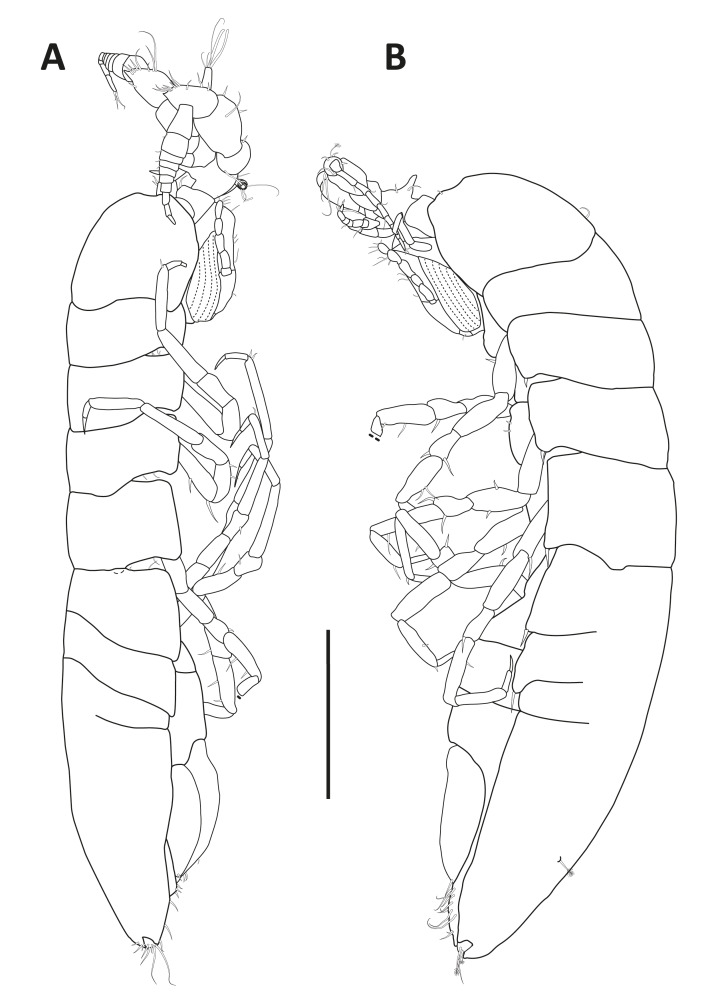
*Haploniscus bulbosus* Henseler, Knauber & Riehl, **sp. nov.** lateral habitus. **A** Male holotype (SMF 62946); **B** Female paratype (SMF 62953). Scale bar = 0.4 mm.

**Body** (Fig. 30A, C, E; Fig. 31; Fig. 32; Fig. 33A; Fig. 34A) length 1.8 mm, 3.5–3.6 width; elongated, cylindrical; non-conglobating; anterior body length (Ceph–Prn4) 0.97 posterior body length (Prn5–Plt); lateral margin interrupted between anterior and posterior body, otherwise continuous; tergite surfaces tuberculate, posterior body medially with less visible ornamentation.

**Ceph** (Figs 30, 31, 32, 33, 34) length 0.67 width, 0.16 body length, width 0.86 body width; trapezoidal, tergite surface tuberculate; anterolateral angles rounded, not projecting; frontal margin concave, width 0.63 Ceph width.

**Prn1** (Figs 31, 33, 34) posterior tergite margin through Prn5 anterior tergite margin smooth, setose. **Prn4** (Figs 31, 33, 34 posterolateral angle non-projecting, rounded, Prn4 lateral margin length 1.2 **Prn5** (Figs 31, 33, 34) lateral margin length; Prn5 anterolateral angle not projecting, rounded; **Prn7** (Figs 31, 33, 34) shape similar to Prn6; Prn5 and Prn6 as well as Prn7 and Plt tergites medially conjoined, segment borders not expressed; Prn1-7 posterolateral margin with single associated seta.

**Plt** (Figs 31, 33, 34) length 0.32 body length, lateral margin anteriorly convex, caudally concave, posterior margin rounded, convex; tergite surface tuberculate, less distinct medially than in remaining body; with a pair of tubercules; posterolateral processes minute, less than 0.10 Plt length, straight, tapering to blunt point, orientated posteriorly.

**Figure 35.**
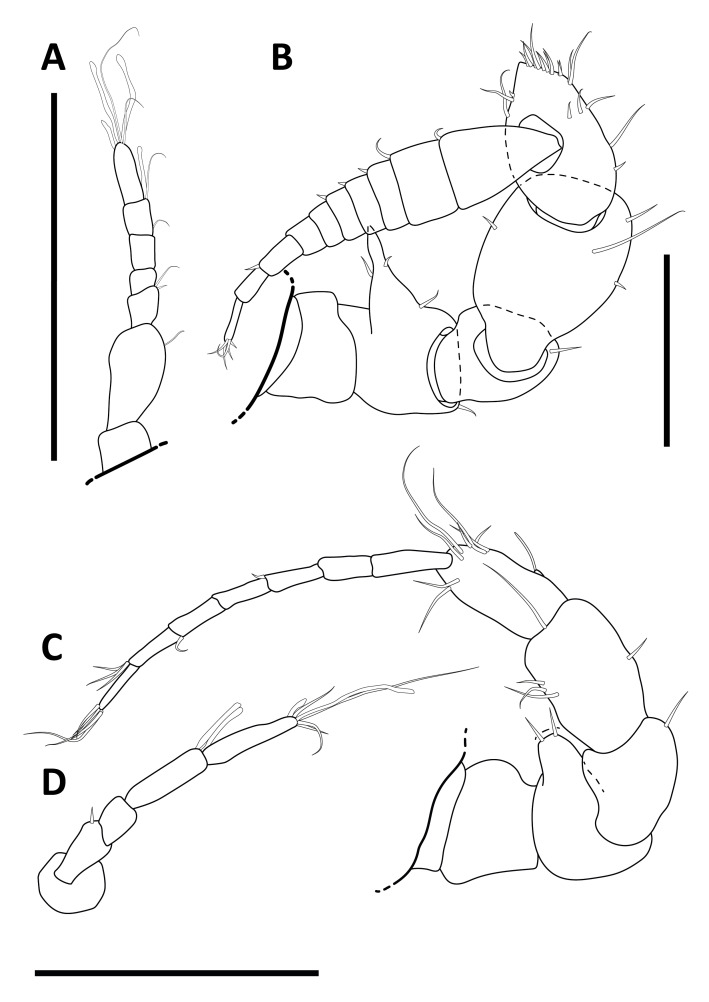
*Haploniscus bulbosus* Henseler, Knauber & Riehl, **sp. nov.** antennae of male holotype (SMF 62946; in situ) and female paratype (SMF 62953; in situ). **A** Male holotype antennula; **B** Male holotype antenna 2; **C** Female paratype antenna 2; **D** Female paratype antennula. Scale bars: 0.4 mm (A), 0.2 mm (B-D).

**A1** (Fig. 35A) length 0.20 body length; flagellum with 4 art. **A2** (Fig. 35B) length 0.36 body length; article 3 length subsimilar width, dorsal projection acutely elongated, orientated dorsally, projection length 0.67 article 3 length; article 5 length subsimilar width, ovoid shape ("inflated"), bulbous; art6 ovoid with distal subtriangular projection; flagellum with 10 art.

**Figure 36.**
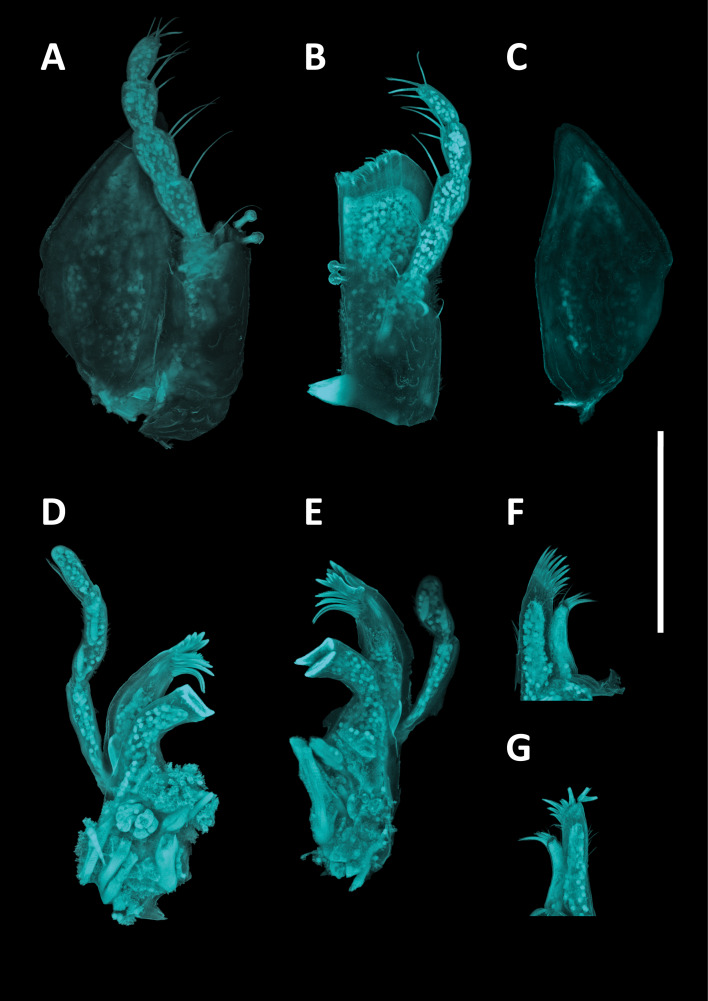
*Haploniscus bulbosus* Henseler, Knauber & Riehl, **sp. nov.** male paratype (SMF 62947) mouthparts. **A** Right maxilliped (dorsal); **B** Left maxilliped (dorsal; without epipod); **C** Left maxilliped (epipod); **D** Right mandible (ventral); **E** Left mandible (ventral); **F** Right maxilla; **G** Left maxilla. Scale bar = 0.15 mm. Confocal laser-scanning micrographs with pseudo-colour.

**Md** (Fig. 36D, E) incisor with five cusps, left Md lacinia mobilis with five cusps, palp length subequal mandible length, palp article 2 curved. **Mxp** (Fig. 36A-C) with two coupling hooks, respectively.

**Figure 37.**
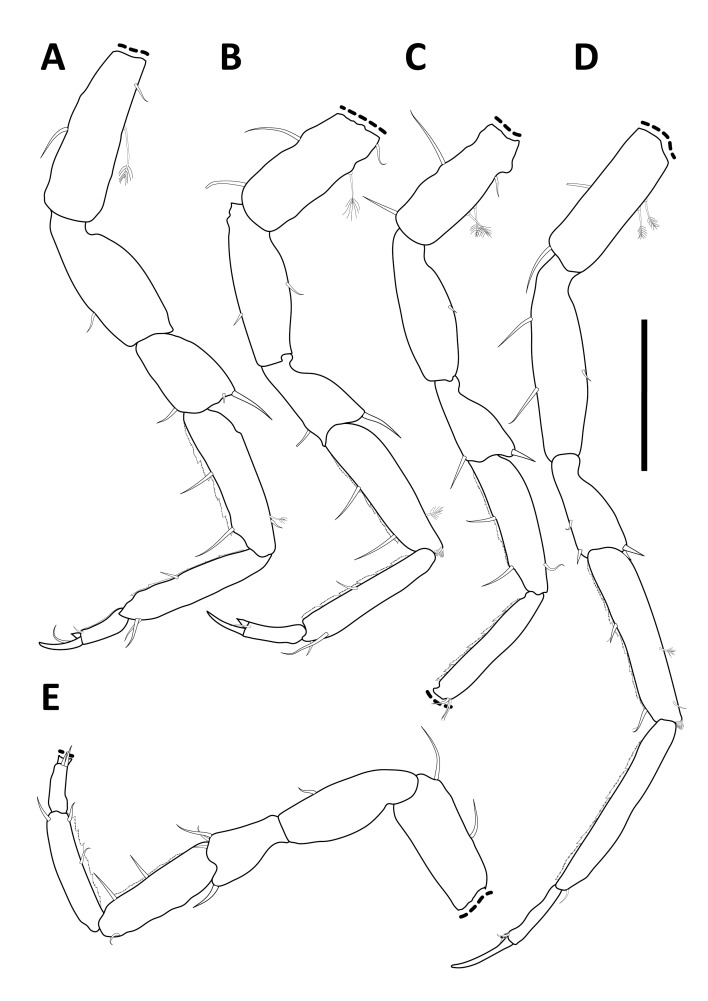
*Haploniscus bulbosus* Henseler Knauber & Riehl, **sp. nov.** male paratype (SMF 62947) pereopods. **A** Pereopod 2; **B** Pereopod 3; **C** Pereopod 4; **D** Pereopod 6; **E** Pereopod 1. Scale bar = 0.2 mm.

**P1** (Fig. 37E) length 0.46 body length. P2 (Fig. 37) length 0.61 body length. **P3** (Fig. 37B) length 0.60 body length. **P6** (Fig. 37D) length 0.69 body length. P lengths gradually increasing from P1 to P6, P7 shorter than P6. P carpi, propodi and dactyli inner margins fringed by comb-like scale rows.

**Figure 38.**
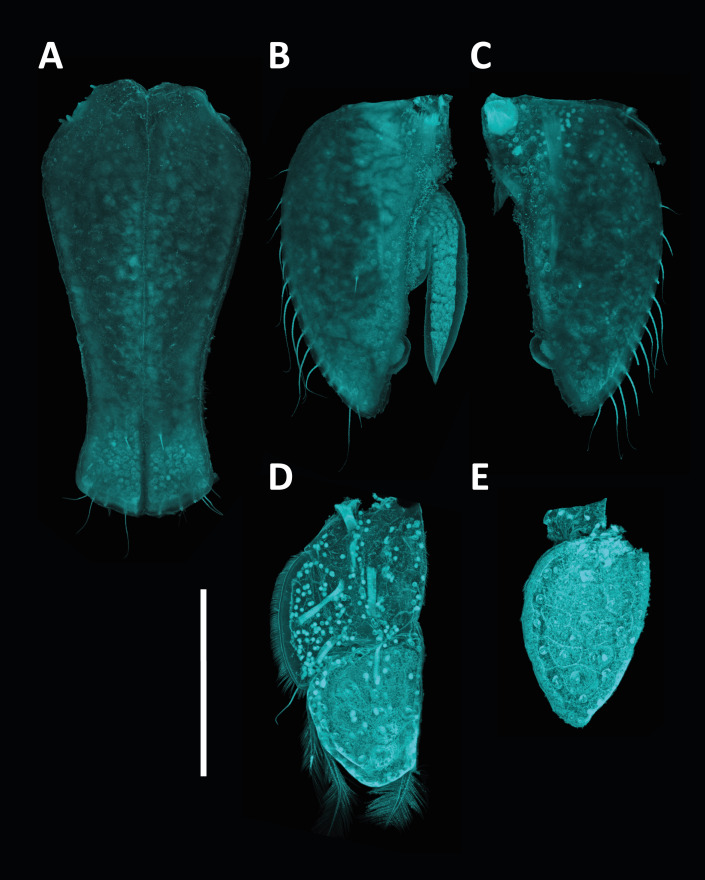
*Haploniscus bulbosus* Henseler, Knauber & Riehl, **sp. nov.** male paratype (SMF 62947) pleopods. **A** Pleopod 1; **B** Right pleopod 2; **C** Left pleopod 2; **D** Pleopod 3; **E** Pleopod 5. Scale bar = 0.15 mm. Confocal laser-scanning micrographs with pseudo-colour.

**Plp1** (Fig. 38A) proximal half trapezoid, distal half subrectangular; lateral lobes indistinct, fused with medial lobes; medial lobes quadrangle, adjoining at apex. **Plp2** (Fig. 38B, C) protopod elongate, suboval, distal margin with continuous row of elongated simple setae, lateral margin with 1–3 short simple setae; endopod stylet short, about half as long as protopod. **Urp** (Figs 31, 33, 34) cylindrical, short, projecting caudally as far as posterior Plt apex; socket position recessed in sternal fold laterally to anal valve.

**Description of the adult female paratype (SMF 62953; where different from male)**

**Figure 39.**
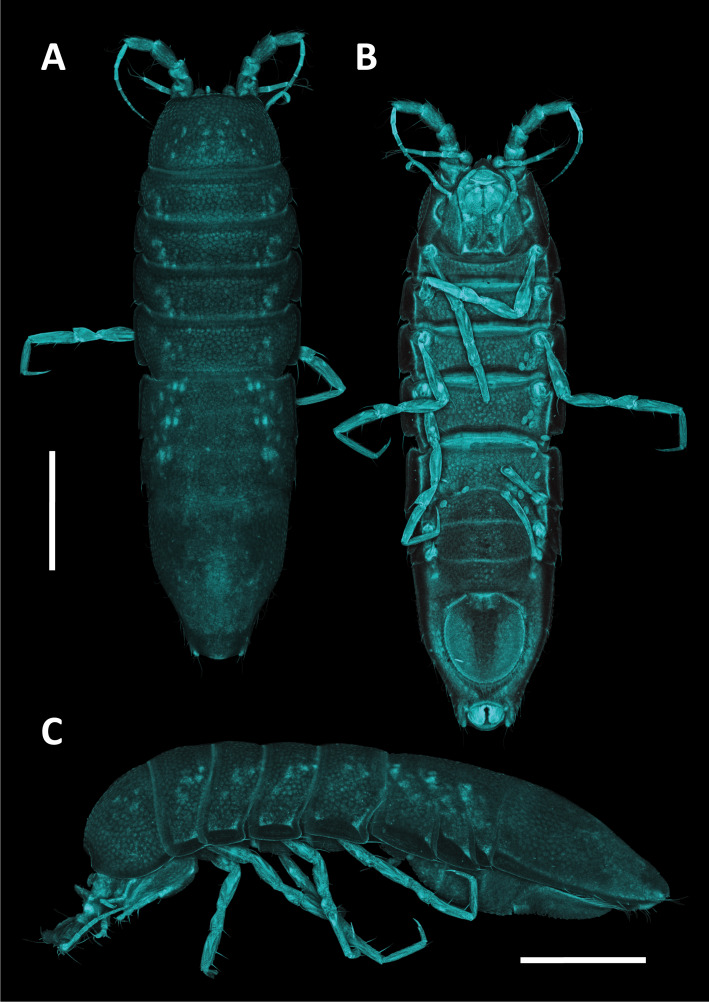
*Haploniscus bulbosus* Henseler, Knauber & Riehl, **sp. nov.** female paratype (SMF 62953) habitus. **A** Dorsal view; **B** Ventral view; **C** Lateral view. Scale bars = 0.4 mm. Confocal laser-scanning micrographs with pseudo-colour.

**Body** (Fig. 30B, D, F; Fig. 39; Fig. 33B, Fig. 34B) length 2.0 mm, 3.4 width; anterior body length (Ceph–Prn4) 0.99 posterior body length (Prn 5–Plt). **Ceph** (Fig. 39; Fig. 33B; Fig. 34B) length 0.58 width, 0.14 body length, width 0.80 body width; width 0.58 Ceph width. **Prn4** (Fig. 39; Fig. 33B; Fig. 34B) lateral margin length 0.78 Prn5 lateral margin length. **Plt** (Fig. 39; Fig. 33B; Fig. 34B) length 0.30 body length.

**A1** (Fig. 35D) flagellum with 3 art. **A2** (Fig. 35C) length 0.36 body length; projection length 0.67 art3 length; art5-6 cylindrical; flagellum with 7 art. **Op** (Fig. 30F; Fig. 39B) length 1.1 width, 0.53 Plt length; anteriorly with median bulge, otherwise circular, surface smooth.

##### Type material

Holotype (SMF 62946), adult male (stage V), 1.8 mm. Paratypes (SMF 62947), adult male (stage V), 1.8 mm and adult female (stage IV), 2.0 mm (SMF 62953).

##### Material examined

Holotype (SMF 62946) and paratypes (SMF 62947, SMF 62953) examined and measured, plus 17 additional specimens from the type locality and another close station on the abyssal plains southeast of the Kuril-Kamchatka Trench (SMF 62948–62952, 62954–62965).

##### Type locality

Northwest Pacific, abyssal plain of the greater Kuril Kamchatka Trench region, KuramBio expedition, St. SO223-10-09, R/V SONNE, EBS, 5264–5266 m, 41°11.37'N – 41°11.17'N, 150°05.63'E – 150°05.60'E.

##### Sexual dimorphism

Female differs in the following characters: larger body size, A1 with three instead of four flagellar art, shape of A2 peduncular art5–6 less ovoid and bulbous, A2 flagellum with elongate, slender art instead of broad and short art.

#### Diagnosis

**Morphological diagnosis**: Body shape elongated, subcylindrical. Cephalothorax frontal margin concave, rostrum absent. Posterior pereonites and pleotelson medially conjoined, segment borders not expressed. Pleotelson lateral margin anteriorly convex, caudally concave, posterior margin rounded, convex. Antenna 2 article 3 dorsal projection acutely elongated; articles 5–6 ovoid (in males); article 6 with subtriangular projection. Sexual dimorphism present.

**Molecular diagnosis**: Differing in the COI gene from other congeneric NWP species in the nucleotides T (position 96 of alignment), C (108), G (164), G (189), C (287), T (297), G (299), G (300), T (369) and A (513).

#### Etymology

The name "*bulbosus*" is derived from Latin, meaning “round” or “bulbous” and refers to the unusual shape of the pleotelson as well as the peduncular articles 5 and 6 of the male antenna 2.

#### Distribution

Only known from two stations in the Northwest Pacific, abyssal plain of the greater Kuril-Kamchatka Trench Region.

#### Taxon discussion

Most haploniscid species possess a dorsoventrally flattened and oval body shape (see, for example, Lincoln (1985a), Lincoln (1985b)). Only some members of this family show an elongated, subcylindrical body shape similar to *H. bulbosus* **sp. nov.**, which include *H. spinifer* Hansen, 1916, *H. ingolfi* Wolff, 1962 and *H. angustus* Lincoln, 1985 from the Atlantic Ocean (Hansen 1916, Wolff 1962, Lincoln 1985b) as well as *H. menziesi* Birstein, 1963 and *H. gibbernasutus* Birstein, 1971 from the Northwest Pacific (Birstein 1963, Birstein 1971).

The second antenna of *H. bulbosus* **sp. nov.** has a unique shape not observed in any other known haploniscid species, though certain features resemble character states typical of *Antennuloniscus* Menzies, 1962 and *Chauliodoniscus* Lincoln, 1985. In particular, the subapical attachment of the second antenna flagellum in *H. bulbosus* **sp. nov.** is similar to that of *Antennuloniscus alfi* Würzberg & Brökeland, 2006 from the Southern Ocean, which also shares the presence of an acute distal tip on the sixth peduncular article. However, unlike *H. bulbosus* **sp. nov.**, *A. alfi* possesses an elongated cylindrical body form and shows a fusion of the fifth and sixth peduncular articles of the second antenna, a feature diagnostic for *Antennuloniscus* species (Würzberg and Brökeland 2006).

The fifth peduncular article of the second antenna in *H. bulbosus* **sp. nov.** is ovoid. A similar state occurs in several *Chauliodoniscus* species, such as *C. tasmanaeus* Lincoln, 1985. In contrast, most *Chauliodoniscus* species, for example, *C. quadrifrons* Menzies, 1962, bear prominent anterolateral projections that are absent in *H. bulbosus* **sp. nov**. Moreover, the lateral margins of the pereonites are more rounded and less projecting in *H. bulbosus* **sp. nov.** than in *Chauliodoniscus*. The overall body shape of *H. bulbosus* **sp. nov.** is elongate and subcylindrical. This habitus is also found in the Northwest Pacific species *H. gibbernasutus* and *H. menziesi*. However, *H. bulbosus* **sp. nov.** lacks the rostrum that is characteristic of *H. gibbernasutus*. It also differs from *H. menziesi* in the configuration of peduncular articles 5 and 6 of the second antenna, which are unique to *H. bulbosus* **sp. nov.**, as well as in pleotelson morphology, which is not trapezoidal in *H. bulbosus* **sp. nov.**, but is in *H. menziesi*. Furthermore, the frontal margin of the cephalothorax in *H. bulbosus* **sp. nov.** is evenly rounded, whereas *H. menziesi* exhibits a medial bulge.

Taken together, the antennal character states of *H. bulbosus* **sp. nov.** overlap with, but do not conform to, diagnostic features used in the definition of *Antennuloniscus* and *Chauliodoniscus*. Since some of these traits have been central to generic diagnoses, the combination of features in *H. bulbosus* **sp. nov.** highlights the need to re-evaluate apomorphies within the family. Ultimately, the unique mosaic of antennal features in *H. bulbosus* **sp. nov.** challenges several generic diagnoses currently applied within Haploniscidae and underscores the necessity for a comprehensive taxonomic revision of the family (cf. Brökeland and Raupach (2008)). This also reinforces the view that the genus Haploniscus, which accommodates *H. bulbosus* **sp. nov.**, often functions as a repository for species lacking clear synapomorphies with other, more clearly defined genera (Brökeland and Wägele 2004).

#### Notes

##### Methods

*Haploniscus bulbosus* **sp. nov.** was collected during the KuramBio expedition onboard R/V SONNE in 2012 (Brandt and Malyutina 2012, Brandt and Malyutina 2015). Specimens were collected using a camera-epibenthic sledge (C-EBS) (Brandt et al. 2013). Immediately after the C-EBS was brought back to deck, the processing of the samples was conducted following Riehl et al. (2014b). The samples were preliminarily sorted on board to class/order level using stereomicroscopes. The sorting process continued at the home laboratory of the Senckenberg Institute using LEICA M60 stereomicroscopes, sorting the specimens to (morpho-) species level.

After designating holo- and paratypes, voucher images were taken using a LEICA M165 C motorised stereomicroscope, combined with a LEICA DMC 4500 camera and LAS-X software. Post-processing of the images was done using Adobe Photoshop 25.11.

After photographic imaging, the specimens were transferred into a 1:1 solution of glycerine and 70% denatured ethanol and stored for three days allowing the ethanol to evaporate slowly to avoid shrinking of the specimens while being transferred to glycerine. Temporary microscopy slides were prepared for scientific drawings of the habitus and appendages. All drawings were made using a LEICA DM 2500 LED microscope with camera lucida. To keep the male holotype intact, it was solely used for drawings of the habitus and the antennae (*in situ*). For further analysis of the appendages, such as pereopods, mouthparts and pleopods, the male paratype was dissected. Pereopods were drawn separately after dissection. Digitalisation of the drawings was performed in Adobe Illustrator 28.6 following the standards of Coleman (2003).

Confocal Laser Scanning Microscopy (CLSM) was used to illustrate the habitus and appendages, including the mouthparts and pleopods. As for the scientific drawings, the specimens were transferred on to temporary slides. For CLSM scanning, the LEICA TCS SPE2 and the LEICA LAS X software were utilised. Scans were made with a 405 nm laser at a resolution of 2048 x 2048 pixels. Habitus scans were made at 200x magnification, while all appendages were scanned at 400x magnification. The ImageJ2 2.14 software was used to merge the resulting image stacks into single total projections and to assign pseudo-colours. Stitching of the multiple habitus scan images was done using Adobe Lightroom 7.5. Subsequent editing of the CLSM-scans was done with Adobe Photoshop.

Measurements were taken from the respective drawings and CLSM scans using the measurement tool in Adobe Acrobat 24.1. Body and antennal measurements are provided for all holo- and paratypes while mouthpart, pereopod and pleopod measurements stem from the male paratype. Body length as well as segment lengths were measured alongside the mid-axis from each specimens’ anterior cephalothorax margin to the posterior pleotelson margin. Segments were measured at their greatest width, following the standards implemented by Hessler (1970). The species description was prepared using a DELTA (Dallwitz 1980, Dallwitz 1993, Dallwitz et al. 2010) database for haploniscid isopods (Knauber et al., unpublished; Senckenberg Ocean Species Alliance (SOSA) et al. 2024). Specimens were deposited at the Senckenberg Museum in Frankfurt, Germany.

The molecular diagnosis was prepared using the tools DeSigNate (Hütter et al. 2020) and MolD (Fedosov et al. 2022) (as embedded in iTaxoTools 0.1, Vences et al. (2021)), based on seven barcodes of the cytochrome-c-oxidase subunit I (COI). Amplification and sequencing were performed using the primer sets LCO1490/HCO2198 (Folmer et al. 1994) and LCO1490-JJ/HCO2198-JJ (Astrin and Stüben 2008) and the settings described for *Mastigoniscus minimus* Wenz, Knauber & Riehl, 2024 (Senckenberg Ocean Species Alliance (SOSA) et al. 2024). All sequences of *H. bulbosus* were compared to ones belonging to other known congeneric species from the NWP (Knauber, in preparation). *Haploniscus hydroniscoides* Birstein, 1963 was excluded from this comparison despite currently being placed within *Haploniscus* Richardson, 1908, as its molecular identity is closer to *Hydroniscus* Hansen, 1916 (Knauber, unpublished). For DeSigNate, binary and asymmetric positions of the alignment with a discriminative power of 1.0 were considered. Using MolD, single nucleotide mDNCs were used to confirm the results of DeSigNate.

Specimen data for this description were (in parts) gathered via the Discovery Laboratory infrastructure of the SENCKENBERG OCEAN SPECIES ALLIANCE.

**Repository**: Material is deposited in the Senckenberg Research Institute and Natural History Museum, Frankfurt (SMF).

### Macrostylis peteri

Riehl sp. nov.

E2B88D91-A3E6-59AE-98D1-68EFBB572D1E

2A637D3B-16C6-4C20-9951-2C9CC38502A4

https://www.marinespecies.org/aphia.php?p=taxdetails&id=118261

#### Materials

- **Type status:** Holotype. **Occurrence:** occurrenceDetails: in sediment; catalogNumber: NMV J60800; occurrenceRemarks: free-living; recordedBy: G.C.B. Poore; individualCount: 1; sex: female; lifeStage: adult; reproductiveCondition: non-ovigerous; preparations: whole animal (ETOH); disposition: in collection; occurrenceID: 101BB144-53A6-50F1-9725-E96107890E93; **Taxon:** scientificName: *Macrostylis peteri* Riehl; kingdom: Animalia; phylum: Arthropoda; class: Malacostraca; order: Isopoda; family: Macrostylidae; genus: Macrostylis; specificEpithet: peteri; taxonRank: species; scientificNameAuthorship: Riehl; vernacularName: Peter's Long-stalked Sea Slater; nomenclaturalCode: ICZN; **Location:** higherGeography: Indian Ocean; continent: Australia; stateProvince: Western Australia; county: Australia; municipality: Ningaloo South; locality: SS07/2005 Station 23 T1 700; verbatimDepth: 715.17; verbatimCoordinates: 22°3'46.44''S, 113°43'22.8''E; verbatimLatitude: 22°3'46.44''S; verbatimLongitude: 113°43'22.8''E; decimalLatitude: -22.0629; decimalLongitude: 113.723; **Identification:** identifiedBy: Torben Riehl; dateIdentified: 2011; **Event:** samplingProtocol: R/V SOUTHERN SURVEYOR cruise SS02/2005, Smith McIntyre Grab; eventDate: 24.07.2005; habitat: sediment; fieldNumber: SS07/2005 23 T1 700; **Record Level:** institutionID: http://grscicoll.org/institution/museum-victoria; institutionCode: NMV; basisOfRecord: PreservedSpecimen
- **Type status:** Paratype. **Occurrence:** occurrenceDetails: in sediment; catalogNumber: NMV J46837; occurrenceRemarks: free-living; recordedBy: G.C.B. Poore; individualCount: 1; sex: female; lifeStage: adult; reproductiveCondition: non-ovigerous; preparations: whole animal (ETOH); disposition: in collection; occurrenceID: BB12D00E-7FBD-5784-A097-35387E727389; **Taxon:** scientificName: *Macrostylis peteri* Riehl; kingdom: Animalia; phylum: Arthropoda; class: Malacostraca; order: Isopoda; family: Macrostylidae; genus: Macrostylis; specificEpithet: peteri; taxonRank: species; scientificNameAuthorship: Riehl; vernacularName: Peter's Long-stalked Sea Slater; nomenclaturalCode: ICZN; **Location:** higherGeography: Indian Ocean; continent: Australia; stateProvince: Western Australia; county: Australia; municipality: Ningaloo South; locality: SS07/2005 Station 23 T1 700; verbatimDepth: 715.17; verbatimCoordinates: 22°3'46.44''S, 113°43'22.8''E; verbatimLatitude: 22°3'46.44''S; verbatimLongitude: 113°43'22.8''E; decimalLatitude: -22.0629; decimalLongitude: 113.723; **Identification:** identifiedBy: Torben Riehl; dateIdentified: 2011; **Event:** samplingProtocol: R/V SOUTHERN SURVEYOR cruise SS02/2005, Smith McIntyre Grab; eventDate: 24.07.2005; habitat: sediment; fieldNumber: SS07/2005 23 T1 700; **Record Level:** institutionID: http://grscicoll.org/institution/museum-victoria; institutionCode: NMV; basisOfRecord: PreservedSpecimen
- **Type status:** Other material. **Occurrence:** occurrenceDetails: in sediment; catalogNumber: NMV J60803; occurrenceRemarks: free-living; recordedBy: G.C.B. Poore; individualCount: 1; sex: male; lifeStage: subadult; reproductiveCondition: immature; preparations: whole animal (ETOH); disposition: in collection; occurrenceID: 8F9890F4-961E-50E9-BCDA-BB1DC55F618C; **Taxon:** scientificName: Macrostylis cf. peteri Riehl; kingdom: Animalia; phylum: Arthropoda; class: Malacostraca; order: Isopoda; family: Macrostylidae; genus: Macrostylis; specificEpithet: cf. peteri; taxonRank: species; scientificNameAuthorship: Riehl; vernacularName: Peter's Long-stalked Sea Slater; nomenclaturalCode: ICZN; **Location:** higherGeography: Indian Ocean; continent: Australia; stateProvince: Western Australia; county: Australia; municipality: Ningaloo North; locality: SS07/2005 Station 33 T2 400; verbatimDepth: 429.392; verbatimCoordinates: 21°58'0.85''S, 113°47'20.4''E; verbatimLatitude: 21°58'0.85''S; verbatimLongitude: 113°47'20.4''E; decimalLatitude: -21.9669; decimalLongitude: 113.789; **Identification:** identifiedBy: Torben Riehl; dateIdentified: 2011; identificationRemarks: species identity unclear due to juvenile stage and lack of genetic data; **Event:** samplingProtocol: R/V SOUTHERN SURVEYOR cruise SS02/2005, Smith McIntyre Grab; eventDate: 24.07.2005; habitat: sediment; fieldNumber: SS07/2005 33 T2 400; **Record Level:** institutionID: http://grscicoll.org/institution/museum-victoria; institutionCode: NMV; basisOfRecord: PreservedSpecimen

#### Description

**Description non-ovigerous (preparatory) female**

**Figure 40.**
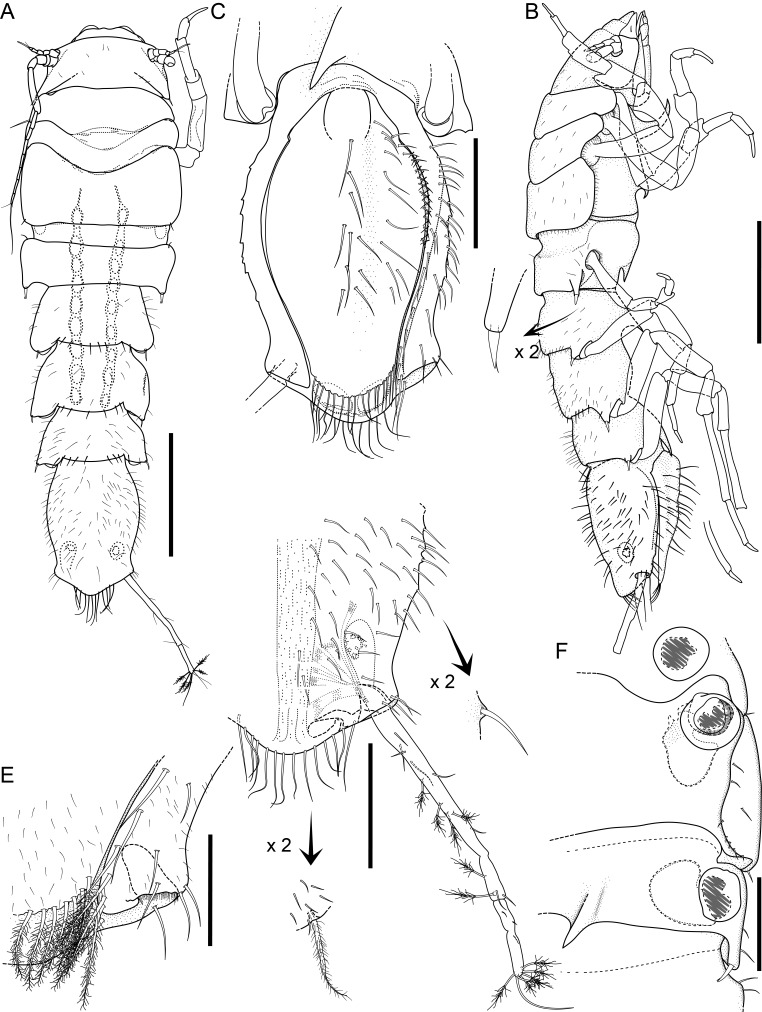
*Macrostylis peteri* Riehl, **sp. nov.**, non-ovigerous female holotype (J60800) habitus. **A** Dorsal habitus; **B** Lateral habitus; **C** Pleotelson, ventral; **D** Pleotelson, posterolateral margin, statocyst and uropod; **E** Pleotelson uropodal insertion, ventral; **F** Sternites 1-3 with developing internal oostegites (ventral). Scale bars: 0.5 mm (A, B), 0.2 mm (C, D, F), 0.1 mm (E).

**Figure 41.**
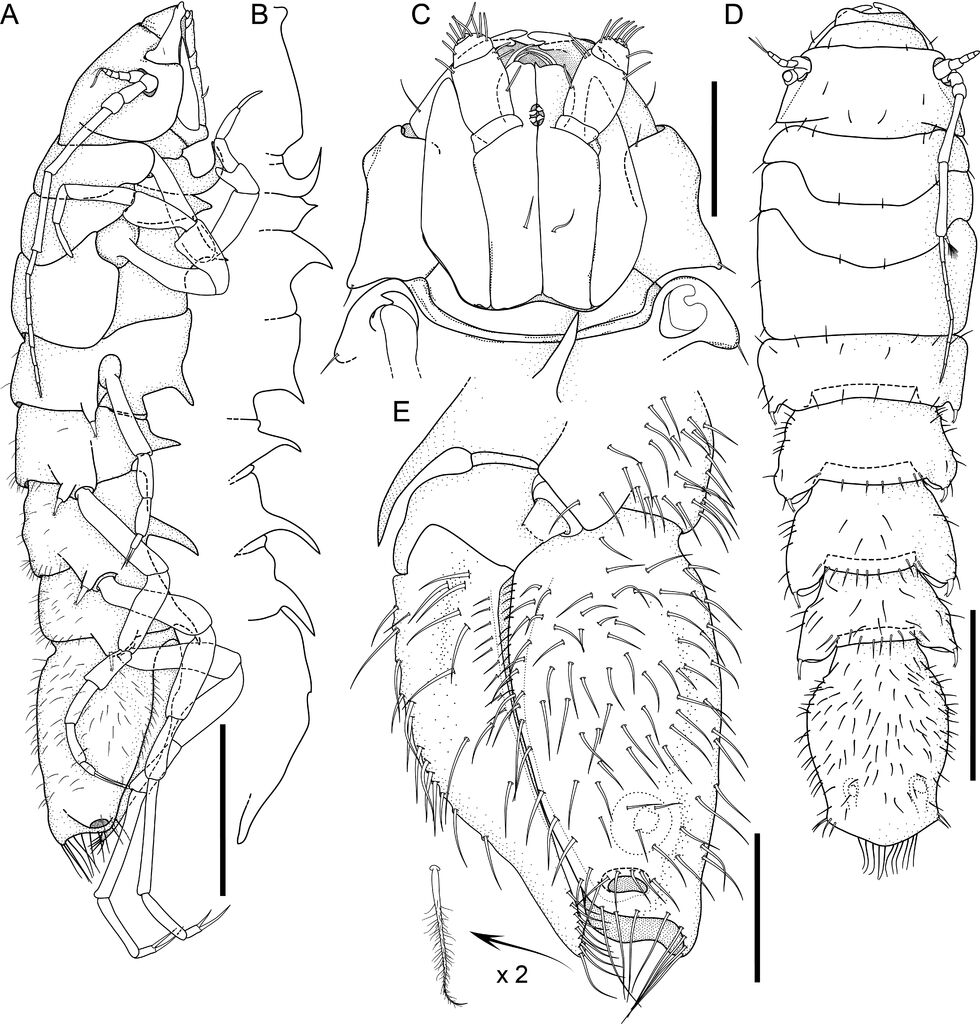
*Macrostylis peteri* Riehl, **sp. nov.**, non-ovigerous female paratype (J46837) habitus. **A** Habitus, lateral; **B** Ventral outline, lateral; **C** Head with maxillipeds, ventral; **D** Habitus, dorsal; **E** Pleotelson, lateral. Scale bars: 0.5 mm (A, B, D), 0.2 mm (C, E).

**Figure 42.**
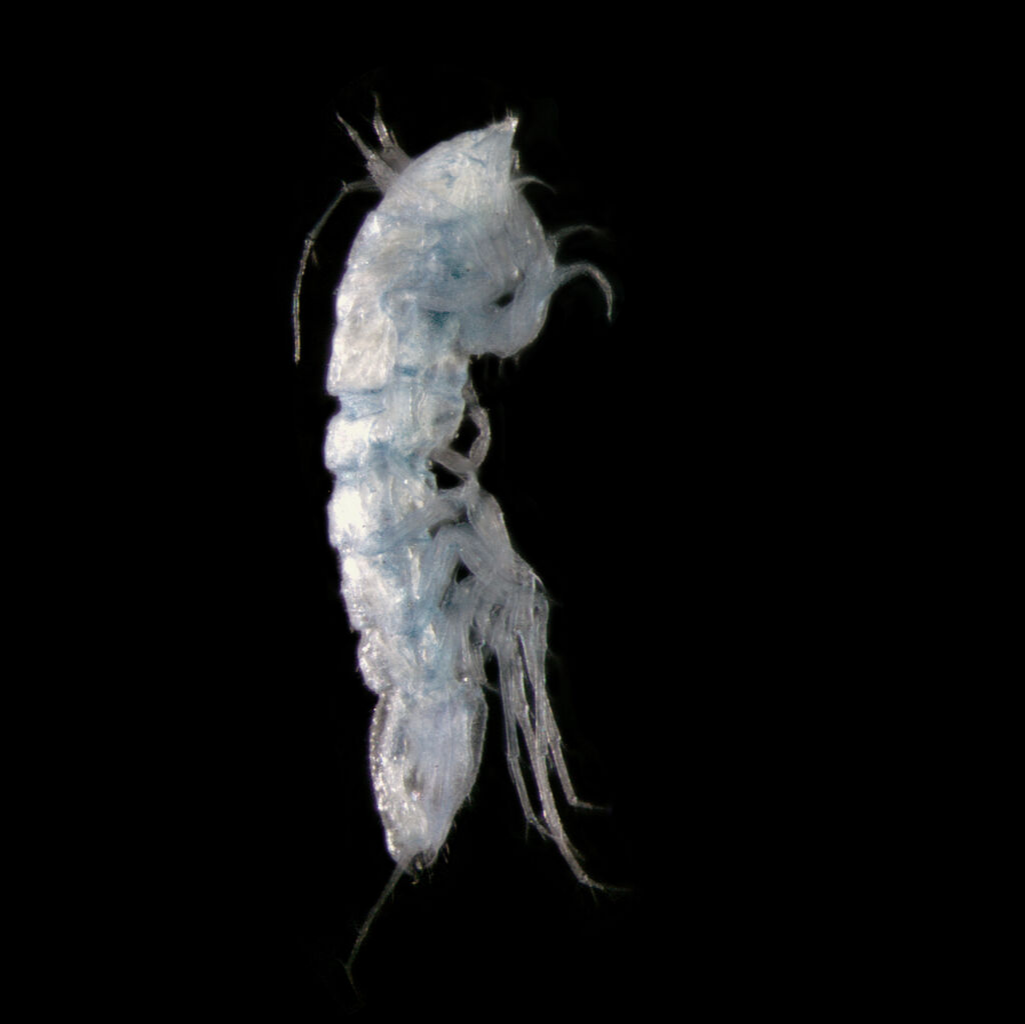
*Macrostylis peteri* Riehl, **sp. nov.**, non-ovigerous female paratype (J46837) habitus, lateral.

**Body** (Figs 40, 41, 42) shape broadest in anterior half, narrowing posteriorly, subcylindrical; length 2.3–2.4 mm, 3.5–3.8 width, tergite and Plt surfaces hirsute, rest of body less setose. **Ventral projections** (Fig. 40B, Fig. 41B) present; Prn1 spine acute and prominent; Prn2 spine directed ventrally, acute, prominent, located medially; Prn3 spine acute, prominent, medially positioned on; Prn4 spine directed posteriorly, acute, prominent, closer to posterior segment border; Prn5 spine acute, prominent, positioned closer to posterior segment border; Prn6 spine acute, prominent, positioned closer to posterior segment border; Prn7 spine prominent, acute; spines 3-7 directed posteroventrally. **Ceph** (Fig. 40A, Fig. 41C) length 0.45–0.66 width, 0.10–0.15 body length; frontal furrow present, laterally bent anteriorly, medially straight; posterolateral setae present, flexibly articulated. **Fossosoma** (Fig. 40A) length 0.85–0.89 width, 0.24 body length; ventral surface without carina, lateral tergite margins confluent, posterolaterally setose. **Prn1-3** (Fig. 40A, B) posterolaterally with simple seta respectively. **Prn4** (Fig. 40A) width 1.1–1.2 pereonite 5 width, length 0.3–0.39 width; with well developed collum, medially widest, with relatively slightly projecting posterolateral margin; lateral margins sinuoid, narrowest anteriorly at collum, widest at coxal insertions, progressively narrowing towards posterolateral protrusions; posterior tergite margin setose, setae flexibly articulated, not extending beyond posterolateral margin; posterolateral margins projecting, contracting laterally, tapering; posterolateral setae robust, spine-like, articulating on pedestals. **Prn5** (Fig. 40A) length 0.48–0.5 width, 1.0–1.4 Prn4 length; posterior tergite margin setose, setae flexibly articulated, not extending beyond posterolateral margin; posterolateral margins rounded; posterolateral setae robust, spine-like, articulating on pedestals. **Prn6** (Fig. 40A) length 0.53–0.57 width, 1.02–1.08 Prn5 length; posterior tergite margin setose; setae flexibly articulated, not extending beyond posterolateral angles; posterolateral margin projecting, rounded; posterolateral setae robust, flexibly articulated. **Prn7** (Fig. 40A) length 0.42–0.45 width; posterior tergite margin setose, setae flexibly articulated, not extending beyond posterolateral margin. Posterolateral margin projecting posteriorly, tapering, subangular; posterolateral setae robust, (149) flexibly articulated. **Plt** (Fig. 40) length 0.23–0.24 body length, 1.4 width, slightly narrower than Prn7, near-elliptic; posterior margin at uropod insertions straight to convex; apex convex, spatulate, length 0.15–0.18 Plt length, with 12 pappose setae positioned on and around apex; pleopodal cavity width 0.76 Plt width; setal ridges visible in dorsal view; longitudinal trough width 0.41 Plt width; anal opening caudally in the trough, exposed and superficial, tilted posteriorly relative to frontal plane.

**Figure 43.**
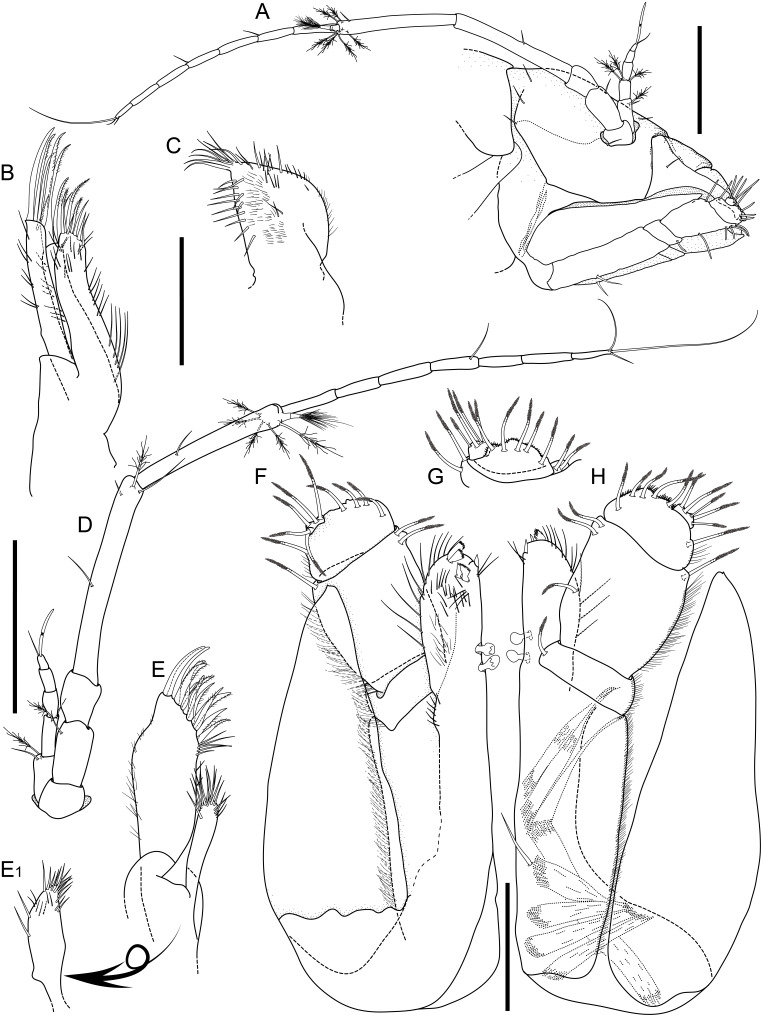
*Macrostylis peteri* Riehl, **sp. nov.**, non-ovigerous female paratype (J46837) head appendages. **A** Head with antennula and antenna, lateral; **B** Maxilla; **C** Paragnaths (right side); **D** Antennula and antenna; **E** Maxillula with inner lobe separately illustrated (E1); **F** Maxilliped (ventral); **G** Maxilliped palp (frontal); **H** Maxilliped (dorsal). Scale bars: 0.2 mm (A, D), 0.1 mm (B, C, E-H).

**Figure 44.**
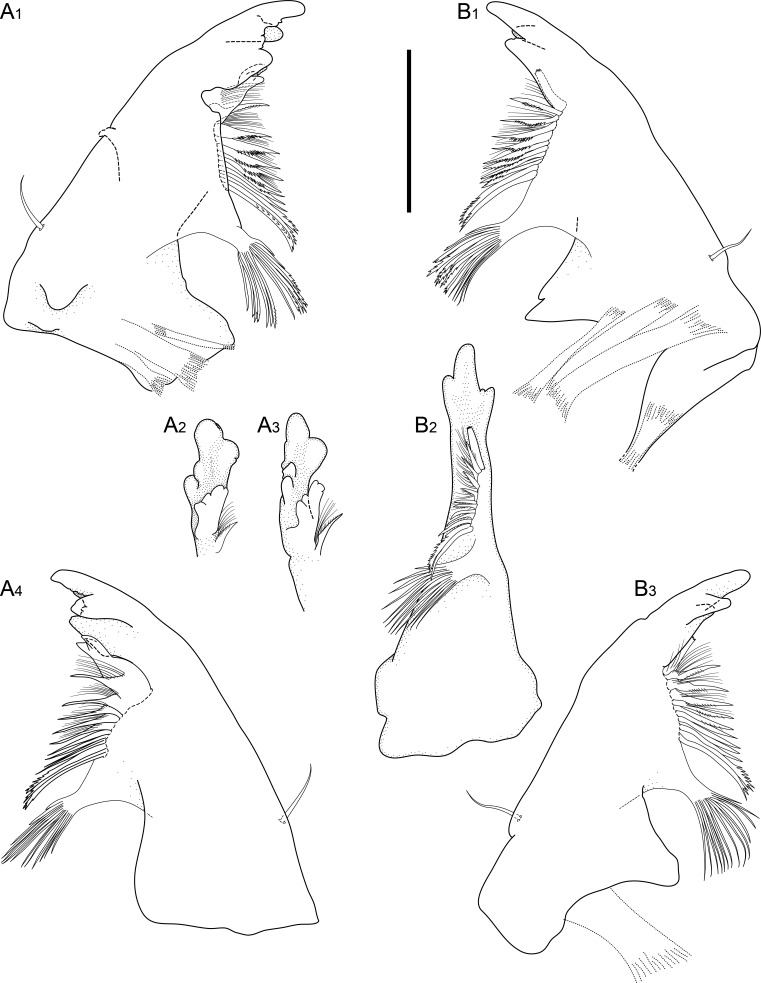
*Macrostylis peteri* Riehl, **sp. nov.**, non-ovigerous female paratype (J46837) mandibles. **A** Left mandible, dorsal (A1); incisor, ventromedial (A2); incisor, medial (A3); ventral (A4); **B** Right mandible, dorsal (B1); medial (B2); ventral (B3). Scale bar: 0.1 mm.

**A1** (Fig. 43A, D) length 0.22 head width, 0.13 A2 length, width 0.53 A2 width; Art decreasing in size from proximal to distal, relative Art length ratios 1.0 : 0.85 : 0.55 : 0.49 : 0.33; Art L/W ratios 2.0, 2.9, 1.8, 1.9, 1.8; all Art longer than wide; Art1 longest and widest; terminal article with 1 aesthetascs; aesthetasc with intermediate belt of constrictions. **A2** (Fig. 43A, D) length 0.4 body length; C length shorter than width; basis length exceeding width, more than twice C length; ischium longer than C; merus and carpus each longer than C, basis and ischium combined; carpus shorter than merus length; (218) flag with 7 Art. **Md** (Fig. 44) with lateral setae; molar process length greater than incisor length; both incisor processes oligodentate with dorsal and ventral subdistal teeth that partly enclose lacinia, left Md incisor process with four cusps, lacinia mobilis robust, similar to incisor process, with four cusps; right Md incisior process with three cusps, lacinia mobilis spine-like, smaller than left lacinia.

**Figure 45.**
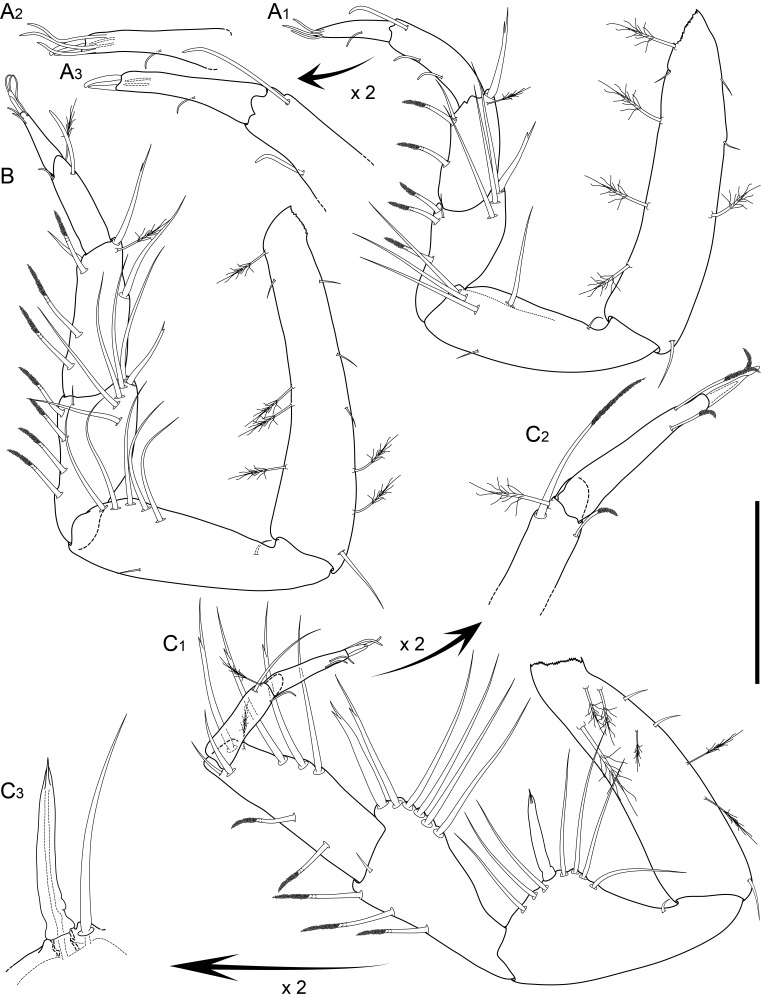
*Macrostylis peteri* Riehl, **sp. nov.**, non-ovigerous female paratype (J46837) anterior pereopods. **A** Pereopod 1 (A1) with magnified dactylus (A2), with sensillae omitted (A3); **B** Pereopod 2; **C** Pereopod 3 (C1), with dactylus (C2) and ischium apical setae (C3) enlarged. Scale bar: 0.2 mm.

**Figure 46.**
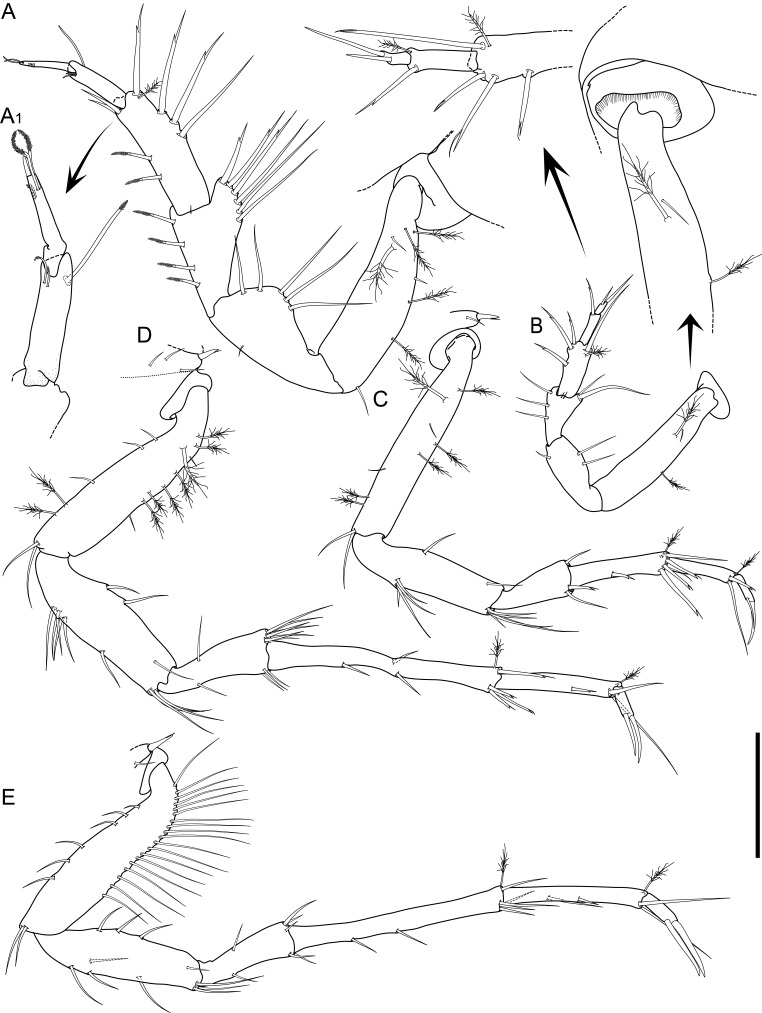
*Macrostylis peteri* Riehl, **sp. nov.**, non-ovigerous female holotype (J60800) pereopods. **A** Pereopod 3 with magnified dactylus (A1); **B** Pereopod 4 with magnified propodus and dactylus; **C** Pereopod 5; **D** Pereopod 6; **E** Pereopod 7. Scale bar: 0.2 mm.

**Figure 47.**
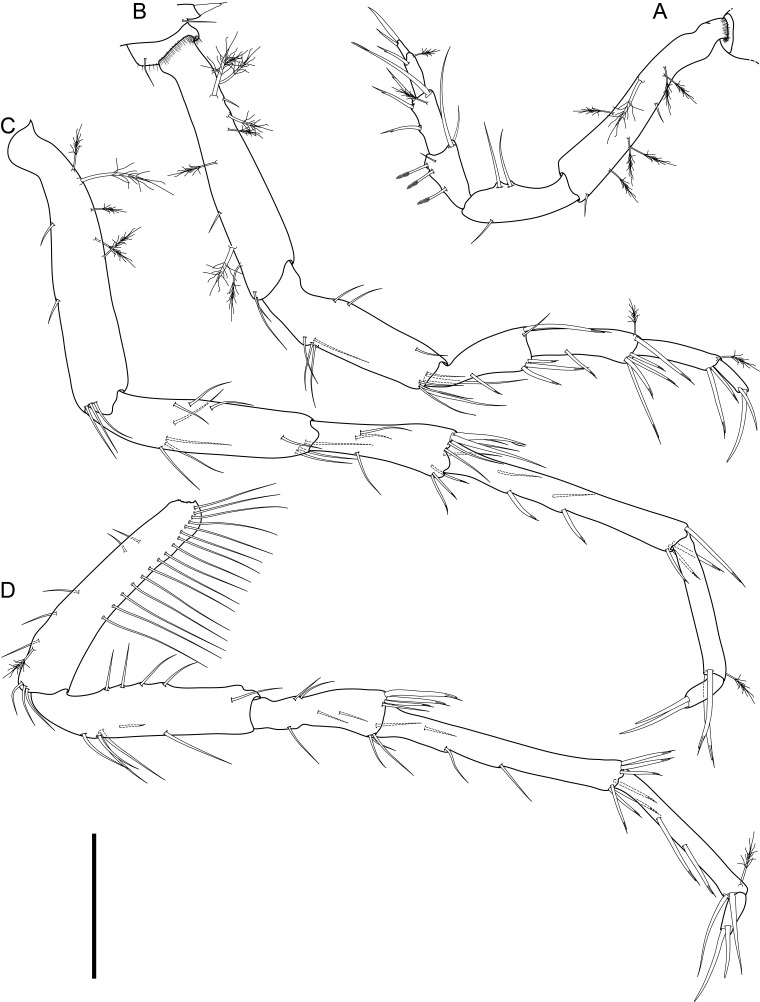
*Macrostylis peteri* Riehl, **sp. nov.**, non-ovigerous female paratype (J46837) posterior pereopods. **A** Pereopod 4; **B** Pereopod 5; **C** Pereopod 6; **D** Pereopod 7. Scale bar: 0.2 mm.

**Mxp** (Fig. 43F-H) basis length 4 width; endite distally with four fan setae, medioventrally setose; palp wider than endite, Art2 wider than Art1; ischium distomedially with one seta, shorter than Art3, Art4 distomedial extension present, Art5 absent; epipod length 3.2 width, 1.0 coxa-basis length. **P1** (Fig. 45) length 0.46 body length; ischium dorsal margin with five simple setae; merus dorsal margin with six setae: five simple, long, one more robust, bifid apically, ventral margin with four medially biserrate, distally sensillate setae; carpus dorsally with three setae: one simple, long, one broom, one bifurcate; dactylus medially-subdistally with three sensillae, terminal claw length 0.28 dactylus length. **P2** (Fig. 45B) length 0.42 body length. Ischium dorsally with 4-5 simple setae submarginally; merus dorsally with 4-5 simple, long setae submarginally and one bifurcate seta distodorsally, ventrally with four medially biserrate, distally sensillate setae, on ventral margin, with 1-2 minute, simple distomedially; carpus dorsally with 2-3 setae: 0-1 simple, one broom, one bifurcate, ventrally with 3-4 setae: 2-3 medially biserrate, distally sensillate, one slender, simple, asetulate; dactylus medially-subdistally with three sensillae. **P3** (Fig. 45C) length 0.48 body length. Ischium dorsal lobe subtriangular; dorsal margin proximally with four asetulate setae; apex with one prominent, robust, sensillate, bifid, straight, flexibly articulated seta; distally with row of three asetulate setae; merus dorsally with six setae: four simple, slender, asetulate, one bifurcate, slender, one bifurcate, robust, ventrally with three medially biserrate, distally sensillate setae; carpus dorsally with five setae: one simple, slender, three bifurcate, slender, one broom; carpus ventrally with four setae: two medially biserrate, distally sensillate, two simple, asetulate; dactylus medial cuticle subdistally with three sensillae. **P4** (Fig. 46B, Fig. 47C) length 0.27 body length; carpus oval in cross section.

**P5** (Fig. 46C, Fig. 47B) length 0.47 body length. **P6** (Fig. 46, Fig. 47C) length 0.63 body length. **P7** (Fig. 46E, Fig. 47D) length 0.63 body length; basis length 4.0 width, dorsal margin row of 16 elongate setae, exceeding beyond proximal half of article, setae longer basis width, ventral margin with row of six elongate setae, setae shorter basis width; ischium length 3.5 width, mediodorsally with three simple setae, medioventrally with three simple scattered setae, distoventrally with one simple seta; merus length 2.7 width, distodorsally with three simple setae, medioventrally with simple one seta, (343) distoventrally with two simple setae; carpus length 8.5 width, distodorsally with two setae: one simple, one broom, medioventrally with two simple setae, distoventrally with four setae; propodus length 7.3 width; dactylus length 3.0 width.

**Op** (Fig. 40C, Fig. 41E) elongate; length 2.0 width, 1.0 pleotelson dorsal length, completely covering anus; distally tapered; distal margin broad, apical width 0.52 operculum width; with rounded, edgeless keel; with lateral fringe consisting of 12–14 setae on either side, with lateral fringe separate from apical row of setae; with 16 short apical setae. **Plp3** endopod plumose setae shorter endopod; exopod monoarticulate, with one conspicuous subapical seta. **Plp4** distal pappose seta absent, exopod lateral fringe of setae present. **Urp** (Fig. 40A, D) length 0.87 pleotelson length; protopod cylindrical, distal margin blunt, endopod insertion terminally; protopod length 10.8 width, 0.65 pleotelson length; endopod width at articulation subequal protopod width, length 6.2 width, 0.36 protopod length.

**Macrostylis cf. peteri**

**Description juvenile male**

**Figure 48.**
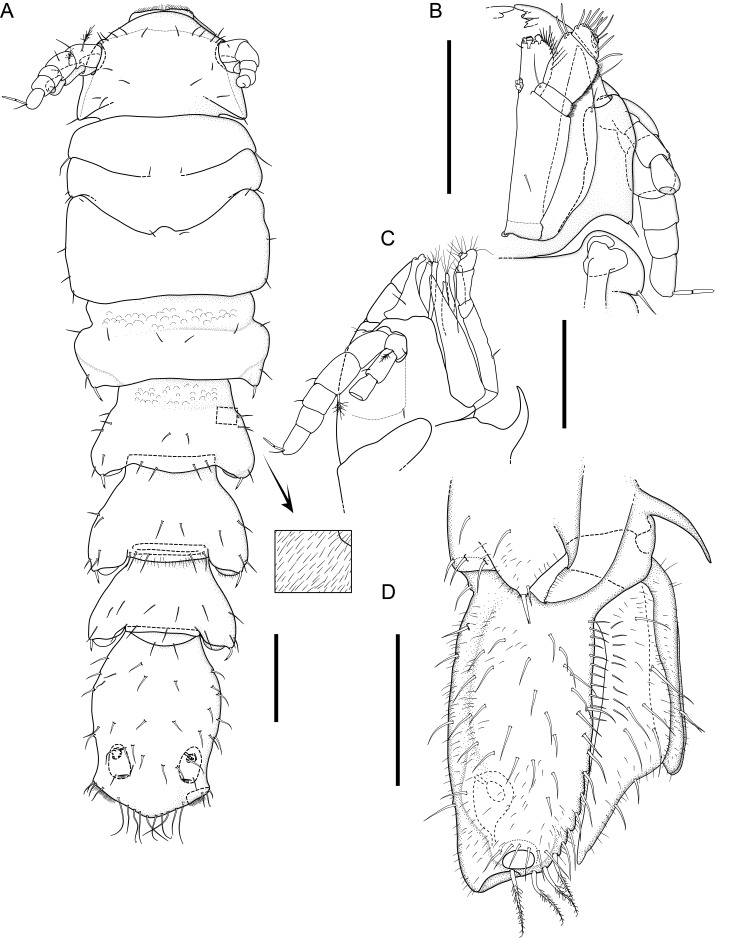
Macrostylis cf. peteri Riehl, **sp. nov.**, subadult male (J60803) habitus. **A** habitus dorsal; **B** Cephalothorax, left half, ventral; **C** Cephalothorax, lateral; **D** Pleotelson, lateral. Scale bar: 0.2 mm.

**Body** (Fig. 48A) more elongate than in female, length 1.84 mm, 4.0 width. **Ventral projections** relatively needle-shaped, slender and pointed; Prn3-4 spines acute, prominent, located medially, directed posteroventrally; Prn4 spine directed posteriorly, prominent; Prn5-7 spines prominent, located closer to posterior segment border, directed posteroventrally. **Cephalothorax** (Fig. 48A, C) length 0.62 width, 0.13 body length; frontal furrow present, with row of small setae, posterior tergum on either side with four setae in subtriangular arrangement, posterolateral setae present. **Fossosoma** (Fig. 48) length 0.87 width. **Prn3** posterolaterally with two asensillate, robust, flexibly articulated setae.

**Prn4** (Fig. 48) integration with other segments clearly distinct from both anterior and posterior pereonites: with well-developed collum, widest medially, relatively small posterolateral projections; lateral margins sinusoid; posterolateral margins tapering; posterolateral setae robust, sensillate, spine-like. **Prn5** posterolateral setae sensillate, robust, flexibly articulated.

**Pln1** (Fig. 48) tergal articulation with Plt present, clearly visible, laterally meeting with Prn7 posterior margin resulting in a wedge-shaped appearance or pleonite 1; medially with pair of simple setae near posterior margin. **Plt** (Fig. 48) hourglass-shaped; length 1.4 width, 0.23 body length, slightly narrower than pereonite 7, posterior margin at uropod insertions straight; posterior apex convex, subtriangular, length 0.17 Plt length, with 10 setae on apex; pleopodal cavity width 0.83 Plt width, longitudinal trough width 0.40 Plt width.

**A1** (Fig. 48B, C) length 0.8 head width; art1, 2 and 5 elongate tubular; art 3–4 squat or noticeably shorter; aesthetascs with intermediate belt of constrictions; art1, 2 elongate, of subsimilar length; art3–4 squat, shorter than article 1; art5 elongate, shorter than art1. **A2** (Fig. 48B, C) coxa squat; basis elongate, cylindrical along whole axis, longer than coxa; ischium elongate, cylindrical along whole axis.

**Figure 49.**
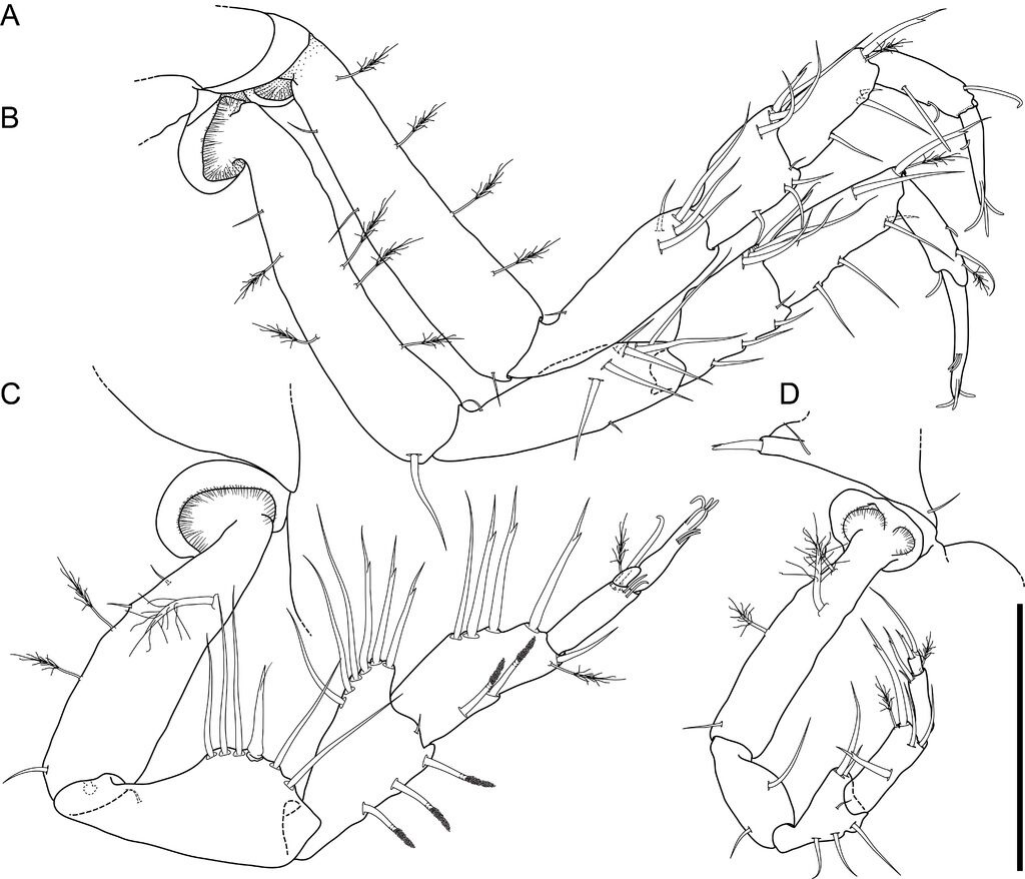
Macrostylis cf. peteri Riehl, **sp. nov.**, subadult male (J60803) anterior pereopods. **A** Pereopod 1; **B** Pereopod 2; **C** Pereopod 3; **D** Pereopod 4. Scale bar: 0.2 mm.

**P1** (Fig. 49A) ischium with dorsal setae submarginally. **P3** (Fig. 49C) length 0.46 body length; dorsal lobe flat and rounded; proximally with three simple setae; apex with one prominent seta, apical seta robust, flexibly articulated, straight, bifid; distally with two simple setae; ischium dorsal margin with row of six setae: three simple, three bifurcate; merus ventrally with three setae, distally setulate; carpus dorsally with four setae in row along margin: one simple, three bifurcate; carpus ventrally with four setae: two medially submarginally, distally sensillate, one broom distally, one simple distally near carpo-propodal articulation.

**Figure 50.**
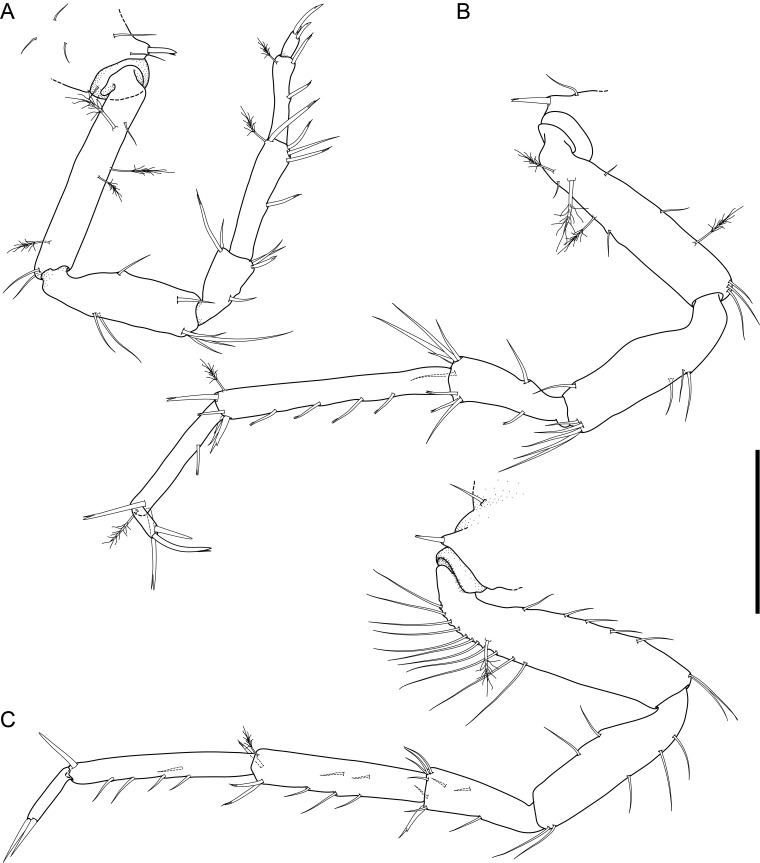
Macrostylis cf. peteri Riehl, **sp. nov.**, subadult male (J60803) posterior pereopods. **A** Pereopod 5; **B** Pereopod 6; **C** Pereopod 7. Scale bar: 0.2 mm.

**P6** (Fig. 50B) length 0.64 body length; art L/W ratios 5.2, 3.3, 2.5, 7.0, 8.0, 2.0; relative art length ratios 1.0, 0.74, 0.48, 0.90, 0.52, 0.13. **P7** (Fig. 50C) length 0.67 body length; relative article length ratios 1.0, 0.70, 0.42, 0.64, 0.67, 0.24; basis length 4.1 width; dorsal margin row of 11 elongate setae; ventral margin with row of five setae, setae shorter basis than width; ischium length 3.3 width, mediodorsally with two simple setae in row, medioventrally with three simple setae in row; distoventrally with two simple setae; merus length 2.8 width, distodorsally with four simple setae, medioventrally with one simple seta; distoventrally with three setae: one slender, simple laterodistally, one robust, bifurcate, one simple, slender mediodistally; carpus length 4.2 width, mediodorsally with two simple setae submarginally, distodorsally with two setae: one broom, one bifid, semi-robust, medioventrally with three simple setae in marginal row, distoventrally with two bifid, semi-robust setae; propodus length 7.3 width; dactylus length 4.0 width.

**Figure 51.**
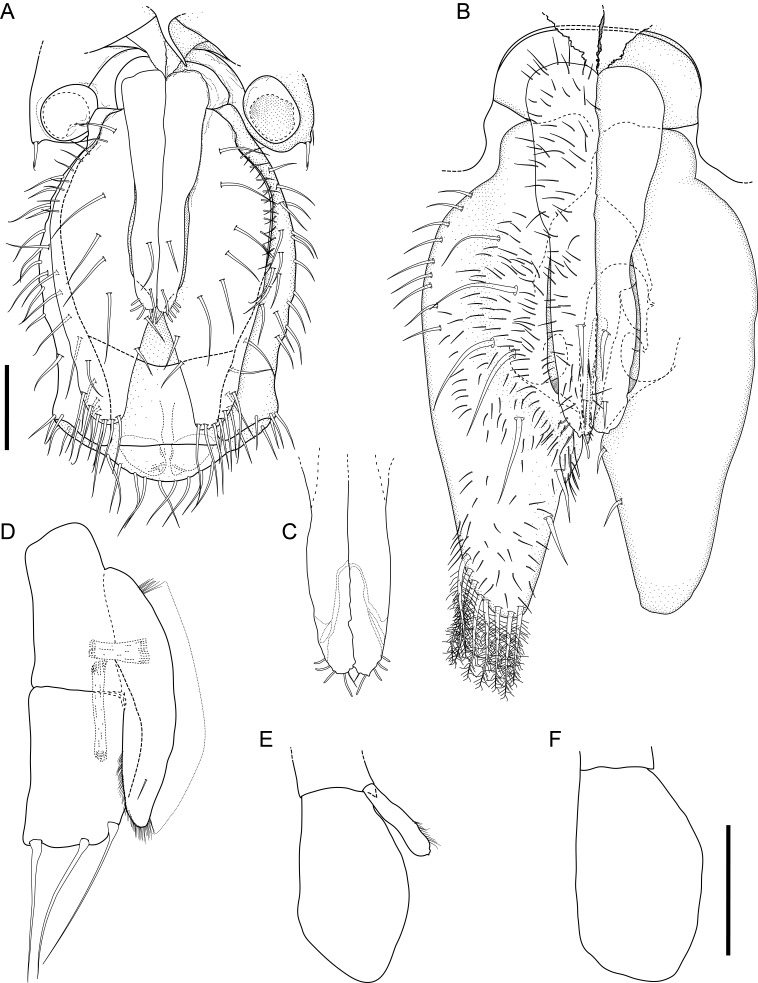
Macrostylis cf. peteri Riehl, **sp. nov.**, subadult male (J60803) pleopods. **A** Pleotelson, ventral (seventh pereopods omitted); **B** Pleopods 1 and 2, in situ; **C** Pleopod 1, distal part; **D** Pleopod 3; **E** Pleopod 4; **F** Pleopod 5. Scale bar: 0.1 mm.

**Plp1** (Fig. 51A, B, C) shorter Plp2, with the latter projecting beyond Plp1; medial lobes distally with five sensillae, ventrally with setae present; distally projecting ventrally beyond Plp2 ventral margin. **Plp2** (Fig. 51B) protopod apex tapering, distally enclosing Plp1 and converging towards counterpart, with nine setae on proximolateral margin; with eight pappose setae distally.

##### Type material

Holotype (NMV J60800), adult, non-ovigerous female; paratype (NMV J46837), adult, non-ovigerous female.

##### Material examined

*Macrostylis peteri* **sp. nov.** Holotype (NMV J60800) and paratype (NMV J46837) and two additional specimens:

- Macrostylis cf. peteri:
  - one sub-adult male (NMV J60803) from near the type locality, station SS07/2005 Station 33 T2 400, 21°58'0.85''S, 113°47'20.4''E, 429.4 m depth.
- *Macrostylis* sp 2:
  - two adult, ovigerous females (NMV J60820) from south-eastern Indian Ocean, Australian continental shelf, off Western Australia, Barrow region, 21°00'30.2"S, 114°22'51.6"E, 397 m depth.
  - one adult, non-ovigerous female (NMV J60801), one adult male (NMV J46835) and two juveniles (NMV J46836) from south-eastern Indian Ocean, Australian continental shelf, off Western Australia, Ningaloo Region, 22°01'26.0"S, 113°39'25.2"E, 1073 m depth.

##### Type locality

South-eastern Indian Ocean, Australian continental shelf, off Western Australia, Ningaloo Region, 22°3'46.44''S, 113°43'22.8''E, 715.2 m depth.

#### Diagnosis

Pleotelson posterior apex subtriangular; male pleonite 1 articulation with pleotelson expressed. Maxilliped dactylus reduced, absent. Pereopod 3 ischium dorsal lobe flat subtriangular, dorsal margin proximally with row of 3–4 asetulate setae; apex with one flexibly articulated, straight, prominent robust, setulate, bifid seta; distally with row of 2–3 asetulate setae. Operculum relatively large, ca. 0.8 pleotelson width, projecting caudally to near pleotelson apex, covering anal opening.

#### Etymology

The specific epithet honours the father of the describer, Claus-Peter Riehl.

#### Distribution

Only known from the type locality (Fig. 52).

#### Taxon discussion

**On the assignment of a male**

As the examined male specimen is not at the adult/copulatory stage, it has not been fully scored and evaluated in DELTA, particularly with respect to morphometric data. This omission is based on established evidence indicating that the morphology of males in many species undergoes significant changes during the terminal (adult) moult (Riehl et al. 2012, Bober et al. 2017). Nevertheless, the specimen exhibits distinct character states that unequivocally place it within the *Macrostylis subinermis* group of the Macrostylidae. Specifically, the antennula displays a characteristic sequence of length-to-width ratios that diverges from the plesiomorphic condition (see, for example, Riehl et al. (2012), Riehl et al. (2014a)) and the arrangement of the first and second pleopods is diagnostic: distally, the first pleopods project posteroventrally from within the second pleopods, which envelop them at their distal region.

Sexual dimorphism can complicate the assignment of conspecific adult males and females. In the case of *Macrostylis peteri* Riehl, **sp. nov.**, male and female specimens were collected from stations separated by several miles (km) and at slightly different depths. Although spatial co-occurrence alone would not definitively confirm conspecificity, it would provide supporting evidence. However, in this instance, the subadult male — typically morphologically similar to the female (Riehl et al. 2012, Riehl 2014) — exhibits several shared characteristics with the female that further substantiate the assumption of conspecificity. These include its slightly smaller body size relative to the female, a common trait in macrostylids (Bober et al. 2017), as well as general morphological similarities, such as the shape of pereonite 4, the distribution of posterolateral pereonal setae, the incomplete tergal separation between pereonites 1 and 2, the size and distribution of ventral spines and the extent of pleopod 2 (operculum), which projects nearly to the posterior pleotelson apex and partially covers the anus. Nevertheless, doubt remains about the conspecifity of the male and female specimens investigated due to the lack of molecular data and the juvenile stage of the male specimen. Hence, the male specimen cannot be allocated to *Macrostylis peteri* Riehl, **sp. nov.** with certainty.

#### Notes

**First macrostylid from Australian waters**

*Macrostylis peteri* Riehl, **sp. nov**. is the first species of the family Macrostylidae reported from Australian waters.

**Oostegite development**

For the first time, the internal development of the oostegites during the preparatory phase could be observed for the family Macrostylidae. This character state differentiates Macrostylidae from the Janiroidea families Munnopsidae and Desmosomatidae which have been discussed as potentially closely related to Macrostylidae. This internal development has been observed as well in Urstylis Riehl, Wilson & Malyutina, 2014 and was one of the arguments suggesting a sister-group relationship between Macrostylidae and Urstylidae (Riehl et al. 2014a).

#### Methods

Samples were collected during the Australian R/V SOUTHERN SURVEYOR cruise SS07-2005 (Williams et al. 2005). Sediment samples were obtained using a Smith-McIntyre grab (Smith and Mcintyre 1954).

Specimens were initially preserved in 70% denatured ethanol. For taxonomic examination, they were subsequently transferred from 70% ethanol into an ethanol-glycerine solution (1:1) before being placed in glycerine. To facilitate morphological illustration, dissected appendages were temporarily mounted on slides following the methodology of Wilson (2008) and stained with methyl green. Permanent slide mounts of dissected appendages were prepared using Euparal, following Riehl and Kaiser (2012). Whole specimens and dissected parts were documented using stack photography with a Visionary Digital™ system at the Australian Museum in Sydney.

Pencil drawings were produced from temporary slide mounts using a compound microscope, equipped with a camera lucida. Morphological character states were coded utilising the DELTA software (Dallwitz 1980, Dallwitz 1993, Dallwitz et al. 2010) and an updated DELTA database for Macrostylidae (Riehl et al. 2012, Riehl and De Smet 2020) which is publicly available via the Zenodo repository (Riehl 2024). Description texts and diagnoses were generated from this database. Linear measurements were derived from line drawings using the distance-measurement tool in Adobe Acrobat Professional, following the protocol outlined by Hessler (1970). Calibration was performed with a stage micrometer and ratios were reported in accordance with Wilson (1989). Line drawings were digitally rendered using Adobe Illustrator CS5 and CS6 (Coleman 2009, Bober and Riehl 2014) and subsequently arranged into figure plates in Adobe Illustrator CC.

Morphological terminology follows established conventions for Janiroidea (Wilson 1989, Riehl et al. 2014a). Ratios described as ‘near’ or ‘subequal’ are defined as within ±5% of the comparative measurement, as per Kavanagh and Wilson (2007). Nomenclature for setae adheres to the classifications of Hessler (1970) and Riehl and Brandt (2010). To ensure consistency and comparability, this study employs nomenclature for the antennal podomeres rather than numerical designation, following Hansen (1893).

**Repository**: Specimens have been deposited in the collection of Museum Victoria, Australia (NMV).

### Hoplopolemius olo

Jóźwiak & Stępień sp. nov.

E1E9068B-DE72-5891-9DB3-27665BD650A9

A6BE6FD7-58AE-49CB-A454-7F4E6F002C14

#### Materials

- **Type status:** Holotype. **Occurrence:** catalogNumber: SMF 57072; recordedBy: Marine Environmental Monitoring Ghana 2012; individualCount: 1; sex: female; lifeStage: adult; reproductiveCondition: ovigerous; establishmentMeans: native; occurrenceStatus: present; occurrenceID: 943DD085-468E-5C32-A894-B9C98FB4D950; **Taxon:** scientificName: *Hoplopolemius olo* Jóźwiak & Stępień; kingdom: Animalia; phylum: Arthropoda; class: Malacostraca; order: Tanaidacea; family: Metapseudidae; genus: Hoplopolemius; specificEpithet: olo; taxonRank: species; scientificNameAuthorship: Jóźwiak & Stępień; nomenclaturalCode: ICZN; **Location:** higherGeography: North Atlantic Ocean; waterBody: Gulf of Guinea; country: Ghana; locality: Gulf of Guinea, station G3/25; verbatimDepth: 28 m; verbatimLatitude: 4°45'52.9"N; verbatimLongitude: 2°07'57.7"W; decimalLatitude: 4.7647; decimalLongitude: -2.1327; **Identification:** identifiedBy: Piotr Jóźwiak; dateIdentified: 2025; **Event:** samplingProtocol: 0.1 m² van Veen grab, sieved throught 0.3 mm mesh; eventDate: 19/11/2012; habitat: Fine sandy mud; fieldNumber: G3/26; **Record Level:** institutionCode: SMF; collectionCode: Crustacea; basisOfRecord: PreservedSpecimen
- **Type status:** Paratype. **Occurrence:** catalogNumber: SMF 57073; recordedBy: Marine Environmental Monitoring Ghana 2013; individualCount: 1; sex: female; lifeStage: preadult; reproductiveCondition: non-ovigerous; establishmentMeans: native; occurrenceStatus: present; occurrenceID: 21BB24E9-428A-5879-910F-EFEA8466AEAC; **Taxon:** scientificName: *Hoplopolemius olo* Jóźwiak & Stępień; kingdom: Animalia; phylum: Arthropoda; class: Malacostraca; order: Tanaidacea; family: Metapseudidae; genus: Hoplopolemius; specificEpithet: olo; taxonRank: species; scientificNameAuthorship: Jóźwiak & Stępień; nomenclaturalCode: ICZN; **Location:** higherGeography: North Atlantic Ocean; waterBody: Gulf of Guinea; country: Ghana; locality: Gulf of Guinea, station G3/25; verbatimDepth: 28 m; verbatimLatitude: 4°45'52.9"N; verbatimLongitude: 2°07'57.7"W; decimalLatitude: 4.7647; decimalLongitude: -2.1327; **Identification:** identifiedBy: Piotr Jóźwiak; dateIdentified: 2025; **Event:** samplingProtocol: 0.1 m² van Veen grab, sieved throught 0.3 mm mesh; eventDate: 19/11/2012; habitat: Fine sandy mud; fieldNumber: G3/26; **Record Level:** institutionCode: SMF; collectionCode: Crustacea; basisOfRecord: PreservedSpecimen

#### Description

**Figure 53.**
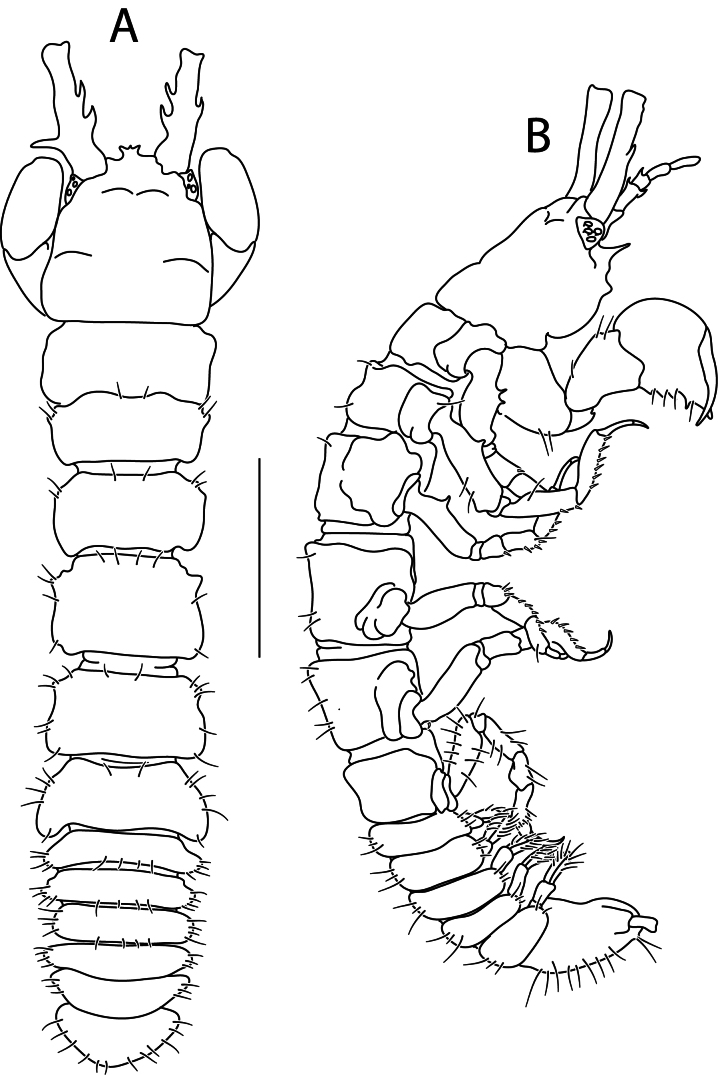
*Hoplopolemius olo* Jóźwiak & Stępień, **sp. nov.** Ovigerous female, holotype (SMF 57072), habitus. **A** Dorsal view; **B** Lateral view. Scale bar: 1 mm.

**Ovigerous female**: Habitus (Fig. 53A, B). Body length (BL) = 4.6 mm, 5.6 times as long as wide (L:W). **Ceph** 1.0 L:W, 0.17× BL, rostrum with three small teeth. **Pereon** 3.0 L:W, 0.6× BL. **Prn** 1–6: 0.4, 0.4, 0.5, 0.6, 0.5, 0.4 L:W, respectively, each with lateral and distal setae. **Pleon** with **Plt** 0.23× BL. **Pl** 1–5 same size, 0.2 L:W. **Plt** with rounded apex.

**Figure 54.**
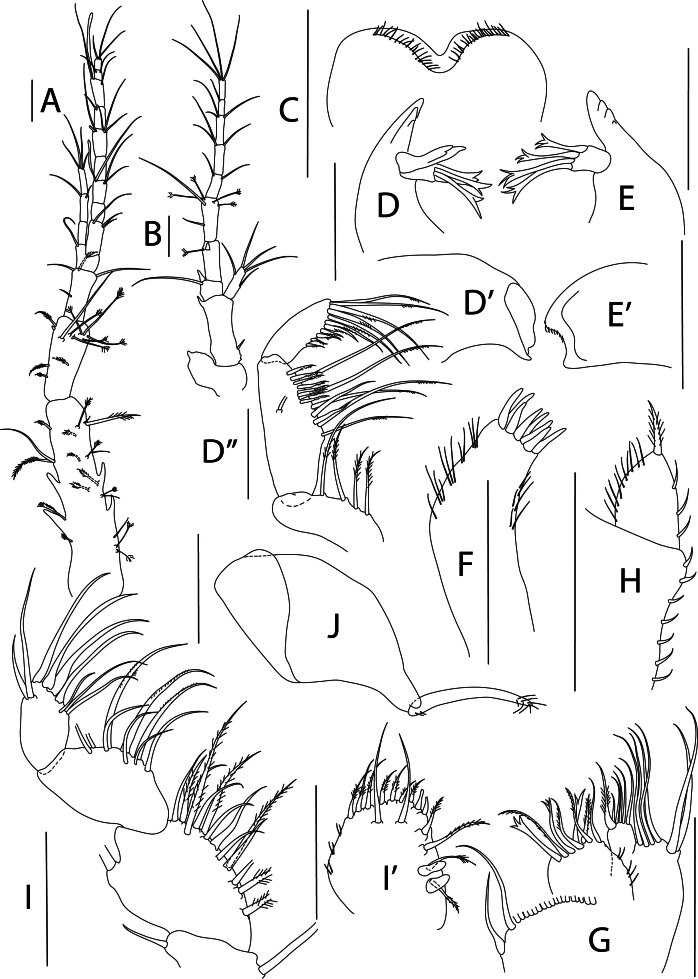
*Hoplopolemius olo* Jóźwiak & Stępień, **sp. nov.** Non-ovigerous female, paratype (SMF 57073). **A** Antennule; **B** Antenna; **C** Labrum; **D** Left mandible; **D**’ Molar of left mandible; **D**’’ Palp of left mandible; **E** Right mandible; **E**’ Molar of right mandible; **F** Maxillule endite; **G** Maxilla; **H** Labium; **I** Maxilliped; **I**’ Endite of maxilliped; **J** Epignath. Scale bars: 0.1 mm.

**A1** (Fig. 54A): articles with simple and broom setae; art1 5.2 L:W, 2.5× art2, with two apophyses on each outer and inner margin; art2 2.3 L:W, 1.7× art3; art3 2.5 L:W; acc flag with three articles; outer flag with nine articles, art3 and art7 with aesthetascs.

**A2** (Fig. 54B): articles with simple and broom setae, art1 with broad inner apophysis; art2 3 L:W, with distal inner spine, squama with two lateral and three distal setae; art3 0.8 L:W, with inner distal spine; art4 and art5 2.5 L:W each, art4 with distal spine; flagellum with five articles.

Mouthparts. **Lbr** (Fig. 54C) setose. **Left md** (Fig. 54D) incisor denticulated, lacinia mobilis with two denticles, setal row of four trifurcated setae, molar broad (Fig. 54D’); palp with three articles, each with row of plumose setae (Fig. 54D’’). **Right md** (Fig. 54E) incisor with four denticles, setal row of three trifurcated setae, molar broad (Fig. 54E’). **Mx1** (Fig. 54F) outer endite with eight terminal spines. **Mx2** (Fig. 54G) typical for suborder. **Lb** (Fig. 54H) lobe with one terminal setae; lobe and lateral margin with row of spines. **Mxp** (Fig. 54I) art1 with distal setae; art2 with row of inner setae and one strong outer seta; art3 and art4 with row of inner setae, two subdistal setae on art4. **Endite** (Fig. 54I’) with row of lateral and distal setae, two subdistal setae and two coupling hooks. **Epignath** (Fig. 54J).

**Figure 55.**
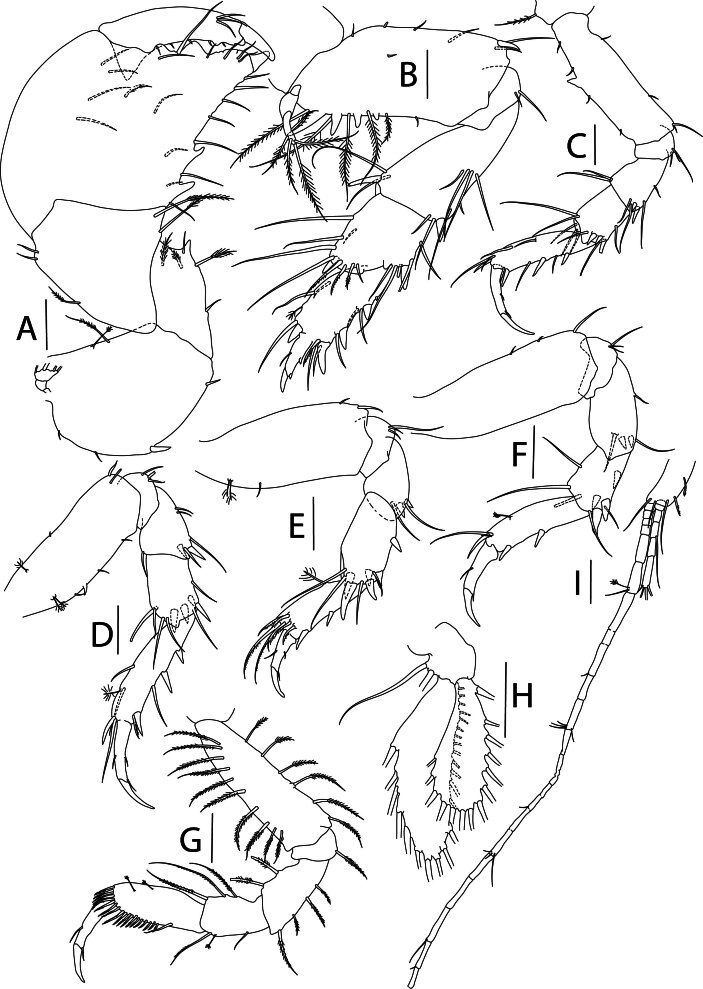
*Hoplopolemius olo* Jóźwiak & Stępień, **sp. nov.** Non-ovigerous female, paratype (SMF 57073). **A** Cheliped; **B** Pereopod 1; **C** Pereopod 2; **D** Pereopod 3; **E** Pereopod 4; **F** Pereopod 5; **G** Pereopod 6; **H** Pleopoda; **I** Uropoda. Scale bars: 0.1 mm.

**Ch** (Fig. 55A) basis 1.5 L:W, with ventral spine; merus with ventral apophysis at mid-length; carpus 1.3 L:W; propodus palm 1.0 L:W, with two ventral apophysis and nine inner setae at the surface; fixed finger 2.0 L:W, with row of inner and ventral setae; dactylus little longer than fixed finger, with three distal setae and row of small inner spines. Exopod with four distal setae.

**P1** (Fig. 55B) longer than the other pereopods, setose; basis with three dorsal apophysis and ventral distal spine; ischium with two setae; merus and carpus with ventral and dorsal spines distally; propodus with four spines along ventral margin and dorsal spine distally; dactylus with unguis 0.7× propodus, dactylus with two ventral apophysis. Exopod with five setae. **P2** (Fig. 55C) setose; basis with dorsal apophysis; merus with two ventral spines; carpus with three ventral spines; propodus with three ventral spines and dorsal spine distally; dactylus with unguis similar in length to propodus, with two ventral spines. **P3** (Fig. 55D) similar to P2, but carpus with four spines (three inner). **P4** (Fig. 55E) setose; merus with two ventral spines; carpus with ventral spine and five spines (two inner) along distal margin; propodus with two ventral spines; dactylus with two ventral spines. **P5** (Fig. 55F) similar to P4, but merus with two inner spines and carpus with four spines (two inner). **P6** (Fig. 55G) basis, merus and carpus with row of plumose setae along ventral and dorsal margins; propodus with row of short plumose setae along ventral and distal margin.

**Plp** (Fig. 55H) biramous, five pairs, all similar; basis with two plumose setae; exopod with 14 setae; endopod with plumose 23 setae.

**U** (Fig. 55I) exopod with five art; endopod (broken) with at least 22 art.

##### Type material

Holotype, ovigerous female (SMF 57072) and paratype, non-ovigerous dissected on slides (SMF 57073).

##### Material examined

Two specimens - holotype (SMF 57072) and paratype (SMF 57073).

##### Type locality

North-eastern tropical Atlantic Ocean, Gulf of Guinea, off Ghanaian coast, 4°45'52.9"N, 2°07'57.7"W, 28 m depth.

#### Diagnosis

Rostrum with three small teeth; smooth lateral margins of Plt; dorsal apophysis on basis of P2–3.

#### Etymology

"Olo" in Polish is a diminutive form of Aleksander. This species is dedicated to Aleksander Jóźwiak, the beloved son of Piotr Jóźwiak and a great (and brave) companion on local and more distant field trips.

#### Distribution

West Africa, Gulf of Guinea.

#### Taxon discussion

The presence of a multi-articulate inner flagellum on the A1, which is shorter than the outer flagellum, along with five pairs of pleopods, are key diagnostic characters of the genus *Hoplopolemius*, as described by Jóźwiak and Błażewicz (2021). The newly-described species represents the fourth known member of the genus and the first recorded from the East Atlantic Ocean. *Hoplopolemius propinquus* and *H. triangulatus* were previously reported from Bermuda (Richardson 1902), while *H. toyoshious* was described from waters near Japan (Larsen and Shimomura 2006).

The new species can be distinguished from its congeners by the following morphological features: (1) the rostrum, which bears three small teeth, whereas it is pointed in other species; (2) the absence of an outer distal spine or apophysis on art1 of A1, a feature present in other species; and (3) the structure of the cheliped, with a fixed finger bearing two ventral apophyses and the basis of P2–3 having a dorsal apophysis. In contrast, the ventral margin of the fixed finger and the dorsal margin of the P2–3 basis are smooth in the other three species.

*Hoplopolemius olo* can also be distinguished from other representatives of the subfamily Chondropodinae occurring along the West African coast, namely *Calozodion pabisi* Jakiel & Jóźwiak, 2015 (Jakiel et al. 2015) and *C. dominiki* Bochert, 2012 (Bochert 2012), by its three-denticulated rostrum. In *C. pabisi*, the rostrum is flat and multidenticulated, whereas in *C. dominiki*, it is triangular, wide and has smooth margins. Additionally, the new species exhibits smooth lateral margins of the pleotelson, whereas *C. pabisi* and *C. dominiki* have lateral apophyses.

#### Methods

The samples were collected in October and November 2012 from the Gulf of Guinea, western Africa, from the R/V *DR. FRIDTJOF NANSEN*, with use of a 0.1 m² van Veen grab. The collected material was sieved through a 0.3 mm mesh and sorted in the laboratory. The paratype of *Hoplopolemius* was dissected with needles, mounted in glycerine on slides and sealed with melted paraffin. Illustrations were initially made using a microscope, equipped with a camera lucida and were subsequently re-drawn digitally using a graphic tablet, following the method described by Coleman (2003).

**Repository**: The type material is deposited at the Senckenberg Natural History Museum in Frankfurt (SMF).

### Nesotanais thalassinus

Stępień sp. nov.

01625A84-DABC-5577-9C20-689AAD41D407

8B54027C-ACDF-46B1-81C0-1FC9676C9168

#### Materials

- **Type status:** Holotype. **Occurrence:** occurrenceDetails: Great Barrier reef, Lizard Island, Coconut beach; catalogNumber: W60130; occurrenceRemarks: coral rubble - coarse; individualCount: 1; sex: female; lifeStage: preadult; reproductiveCondition: non-ovigerous; establishmentMeans: native; occurrenceStatus: present; occurrenceID: 6D4B5965-C019-5186-ADF9-CDAA8945EA1E; **Taxon:** scientificName: *Nesotanais thalassinus* Stępień; kingdom: Animalia; phylum: Arthropoda; class: Malacostraca; order: Tanaidacea; family: Nototanaidae; genus: Nesotanais; specificEpithet: thalassinus; taxonRank: species; scientificNameAuthorship: Stępień; nomenclaturalCode: ICZN; **Location:** higherGeography: Pacific Ocean; waterBody: Southeast Pacific Ocean; island: Lizard Island; country: Australia; countryCode: AU; locality: Great Barrier Reef, station CGLI 003; verbatimDepth: 2 m; verbatimLatitude: 14°41'28.2"S; verbatimLongitude: 145°28'11.3"E; decimalLatitude: -14.69117; decimalLongitude: 145.4698; **Identification:** identifiedBy: Anna Stępień; dateIdentified: 2025; **Event:** samplingProtocol: scuba diving, sieved throught 0.3 mm mesh; eventDate: 04-05-2008; habitat: coral rubble - coarse; fieldNumber: CGLI 003; **Record Level:** institutionID: https://www.museum.qld.gov.au/tropics; institutionCode: QMT; basisOfRecord: PreservedSpecimen
- **Type status:** Paratype. **Occurrence:** occurrenceDetails: Great Barrier reef, Lizard Island, Hicks Reef; catalogNumber: QMT W60131; occurrenceRemarks: dead coral heads on spur; individualCount: 1; sex: male; lifeStage: adult; establishmentMeans: native; occurrenceStatus: present; occurrenceID: FE49FC73-1E49-5F36-9FAC-2B455068960C; **Taxon:** scientificName: Nesotanais thalassinus Stępień; kingdom: Animalia; phylum: Arthropoda; class: Malacostraca; order: Tanaidacea; family: Nototanaidae; genus: Nesotanais; specificEpithet: thalassinus; taxonRank: species; scientificNameAuthorship: Stępień; nomenclaturalCode: ICZN; taxonRemarks: used as allotype; **Location:** higherGeography: Pacific Ocean; waterBody: Southeast Pacific Ocean; island: Lizard Island; country: Australia; countryCode: AU; locality: Great Barrier Reef, station LIZ09-16D; verbatimDepth: 16 m; verbatimLatitude: 14°26'52.9"S; verbatimLongitude: 145°29'57.1"E; decimalLatitude: -14.44803; decimalLongitude: 145.4992; **Identification:** identifiedBy: Anna Stępień; dateIdentified: 2025; **Event:** samplingProtocol: scuba diving, sieved throught 0.3 mm mesh; eventDate: 21-02-2009; habitat: dead coral heads on spur; fieldNumber: LIZ 09-16D; **Record Level:** institutionID: https://www.museum.qld.gov.au/tropics; institutionCode: QMT; basisOfRecord: PreservedSpecimen
- **Type status:** Paratype. **Occurrence:** occurrenceDetails: Great Barrier reef, Lizard Island, Hicks Reef; catalogNumber: QMT W60135; occurrenceRemarks: dead coral heads on spur; individualCount: 2; sex: female; lifeStage: preadult; reproductiveCondition: non-ovigerous; establishmentMeans: native; occurrenceStatus: present; occurrenceID: DA129350-B0E0-5431-823A-BB234F56831A; **Taxon:** scientificName: *Nesotanais thalassinus* Stępień; kingdom: Animalia; phylum: Arthropoda; class: Malacostraca; order: Tanaidacea; family: Nototanaidae; genus: Nesotanais; specificEpithet: thalassinus; taxonRank: species; scientificNameAuthorship: Stępień; nomenclaturalCode: ICZN; **Location:** higherGeography: Pacific Ocean; waterBody: Southeast Pacific Ocean; island: Lizard Island; country: Australia; countryCode: AU; locality: Great Barrier Reef, station LIZ09-16D; verbatimDepth: 16 m; verbatimLatitude: 14°26'52.9"S; verbatimLongitude: 145°29'57.1"E; decimalLatitude: -14.44803; decimalLongitude: 145.4992; **Identification:** identifiedBy: Anna Stępień; dateIdentified: 2025; **Event:** samplingProtocol: scuba diving, sieved throught 0.3 mm mesh; eventDate: 21-02-2009; habitat: dead coral heads on spur; fieldNumber: LIZ 09-16D; **Record Level:** institutionID: https://www.museum.qld.gov.au/tropics; institutionCode: QMT; basisOfRecord: PreservedSpecimen
- **Type status:** Paratype. **Occurrence:** occurrenceDetails: Great Barrier reef, Lizard Island, Yonge Reef; catalogNumber: QMT W60136; occurrenceRemarks: small coral rubble on sand; individualCount: 1; sex: female; lifeStage: preadult; reproductiveCondition: non-ovigerous; establishmentMeans: native; occurrenceStatus: present; occurrenceID: BA9EFA71-15DA-5D69-A018-D809D5DBFD81; **Taxon:** scientificName: Nesotanais thalassinus Stępień; kingdom: Animalia; phylum: Arthropoda; class: Malacostraca; order: Tanaidacea; family: Nototanaidae; genus: Nesotanais; specificEpithet: thalassinus; taxonRank: species; scientificNameAuthorship: Stępień; nomenclaturalCode: ICZN; **Location:** higherGeography: Pacific Ocean; waterBody: Southeast Pacific Ocean; island: Lizard Island; country: Australia; countryCode: AU; locality: Great Barrier Reef, station LIZ 09-10F; verbatimDepth: 15 m; verbatimLatitude: 14°36'49.8"S; verbatimLongitude: 145°37'05.5"E; decimalLatitude: -14.61383; decimalLongitude: 145.6182; **Identification:** identifiedBy: Anna Stępień; dateIdentified: 2025; **Event:** samplingProtocol: scuba diving, sieved throught 0.3 mm mesh; eventDate: 18-02-2009; habitat: small coral rubble on sand; fieldNumber: LIZ 09-10F; **Record Level:** institutionID: https://www.museum.qld.gov.au/tropics; institutionCode: QMT; basisOfRecord: PreservedSpecimen
- **Type status:** Paratype. **Occurrence:** occurrenceDetails: Great Barrier reef, Lizard Island; occurrenceRemarks: coral rubble; individualCount: 1; sex: female; lifeStage: preadult; reproductiveCondition: non-ovigerous; establishmentMeans: native; occurrenceStatus: present; occurrenceID: AFC89B5B-D047-55F4-A501-5E566B06C215; **Taxon:** scientificName: Nesotanais thalassinus Stępień; kingdom: Animalia; phylum: Arthropoda; class: Malacostraca; order: Tanaidacea; family: Nototanaidae; genus: Nesotanais; specificEpithet: thalassinus; taxonRank: species; scientificNameAuthorship: Stępień; nomenclaturalCode: ICZN; **Location:** higherGeography: Pacific Ocean; waterBody: Southeast Pacific Ocean; island: Lizard Island; country: Australia; countryCode: AU; locality: Great Barrier Reef, station CGLI 025A; verbatimDepth: 12 m; verbatimLatitude: 14°38'44.4"S; verbatimLongitude: 145°27'11.7"E; decimalLatitude: -14.64567; decimalLongitude: 145.45325; **Identification:** identifiedBy: Anna Stępień; dateIdentified: 2025; **Event:** samplingProtocol: scuba diving, sieved throught 0.3 mm mesh; eventDate: 14-04-2008; habitat: coral rubble; fieldNumber: CGLI 025A; **Record Level:** institutionID: https://www.museum.qld.gov.au/tropics; institutionCode: QMT; basisOfRecord: PreservedSpecimen
- **Type status:** Paratype. **Occurrence:** occurrenceDetails: Great Barrier reef, Lizard Island; catalogNumber: QMT W60134; occurrenceRemarks: dead coral heads on reef edge; individualCount: 3; sex: female; lifeStage: preadult; reproductiveCondition: non-ovigerous; establishmentMeans: native; occurrenceStatus: present; occurrenceID: E8684079-496A-5108-97AE-7AB7C4A52EC5; **Taxon:** scientificName: Nesotanais thalassinus Stępień; kingdom: Animalia; phylum: Arthropoda; class: Malacostraca; order: Tanaidacea; family: Nototanaidae; genus: Nesotanais; specificEpithet: thalassinus; taxonRank: species; scientificNameAuthorship: Stępień; nomenclaturalCode: ICZN; **Location:** higherGeography: Pacific Ocean; waterBody: Southeast Pacific Ocean; island: Lizard Island; country: Australia; countryCode: AU; locality: Great Barrier Reef, station LIZ09-16E; verbatimDepth: 5-7 m; verbatimLatitude: 14°26'52.9"S; verbatimLongitude: 145°29'57.1"E; decimalLatitude: -14.44803; decimalLongitude: 145.4992; **Identification:** identifiedBy: Anna Stępień; dateIdentified: 2025; **Event:** samplingProtocol: scuba diving, sieved throught 0.3 mm mesh; eventDate: 21-02-2009; habitat: dead coral heads on reef edge; fieldNumber: LIZ 09-16E; **Record Level:** institutionID: https://www.museum.qld.gov.au/tropics; institutionCode: QMT; basisOfRecord: PreservedSpecimen
- **Type status:** Paratype. **Occurrence:** occurrenceDetails: Great Barrier reef, Lizard Island; catalogNumber: QMT W60137; occurrenceRemarks: dead Pocillopora head; individualCount: 4; sex: female; lifeStage: preadult; reproductiveCondition: non-ovigerous; establishmentMeans: native; occurrenceStatus: present; occurrenceID: 276EFB7B-7369-5853-B0C1-FFB91E960EA4; **Taxon:** scientificName: Nesotanais thalassinus Stępień; kingdom: Animalia; phylum: Arthropoda; class: Malacostraca; order: Tanaidacea; family: Nototanaidae; genus: Nesotanais; specificEpithet: thalassinus; taxonRank: species; scientificNameAuthorship: Stępień; nomenclaturalCode: ICZN; **Location:** higherGeography: Pacific Ocean; waterBody: Southeast Pacific Ocean; island: Lizard Island; country: Australia; countryCode: AU; locality: Great Barrier Reef, station CGLI 008A; verbatimDepth: 8 m; verbatimLatitude: 14°38'55.4"S; verbatimLongitude: 145°26'59.5"E; decimalLatitude: -14.64872; decimalLongitude: 145.44987; **Identification:** identifiedBy: Anna Stępień; dateIdentified: 2025; **Event:** samplingProtocol: scuba diving, sieved throught 0.3 mm mesh; eventDate: 06-04-2008; habitat: dead Pocillopora head; fieldNumber: CGLI 008A; **Record Level:** institutionID: https://www.museum.qld.gov.au/tropics; institutionCode: QMT; basisOfRecord: PreservedSpecimen
- **Type status:** Paratype. **Occurrence:** occurrenceDetails: Great Barrier reef, Lizard Island; catalogNumber: QMT W60138; occurrenceRemarks: Halimeda and rubble; individualCount: 1; sex: female; lifeStage: preadult; reproductiveCondition: non-ovigerous; establishmentMeans: native; occurrenceStatus: present; occurrenceID: 5381E519-2B75-5506-B2A9-16CFFF4CDF8D; **Taxon:** scientificName: *Nesotanais thalassinus* Stępień; kingdom: Animalia; phylum: Arthropoda; class: Malacostraca; order: Tanaidacea; family: Nototanaidae; genus: Nesotanais; specificEpithet: thalassinus; taxonRank: species; scientificNameAuthorship: Stępień; nomenclaturalCode: ICZN; **Location:** higherGeography: Pacific Ocean; waterBody: Southeast Pacific Ocean; island: Lizard Island; country: Australia; countryCode: AU; locality: Great Barrier Reef, station CGLI 012; verbatimDepth: 3 m; verbatimLatitude: 14°41'13.5"S; verbatimLongitude: 145°27'56.2"E; decimalLatitude: -14.68708; decimalLongitude: 145.4656; **Identification:** identifiedBy: Anna Stępień; dateIdentified: 2025; **Event:** samplingProtocol: scuba diving, sieved throught 0.3mm mesh; eventDate: 07-04-2008; habitat: Halimeda and rubble; fieldNumber: CGLI 012; **Record Level:** institutionID: https://www.museum.qld.gov.au/tropics; institutionCode: QMT; basisOfRecord: PreservedSpecimen
- **Type status:** Paratype. **Occurrence:** occurrenceDetails: Great Barrier reef, Lizard Island; catalogNumber: QMT W60132; individualCount: 8; sex: female; lifeStage: preadult; reproductiveCondition: non-ovigerous; establishmentMeans: native; occurrenceStatus: present; occurrenceID: 5D065D9D-0771-5933-8514-B6FE5F9990A2; **Taxon:** scientificName: *Nesotanais thalassinus* Stępień; kingdom: Animalia; phylum: Arthropoda; class: Malacostraca; order: Tanaidacea; family: Nototanaidae; genus: Nesotanais; specificEpithet: thalassinus; taxonRank: species; scientificNameAuthorship: Stępień; nomenclaturalCode: ICZN; **Location:** higherGeography: Pacific Ocean; waterBody: Southeast Pacific Ocean; island: Lizard Island; country: Australia; countryCode: AU; locality: Great Barrier Reef, station LIZ09-02B; verbatimDepth: 13 m; verbatimLatitude: 14°22'13.3"S; verbatimLongitude: 145°21'56.9"E; decimalLatitude: -14.37036; decimalLongitude: 145.3658; **Identification:** identifiedBy: Anna Stępień; dateIdentified: 2025; **Event:** samplingProtocol: scuba diving, sieved throught 0.3 mm mesh; eventDate: 24-02-2009; habitat: coral ruble; fieldNumber: LIZ 09-02B; **Record Level:** institutionID: https://www.museum.qld.gov.au/tropics; institutionCode: QMT; basisOfRecord: PreservedSpecimen
- **Type status:** Paratype. **Occurrence:** occurrenceDetails: Great Barrier reef, Lizard Island; catalogNumber: QMT W60139; occurrenceRemarks: rubble; individualCount: 1; sex: female; lifeStage: preadult; reproductiveCondition: non-ovigerous; establishmentMeans: native; occurrenceStatus: present; occurrenceID: 21D09A8C-D5F5-54EA-8EAE-A4DA5D211317; **Taxon:** scientificName: *Nesotanais thalassinus* Stępień; kingdom: Animalia; phylum: Arthropoda; class: Malacostraca; order: Tanaidacea; family: Nototanaidae; genus: Nesotanais; specificEpithet: thalassinus; taxonRank: species; scientificNameAuthorship: Stępień; nomenclaturalCode: ICZN; **Location:** higherGeography: Pacific Ocean; waterBody: Southeast Pacific Ocean; island: Lizard Island; country: Australia; countryCode: AU; locality: Great Barrier Reef, station CGLI046C; verbatimDepth: 15 m; verbatimLatitude: 14°34'47.0"S; verbatimLongitude: 145°36'36.4"E; decimalLatitude: -14.57972; decimalLongitude: 145.6101; **Identification:** identifiedBy: Anna Stępień; dateIdentified: 2025; **Event:** samplingProtocol: scuba diving, sieved throught 0.3 mm mesh; eventDate: 20-04-2008; habitat: rubble; fieldNumber: CGLI046C; **Record Level:** institutionID: https://www.museum.qld.gov.au/tropics; institutionCode: QMT; basisOfRecord: PreservedSpecimen
- **Type status:** Paratype. **Occurrence:** occurrenceDetails: Great Barrier reef, Lizard Island; catalogNumber: QMT W60140; occurrenceRemarks: no data; individualCount: 1; sex: male; lifeStage: adult; establishmentMeans: native; occurrenceStatus: present; occurrenceID: 0ED2B6E6-336B-5A30-957E-B2871D5C05B5; **Taxon:** scientificName: *Nesotanais thalassinus* Stępień; kingdom: Animalia; phylum: Arthropoda; class: Malacostraca; order: Tanaidacea; family: Nototanaidae; genus: Nesotanais; specificEpithet: thalassinus; taxonRank: species; scientificNameAuthorship: Stępień; nomenclaturalCode: ICZN; **Location:** higherGeography: Pacific Ocean; waterBody: Southeast Pacific Ocean; island: Lizard Island; country: Australia; countryCode: AU; locality: Great Barrier Reef, station CGLI018B; verbatimDepth: 2 m; verbatimLatitude: 14°41'20.4"S; verbatimLongitude: 145°28'12.0"E; decimalLatitude: -14.689; decimalLongitude: 145.47; **Identification:** identifiedBy: Anna Stępień; dateIdentified: 2025; **Event:** samplingProtocol: scuba diving, sieved throught 0.3 mm mesh; eventDate: 2008; habitat: no data; fieldNumber: CGLI018B; **Record Level:** institutionID: https://www.museum.qld.gov.au/tropics; institutionCode: QMT; basisOfRecord: PreservedSpecimen

#### Description

**Figure 56.**
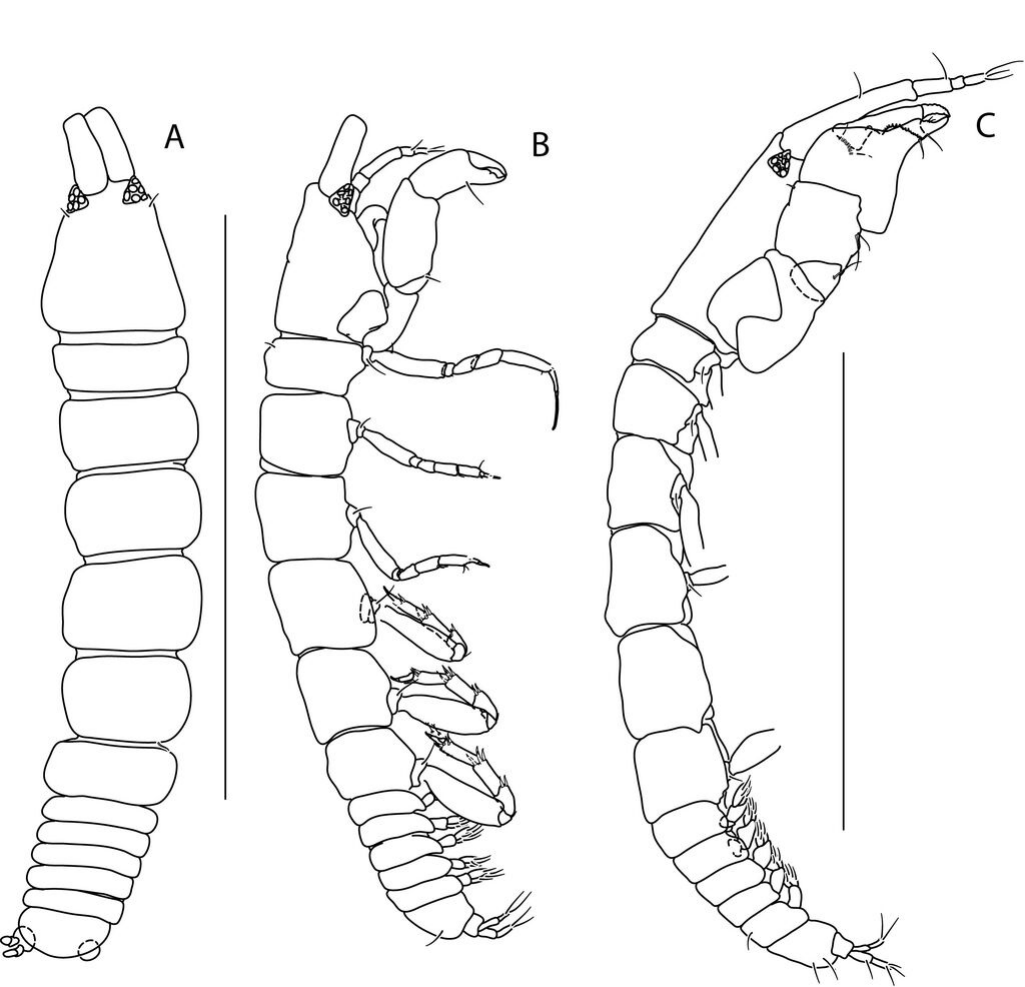
*Nesotanais thalassinus* Stępień, **sp. nov.** Non-ovigerous female holotype [W60130], habitus. **A** Dorsal view; **B** Lateral view. Adult male allotype [W60131], habitus; **C** Lateral view. Scale bars: 1 mm.

**Non-ovigerous female holotype**: Habitus (Fig. 56A, B). Body length (BL) = 1.3 mm, 5.4 times as long as wide (L:W). **Ceph** 1.2 L:W, 0.2× BL, pear-shaped, with pair of proximal setae. **Pereon** 3.5 L:W, 0.6× BL. **Prn** 1–6: 0.3, 0.4, 0.5, 0.6, 0.6, 0.4 L:W, respectively. **Pleon** with **Plt** 0.2× BL. **Pl** 1–5 same size, 0.2 L:W. **Plt** 0.5 L:W, with rounded apex.

**Figure 57.**
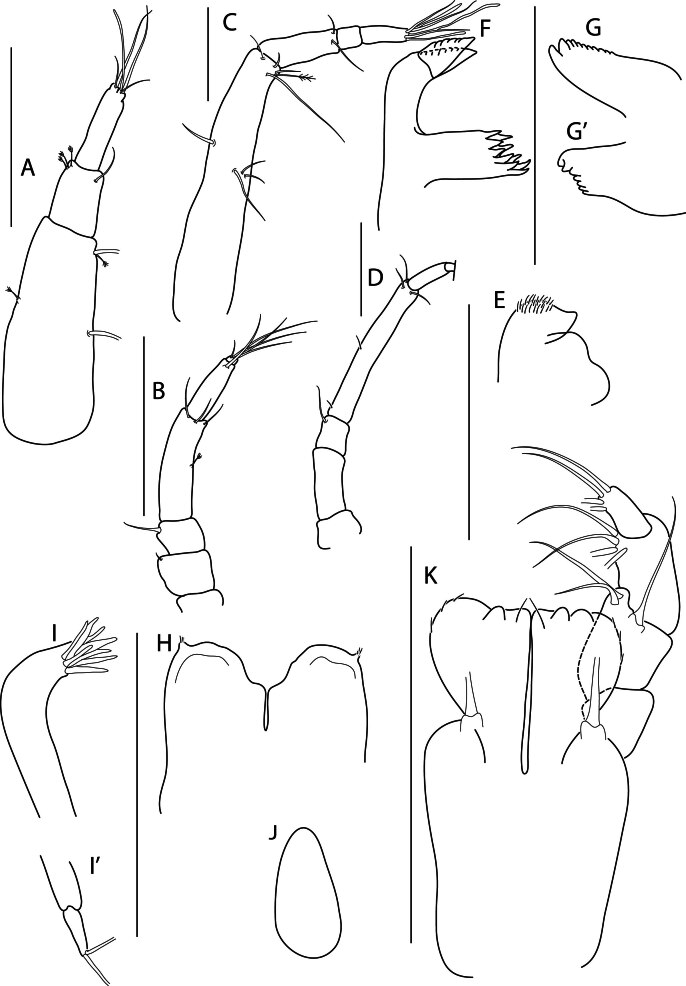
*Nesotanais thalassinus* Stępień, **sp. nov.** Non-ovigerous female paratype [W60139] (A–B, F–K), male paratype [W60140] (C–D). **A** A1; **B** A2; **C** A1; **D** A2; **E** Lbr; **F** Left Md; **G** Right Md; **G**’ Molar of right Md; **H** Lbi; **I** Mx1 endite; **I**’ Mx1 palp; **J** Mx2; **K** Mxp. Scale bars: 0.1 mm.

**A1** (Fig. 57A): art1 2.6 L:W, 3.6× art2, with two simple and two broom setae; art2 1.2 L:W, 0.7× art3, with three broom and three simple setae; art3 3.1 L:W, with aesthetasc and four simple setae.

**A2** (Fig. 57B): art2 0.7 L:W, 1.2× art3, with simple seta; art3 0.6 L:W, 0.3× art4, with inner seta; art4 5.0 L:W, 1.3× art4, with broom seta and three simple setae; art5 3.2 L:W, with simple seta; art6 minute, with five setae.

Mouthparts. **Lbr** (Fig. 57E) hood-shaped, setose. **Left md** (Fig. 57F) incisor denticulated, lacinia mobilis as large as incisor, molar broad. **Right md** (Fig. 57G, G’) incisor denticulated, molar broad. **Lb** (Fig. 57H) bilobated, setose on distal corners. **Mx1** (Fig. 57I, I’) endite with eight distal spines, palp with two setae. **Mx2** (Fig. 57J) ovate. **Mxp** (Fig. 57K) basis with simple seta; art1 naked; art2 with three setae; art3 and art4 with four setae each. Endite with one, two rounded tubercles and simple seta distally.

**Figure 58.**
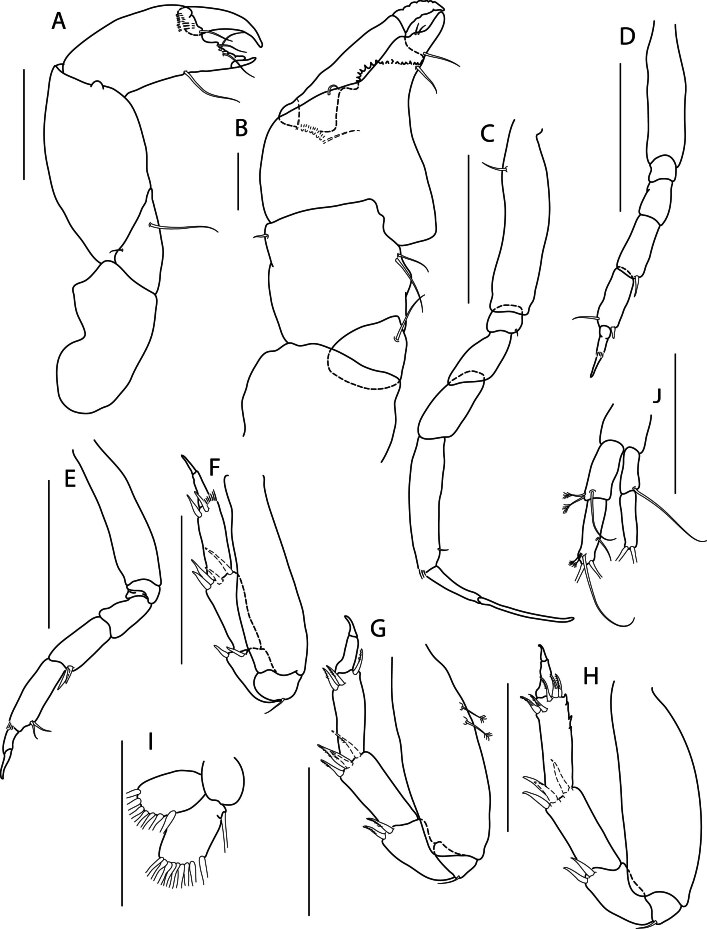
*Nesotanais thalassinus* Stępień, **sp. nov.**, non-ovigerous female paratype [W60139] (A, C–J), male paratype [W60140] (B). **A** Ch; **B** Ch; **C** P1; **D** P2; **E** P3; **F** P4; **G** P5; **H** P6; **I** Plp; **J** U. Scale bars: 0.1 mm.

**Ch** (Fig. 58A) basis 1.5 L:W; merus with simple ventral seta and short seta at mid-surface; carpus 1.8 L:W; propodus palm 2.0 L:W, with proximal seta and row of inner setae near dactylus insertion; fixed finger 1.1 L:W, with three inner setae and ventral seta; dactylus little longer than fixed finger.

**P1** (Fig. 58C) basis with simple seta; ischium with simple seta; propodus with simple subdistal setae and three setae near dactylus insertion; dactylus with unguis 1.2× propodus. **P2** (Fig. 58D) carpus with thin and long distal spine; propodus with subdistal seta and distal spine. **P3** (Fig. 58E) carpus with two elongated distal spines; propodus with two subdistal setae and distal comb of short setae near dactylus insertion; **P4** (Fig. 58F) ischium with seta; merus with two elongated distal spines; carpus with four elongated distal spines (two located on inner side); carpus with two distal spines and comb of short setae near dactylus insertion. **P5** (Fig. 58G) similar to P4, but basis with two broom setae and propodus with three disatl spines. **P6** (Fig. 58H) similar to P4, but propodus with three setulose setae near dactylus insertion.

**Plp** (Fig. 58I) biramous, five pairs, all similar; basis naked; exopod with ten inner setae; endopod with seven inner setae.

**U** (Fig. 58J) exopod with two articles, each with simple setae; endopod with two articles, each with broom and simple setae.

**Male paratype (allotype)**. Habitus (Fig. 56C) Body length (BL) = 2.0 mm. **Ceph** 0.2× BL. **Pereon** 0.6× BL. **Pleon** with **Plt** 0.2× BL.

**A1** (Fig. 57C) art1 5.8 L:W, 4.0× atr2, with eight simple setae and broom seta; art2 2.6 L:W, 3.0× art3, with simple setae; art3 as long as wide; art4 with three aesthetascs.

**A2** (Fig. 57D) art1 0.7 L:W, 0.4× art2; art2 2.0 L:W, 2.0× art3; art3 as long as wide, 0.2× art4, with setae on dorsodistal corner; art4 6.4 L:W, 3.7×art5, with five simple setae; art 5 4.0 L:W, art6 broken.

**Ch** (Fig. 58B) merus with simple ventral seta; carpus quadrate, with two ventral and dorsal setae; propodus strongly dilated downwards to form a flange in ventral edge, with simple proximal setae and row of inner setae near dactylus insertion, fixed finger with two ventral and two inner setae; dactylus with two inner tooth (small and large) and crenulated inner margin.

##### Type material

Holotype (QMT W60130) and paratype (allotype; QMT W60131), collected from Lizard Island, Great Barrier Reef, Australia, at depths of 2–16 m in 2008–2009. The holotype is a pre-adult female; the allotype is an adult male. Paratypes (QMT W60132–W60140) include pre-adult females and two males from multiple stations around Lizard Island (2–16 m), collected from coral rubble, dead coral heads, *Pocillopora* heads and associated rubble habitats.

##### Material examined

Holotype (QMT W60130), ♀ pre-adult, non-ovigerous, Great Barrier Reef, Lizard Island, Coconut Beach, Station CGLI 003, 2 m depth, 14°41′28.2″S, 145°28′11.3″E, coral rubble, collected by scuba diving, 4 May 2008.

Paratype (allotype; QMT W60131), ♂ adult, Great Barrier Reef, Lizard Island, Hicks Reef, Station LIZ09-16D, 16 m depth, 14°26′52.9″S, 145°29′57.1″E, dead coral heads on spur, collected by scuba diving, 21 February 2009.

Paratypes.

- two ♀♀ pre-adults, non-ovigerous (QMT W60135), same data as allotype (LIZ09-16D, 16 m depth, 21 Feb 2009).
- one ♀ pre-adult, non-ovigerous (QMT W60133), Lizard Island, Yonge Reef, Station LIZ09-10F, 15 m depth, 14°36′49.8″S, 145°37′05.5″E, small coral rubble on sand, 18 Feb 2009.
- one ♀ preadult, non-ovigerous (QMT W60136), Lizard Island, Station CGLI 025A, 12 m depth, 14°38′44.4″S, 145°27′11.7″E, coral rubble, 14 Apr 2008.
- three ♀♀ pre-adults, non-ovigerous (QMT W60134), Lizard Island, Hicks Reef, station LIZ09-16E, 5–7 m depth, 14°26′52.9″S, 145°29′57.1″E, dead coral heads on reef edge, 21 Feb 2009.
- four ♀♀ pre-adults, non-ovigerous (QMT W60137), Lizard Island, Station CGLI 008A, 8 m depth, 14°38′55.4″S, 145°26′59.5″E, dead *Pocillopora* head, 6 Apr 2008.
- one ♀ pre-adult, non-ovigerous (QMT W60138), Lizard Island, Station CGLI 012, 3 m depth, 14°41′13.5″S, 145°27′56.2″E, *Halimeda* and rubble, 7 Apr 2008.
- eight ♀♀ pre-adults, non-ovigerous (QMT W60132), Lizard Island, Station LIZ09-02B, 13 m depth, 14°22′13.3″S, 145°21′56.9″E, small coral rubble, 24 Feb 2009.
- one ♀ pre-adult, non-ovigerous (QMT W60139), Lizard Island, Station CGLI 046C, 15 m depth, 14°34′47.0″S, 145°36′36.4″E, coral rubble, dissected specimen, 20 Apr 2008.
- one ♂ adult (QMT W60140), Lizard Island, Station CGLI 018B, 2 m depth, 14°41′20.4″S, 145°28′12.0″E, no habitat data, dissected specimen, 2008.

##### Type locality

Southeast Pacific Ocean, Great Barrier Reef, Lizard Island, Coconut Beach, 2–16 m depth.

#### Diagnosis

A1 art1 with a length-to-width ratio of 2.6; A2 art2 only with short seta; elongated cheliped propodus, with a length-to-width ratio of 2.0; male with smooth cheliped surface.

#### Etymology

The species name *thalassinus*, derived from Latin meaning "marine", refers to the environmental preference of this species for fully saline seawater, while most representatives of *Nesotanais* are associated with brackish habitats.

#### Distribution

Great Barrier Reef, Australia.

#### Taxon discussion

The species described herein represents the fifth known member of the genus *Nesotanais*, the first from the vicinity of Australia and the second from fully saline habitats. *Nesotanais lacustris* is known from the brackish Lake Tegano on Rennell Island, Solomon Islands (Shiino 1968), *N. maclaughlinae* from the vicinity of a marine cave at Palau Island in Micronesia (Guţu and Iliffe 1989), *N. rugula* from the brackish Songkhla Lake in Thailand (Bamber et al. 2003) and *N. ryukyuensis* from a brackish river on Okinawa Island (Kakui et al. 2010).

The female of the new *Nesotanais* species can be distinguished from its congeners by a relatively wide art1 of A1, with a length-to-width (L:W) ratio of 2.6 in the new species, compared to 3.5 in *N. lacustris* and *N. rugula* and 2.5 in *N. ryukyuensis*. Furthermore, the new species is characterised by an elongated cheliped propodus with an L:W ratio of 2.0, whereas it is 1.5, 1.2 and 1.2 in *N. lacustris*, *N. ryukyuensis* and *N. rugula*, respectively. Additionally, the dactylus plus unguis of P1 is relatively long, exceeding the length of the propodus, whereas, in other *Nesotanais* females, it is either shorter or equal in length.

The male of the new species can be distinguished from *N. ryukyuensis* and *N. rugula* by the absence of a series of cuticular ridges on the surface of the cheliped. Both the male and female of the new species can be further differentiated from all other *Nesotanais* species by the absence of a long, thin spine on art2 of the A2, which is present in all previously described species.

#### Methods

The samples were collected as part of the CENSUS OF CORAL REEFS project (CReefs), from the vicinity of Lizard Island at the Great Barrier Reef. Coral rubble fragments were collected manually during SCUBA diving and placed into 20-litre buckets containing either a mixture of fresh and seawater or seawater with a few drops of formaldehyde for several hours to encourage the organisms to exit their microhabitats (e.g. tubes and crevices). The samples, with live animals still present, were then washed through a fine 0.3 mm mesh and the residue was examined under a microscope. Tanaidacean specimens were subsequently collected and preserved in 80% ethanol. Specimens of *Nesotanais* were dissected with needles, mounted in glycerine on slides and sealed with the melted paraffin. Illustrations were initially made using a microscope equipped with a camera lucida and were subsequently re-drawn digitally using a graphic tablet, following the method described by Coleman (2003).

**Repository**: The type materials have been deposited at the Queensland Museum Tropics (QMT).

## Checklists

### Systematic notes and amendments

#### Laevidentalium

Cossmann, 1888

60D16CB9-6063-5F62-8A42-3EBDD82A9CCE

**Type species**: *Dentalium incertum* Deshayes, 1826, by original designation.

##### Notes

The genus *Laevidentalium* Cossmann, 1888 (Cossmann 1888, p. 7) was introduced to include dentaliid scaphopods whose shells have an oval cross-section, truncate apical opening without a notch and a smooth surface marked by growth lines only. Scarabino (1995), p. 291, provides an extended generic diagnosis including both shell and radular characters; however, as the type taxon *Dentalium incertum* Deshayes, 1826 is an Eocene fossil, the placement of all other fossil and extant scaphopod species in this conchologically rather featureless genus remains to be clarified (Scarabino and Scarabino 2011).

#### Cuspidariidae

Dall, 1886

8340B102-B821-5034-A0B5-6CBA116A869D

##### Notes

With over 260 species (~ 1/3 of all Anomalodesmata), typically found in deeper waters (30 to 7,242 m), Cuspidariidae is likely the best-studied family of carnivorous bivalves. The presence of a rostrate shell in most species generally aids in the recognition and identification of its members. Although its taxonomy is relatively well-resolved and it is consistently recovered as monophyletic in most phylogenies (Harper et al. 2006, Combosch et al. 2017, Machado and Passos 2022), the internal relationships between Cuspidariidae and other families of predatory bivalves (with or without a muscular septum) remain a subject of ongoing debate. More recently, however, broader phylogenetic studies (e.g. with a greater representation of taxa) have shown that Cuspidariidae forms a sister group with Spheniopsidae and/or Halonymphidae and Protocuspidaridae (Cuspidarioidea) (Crouch et al. 2021, Machado and Passos 2022) — diverging from previous studies where Cuspidariidae was recovered as the sister group of Poromyidae (Poromyoidea) or Verticordiidae (Verticordioidea) (Dreyer et al. 2003, Harper et al. 2006, Bieler et al. 2014, Combosch et al. 2017). These more recent findings also suggest two possible independent origins of the muscular septum in Anomalodesmata: once in Cuspidarioidea and once in Poromyoidea (Machado and Passos 2022), as well as a deep division into two distinct clades: (i) a non-carnivorous clade and (ii) a generally deep-water marine carnivorous lineage (Machado and Passos 2022, González-Delgado et al. 2024). Although well known for their shell features, more than 90% of Cuspidariidae still lack detailed anatomical studies. In this regard, *M. aleutiana* Machado & Sigwart, **sp. nov.** contributes additional knowledge to this key family, which is crucial for understanding the evolution of carnivorous bivalves.

#### Myonera

Dall & E. A. Smith, 1886

280FF894-AB96-5564-BD76-EFEF22B9C6CA

**Type species**: *Myonera paucistriata* Dall, 1886.
**Composition**: Twenty-one extant valid species, of which twenty are listed in MolluscaBase (2025) and one is newly described here. *Myonera acutecarinata* (Dautzenberg & H. Fischer, 1906), *Myonera alleni* Poutiers, 1995, *Myonera angularis* (Jeffreys, 1876), *Myonera atlasiana* Utrilla, Rueda & C. Salas, 2020, *Myonera bicarinata* E. A. Smith, 1896, *Myonera canariensis* (De Boer, 1985), *Myonera dautzenbergi* Prashad, 1932, *Myonera dispar* (Dall, Bartsch & Rehder, 1938), *Myonera garretti* Dall, 1908, *Myonera gigantea* (A. E. Verrill, 1884), *Myonera kaiwa* Oliveira & Absalão, 2009, *Myonera lamellifera* (Dall, 1881), *Myonera limatula* (Dall, 1881), *Myonera lischkei* (E. A. Smith, 1891), *Myonera pailoloana* (Dall, Bartsch & Rehder, 1938), *Myonera paucistriata* Dall, 1886, *Myonera pretiosa* A. E. Verrill & K. J. Bush, 1898, *Myonera rostra* Poutiers & F. R. Bernard, 1995, *Myonera sulcifera* (Jeffreys, 1882), *Myonera tasmanica* (Knudsen, 1970) and *Myonera aleutiana* Machado & Sigwart, **sp. nov.**

##### Diagnosis

Shell small to medium size (3.5 to 24 mm in length), thin, fragile, outline variable, inequilateral, rostrate (mostly) or posterior end truncate, usually inflated, right valve generally larger than left, with margins overlapping. Externally with concentric and/or radial ornamentation, usually covering the entire shell, starting from the umbones. Hinge plate feeble, edentate in both valves. Ligament internal, resilifer posteriorly pointed or nearly vertical; presence of lithodesma. Labial palps small. Muscular septum usually with four pairs of pores (after Knudsen (1970), Allen and Morgan (1981), Poutiers and Bernard (1995) and Coan and Valentich-Scott (2012)).

#### Metharpinia

Schellenberg, 1931

5D82CA95-B1DC-5536-B799-BCDEACE29AFD

**Type species**: *Metharpinia longirostris* Schellenberg, 1931 (type by subsequent designation).
**Composition**: Eleven valid species. *Metharpinia coronadoi* J.L. Barnard, 1980, *Metharpinia dentiurosoma* Alonso de Pina, 2003, *Metharpinia floridana* (Shoemaker, 1933), *Metharpinia grandirama* Alonso de Pina, 2003, *Metharpinia iado* Alonso de Pina, 2003, *Metharpinia jonesi* (J.L. Barnard, 1963), *Metharpinia longirostris* Schellenberg, 1931, *Metharpinia oripacifica* J.L. Barnard, 1980, *Metharpinia protuberantis* Alonso de Pina, 2001, *Metharpinia taylorae* Andrade, Johnsson & Senna, 2015 and *Metharpinia hirsuta* Souza-Filho & Andrade, **sp. nov.**

##### Diagnosis

Rostrum constricted. Eyes present. Antenna 1 article 2 elongate or of medium length, ventral setae proximally placed. Antenna 2 article 1 not ensiform, facial stout setae on article 4 in two or more rows, article 5 ordinary in size. Right mandibular incisor with 2–3 teeth; molar not triturative, with four or more splayed stout setae; palpar hump small, apex of palp article 3 oblique. Maxilla 1 inner plate with 3–4 setae, palp 2‑articulate. Maxilliped ordinary, apex of palp article 3 weakly protuberant, article 4 elongate, apical nail distinct, partially fused. Gnathopods 1–2 ordinary, small, similar; carpus with ordinary length to elongate, free, without eusirid attachment; propodus ordinary, ovatorectangular, poorly setose anteriorly; palm acute. Pereopods 3–4 carpus with posteroproximal setae; propodus with stout facial setae. Pereopod 5–6 basis, merus and carpus broad. Pereopod 7 ischium and dactylus ordinary. Epimeral plate 3 ordinary, bearing four or more long setae. Uropods 1–2, one or more rami with subapical stout setae or nails. Uropod 3 at least one of rami longer than peduncle; outer ramus 2‑articulate, article 2 with 2–3 apical setae. Diagnosis amended from Andrade and Senna (2020).

#### Bopyridae

Rafinesque, 1815

59535698-F115-51F1-B514-0E12BEE8F2FB

##### Parasite of

Eucalliacid shrimp (Axiidea, Eucalliacidae). Including *Zeaione everta* **gen. et sp. nov.**, there are now 20 recent species of bopyrid isopods known to parasitise axiidean shrimp worldwide as ectoparasites (Markham 2001, An et al. 2009, Boyko et al. 2017, Romero-Rodríguez and Álvarez 2025, this work) and one fossil undescribed species (Franţescu 2014) (Table 6) which could belong to either Bopyridae or Ionidae. Bopyrids on axiidean shrimp have been poorly studied when compared to those on upogebiids (see, for example, Markham (2001)) and are in need of more intensive sampling and study. Seven species of Ionidae are known from axiidean hosts; these have been reviewed by Hernáez et al. (2023).

#### Hoplopolemius

Sganga & Roccatagliata, 2016

1052E07F-FE95-5F17-8AF0-ADE2DB4B7EEB

**Type species**: *Hoplopolemius propinquus* (Richardson, 1902).
**Composition**: Four valid species. *Hoplopolemius propinquus* (Richardson, 1902), *Hoplopolemius toyoshious* (Larsen & Shimomura, 2006), *Hoplopolemius triangulatus* (Richardson, 1902) and *Hoplopolemius olo* Jóźwiak & Stępień, **sp. nov.**

##### Diagnosis

Rostrum without marginal serration. Antennule peduncle article 1 with more than one lateral apophysis; with inner flagellum multiarticulate and shorter than outer flagellum. Pereopod 1 exopod with last article elongated, having clearly less than 20 plumose marginal setae; propodus wide, not much longer than thick or the length of the carpus. Five pairs of pleopods.

#### Nesotanais

Shiino, 1968

4809E409-F43A-5F87-9231-58B9E0C6D776

**Type species**: *Nesotanais lacustris* Shiino, 1968.
**Composition**: Five valid species. *Nesotanais lacustris* Shiino, 1968, *Nesotanais maclaughlinae* Guţu & Iliffe, 1989, *Nesotanais rugula* Bamber, Bird & Angsupanich, 2003, *Nesotanais ryukyuensis* Kakui, Kajihara & Mawatari, 2010 and *Nesotanais thalassinus* Stępień, **sp. nov.**

##### Diagnosis

Diagnosis changed after Shiino (1968):

Female: Eyes present, well developed. A1 three-articled. A2 six-articled. Mxp fused at the base. Pereopods with distinct ischium. Oostegites in four pairs. Plp biramous, five pairs. U biramous, both rami with two articles.

Male: Eyes present, well developed. A1 four-articled. A2 six-articled. Ch large, propodus strongly dilated downwards to form a flange in ventral edge. P, Plp and U similar to female.

## Supplementary Material

XML Treatment for Nicon salinus

XML Treatment for Spinther bohnorum

XML Treatment for Craspedochiton zefranki

XML Treatment for Ferreiraella charazata

XML Treatment for Pycnodontochiton

XML Treatment for Pycnodontochiton sinensis

XML Treatment for Veleropilina gretchenae

XML Treatment for Laevidentalium wiesei

XML Treatment for Myonera aleutiana

XML Treatment for Apotectonia senckenbergae

XML Treatment for Metharpinia hirsuta

XML Treatment for Zeaione

XML Treatment for Zeaione everta

XML Treatment for Haploniscus bulbosus

XML Treatment for Macrostylis peteri

XML Treatment for Hoplopolemius olo

XML Treatment for Nesotanais thalassinus

XML Treatment for Laevidentalium

XML Treatment for Cuspidariidae

XML Treatment for Myonera

XML Treatment for Metharpinia

XML Treatment for Bopyridae

XML Treatment for Hoplopolemius

XML Treatment for Nesotanais

## Figures and Tables

**Figure 52a.**
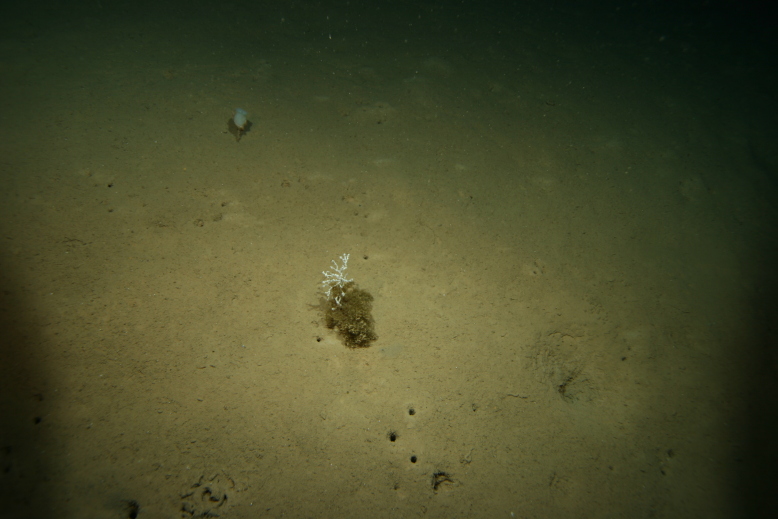
Image SS200507_25_202550 showing soft coral (Cnidaria) with organic debris in the front, stalked cup-shaped sponge (Porifera, Hexactinnelida) in the back);

**Figure 52b.**
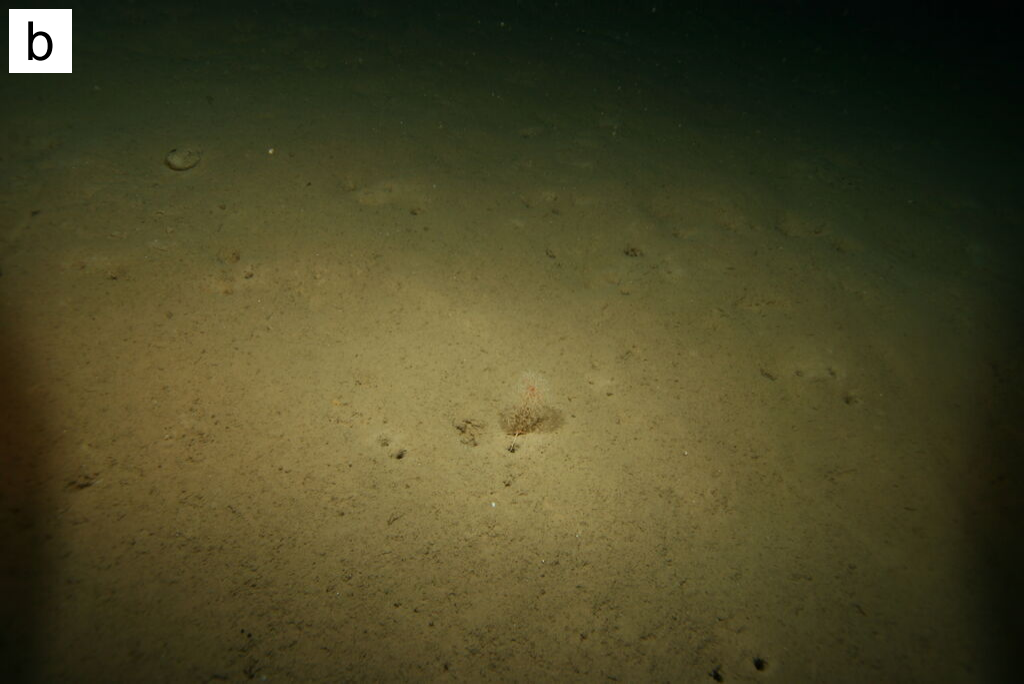
Image SS200507_25_202708 showing fine-branched coral (Cnidaria) with associated brittlestar (Ophiuroidea);

**Figure 52c.**
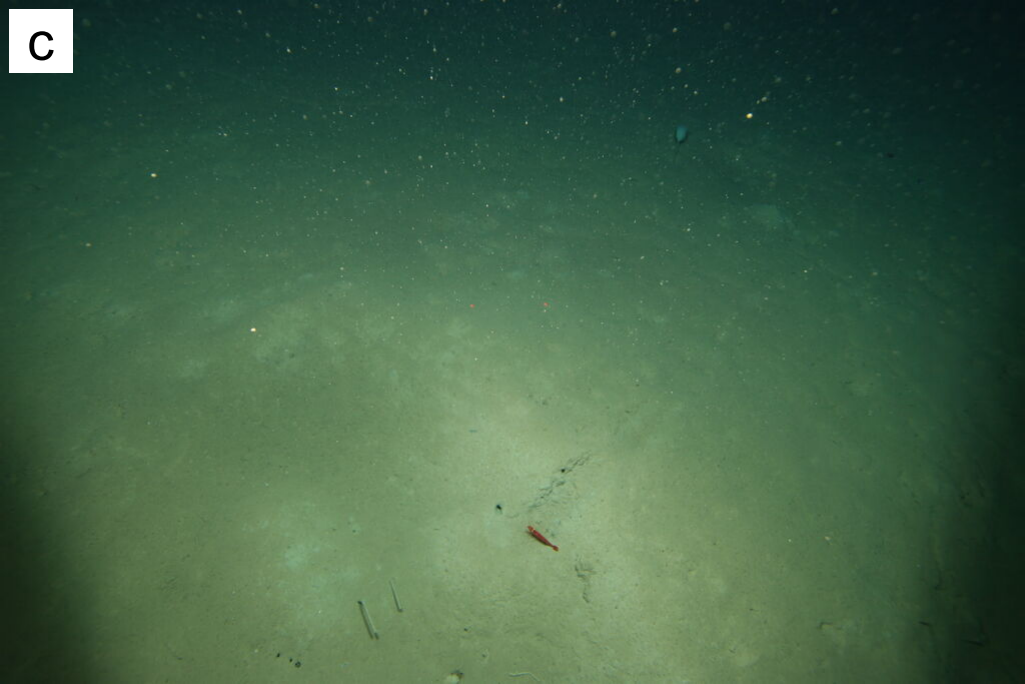
Image SS200507_25_202810 showing decapod crustacean (potentially Palaemonidae) and tubes of uncertain origin in the front, stalked cup-shaped sponge (Porifera, Hexactinnelida) in the back;

**Figure 52d.**
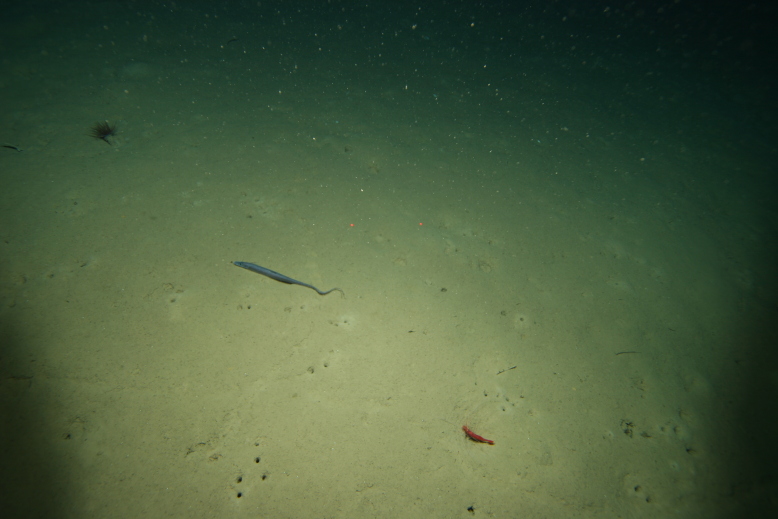
Image SS200507_25_203018 showing a decapod crustacean (potentially Palaemonidae) in the front and an eel-shaped ray-finned fish (Osteichtyes: cf. Halosauridae) in the back.

**Table 1.** Morphological comparison of nereidid species belonging to the genus *Nicon* Kinberg, 1865.

**Species**	**Longest tentacular cirri reaching to**…	**Dorsal cirrus**	**Notopodial dorsal ligules**	**Notopodial prechaetal lobes**	**Notopodial median ligule**	**Neuropodial spinigers**	**Neuropodial falcigers**
*N. ablepsia* Wang, Cheng & Wang, 2021	chaetiger 6	long, inserted in middle of ligule	sub-conical	absent	sub-conical	homogomph	heterogomph
*N. abyssalis* Hartman, 1967	chaetiger 2	shorter dorsal ligule, inserted at base of ligule	reduced, cirriform	absent	elongate in posterior chaetigers	heterogomph; homogomph?	homogomph?; heterogomph?
*N. aestuariensis* Knox, 1951	chaetiger 5 (4–6)	shorter dorsal ligules, inserted at base of ligule	conical	shorter	conical	homogomph; heterogomph	heterogomph
*N. japonicus* Imajima, 1972	chaetiger 2	longer than ligule, inserted at base of ligule; posteriorly much longer	triangular	subulate, shorter than median ligule; absent posteriorly	triangular	homogomph; heterogomph	heterogomph; pseudo-compund with bifid tips in median parapodia; a simple chaeta in posterior parapodia
*N. maculatus* Kinberg, 1865	chaetiger 5–9	extending beyond dorsal ligules	blunty conical	absent	blunty conical	homogomph	heterogomph
*N. moniloceras* (Hartman, 1940)	chaetiger 7–9 (moniliform)	slender, longer than dorsal ligule; small posteriorly	digitiform, triangular posteriorly	absent	digitiform, triangular posteriorly	homogomph; heterogomph	heterogomph
*N. orensanzi* de León-González & Trovant, 2013	chaetiger 2	short, inserted at base of ligule	longer, cirrus-like on median and posterior chaetigers	small anteriorly; absent in median and posteriorly	subulate	Homogomph; heterogomph	sesquigomph
*N. pettiboneae* de León-González & Salazar-Vallejo, 2003	chaetiger 5	longer than ligule, inserted medially on ligule	triangular	absent	triangular	homogomph	heterogomph; sesquigomph
*N. rotundus* Hutchings & Reid, 1990	chaetiger 2	extending beyond dorsal ligules	subtriangular along body	shorter	conical, triangular posteriorly	homogomph	homogomph; heterogomph
*N. yaquinae* Fauchald, 1977	chaetiger 4 (posterior dorsal cirri lost)	slender, longer than dorsal ligule; attached medially on ligule	subtriangular	absent	triangular	Homogomph; heterogomph	heterogomph
*Nicon salinus* Hernández-Alcántara & Dávila-Jiménez **sp. nov.**	chaetiger 2	short, inserted in base of ligule	longer, cirrus-like on median and posterior chaetigers	subulate, similar in length to median ligule; short in median; absent posteriorly	triangular	homogomph	homogomph

**Table 2.** Morphological characters of *Spinther* spp. Modified after Yamamoto & Imajima, 1985 and species accepted on WoRMS.

Species	Author	Type Locality	Ventrum	Parapodial extension	Neurochaetae	Notochaetae	Number of segments	Length in mm	References
*Spinther alaskensis*	Hartman, 1948	Aleutians, Alaska	papillate	present	falcate smooth	entire, except for very few, very slender bifid ones, golden and entire, about two times as long as skin folds	46–47	ca. 28	Hartman (1948); Uschakov (1950)
*Spinther arcticus*	(M. Sars, 1851)	Northern Norway	smooth	absent	falcate smooth	bifid only, distal part spread	12–24	1–9	Graff (1888)
*Spinther australiensis*	Augener, 1913	Western Australia	smooth	absent	falcate lateral tooth	bifid only	15–31	4.5–7.5	Hartman (1948)
*Spinther bohnorum*	Tilic & Rouse, sp. nov.	French Polynesia	smooth	absent	falcate lateral tooth	entire and bifid, distal part spread	7–13	0.5–1.5	this study
*Spinther citrinus*	(Stimpson, 1853)	Eastern Canada	papillate	present	falcate smooth	largely entire, a few bifid, both equally thick	30–48	11–26	Hartman (1948)
*Spinther ericinus*	Yamamoto & Imajima, 1985	Japan	smooth and segmentally ridged	present	falcate smooth	bifid only, distal part spread	27	10.1	Yamamoto and Imajima (1985)
*Spinther hystrix*	Uschakov, 1950	Eastern Russia	papillate	present	falcate smooth	entire, except for very few, very slender bifid ones, golden and entire, about five times as long as skin folds	up to 50	max. 50	Uschakov (1950)
*Spinther japonicus*	Imajima & Hartman, 1964	Japan	smooth	absent	falcate smooth	bifid and entire, the ratio is between 1:2 and 1:3	29	5	Imajima and Hartman (1964)
*Spinther oniscoides*	Johnston, 1845	Ireland	papillate	present	falcate smooth	bifid only	20–25	4–13	Hartman (1948)
*Spinther sagamiensis*	Imajima, 2003	Japan	papillate	present	falcate smooth	bifid only	14	2.7	Imajima (2003)
*Spinther usarpia*	Hartman, 1967	Antarctic Peninsula	smooth	absent	falcate smooth	bifid only, distal part not spread	20	3	Hartman (1967)
*Spinther vegae*	Augener, 1928	Bering Strait	papillate	present	falcate smooth	bifid and entire, both equal in number and equally thick	43–52	20–25	Hartman (1948)

**Table 3.** Morphological comparison of Pacific *Veleropilina* spp. Characteristics of *Veleropilina gretchenae* **sp. nov.** are noted in bold, as are shared character states for other species. Data modified from Kano et al. (2012) and including additional information on *V. oligotropha* (Rokop, 1972) from Wiklund et al. (2017) and the specimen sequenced by Chen et al. 2025 (SMF 360689).

	Locality	Depth (m)	Max. shell length (mm)	Shell width / length	Shell height / length	Shell sculpture	Position of apex	Apical cap angle	Apical cap size (μ m)	Early teleoconch sculpture	Gills	Largest ‘lateral’ radular tooth
*Veleropilina gretchenae* sp. nov.	Alaska, North-East Pacific	6465	5.2	0.77	0.38	reticulate	outside	100	280	concentric	5	3rd tooth?
*Veleropilina veleronis* (Menzies & Layton, 1963)	Mexico, East Pacific	2730–2769	2.6	0.79–0.86	0.28	reticulate	outside	90	150–200	[?]	5	3rd tooth
*Veleropilina oligotropha* (Rokop, 1972)	Hawaii, Central Pacific; Clarion-Clipperton Zone	4050–6079	3	0.80–0.84	0.26–0.28	reticulate	marginal / inside	50	210	reticulate	5	[?]
*Veleropilina capulus* (Marshall, 2006)	New Zealand, South Pacific	880–970	2.4	0.79	0.38	reticulate	outside	85	190	concentric	[?]	[?]
*Veleropilina seisuimaruae* Kano et al., 2012	Japan, West Pacific	816–841	3.15	0.80	0.32	reticulate	outside	100	220	smooth	5	3rd tooth
*Veleropilina* sp. Waren & Gofas, 1996	Mexico, East Pacific	1950	1.7	0.74	[?]	reticulate	inside	[?]	200	smooth	5	all similar

**Table 4.** Shell measurements [mm] for specimens of *Laevidentalium wiesei* Sahlmann, 2012. Abbreviations: LTot, total shell length; ApL, ventral aperture length (= ApH of Shimek and Moreno (1996)); ApW, ventral aperture width; arc, maximum perpendicular distance from anterior (concave) face to a straight line connecting dorsal and ventral apertures; Larc, distance along connecting dorsoventral line from dorsal (apical) end to the point where arc was measured; NA, not available; n, sample size for measurements; SE, standard error of the mean.

Specimen	Anemone present	LTot	ApL	LTot/ApL	ApW	ApW/ApL	arc	Larc
HNC 43947	yes	38.5	5.4	7.13	5.9	1.09	3.5	17.2
HNC 82015	no	43.8	5.2	8.42	5.9	1.13	3.7	23.2
SMF 366425	yes	30.5	3.3	9.24	3.7	1.12	1.1	16.4
SMF 366426	yes	45.6	6.7	6.81	7.4	1.10	2.7	18.7
SMF 373200 (shell broken)	yes	NA	5.8	NA	6.2	1.07	NA	NA
SMF 374281 (specimen 1)	no	39.4	4.9	8.04	5	1.02	5.6	21.9
SMF 374281 (specimen 2)	yes	44.3	6.5	6.82	7.1	1.09	3.3	18.9
n		6	7	6	7	7	6	6
Mean		40.4	5.4	7.7	5.9	1.1	3.3	19.4
SE		2.3	0.4	0.4	0.5	0.0	0.6	1.1

**Table 5.** The characters differing between *Apotectonia senckenbergae* Momtazi & Riehl, **sp. nov.** and the type species of the genus *A. heterostegos* Barnard & Ingram, 1990. Character abbreviations: A1 – Antenna 1; A2 – Antenna 2; Art – Article; C – Coxa; Ep – Epimeron; Flag – Flagellum; Mxp – Maxilliped; P – Pereopod; U – Uropod. Other abbreviations: n.d. – not documented; s.f. – setal formula.

Species\ characters	*A. senckenbergae*	*A. heterostegos* (Holotype)	*A. heterostegos* (Juvenile)
Length [mm]	15	11.7	12.4
Ep1-3 s.f.	0+2+3	1+3+4	1+2+3
A1/A2 length	1.06	1.5	n.d.
A1 Flag Art	5	6	4
Mxp inner plate	With plumose setae	Without plumose setae	n.d.
Propodus of P1	2 median + 1 distal robust setae	1 distal robust seta	n.d.
C1	1 distoventr. + 1 superficial setae	1 distoventral + 1 superficial setae	n.d.
C2	1 distoventr. + 3 superficial setae	2 distoventral + 4 superficial setae	n.d.
C3	1 distoventr. + 1 superficial setae	2 distoventr. + 1 superficial +1 ventral setae	n.d.
Inner margin of outer ramus in U2	Serrated with two robust setae	asetose	n.d.
P5 carpus posterior margin	with 1 pair of setae	with 3 pairs of setae	n.d.

**Table 6.** List of all known bopyrids (Bopyridae, Keponinae and Pseudioninae) found on hosts in Decapoda, Axiidea (host and parasite taxonomy updated from Markham (2001)). Markham (2001), Table 1, listed the axiidean *Callichirus tyrrhea* [*sic*] (= *Gilvossius tyrrhenus* (Petagna, 1792)) as a host of *Gyge branchialis* Cornalia & Panceri, 1861 (Pseudioninae) in the North Sea, purportedly based on data from Le Sueur (1954), but this is incorrect. Le Sueur (1954), p. 216, listed *Upogebia* sp. as the only host for this bopyrid and in Markham (2001), Table 2, the only species of bopyrid listed as being found on this host was *Ione thoracica* (Montagu, 1808) (Ionidae). There are no records of *G. branchialis* occurring on an axiidean host.

Bopyrid Species	Bopyrid Subfamily	Host Species - Family	Locality	References
*Acrobelione reverberii* (Restivo, 1970)	Pseudioninae	*Necallianassa truncata* (Giard & Bonnier, 1890) - Callianassidae	Italy	Markham (2001), Boyko et al. (2017)
*Castrione digiticaudata* Markham, 1995	Keponinae	*Marcusiaxius wamsoi* Poore, 1997 - Micheleidae	Irian Jaya (Papua)	Markham (2001)
*Castrione longicaudata* Brasil Lima, 1980	Keponinae	*Marcusiaxius lemoscastroi* Rodrigues & de Carvalho, 1972 - Micheleidae	Brazil	Markham (2001)
*Castrione longicaudata* Brasil Lima, 1980	Keponinae	*Meticonaxius* sp. - Micheleidae	Brazil	Markham (2001)
*Zeaione everta* **gen. et sp. nov.**	Pseudioninae	*Eucalliaxiopsis aequimana* (Baker, 1907) - Eucalliacidae	Victoria, Australia	herein
*Gigantione elconaxii* Markham, 1994	Pseudioninae	*Eiconaxius* sp. – Axiidae	New Caledonia	Markham (2001)
*Gigantione pikei* Page, 1985	Pseudioninae	*Axiopsis* sp. - Axiidae	New Zealand	Markham (2001)
*Gigantione uberlackerae* Adkison, 1984	Pseudioninae	*Paraxiopsis* sp. - Axiidae	Gulf of Mexico	Markham (2001)
*Ionella agassizi* Bonnier, 1900	Pseudioninae	*Neotrypaea uncinata* (H. Milne Edwards, 1837) - Callianassidae	Chile	Markham (2001)
*Ionella compressa* (Shiino, 1964)	Pseudioninae	*Neocallichirus jousseaumei* (Nobili, 1904) - Callianassidae	Japan	Markham (2001) (as *Pseudione compressa*), Boyko et al. (2017)
*Ionella compressa* (Shiino, 1964)	Pseudioninae	*Paratrypaea bouvieri* (Nobili, 1904) - Callianassidae	Japan	Boyko et al. (2017)
*Ionella fimbriata* Romero-Rodríguez & Álvarez, 2024)	Pseudioninae	*Neocallichirus grandimana* (Gibbes, 1850) -Callichiridae	Veracruz, Mexico	Romero-Rodríguez and Álvarez (2025)
*Ionella maculata* Markham 1994	Pseudioninae	“*Callianassa*” sp. - Callianassidae(?)	New Caledonia	Markham (2001)
*Ionella murchisoni* Danforth, 1970	Pseudioninae	“*Callianassa*” sp. - Callianassidae(?)	Hawaii	Danforth (1970), Markham (2001)
*Progebiophilus elongatus* An, Williams & Yu, 2009	Pseudioninae	*Neotrypaea japonica* (Ortmann, 1891) - Callianassidae	Shandong Province, China	An et al. (2009)
*Pseudione borealis* Caspers, 1939	Pseudioninae	*Callianassa marchali* Le Loeuff & Intes, 1974) - Callianassidae	Ivory Coast	Markham (2001)
*Pseudione borealis* Caspers, 1939	Pseudioninae	*Callianassa subterranea* (Montagu, 1808) - Callianassidae	Helgoland, Germany; Bay of Biscay	Markham (2001)
*Pseudione callianassae* Kossmann, 1881	Pseudioninae	*Necallianassa truncata* (Giard & Bonnier, 1890) - Callianassidae	Italy	Boyko et al. (2017)
*Pseudione hanseni* Nierstrasz & Brender à Brandis, 1923	Pseudioninae	*Paraxiopsis brocki* (De Man, 1888) - Axiidae	Indonesia	Markham (2001)
*Pseudione longicauda* Shiino, 1937	Pseudioninae	*Neotrypaea harmondi* (Bouvier, 1901) - Callianassidae	Japan	Boyko et al. (2017)
*Pseudione longicauda* Shiino, 1937	Pseudioninae	*Neotrypaea japonica* (Ortmann, 1891) - Callianassidae	Japan	Markham (2001), Boyko et al. (2017)
*Pseudione longicauda* Shiino, 1937	Pseudioninae	*Neotrypaea petalura* (Stimpson, 1860) - Callianassidae	Hong Kong	Markham (2001), Boyko et al. (2017)
*Pseudione longicauda* Shiino, 1937	Pseudioninae	*Paratrypaea bouvieri* (Nobili, 1904) - Callianassidae	Japan	Boyko et al. (2017)
*Pseudione murawaiensis* Page, 1985	Pseudioninae	*Balsscallichirus balssi* (Monod, 1933) - Callianassidae	New Zealand	Markham (2001)
*Robinione brattstroemi* (Stuardo, Vega & Cespedes, 1986)	Pseudioninae	*Neotrypaea uncinata* (H. Milne Edwards, 1837) - Callianassidae	Chile	Markham (2001), Boyko et al. (2017)
*Robinione overstreeti* (Adkison & Heard, 1995)	Pseudioninae	*Callichirus islagrande* (Schmitt, 1935) - Callichiridae	Gulf of Mexico from west coast of Florida to Texas, USA	Boyko et al. (2017)
*Robinione overstreeti* (Adkison & Heard, 1995)	Pseudioninae	*Callichirus santarosaensis* Sakai & Türkay, 2012 - Callichiridae	Tabasco, Mexico	Boyko et al. (2017)
Fossil Bopyridae or Ionidae gen. et sp. indet. “infected with boparid [sic] isopod on both sides”	Indeterminate; could also belong to Ionidae	*Axiopsis sampsonumae* Franţescu, 2014 - Axiidae	Texas	Franţescu (2014)
